# Molecular evolution of the vertebrate TLR1 gene family - a complex history of gene duplication, gene conversion, positive selection and co-evolution

**DOI:** 10.1186/1471-2148-11-149

**Published:** 2011-05-28

**Authors:** Yinhua Huang, Nicholas D Temperley, Liming Ren, Jacqueline Smith, Ning Li, David W Burt

**Affiliations:** 1 Division of Genetics and Genomics, The Roslin Institute and R(D)SVS, University of Edinburgh, Roslin, Midlothian EH25 9PS, UK; 2 MRC Human Genetics Unit and the Division of Cancer Research, Institute of Genetics and Molecular Medicine, The University of Edinburgh, Crewe Road South, Edinburgh, EH4 2XR, UK; 3 State Key Laboratory for Agrobiotechnology, China Agricultural University, Beijing 100094, People's Republic of China

**Keywords:** Gene Duplication, Gene Conversion, Positive Selection, Co-evolution, TLR1 gene family

## Abstract

**Background:**

The Toll-like receptors represent a large superfamily of type I transmembrane glycoproteins, some common to a wide range of species and others are more restricted in their distribution. Most members of the Toll-like receptor superfamily have few paralogues; the exception is the TLR1 gene family with four closely related genes in mammals TLR1, TLR2, TLR6 and TLR10, and four in birds TLR1A, TLR1B, TLR2A and TLR2B. These genes were previously thought to have arisen by a series of independent gene duplications. To understand the evolutionary pattern of the TLR1 gene family in vertebrates further, we cloned the sequences of TLR1A, TLR1B, TLR2A and TLR2B in duck and turkey, constructed phylogenetic trees, predicted codons under positive selection and identified co-evolutionary amino acid pairs within the TLR1 gene family using sequences from 4 birds, 28 mammals, an amphibian and a fish.

**Results:**

This detailed phylogenetic analysis not only clarifies the gene gains and losses within the TLR1 gene family of birds and mammals, but also defines orthologues between these vertebrates. In mammals, we predict amino acid sites under positive selection in TLR1, TLR2 and TLR6 but not TLR10. We detect co-evolution between amino acid residues in TLR2 and the other members of this gene family predicted to maintain their ability to form functional heterodimers. In birds, we predict positive selection in the TLR2A and TLR2B genes at functionally significant amino acid residues. We demonstrate that the TLR1 gene family has mostly been subject to purifying selection but has also responded to directional selection at a few sites, possibly in response to pathogen challenge.

**Conclusions:**

Our phylogenetic and structural analyses of the vertebrate TLR1 family have clarified their evolutionary origins and predict amino acid residues likely to be important in the host's defense against invading pathogens.

## Background

The innate immune response is the first line of defense against invading pathogens and is required for an efficient adaptive immune response. Toll-like receptors (TLRs) play a crucial role in innate immunity by recognizing specific pathogen-associated molecular patterns including lipoproteins, lipopeptides, lipopolysaccharide, flagellin, dsRNA, ssRNA and CpG DNA motifs (Akira and Takeda 2004; West et al. 2006). Phylogenetic analysis has classified vertebrate TLRs into six major gene families [1-3]: TLR1, TLR3, TLR4, TLR5, TLR7 and TLR11. Most vertebrates have a single gene for each TLR gene family [1,2]; the major exception is the TLR1 gene family, which appears to have multiple, paralogous genes. In mammals, the TLR1 gene family consists of four members: TLR1, TLR2, TLR6 and TLR10. In birds, there are also four paralogues with multiple names in the literature, two TLR1-like genes: TLR1La (TLR1 type 1, TLR1.1, TLR1/6/10 and TLR16) and TLR1Lb (TLR1 type 2 and TLR1.2), and two TLR2-like genes: TLR2a (TLR2 type 1 and TLR2.1) and TLR2b (TLR2 type 2 and TLR2.2). More distantly related members of the TLR1 gene family are TLR14 and TLR15 found in mammals and birds, respectively. Phylogenetic and genome analyses suggest that TLR1- and TLR2-like genes diverged from a common ancestor early in vertebrate evolution [1,2]. The genes of the TLR1 gene family are usually in tandem in both avian and mammalian genomes and are likely to be the product of successive rounds of tandem gene duplications from an ancestral gene.

Gene duplication followed by sequence divergence is one of the most important mechanisms for generating new genes with novel functions [4,5]. Genetic drift and positive selection have both played a role in the fixation and early evolution of duplicated genes [6-9]. Many studies have classified genes showing evidence of positive selection [10], where genes with roles in host defense/immunity, chemosensory perception and reproduction are particularly over represented. Analysis of the non-synonymous/synonymous substitution ratio revealed the presence of both strictly conserved and rapidly evolving region in the TLR-related genes in primates[11-13]. A recent comparative sequence analysis from a panel of domestic chickens and wild jungle fowl suggest that TLRs show positive selection [14]. More specifically, an earlier study suggested that the leucine-rich repeats of chicken TLR1La and TLR1Lb may have been subject to positive selection. In contrast, the sequences of all other avian TLRs were under purifying selection [15], which appears to be the trend for all TLRs in mammals [1].

In mammals, TLR2 dimerizes with either TLR1 to recognize microbial triacyl lipoproteins or TLR6 to recognize diacyl lipopeptides found in Mycoplasma, lipoteichoic acid of Gram-positive bacteria or Zymosan of yeasts [16,17]. Recently, human TLR10 has also been shown to dimerize with TLR2 and to recognize triacyl lipoproteins [18], although signaling by this complex appears to differ from the other TLR1/6/2 complexes. In chicken [19,20] expression of TLR1La or b, or TLR2a or b alone fail to be activated by exogenous agonists; however heterodimers of all four TLR1/TLR2 combinations were activated by both diacyl (Malp-2) and triacyl (Pam3) lipopeptides, with the exception of TLR2a/TLR1Lb, which was activated by Pam3 but not Malp-2. In addition, TLR2a/TLRL1b was also activated by peptidoglycan. The interaction of these TLR1- and TLR2-like proteins in the absence of any agonist was also shown by immunoprecipitation. Thus, chicken TLR1-like proteins interact with TLR2-like proteins and recognize agonists identical to those used in mammals by heterodimers between TLR2 and TLR1, 6 or 10.

A recent study [21] showed sequence divergence in the C-terminal regions of TLR1 and TLR6 in mammals to be severely limited by gene conversion. This 300 amino acid region encodes the LRR16-19 motifs, the C-terminal cap motif, the transmembrane domain and most of the intracellular TIR domain. The need to co-evolve with binding partners, such as MyD88 and TIRAP may explain the selective advantage for gene conversion in the TIR domains of TLR1 and TLR6. A recent report also found evidence for gene conversion in the N- and C-terminal regions of the duplicated TLR2A and TLR2B genes found in the genomes of chicken and zebra finch [22]. Thus, gene conversion may be a general mechanism that limits the divergence of critical regions in these proteins, while allowing other domains, such as the ligand binding domain, to evolve diversified functions.

The aim of this work was to seek possible explanations for the apparent multiple, independent gene duplications proposed to account for the diversity and size of the TLR1 gene family [23]. From a phylogenetic analysis of many avian and mammalian TLR1-like and TLR2-like genes we have defined the likely role of positive selection, gene duplication, gene conversion and gene co-evolution in the evolutionary dynamics of this family. Our phylogenetic and structural analysis of vertebrate TLR1 gene family members also predicts amino acid residues likely to be important in the primary function of TLRs in the host's defense against invading pathogens.

## Methods

### Nucleic acid extraction

Total RNA from turkey spleen was extracted by TRIZOL (Invitrogen, Paisley, UK) according to the manufacture's protocol. The sample was re-suspended in a final volume of 50 μl of RNAse-free water. A routine phenol/chloroform extraction method was used to purify turkey genomic DNA. Both the quantity and quality of total RNA and genomic DNA were assessed at OD_260 _and OD_280 _using a NanoDrop ND-1000 spectrophotometer (NanoDrop Technologies, Wilmington, USA).

### DNA cloning and sequencing

BAC clones containing TLR1A/TLR1B or TLR2A/TLR2B sequence were screened by 4D, two-step PCR from a *HindIII*, female Beijing duck genomic BAC library with inserts from vector pIndig-5 (Epi-Center, USA). The primers used to screen the duck BAC library, were designed from the orthologous sequences in other species such as chicken, pig, human or the partial sequence of duck (AY838880) (Additional file 1, Table S1) [24]. Positive clones were confirmed by sequence analysis of PCR products, then subcloned into pUC118 and sequenced by the shotgun method, (with an average of 6.38Х coverage) using Big Dye Terminator V3.1 (ABI, Foster city, USA) on an ABI3130X Sequencer (ABI, Columbia, MD). Base calling and quality assessment were performed using Phred [25,26], Assembly was performed using Phred and Consed [27,28]. Sequence identity between duck and chicken genomic sequences was analyzed using zPicture [29].

To clone the TLR1 gene family sequences from turkey, PCR reactions were carried out in 100ųl total volume, containing 100 ng turkey genomic DNA, 50 mM Tris/HCl (pH8.3), 10 mM KCl, 50 mM (NH_4_)_2_SO_4_, 2.0 mM MgCl_2_, 200 mM dNTP, 10 U FastStart Taq DNA polymerase (Roche, Mannheim, Germany), and 100 pmol each primer designed from the orthologous sequence in chicken and duck (Additional file 1, Table S1). PCR reaction conditions were: denaturing for 5 min at 94°C, followed by 94°C for 30s, annealing for 30s, and 72°C for 1 min per 1 kb, 35 cycles, with a final 7 min elongation step at 72°C. PCR products were purified from agarose gels with the QIAEXII gel extraction kit (Qiagen, Hilden, Germany), ligated into pCR4-TOPO (Invitrogen, Carlsbad, USA), transformed into *E. coli *TOP10 competent cells and plasmid DNA was sequenced using T3 or T7 universal primer. In addition, the sequences at the 3`end of TLR2A and TLR2B were cloned from turkey spleen RNA by 3`-RACE using the FirstChoice RLM-RACE kit (Ambion, Austin, USA) and confirmed against the genomic sequence of turkey.

### Sequence analysis

Sequences from the TLR1 gene family were collected from the National Center for Biotechnology Information (NCBI) (http://www.ncbi.nlm.nih.gov) using the Blast search program [30], retrieved through searches in ENSEMBL (http://www.ensembl.org/index.html) and identified by Blat searches on the UCSC Genome Bioinformatics database (http://genome.ucsc.edu/). The alignments of amino acid and nucleotide sequence used for the analysis of gene conversion and the construction of phylogenetic trees, and for the calculation of percentage identities were made using ClustalX (version 2.0) [31]. Sequence homologies were displayed using JalView 2.5.1[32].

### Gene conversion analysis

Recombination analysis was performed using GENECONV (version 1.8) with default settings [33]. The more conservative and accurate p-values from permutations (10,000 pseudo-replicates) were used and p-values (p < 0.05) from global fragments were corrected for multiple comparisons. The aligned sequences were examined for possible gene conversion events using the sliding-window genetic diversity plot (Simplot) (version 3.5.1) by bootscan with a neighbor-joining (NJ) tree, maximum-likelihood distance model, with 1,000 pseudo-replicates in a 200 bp window and with a step size of 20 bp [34].

### Phylogenetic analysis

Molecular phylogenies based on sequence alignments are only as accurate as the alignment data from which they are produced; consequently it is important that the alignment data quality is determined [35]. In order to assess the alignment's tree like structure, likelihood mapping was carried out using Tree-Puzzle (version 5.2) [36], which generally showed alignments to have strong phylogenetic signals with 89-94% of quartets (1,000 pseudo-replicates) support a unique phylogeny (*data not shown*).

Phylogenetic relationships among orthologues and paralogues of TLR1 and TLR2 subfamilies were carried out on the protein alignment using PHYML (version 2.4.4) [37]. After selection of the best substitution model for the aligned protein or DNA sequence by comparing the likelihood values of different models, two rounds of analysis were used to search the maximum-likelihood trees under the JTT model of molecular evolution with 4 substitution rates classes for protein sequences or HKY85 for DNA sequences. The phylogenetic tree, the proportion of invariable sites and Gamma distribution for protein sequence or transition/transversion ratio for DNA sequence parameters were estimated under the above model in the first round. Then, the robustness of the inferred tree was assessed using bootstrapping (1,000 pseudo-replicates) by fixing the proportion of invariable sites and Gamma distribution or transition/transversion parameters in the second round. Gene Trees were displayed using FigTree (version 1.3.1) (http://tree.bio.ed.ac.uk/).

### Estimation of the time of gene duplication events

The fossil calibration time of the orthologous TLRs was inferred from species divergence times obtained from the TIMETREE web site (http://www.timetree.org/index.php). Divergence times of gene conversion and duplication were estimated under global and local clock models with two runs using CODEML or BASEML within the PAML package (version 4) [38]. Firstly, the "kappa" (the transition/transversion rate ratio) and "omega" (the non-synonymous/synonymous rate ratio) for CODEML or "alpha" (gamma-distributed rate) for BASEML were estimated, and then the divergence times were estimated with fixed values for "kappa" and "omega" for CODEML or "alpha" for BASEML.

### Maximum likelihood tests of positive selection

Multiple sequence alignment of coding sequences were created using DAMBE [39], first on the translated DNA sequences and then the nucleotide sequence of codon triplets was recovered by back translation. DAMBE was also used to check for saturation of nucleotide substitutions using a plot of the number of transitions and transversions for each pairwise comparison against the genetic distance calculated with the F84 model of nucleotide substitution [40], which allows different equilibrium nucleotide frequencies and a transition rate-transversion rate bias. Multiple sequence alignments not showing saturation of nucleotide substitutions were then analysed further using CODEML[38]. A series of nested likelihood ratio tests (LRTs) were performed using CODEML to investigate whether some sites were under positive selection in each TLR group. The first test compared M1A (nearly neutral: p0, p1, ω0 < 1, ω1 = 1, NS sites = 1) against M2A (positive selection: p0, p1, p2, ω0 < 1, ω1 = 1, ω2 < 1, NS sites = 2). The second test compared M7 (beta: p, q, NS sites = 7) with M8 (beta & ω: p0, p1, p, q, ω_s _> 1, NS sites = 8). The third test was between M8A (beta & ω_s _= 1: fix omega = 1, omega = 1, NS sites = 8) and M8.

### Intra- and intermolecular co-evolution analysis

To identify co-evolutionary patterns, we used the parametric method based on correlated evolutionary patterns among amino-acid sites [41] implemented in CAPS (Version 1.0) [42]. The probabilities and significance of the correlated evolutionary patterns among amino-acid sites were estimated using a large number (10,000) of random samplings and a small α value (0.001) to minimize a false-positive rate (type I error). CAPS implements the step-down permutation procedure [41] to correct for multiple testing. The scores for the amino-acid substitutions were obtained using the appropriate blocks substitution matrix (BLOSUM80) [43], depending on the similarity of the protein sequences. Possible interactions were also filtered based on cellular compartments: extracellular, transmembrane and cytoplasmic. All amino-acid sites reported in the co-evolutionary analyses refer to the positions in the protein sequences of human TLR1, TLR2, TLR6 and TLR10.

### Visualization of co-evolutionary networks

Cytoscape (Version 2.7.0) [44] was used to visualize the co-evolutionary networks identified by CAPS, and to generate the networks of correlation between co-evolving amino acids. The correlation coefficients generated in CAPS were used to determine the color of the lines between nodes (amino-acid residues).

### Protein structure analysis

The Uniprot protein database (http://www.uniprot.org) was used as an initial resource to define the protein domain locations of specific amino acid residues in TLR proteins. If there were no annotated protein domains, these were determined by aligning genes with their human orthologues, and then inferring domains from Uniprot. To examine potential functional or structural significance of specific amino acid residues, their location was mapped onto the known or predicted three-dimensional structures of TLR proteins. The known structures of human TLR1/2/6 (Jin et al. 2007) were used directly and as templates to predict the structures of human TLR10 using CPHmodels (version 3.0), a protein homology modeling server [45]. Template recognition was based on profile-profile alignment guided by secondary structure and exposure predictions. The atomic coordinates for the PDB models of human TLR1 and TLR2 proteins suggested the secondary structure of human TLR10 was similar to human TLR1 (E value 7.0 × 10^-89^). Amino acid residues were mapped onto these structures using the protein BLAST alignment of each group, and a PyMOL script file was generated for visualization using PyMOL (version 1.1) (http://www.pymol.org).

## Results

### Isolation of Toll-like receptor-like 1 and 2 genes from the turkey and duck genomes

The coding sequences for chicken and zebra finch members of the TLR1 gene family were extracted from sequence databases. The coding sequences for turkey TLR1-like and TLR2-like genes were cloned from genomic DNA by PCR using primers based on the chicken orthologues. The 3`-end mRNA sequences of turkey TLR2A and TLR2B were cloned by 3`-RACE with the primers shown in Additional file 1, Table S1. Duck BAC clones containing genes for TLR1A, TLR1B, TLR2A and TLR2B were isolated by four-dimensional, two-step PCR and sequenced (see *Materials and Methods*). A 67,954 bp BAC clone (DBS1203G04, GenBank accession FJ477859) contained both TLR1A and TLR1B genes, and a 75,080 bp BAC clone (DBS1405N01, GenBank accession FJ477862) contained TLR2A and TLR2B (Additional file 2). The coding sequences of the TLR1 gene family in duck and turkey are encoded by single exons, as found in the chicken. Sequence alignment revealed the average percentage identity for pair-wise comparisons between the orthologues in chicken/duck to be 85.3 ± 1.5% for nucleotide and 79.8 ± 2.5% for amino acid sequences, and in chicken/turkey to be 93.0 ± 1.2% for nucleotide and 90.3 ± 2.2% for protein sequences. Sequences for the duck and turkey TLR1 genes were submitted to GenBank (Accessions FJ477857-FJ477862).

### Multiple gene conversion events during the evolution of the TLR1 gene family

Maximum-likelihood (ML) trees for TLR1-like (Additional file 3, Figure S2A) and TLR2-like (Additional file 3, Figure S2B) genes were initially constructed using all available vertebrate coding sequences (Additional file 4). However, the results suggested that paralogues were more similar than orthologues. Such a result could be explained if one or more gene conversion events between paralogues had occurred during their evolution. To establish whether gene conversion has occurred between paralogues and to identify its boundaries, we analyzed the sequences of three groups using GENECONV [33] and SIMPLOT [34]. Group 1: avian TLR1A/TLR1B (Additional file 5, Figure S3A), group 2: avian TLR2A/TLR2B (Additional file 5, Figure S3B) and group 3: mammalian TLR1/TLR6 (Additional file 5, Figure S3C). No gene conversion was detected between mammalian TLR1/TLR10 or TLR6/TLR10 sequences (*data not shown*). The results with GENECONV (Table 1) were highly significant (p values < 10^-27^-10^-66^) between the paralogues of the same species in groups 1, 2 and 3 providing evidence of gene conversion between ancestral sequences of paralogues. SIMPLOT also showed high sequence identity between the paralogues in these groups (Additional file 6). To examine whether gene conversion had occurred outside the coding region, we re-examined multiple alignments including 3`-UTRs using SIMPLOT. The boundaries of gene conversion were the same as those without the 3`-UTR except for *Bos taurus *TLR1/6 genes (Additional file 7), which extended into the 3'-UTR for ~200 bp.

**Table 1:** Gene conversion analysis for the TLR1 gene family using coding sequences from birds and mammals

Group	Sequence names	BC KA P-value	N	Central	C	Number of Polymorphisms	Number of Differences	Total Differences
Avian TLR1A/B	GgalTLR1A:GgalTLR1B	3.61E-51	1-1177	--	1178-2374	301	16	415
	MgalTLR1A:MgalTLR1B	2.46E-50	1-1177	--	1178-2387	307	26	411
	AplaTLR1A:AplaTLR1B	1.47E-43	1-1190	--	1191-2397	308	38	432
	TgutTLR1A:TgutTLR1B	3.20E-60	1-1186	--	1187-2466	335	1	354
Avian TLR2A/B	GgalTLR2A:GgalTLR2B	3.16E-40	63-713	714-1290	1291-2375	418	2	287
	MgalTLR2A:MgalTLR2B	3.62E-33	47-713	714-1328	1329-2385	428	7	275
	AplaTLR2A:AplaTLR2B	2.36E-37	34-725	726-1284	1285-2371	445	22	294
	TgutTLR2A:TgutTLR2B	4.24E-39	42-718	719-1284	1285-2328	420	14	296
Mammalian TLR1/6	HsapTLR1;HsapTLR6	1.75E-32	1-1315	--	1316-1960	303	13	529
	PtroTLR1;PtroTLR6	9.80E-34	1-1315	--	1316-2235	405	35	525
	PpygTLR1;PpygTLR6	2.15E-32	1-1323	--	1324-2235	403	33	514
	CjacTLR1;CjacTLR6	2.45E-36	1-1315	--	1316-2231	402	21	504
	MmulTLR1;MmulTLR6	5.10E-38	1-1315	--	1316-2202	391	22	520
	RnorTLR1;RnorTLR6	1.12E-66	1-1332	--	1334-2225	391	5	590
	MmusTLR1;MmusTLR6	4.09E-62	1-1311	--	1312-2230	402	12	585
	BtauTLR1;BtauTLR6	1.30E-38	1-1332	--	1333-2403	475	16	473
	SscrTLR1;SscrTLR6	2.48E-37	1-1308	--	1309-2340	460	29	495
	EcabTLR1;EcabTLR6	8.00E-27	1-1315	--	1316-2234	404	48	529
	CfamTLR1;CfamTLR6	9.51E-40	1-1315	--	1316-2235	405	16	506
	EeurTLR1;EeurTLR6	1.91E-52	1-1308	--	1309-2235	407	14	550

The boundaries of gene converted regions were based on GENECONV analysis with mismatches allowed (/g1 = 1). The avian species are chicken, turkey, duck and zebra finch and the mammalian species are cattle, dog, marmoset, horse, hedgehog, human, rhesus monkey, mouse, orangutan, chimpanzee, rat and pig. The positions of fragments are given with respect to the aligned sequences of avian TLR1A/B, avian TLR2A/B or mammalian TLR1/6. The gene-converted fragments of the TLR1 gene family members are the C-terminal regions of avian TLR1A/B, avian TLR2A/B, mammalian TLR1/6, and the N-terminal regions of avian TLR2A/B. "BC KA p-values": Bonferroni-corrected KA (BLAST-like) P values. "Number of Polymorphisms", "Number of Differences" and "Total Differences" represents the number of polymorphic sites within gene-converted fragments of each group, number of mismatched sites between gene-converted paralogous fragments from the same species and total number of mismatched sites between aligned paralogous sequences from the same species, respectively.

We divided the sequences of groups 1, 2 and 3 into regions predicted to have undergone gene conversion or not (Table 1). Amino acid sequences encoded by these regions were used to create separate ML phylogenies using PHYML [37] for group 1, 2 or 3 genes. A TLR1 or TLR2 orthologue from zebrafish (*Danio rerio*) was used as an outgroup gene to root these trees. The ML trees of group 1, 2 and 3 genes calculated using the gene conversion free regions (Additional file 8, Figure S6A, Additional file 9, Figure S7B, Additional file 10, Figure S8A) were as expected from the species tree. In contrast, using group 1, 2 and 3 sequences in the regions predicted to be susceptible to gene conversion, showed paralogues to be more similar to each other than the corresponding orthologues (Additional file 8, Figure S6B, Additional file 9, Figure S7A, S7C). For most species, except primates, group 3 paralogues were more similar than orthologues and clustered together in TLR1/6 pairs within each species (Additional file 10, Figure S8B). However, for most primates, except the common marmoset (*Callithrix Jacchus*), we find orthologues to be more similar, indicating the last gene conversion event occurred prior to the *Callithric Jacchus/Macaca *mulatta split, 45 million years ago (Mya).

The sequence identities in specific regions between orthologues and paralogues are summarized in Additional file 1, Table S2. For groups 1 and 3 sequence identities in the C-terminal regions of paralogues (within the same species) were significantly greater than the corresponding identities between orthologues (p < 0.05). In contrast, sequence identities between the N-terminal regions of paralogues were lower than orthologues (p < 0.05). For group 2 sequence identities of N- and C-terminal regions of paralogues were higher than orthologues (p < 0.05). However, the central region was more conserved between orthologues (p < 0.05).

Amino acid sequences that remained invariant throughout evolution or became identical through convergent evolution are less likely to be encoded by the same codons than sequences that became identical after gene conversion[21]. To discriminate between these possibilities as the cause for the greater sequence identity between paralogues, we compared the codon usage of conserved (and identical) amino acids in group 1, 2 and 3 genes. Overall, paralogues share 71.5%, 82.5% and 62.0% amino acid identity in groups 1, 2 and 3, respectively (Additional file 1, Table S2). Analysis of codon usage for conserved amino acids (Additional file 1, Table S3) in groups 1 and 3 indicated that identical codons were more frequently used in the C-terminal (96-99%) than in the N-terminal (57-66%) region. In group 2 identical codons in both N- and C-terminal regions were used more frequently (99%) than those in the central region (56%).

Taken together these results support a process of gene conversion rather than gene convergence, as the major cause for increased sequence identity between paralogues.

### Identification of TLR2 pseudogenes in mammalian genomes

Two functional copies of TLR2 (TLR2A and TLR2B) were found in the genomes of chicken, turkey, duck and zebra finch, extending the earlier observations [2]. To examine the evolutionary history of the TLR2 gene duplication, we searched all available vertebrate genome sequences for possible functional or non-functional sequences. Two functional copies of TLR2 were also detected in *Xenopus tropicalis *but only one functional gene was identified in 5 species of fish and 14 species of mammals. Previous work [1] detected a TLR2 pseudogene (TLR2P) in tandem with the functional copy in the genomes of opossum, dog and human. We have extended this observation to include an additional five mammalian genomes: chimpanzee, orangutan, rhesus monkey, marmoset and horse (Additional file 4). The identification of multiple TLR2 genes and pseudogenes on many mammals and *Xenopus tropicalis *suggests an early gene duplication event in the history of the TLR2 gene family. In most cases, mammalian TLR2 functional genes and pseudogenes formed distinct clusters (Additional file 11) as expected from the species tree. The exception was the horse, where its functional gene was more similar to its pseudogene, suggesting a recent gene conversion event between these paralogues. This was supported by finding high sequence homology between these paralogues using SIMPLOT (Additional file 6, Figure S4D).

### A complex history of gene duplication and gene conversion during the evolution of the vertebrate TLR1 gene family

**Figure 1:**
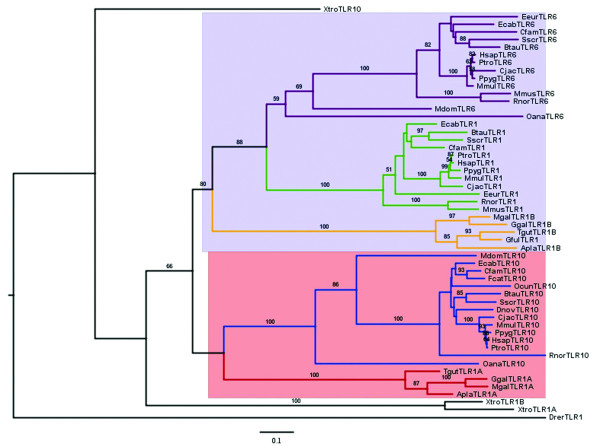
**ML tree of the TLR1 subfamily based on vertebrate N-terminal amino acid sequences**. The tree is rooted with *Danio rerio *and the sequences are listed in Additional file 4. The bootstrap values of 1,000 pseudo-replicates are shown as percentages at nodes. Bootstrap values are only shown for nodes with greater than 50% support. The clades of avian TLR1A/B, mammalian TLR1/6/10 are in red, orange, green, purple and blue, respectively. The branches of avian TLR1A and mammalian TLR10 are in a pink background. The branches of avian TLR1B and mammalian TLR1/TLR6 are in a lilac background.

**Figure 2:**
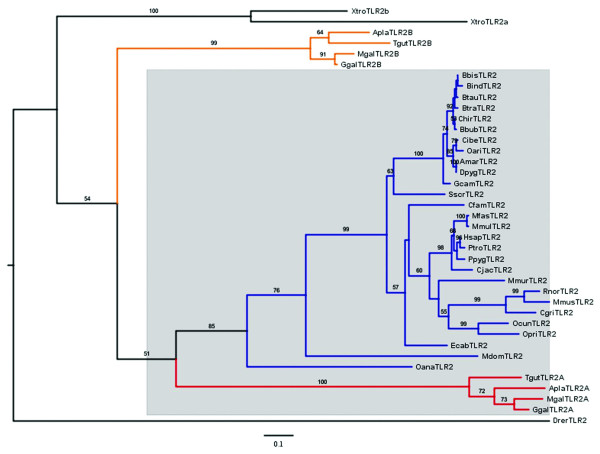
**ML tree of the TLR2 subfamily based on vertebrate central amino acid sequences**. The tree is rooted with *Danio rerio *and sequences are listed in Additional file 4. The bootstrap values of 1,000 pseudo-replicates are shown as percentages at nodes. Bootstrap values are only shown for nodes with greater than 50% support. The clades of avian TLR2A/B, mammalian TLR2 are in red, orange and blue, respectively. The branches of avian TLR2A and mammalian TLR2 are in a lilac background.

To examine the evolutionary origins of the TLR1 gene family we used the regions free of gene conversion defined above (Table 1) to identify orthologues between avian and mammalian genes. The ML tree based on the region free of gene conversion of the TLR1 subfamily is split into two distinct branches (Figure 1). In the first branch, the mammalian TLR1 and TLR6 genes showed closest similarity with the avian TLR1B genes (bootstrap value = 750/1000). For the second branch, the mammalian TLR10 gene clustered with the avian TLR1A genes (bootstrap value = 657/1000). These conclusions were supported further by finding high sequence identity between 3'-UTRs of human TLR1/chicken TLR1B (Additional file 12, Figure S10A) and 5'-UTRs of human TLR10/chicken TLR1A (Additional file 12, Figure S10B). A ML gene tree based on the central sequence free of gene conversion in the TLR2 subfamily showed the lineage for the single mammalian functional TLR2 gene to be more closely related to avian TLR2A than TLR2B (bootstrap value = 513/1000; Figure 2).

The dates for gene duplication were estimated from an analysis of the sequences free of gene conversion using CODEML [38] and summarized in Additional file 1, Table S4. Either global or local clock models of nucleotide substitution were used and calibrated using dates from the fossil record. For the TLR1 subfamily, we estimate an initial gene duplication event 359 Mya that gave rise to the TLR1A-TLR10 and TLR1B-TLR1/6 lineages, which was prior to the common ancestor for birds and mammals 310-325 Mya. Subsequently a further gene duplication 270-282 Mya gave rise to the TLR1 and TLR6 genes in mammals. The gene duplication event that gave rise to the TLR2 subfamily was estimated to be 341-356 Mya, which was prior to the common ancestor for birds and mammals 310-325 Mya. Similarly, the dates for gene conversion were inferred from an analysis of sequences predicted to have undergone gene conversion. Predicted dates were very variable between all species, varying from 0-47 Mya and generally older in mammals (Additional file 1, Table S4).

### Positive selection in the coding regions of the TLR1 gene family in birds and mammals

The estimation of substitution saturation from either the TLR1 or TLR2 subfamilies suggested the number of transitions reached a plateau when d > 0.7 (higher than the number of 160 Mya). Beyond this time, positions are saturated for pairs of nucleotide sequences (Additional file 13). Based on these comparisons we then used site-specific CODEML analysis to detect evidence of positive selection on the full length sequences of TLR1 gene family orthologues[38,46]: four groups of mammals (A: 12 TLR1, B: 12 TLR6, C: 15 TLR10 and D: 27 TLR2 species) and four birds (E: 4 TLR1A, F: 4 TLR1B, G: 4 TLR2A, H: 4 TLR2B species).

Maximum likelihood estimates of parameters under codon models of variable ω (the ratio between nonsynonymous and synonymous substitution rates) values across sites in the TLR dataset are listed in Table 2. For mammalian TLR1 parameter estimates under M2A suggest 2% of sites to be under positive selection with ŵ_2 _= 3.5. Estimates under M8 suggest 4% of sites to be under positive selection with ŵ_s _= 2.6. Four sites were inferred to have ω > 1 with high posterior probabilities (p > 0.9) under M8: residues 174T, 238A, 286Q and 599S. Similar estimates were found for TLR6. Estimates under M2A suggest 2% of sites to be under positive selection with ŵ_2 _= 2.8. Estimates under M8 suggest 4% of sites to be under positive selection with ŵ_s _= 2.0. Only, one site was inferred to have ω > 1 (p > 0.9) under M8: residue 90L and two under M2: 90L, 296D. TLR10 estimates under M2A did not suggest any sites to be under positive selection. Estimates under M8 suggest 2% of sites to be under positive selection with ŵ_s _= 2.5. No sites were inferred to have ω > 1 (p > 0.9) under either M2A or M8 models. Mammalian TLR2 estimates under M2A did not suggest any sites to be under positive selection. Estimates under M8 suggest 2% of sites to be under positive selection with ŵ_s _= 2.1. Two sites were inferred to have ω > 1 (p > 0.9) under M8: residues 453G and 766M.

**Table 2:** Positive selection in the TLR1 gene family in birds and mammals

**Group**^**1**^	No. of sequences	LL Test	-2(lnλ)	P-value	Proportion of sites ω> 1	**Positively selected sites**^**2**^
Mammalian TLR1	12	M1A vs. M2A	15.87	3.58E-04	ω= 3.46 (p = 0.02)	286Q, 599S*
		M7 vs. M8	26.84	1.49E-06	ω= 2.60 (p = 0.04)	174T, 238A, 286Q*, 599S**
		M8A vs. M8	20.17	3.54E-06	ω= 2.84 (p = 0.02)	
Mammalian TLR6	15	M1A vs. M2A	12.73	1.72E-03	ω = 2.84 (p = 0.02)	90L*, 296D
		M7 vs. M8	20.66	3.26E-05	ω = 2.02 (p = 0.04)	90L
		M8A vs. M8	17.79	1.24E-05		
Mammalian TLR10	12	M1A vs. M2A	0	1.00		ns
		M7 vs. M8	7.78	2.05E-02	ω = 2.47 (p = 0.02)	ns
		M8A vs. M8	6.06	6.89E-03		
Mammalian TLR2	27	M1A vs. M2A	0	1.00		ns
		M7 vs. M8	14.65	6.58E-04	ω = 2.07 (p = 0.02)	453G, 766M
		M8A vs. M8	8.21	2.09E-03		
Avian TLR1A	4	M1A vs. M2A	3.44	0.18		ns
		M7 vs. M8	3.63	0.16		ns
		M8A vs. M8	3.39	3.28E-02		
Avian TLR1B	4	M1A vs. M2A	0	1.00		ns
		M7 vs. M8	0.21	0.90		ns
		M8A vs. M8	0	0.48		
Avian TLR2A	4	M1A vs. M2A	4.82	8.98E-02	ω = 2.85 (p = 0.04)	ns
		M7 vs. M8	6.81	3.32E-02	ω = 2.19 (p = 0.09)	184T, 304N, 506S
		M8A vs. M8	4.75	1.47E-02		
Avian TLR2B	4	M1A vs. M2A	6.22	4.46E-02	ω = 2.00 (p = 0.11)	ns
		M7 vs. M8	9.81	7.43E-03	ω = 1.83 (p = 0.14)	175T, 260V, 496S
		M8A vs. M8	6.28	6.09E-03		

^1^The sequences for this analysis are provided in Additional file 4. ^2^The referenced sequences for mammalian and avian genes are human and duck, respectively. Amino acid sites inferred to be under positive selection from Bayes-Empirical-Bayes (BEB) analysis are shown with Log-likelihoods greater than 90% (blank), 95% (*) or 99% (**).

Avian TLR1A and TLR1B parameter estimates under M2A or M8 models did not suggest any sites to be under positive selection. The lack of evidence for positive selection in the avian TLR1A/TLR1B genes may have been due to lack of power with only four sequences to compare, however this was not the case for the four avian TLR2A/TLR2B sequences. TLR2A estimates under M2A suggest 4% of sites to be under positive selection with ŵ_2 _= 2.9. Estimates under M8 suggest 9% of sites to be under positive selection with ŵ_s _= 2.2. Three sites were inferred to have ω > 1 (p > 0.9) under M8: residues 184T, 304N and 506S. TLR2B estimates under M2A suggest 11% of sites to be under positive selection with ŵ_2 _= 2.0. Estimates under M8 suggest 14% of sites to be under positive selection with ŵ_s _= 1.8. Three sites were inferred to have ω > 1 (p > 0.9) under M8: residues 175T, 260V and 496S.

### Co-evolution of heterodimers between TLR1/6/10 and TLR2 in mammals

The CAPS analysis between 11 pairs of mammalian TLR1/2 sequences identified 28 groups of co-evolving amino acid residues (Additional file 14). Within TLR1, 35 residues were identified as co-evolving with 141 in TLR2 with correlation coefficients between 0.50 and 0.82 (P < 0.001). Most co-evolving residues were located in the extracellular region (33 in TLR1 and 130 in TLR2) and far fewer (2 in TLR1 and 8 in TLR2) in cytoplasmic regions. Similarly, we identified 63 groups of co-evolving amino acid residues using 12 pairs of TLR6/2 sequences (Additional file 15). Within TLR6, 76 amino acid residues were identified as co-evolving with 300 amino acid residues within TLR2 with correlation coefficients between 0.50 and 0.88 (P < 0.001). All of the inter-co-evolving sites of TLR1 and TLR6 were distributed in extracellular regions. Finally, we identified 76 groups of co-evolving amino acid residues using 12 pairs of TLR10/2 sequences (Additional file 16). Within TLR10, 91 amino acid residues were identified as co-evolving with 146 amino acid residues within TLR2 with correlation coefficients between 0.50 and 0.95 (P < 0.001). Most co-evolving residues within TLR10 were located in the extracellular region (56 in TLR10 and 102 in TLR2). However, inter-co-evolving sites were also detected in transmembrane (1 in TLR10 and 1 in TLR2) and cytoplasmic (2 in TLR10 and 4 in TLR2) regions.

We also looked for evidence of intra-molecular co-evolution using intra-protein CAPS analysis with the sequences of TLR1 family genes. Within TLR1, 111 amino acids from the extracellular region and 12 from the cytoplasmic region were identified as co-evolving with correlation coefficients between 0.72 and 1.00 (p < 0.001). However, there was no evidence of co-evolving amino acids from an analysis of 27 mammalian TLR2 sequences (p < 0.001). Within TLR6, 130 amino acids from the extracellular region and 14 from the cytoplasmic region which were identified as co-evolving with correlation coefficients between 0.61 and 0.99 (p < 0.001). Within TLR10, 29 amino acids from the extracellular region, 1 from the transmembrane domain and 6 from the cytoplasmic region were identified as co-evolving with correlation coefficients between 0.75 and 1.00 (p < 0.001).

## Discussion

TLRs play a crucial role in the host's early defense against invading pathogens [47,48]. In the past few years our understanding of the mechanisms by which TLRs recognize pathogens and induce distinct signaling pathways has increased significantly, particularly in mammals [49,50]. The tertiary structures of various TLRs and ligand complexes have also been determined [16,51]. In contrast, studies on avian TLRs have been very limited[2,15,19,20,52-54]. In this paper, we examined the evolution and selective constraints on members of the TLR1 gene family in both birds and mammals. Here we discuss the details of these evolutionary origins and the significance of selection constraints on the structure and function of this family of cell surface receptors.

### Evolutionary origins of the TLR1 gene family

Previous phylogenetic analyses of the TLR1 gene family suggested there had been many independent gene duplications in birds and mammals [1,2,23]. This contrasts with the TLR3, TLR4, TLR5 and TLR7 families where gene trees generally recapitulate species trees [1]. Detailed analysis presented in this paper, together with the previous studies by Kruithof et al. and Cormican et al, [21,22] suggests gene conversion events between paralogues had occurred during the evolution of the TLR1 gene family in both birds and mammals, giving rise to ambiguous orthologous relationships. We show that gene conversion events have occurred independently in most avian and mammalian lineages during the last 45 million years. Generally gene conversion events have been more recent in birds (0-20 Mya) than mammals (5-45 Mya). Gene conversion occurred in the mammalian TLR1/TLR6 genes 42-44 Mya in the common ancestor of four primate species (human, chimpanzee, orangutan and rhesus monkey) and independently in other mammals. Interestingly, gene conversion in the N- and C-terminal regions of avian TLR2A/TLR2B may have occurred independently. For example, in the duck we predict these events to be separated by 14 million years. These results suggest that N- and C-terminal regions of avian TLR2 genes may be under different selective constraints.

To understand more about the evolutionary origins of the TLR1 gene family, we constructed gene trees based on sequences from the TLR1 or TLR2 subfamilies free from gene conversion. For the TLR1 subfamily, two groups of orthologues were defined in birds and mammals as avian TLR1A/mammalian TLR10 and avian TLR1B/mammalian TLR1/TLR6, predicted to be the product of a gene duplication event 359 Mya. Subsequently in mammals, a further gene duplication event 270-282 Mya gave rise to the paralogues TLR1/6. This orthology between TLR1A/TLR10 and TLR1B/TLR1/TLR6 was strengthened by finding additional sequence identity in the flanking, non-coding regions of avian and mammalian genes (Additional file 12). Similarly, phylogenetic analysis strongly indicated that TLR2A to be orthologous to the functional TLR2 gene in mammals, sharing a common ancestor with TLR2B 341-356 Mya, prior to the split of reptiles and mammals 310-325 Mya. This analysis prompted us to search the genomes of mammals for TLR2 paralogues, possibly missed by current annotations. TLR2P was found in tandem with the functional TLR2 gene in human, chimpanzee, orangutan, rhesus monkey, marmoset, horse and dog. Together with the predicted time of divergence of the TLR2A/B genes (341-356 Mya), these results supposed that the TLR2B/2P genes were orthologues. In mammals, TLR2P may have recently been functional and independently pseudogenised in each species, possibly due to selection pressure against duplicate TLR2 genes. If true, this would suggest that different selective constraints are acting on the duplicate TLR2 genes in birds. This would be consistent with the more broad responses found in avian TLR1/2 heterodimers, possibly in response to a more diverse set of pathogens.

### Functional and structural significance of selection constraints within the TLR1 family

**Figure 3:**
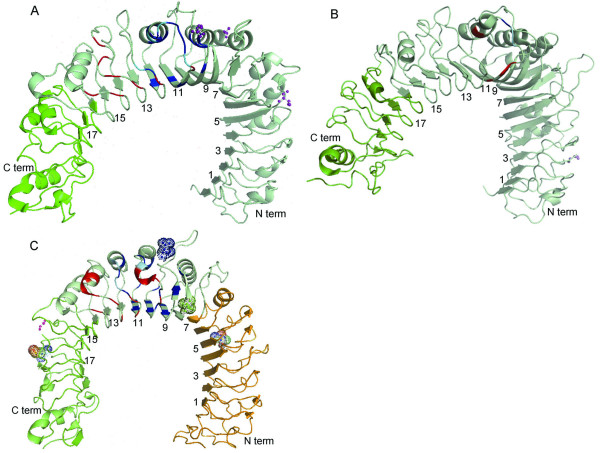
**Distribution of positively selected amino acid residues mapped onto the surface of TLR1 family protein structures**. The protein structures are from Jin et al. (2007) and Kang et al. (2009). The N, Central and C regions are defined in Table 1. The number represents the leucine-rich repeats. The regions in red, blue and cyan represent the TLR1/TLR6 and TLR2 interface, the ligand binding domain, and the region encompassing both interface and ligand binding, respectively. Pink ball and stick figures represent those amino acid sites under positive selection in mammals. Green and multi-coloured ball and stick figures represent amino acid sites under positive selection in avian TLR2-like proteins. Blue ball and stick figures represent sites within the TLR1 and TLR2 ligand binding domain under positive selection in avian TLR2A. A: Human TLR1. B: Mouse TLR6. C: Human TLR2.

Gene conversion is a process of non-reciprocal transfer of genetic information between genes. It is favored by high sequence homology, for example between tandem, duplicated genes and leads to their concerted evolution. Such a process has already been proposed to take place between the closely related TLR1/6 genes in mammals and the TLR2A/B genes in chicken and zebra finch [21,22]. Structural analysis based on the reported crystal structure of the TLR1-subfamily genes in human and mouse [17,51] suggested that gene conversion regions between the paralogues (avian TLR1A/B, mammalian TLR1/6) were mapped to a single region ranging from LRR14 to the TIR domain in birds and from LRR16 to the TIR domain in mammals (Figure 3A and 3B). For the TLR2 subfamily, gene conversion in avian TLR2A/B mapped to two regions; the first stretching from the N-terminus to LRR8 and the second from LRR15 to the TIR domain (Figure 3C). These results suggest that gene conversion may have played an important role in maintaining the conservation of the dimerization domain, transmembrane domain and the C-terminal intracellular TIR domain in TLR1-gene family. This may have been a selective advantage by maintaining co-evolution with binding partners, such as MyD88, TRIF, TIRAF and TRAM required for intracellular signaling [49,50]. In birds, an additional region of gene conversion was mapped in an N-terminal domain of the TLR2 subfamily. The function of this region is unknown and may bind to an uncharacterized binding partner.

Many genes of the immune system and those involved in host-pathogen interactions, such as the MHC complex, TRIM5alpha and HAVCR1, and show significant evidence of positive selection [10,55,56]. However, work so far suggests that most amino acid sites in TLRs are subject to purifying selection [1]. Domain-switching experiments between TLR1/6 showed the LRR9-12 modules to play crucial roles in lipopeptide recognition [57]. These modules constitute a critical portion of the central domain and form the ligand binding domain in TLR1/6/10. They also represent the most divergent regions and are likely to be subject to positive selection in response to pathogen infection. In mammals, our analysis detected positively selected (PS) sites in exposed regions in both TLR1 (238A, 286Q Figure 3A) and TLR6 (296D Figure 3B) at similar regions (suggesting both regions are functionally important, and possibly share a common function) in or near the central domain but not in TLR2. There were other residues inferred to be subject to positive selection, in the extracellular domain of TLR1 (174T in LRR6 Figure 3A), TLR6 (90L in LRR2 Figure 3B) and TLR2 (453G), and in the transmembrane domain of TLR1 (599S p > 0.99) and the TIR domain of TLR2 (766M). Changes in these sites may modify the ligand-binding activity of the TLR1/2 and TLR6/2 complexes. These results suggest that in mammals, changes in the ligand-binding activity of TLR1 and TLR6 may be modified through positive selection. In contrast to mammals, PS sites were detected in both avian TLR2A (304N in LRR10) and TLR2B (260V in LRR9), which map onto an exposed region (Figure 3C) in or near the ligand-binding domain and shows species-specific response to lipopeptides. The other sites are located at homologous positions in TLR2A (184T, 506S) and TLR2B (175T, 496S) in the extracellular domain at LRR6 and LRR19, respectively (Figure 3C), also suggesting those regions shared important functions. These results suggest that in birds, changes in the ligand-binding activity of TLR2A/B may be modified through positive selection, which contrasts with mammals where this appears not to be case. Also, unlike mammals, we did not find any sites showing high posterior probabilities in either the TLR1A/B molecules. These results could be due to lack of power in the small samples or they may indicate a difference in the selective constraints between TLR1 family proteins in birds and mammals.

TLR2 initiates a potent immune response by recognizing diacylated and triacylated lipopeptides. Its ligand specificity is controlled by whether it heterodimerizes with TLR1/6/10 [16-18]. Therefore extensive protein-protein interactions between TLR1/6/10 and TLR2 are to be expected, which should be reflected in a network of co-evolving amino acids. Intra-molecular CAPS analysis detected extensive co-evolution between residues within TLR1, 6 and 10 consistent with their ability to form homodimers. TLR2 does not form homodimers, which was reflected in the lack of evidence for any co-evolution between amino acid residues even with the alignment of sequences from 27 species. Inter-molecular CAPS analysis found extensive co-evolution between amino acids in TLR2 complexes with TLR1/2/10. Interestingly, amino acid residues from different domains within TLR2 were observed as co-evolving with the same residues in TLR1, TLR6 or TLR10, suggesting that there is a high codependence of evolution between the functional domains of TLR2.

**Figure 4:**
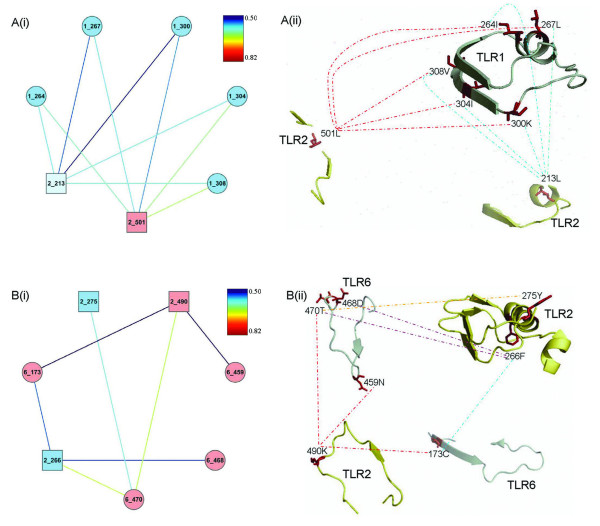
**Distribution of co-evolving amino acids residues mapped onto the surface of TLR2 family protein structures**. The protein structures are from Jin et al. (2007) and Kang et al. (2009). Co-evolving amino acids are shown as either network diagrams (displayed using Cytoscape) or mapped onto tertiary structures of protein domains (using PyMOL) for A: human TLR1/TLR2 and B: mouse TLR6/TLR2 complexes. In the network diagrams the TLR1/TLR6 and TLR2 amino acids are depicted by ellipse and rectangle shapes, respectively. Cyan, pale green and pink amino acid residues represent the ligand binding domain, α helices near the ligand binding domain and the extracellular domain, respectively. The color of lines in A(i) and B(i) represents the correlation coefficients of co-evolving amino acids. In the tertiary structures the peptide fragments from TLR1/TLR6 and TLR2 are in pale green and yellow, respectively. Amino acids joined by the same color of the broken lines are clustered to the one evolutionary group.

Most residues within TLR1 identified as co-evolving with TLR2 were located in the extracellular region (33 TLR1 and 130 TLR2) and far fewer (1 TLR1 and 2 TLR2) between cytoplasmic regions. Similar numbers of co-evolving residues were detected throughout the dimerization domains of TLR1 (7/76) and TLR2 (8/81). TLR2 however, has almost twice the number of co-evolving sites (17/90) in the lipid pocket compared to the lipid channel in TLR1 (10/82), consistent with the predicted interactions based on tertiary structures [16]. There are also five other small clusters of co-evolving amino acid residues, the largest involves residues in the lipid channel of TLR1 and the dimerization domain of TLR2 (Figure 4A) suggesting structural/functional co-dependence. In addition, the TIR domains of TLR1/TLR2 show co-evolution between residue 653 in TLR1 and residues 709 and 769 in TLR2. These residues are located between highly conserved regions thought to be important in TIR signaling [58].

**Figure 5:**
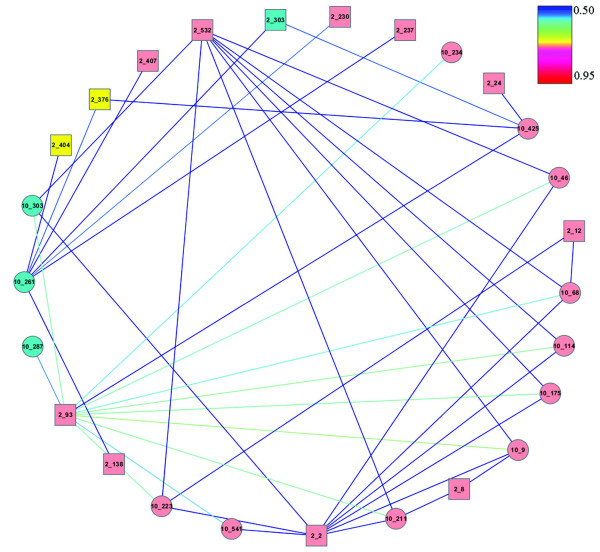
**Sub-network of co-evolving amino acids residues within the TLR10/2 complex**. Co-evolving amino acids are displayed using Cytoscape as described in the legend for Figure 4. The color of the lines represents the correlation coefficients of co-evolving amino acids.

TLR10 has been shown to have a similar ligand specificity and structure to TLR1, but to use a distinct signaling pathway in these and other TLRs [18]. Like TLR1, TLR10 has an extensive network of co-evolving sites with TLR2 in the extracellular region (56 TLR10 and 102 TLR2) but has some sites in both the transmembrane (1 TLR10 and 1 TLR2) and cytoplasmic (2 TLR10 and 4 TLR2) regions. Computational modeling and mutational analysis of the TLR10/TLR2 complex [18] suggested the presence of a TLR2 dimer interface and the lipopeptide-binding channel homologous to that found in TLR1. We also find there is a cluster of 26 co-evolving sites (Figure 5) involving four residues in ligand binding and two in dimerization domains. Interestingly, there were many more co-evolving sites within the TIR domain of the TLR10/TLR2 complex than found in TLR1/TLR2. Both complexes bind MyD88 but the signaling pathway for TLR10 seems to be distinct from other TLRs. The extra constraints detected in the TIR domain may represent binding sites for, as yet, uncharacterized proteins.

TLR6 recognizes diacylated lipopeptides by the formation of a heterodimer with TLR2. Previous analysis suggested that TLR6 did not have a lipid-binding channel as found in TLR1 [17]. This correlates with the lack of co-evolving sites in the homologous region in TLR6 but extensive co-evolution within the lipid binding pocket in TLR2 (29/90 residues). Protein-protein interactions are stronger in TLR6/TLR2 than TLR1/TLR2 where the dimerization interface in TLR6/TLR2 is 80% larger than TLR1/TLR2 [51]. This is consistent with there being more co-evolving sites in TLR2 (35/81) than TLR6 (6/79), which has a similar number to that found in TLR1 (7/76). There are also two other small clusters of co-evolving amino acid residues, the largest also involving residues in the lipid pocket of TLR2 and residues (459, 468, 470 and 490) elsewhere in the TLR6 protein (Figure 4B), which may represent other sites of functional and/or structural significance, but this awaits further investigation.

## Conclusions

The TLR1 gene family was previously thought to have arisen by a series of independent gene duplications in multiple vertebrates. However, we show here that the true history of gene duplication events has been masked by gene conversion between paralogous genes. Our detailed phylogenetic analysis not only clarifies the gene gains and losses within the TLR1 families of birds and mammals, but also defines orthologues between these vertebrates. TLR1 and 6 in mammals are the orthologues of avian TLR1B, and TLR10 in mammals is the orthologue of TLR1A. The functional TLR2 gene in mammals is the orthologue of the avian TLR2A genes. The mammalian orthologue of avian TLR2B has been lost in most mammals, with pseudogene fragments (TLR2P) present in a few species. In mammals, we predict amino acid sites under positive selection in TLR1, TLR2 and TLR6 but not TLR10 and detect co-evolution of amino acid residues between TLR1/TLR2 proteins that may be required to maintain their ability to form functional TLR1/2 complexes. In birds, we were able to predict positive selection in the TLR2A/B genes at functionally significant amino acid residues but not in TLR1A/B. In both birds and mammals these amino acid residues include known structural and functional sites, involved in ligand binding and dimerization. This analysis also predicts many other sites of functional and/or structural significance, but further investigations are required to define these roles. This analysis also suggests divergence in the function of avian and mammalian TLR1 gene family proteins, each under different selective constraints. In mammals, it appears that TLR1 and 6 are subject to positive selection more significant than TLR2, where in birds the reverse seems to be the case for TLR2-like genes. In contrast, the TLR10/TLR2 complex does not appear to be subject to positive selection. Our phylogenetic and structural analyses of vertebrate TLR1 family members has clarified their evolutionary history and predicts amino acid residues likely to be important in their primary function in the host's defense against invading pathogens.

## Abbreviations

Amar: *Antidocas marsupialis *(springbok); Apla: *Anas platyrhynchos *(duck); Bbis: *Bison bison *(american bison); Bbub: *Bubalus bubalis *(water buffalo); Bind: *Bos indicus *(gudali zebu); Btau: *Bos taurus *(cow); Btra: *Boselaphus tragocamelus *(nilgai); Cfam: *Canis familiaris *(dog); Cgri: *Cricetulus griseus *(chinese hamster); Chir: *Capra hircus *(goat); Cibe: *Capra ibex *(ibex); Cjac: *Callithrix Jacchus *(marmoset); Dnov: *Dasypus novemcinctus *(nine-banded armadillo); Dpyg: *Pongo pygmaeus *(phillipsi blesbok); Drer: *Danio rerio *(zebra fish); Ecab: *Equus caballus *(horse); Eeur: *Erinaceus europaeus *(hedgedog); Gcam: *Giraffa camelopardalis *(giraffe); Gful: *Gyps fulvus *(griffon vulture); Ggal: *Gallus gallus *(chicken); Hsap: *Homo sapiens *(human); Mdom: *Monodelphis domestica *(gray short-tailed opossum); Mfas: *Macaca fascicularis *(crab-eating macaque); Mgal: *Meleagris gallopavo *(turkey); Mmul: *Macaca mulatta *(rhesus monkey); Mmur: *Microcebus murinus *(mouse lemur); Mmus: *Mus musculus *(mouse); Oana: *Ornithorhynchus anatinus *(platypus); Oari: *Ovis aries *(sheep); Ocun: *Oryctolagus cuniculus *(rabbit); Opri: *Ochotona princeps *(pika); Ppyg: *Pongo pygmaeus *(orangutan); Ptro: *Pan troglodytes *(chimpanzee); Rnor: *Rattus norvegicus *(rat); Sscr: *Sus scrofa *(pig); Tgut: *Taeniopygia guttata *(zebra finch); Xtro: *Xenopus tropicalis *(western clawed frog).

## Authors' contributions

YH cloned the sequences of duck and turkey, prepared the sequences from the public databases, carried out the gene conversion analysis, constructed the phylogenetic topology, identified positively selected sites and drafted the manuscript. NT, LM and NL participated in the sequence of duck and turkey genes. JS helped to revise the manuscript. DB carried out the codon usage analysis, identified the co-evolutionary sites, participated in the identification of positively selected sites, helped to draft and revise the manuscript, and supervised the project. All authors read and approved the final manuscript.

## Additional files

Sequence clone and identities: Additional file 1, Table S1-2, Additional file 2, 5, 12 Gene conversion: Additional file 1, Table S3-4, Additional file 6, 7 Phylogenetic topology: Additional file 3, 8, 9, 10, 11 Positive selection: Additional file 13 Co-evolution: The groups, correlation coefficients and distribution on human or mouse tertiary structures of the inter-co-evolving sites in mammalian TLR1/2, TLR6/2 and TLR10/2 pairs are listed in Additional file 14, 15, 16. The co-evolutionary network identified by CAPS in mammalian TLR1/2, TLR6/2 and TLR10/2 pairs can be visualized with Cytoscape version 2.7.0 software using Additional file 14, 15, 16.

## Supplementary Material

Additional file 1 **Additional Tables**. Table S1. lists Genbank accession number, primer sequences and size of PCR products for the TLR1 family in duck and turkey. Table S2. lists the sequence identities between the orthologues and paralogues of the TLR1 gene family in birds and mammals. Table S3. lists the comparison of codon-usage bias in gene-conversion sequence versus non gene-conversion sequence of the TLR1 gene family in birds and mammals. Table S4. lists maximum likelihood estimates of divergence times (Mya) for gene conversion and duplication of the TLR1 gene family in birds and mammals under global- and local-clock models. Click here for file

Additional file 2 **Figure S1. Dot-plot comparisons of genomic sequences for the duck and chicken TLR1- and TLR2-like genes**. Additional file 2 shows the genomic sequence identities of the TLR1-gene family between the duck and chicken. Dot-plot comparisons of genomic sequences for the duck and chicken TLR1- and TLR2-like genes. A: the black and red arrows represent the coding sequences of TLR1A and TLR1B, respectively. Duck TLR1A and TLR1B coding sequences are located at positions 43,905-45,551 bp and 17,445-19,397 bp on the Y-axis, respectively; Chicken TLR1A and TLR1B coding sequences are located at positions 40,473-42,929 bp and 30,001-31,959 bp on the X-axis, respectively. B: the red and black arrows represent the coding sequences of TLR2A and TLR2B, respectively. Duck TLR2A and TLR2B coding sequences are located at positions 14,425-16,806 bp and 22,976-25,327 bp on the Y-axis, respectively; Chicken TLR2A and TLR2B coding sequences are located at positions 34,480-36,851 bp and 42,151-44,741 bp on the X-axis, respectively. Click here for file

Additional file 3 **Figure S2. ML trees of the vertebrate TLR1 and TLR2 subfamilies based on the multiple alignments of full length peptide sequences**. Additional file 3 shows the orthologous relationships of TLR1 and TLR2 subfamilies base on the multiple alignments of full length peptide sequences. ML trees of the vertebrate TLR1 and TLR2 subfamilies based on multiple alignments of full length peptide sequences. The sequences are listed in Table 1 and Additional file 4. These trees have been rooted with *Danio rerio*. The bootstrap values of 1,000 pseudo-replicates are shown as percentages at nodes. Bootstrap values are only shown for nodes with large than 50% support. A: TLR1 subfamily, avian TLR1A/B, and mammalian TLR1/6/10 clades are highlighted in orange, green, pink, lilac and cyan, respectively. Phylogenetic analysis derived from full length peptide sequences suggested that mammalian TLR1 subfamily diverged after the split of mammals and birds. In mammals, one duplication event may have occurred after the split of birds and mammals, but before the divergence of Montremes and Theria, which led to the TLR10 and TLR1/6 lineages. Subsequently, a further duplication event may have occurred during the divergence of Montremes/Theria to Laurasiatheria/Euarchontoglires giving rise to the TLR1 and TLR6 genes. In birds, a single duplication event prior to the split of Passeriforme and Galloanserae gave rise to the TLR1A and TLR1B genes. B: TLR2 subfamily, chicken TLR2A/B, turkey TLR2A/B, zebra finch TLR2A/B and mammalian TLR2 clades are highlighted in pink, cyan, green and lilac, respectively. The phylogenetic tree suggested that many recent independent duplications gave rise to the TLR2A and TLR2B genes in birds. Click here for file

Additional file 4 **The coding and pseudogene sequences of the vertebrate TLR1 gene family**. Additional file 4 provides gene name, species, common name, source, length and sequence of the TLR1 gene family from 34 species. Click here for file

Additional file 5 **Figure S3. Multiple sequence alignments of the TLR1 gene family with full length amino acid sequences**. Additional file 5 shows the alignments of the TLR1 gene family with full length amino acid sequences. Multiple sequence alignments of the TLR1 gene family with full length amino acid sequences. Alignments were made using Jalview 2.5.1 [32]. A: group 1, avian TLR1A/B. B: group 2, avian TLR2A/B. C: group 3, mammalian TLR1/6. Click here for file

Additional file 6 **Figure S4. Sequence similarity plots of coding and pseudogene sequences of the TLR1 gene family in birds and mammals**. Additional file 6 shows the sequence identity of coding and pseudogene sequences of the TLR1 gene family in birds and mammals. Sequence similarity plots of coding and pseudogene sequences of the TLR1 gene family in birds and mammals. Bootscan plots were calculated using SIMPLOT (Lole et al. 1999) with a sliding window size of 200 bp, step size of 20 bp, 1000 pseudo-replicates and neighbour-joining tree analysis. The avian and mammalian sequences are listed in Additional file 4, and the N, Central and C regions are defined in Table 1. The query sequences for panels A, B, C and D are GgalTLR1A, GgalTLR2A, BtauTLR1 and EcabTLR2P. The vertical axis is the % of permuted trees from 1000 bootstrap replicates. The horizontal axis indicates the nucleotide positions in base pairs. In panel D the suffixes "C" and "P" represent the homologous regions in TLR2 for functional genes and pseudogenes, respectively. A: TLR1A compared with TLR1B in birds. The N region of the query sequence (GgalTLR1A) is more similar to that of its orthologue (MgalTLR1A) than its paralogue (GgalTLR1B). However, the case in the C region is the reverse. B: TLR2A compared with TLR2B in birds. Both the N and C regions of the query sequence (GgalTLR2A) are more similar in the orthologue (MgalTLR2A) than its paralogue (GgalTLR2B), whereas, the reverse is true in the Central region. C: TLR1 compared with TLR6 in mammals. The N region of the query sequence (BtauTLR1) is more similar to its orthologue (EeurTLR1) than its paralogue (BtauTLR6). However, the case in the C region is the reverse. D: TLR2 pseudogenes compared with TLR2 functional genes in mammals. The query sequence (EcabTLR2P) is more conserved with the C region (which is defined according to the corresponding region of birds as shown in Table 1) of its paralogue (EcabTLR2C) than its orthologues (TLR2P of the other 5 species). In summary, sequence similarity of coding and pseudogene sequences of the TLR1 gene family indicate that orthologues are more like each other than the paralogue from the same species in the N region. However, the reverse is true in the C region, thus suggesting that gene conversion occurred in the C region. For TLR2, paralogues from the same species are more similar to each other than their orthologues in both the N and C regions, whereas, the case in the Central region is the reverse, implying that the N and C region have undergone gene conversion. Click here for file

Additional file 7 **Figure S5. Sequence similarity plots of the TLR1 subfamily using coding and non-coding 3`-UTR sequences in birds and mammals**. Additional file 7 shows the sequence identify of the TLR1 subfamily with coding and non-coding 3`-UTR sequences in birds and mammals. Sequence similarity plots of the TLR1 subfamily using coding and non-coding 3`-UTR sequences in birds and mammals. Plots were calculated using the SIMPLOT, as described in Figure S4. A: TLR1A compared with TLR1B in birds with GgalTLR1A (coding sequence: 1-2462 bp) as query. The N- and 3`-UTR regions of the query sequence are more similar to the corresponding regions of its orthologue (MgalTLR1A) than its paralogue (GgalTLR1B). However, the reverse is true in the C region. B and C: TLR1 compared with TLR6 in mammals, with SscrTLR1 (coding sequence: 10-2407 bp) and BtauTLR1 (coding sequence: 10-2394 bp) as queries, respectively. The N- and 3`-UTR regions of the query sequence in panel B (SscrTLR1) are more like the corresponding regions of its orthologue (BtauTLR1) than its paralogue (SscrTLR6), and the reverse is true in the C region. For panel C, the N region of the query sequence (BtauTLR1) is more similar to its orthologue (EeurTLR1) than its paralogue (BtauTLR6). However unike any other comparison both the C- and 3`-UTR regions of the query sequence (BtauTLR1) are more like to the corresponding regions of its paralogue (BtauTLR6) than any of its orthologues (TLR1 of the other 12 species). In summary, sequence similarity of coding and 3`-UTR sequences of the TLR1 subfamily indicates that orthologues are more similar to each other than the paralogue from the same species in both of the N- and 3`-UTR regions. However, the reverse is true in the C region. These results suggest gene conversion usually occurred in the coding, C-region of members of the TLR1 subfamily. The exception was BtauTLR1, where gene conversion appears to have extended from the coding C-region into the non-coding 3'-UTR for ~200 bp. Click here for file

Additional file 8 **Figure S6. ML trees of avian TLR1A/B based on amino acid sequences from either N or C terminal regions**. Additional file 9 shows the orthologous relationships of TLR1 subfamily in birds based on amino acid sequences from either N or C terminal regions. ML trees of avian TLR1A/B based on amino acid sequences from either N or C terminal regions. These trees have been rooted with *Danio rerio. Xenopus tropicalis *and *Homo sapiens *are included as outgroups and the sequences are listed in Table 1. The bootstrap values of 1,000 pseudo-replicates are shown as percentages at nodes. Bootstrap values are only shown for nodes with large than 50% support. A: ML tree based on N-terminal amino acid sequences. The avian TLR1A/B branches are shown in red and orange respectively. The clade containing avian TLR1A and human TLR10 is highlighted in pink, while the clade containing avian TLR1B and human TLR1/TLR6 is in lilac. B: ML tree based on C-terminal amino acid sequences. The TLR1A/B nodes in duck, chicken, turkey and zebra finch are in red, blue, green and orange, respectively. Click here for file

Additional file 9 **Figure S7. ML trees of avian TLR2A/B based on amino acid sequences from either N, central or C terminal regions**. Additional file 9 shows the orthologous relationships of TLR2 subfamily in birds based on amino acid sequences from either N, central or C terminal regions. ML trees of avian TLR2A/B based on amino acid sequences from either N, central or C terminal regions. These trees have been rooted with *Danio rerio*. *Xenopus tropicalis *and *Homo sapiens *are included as outgroups, and the sequences are listed in Table 1. The bootstrap values of 1,000 pseudo-replicates are shown as percentages at nodes. Bootstrap values are only shown for nodes with large than 50% support. A: ML tree based on N-terminal amino acid sequences. The TLR2A/B nodes in duck, chicken, turkey and zebra finch are shown in red, blue, green and orange, respectively. B: ML tree based on amino acid sequences in the central region of TLR2. The avian TLR2A/B nodes are shown in red and orange, respectively. The avian TLR2A and human TLR2 clade is highlighted in pink, while the avian TLR2B clade is in lilac. C: ML tree based on C-terminal amino acid sequences. The TLR2A/B nodes in duck, chicken, turkey and zebra finch are highlighted in red, blue, green and orange, respectively. Click here for file

Additional file 10 **Figure S8. ML trees of TLR1/6 in mammals based on amino acid sequences either N or C terminal regions**. Additional file 10 shows the orthologous relationships based on amino acid sequences from either N or C terminal regions. ML trees of TLR1/6 in mammals based on amino acid sequences from either N or C terminal regions. These trees have been rooted with *Danio rerio. Xenopus tropicalis *and *Gallus gallus *are included as outgroups and the sequences are listed in Table 1. The bootstrap values of 1,000 pseudo-replicates are shown as percentages at nodes. Bootstrap values are only shown for nodes with large than 50% support. A: ML tree based on N-terminal amino acid sequences. The mammalian TLR1/6 nodes are shown in green and purple, respectively. B: ML tree based on C-terminal amino acid sequences. The TLR1 and TLR6 nodes in human, chimpanzee, orangutan and rhesus monkey are shown in blue and red, respectively while the TLR1/6 nodes in cattle, dog, hedgehog, horse, marmoset, mouse, pig, and rat are shown in green. Click here for file

Additional file 11 **Figure S9. ML tree of functional and pseudogenised TLR2 genes in mammals based on gap-free, multiple DNA sequence alignments**. Additional file 11 shows the orthologous relationships of TLR2 subfamily in mammals based on gap-free, multiple DNA sequence alignments. ML tree of functional and pseudogenised TLR2 genes in mammals based on gap-free, multiple DNA sequence alignments. The tree has been rooted with *Ornithorhynchus anatinus*. The bootstrap values of 1,000 pseudo-replicates are shown as percentages at nodes. Bootstrap values are only shown for nodes with large than 50% support. The suffixes "C" and "P" represent the homologous regions in TLR2 for functional genes and TLR2 pseudogenes, respectively. The nodes of functional and TLR2P in human, chimpanzee, orangutan and rhesus monkey are shown in red and blue, respectively while the node of functional and TLR2P in horse is shown in green. Click here for file

Additional file 12 **Figure S10. Dot-plot comparisons of TLR1 subfamily mRNA sequences in human and chicken showing homology of coding and non-coding sequences**. Additional file 12 shows the sequence identities of TLR1 subfamily mRNA sequences in coding and non-coding regions of the human and chicken. Dot-plot comparisons of TLR1 subfamily mRNA sequences in the human and chicken showing homology of coding and non-coding sequences. The X-axis represents the sequence from human and the Y-axis the sequence from chicken. The regions between the black and the red arrows are the coding sequences from human and chicken, respectively. A: human TLR1 (coding sequence: 231-2591 bp) compared with chicken TLR1B (coding sequence: 334-2292 bp). B: human TLR10 (coding sequence: 485-2920 bp) compared with chicken TLR1A (coding sequence: 166-2622 bp). Click here for file

Additional file 13 **Figure S11. Plots of transitions/transversions versus genetic distance for TLR1 and TLR2 subfamilies**. Additional file 13 shows plots of transitions/transversions versus genetic distance of TLR1 and TLR2 subfamilies. Plots of transitions/transversions versus genetic distance for TLR1 and TLR2 subfamilies. The estimated number of transitions (s) and transversions (v) for each pairwise comparison is plotted against the genetic distance (d) calculated with the F84 model of nucleotide substitution using DAMBE (Xia and Xie 2001). Click here for file

Additional file 14 **The groups, correlation coefficients and distribution on human TLR1/2 tertiary structure of the inter-co-evolving sites in mammalian TLR1/2 pairs**. Additional file 14 lists the groups, correlation coefficients and distribution on human TLR1/2 of inter-co-evolving sites in mammalian TLR1/2 pairs. Click here for file

Additional file 15 **The groups, correlation coefficients and distribution on mouse TLR6/2 tertiary structures of the inter-co-evolving sites in mammalian TLR6/2 pairs**. Additional file 15 lists the groups, correlation coefficients and distribution on mouse TLR6/2 tertiary structures of the inter-co-evolving sites in mammalian TLR6/2 pairs. Click here for file

Additional file 16 **The groups, correlation coefficients and distribution on human TLR10/2 tertiary structures of the inter-co-evolving sites in mammalian TLR10/2 pairs**. Additional file 16 lists the groups, correlation coefficients and distribution on human TLR10/2 tertiary structures of the inter-co-evolving sites in mammalian TLR10/2 pairs. Click here for file
