# Supplementary material for: Molecular evolution of the vertebrate TLR1 gene family - a complex history of gene duplication, gene conversion, positive selection and co-evolution
Source: BMC Evol Biol. 2011 May 28;11:149. doi: 10.1186/1471-2148-11-149 (PMC3125219; doi:10.1186/1471-2148-11-149)
Supplement: Additional file 1 — Additional Tables. Table S1. lists Genbank accession number, primer sequences and size of PCR products for the TLR1 family in duck and turkey. Table S2. lists the sequence identities between the orthologues and paralogues of the TLR1 gene family in birds and mammals. Table S3. lists the comparison of codon-usage bias in gene-conversion sequence versus non gene-conversion sequence of the TLR1 gene family in birds and mammals. Table S4. lists maximum likelihood estimates of divergence times (Mya) for gene conversion and duplication of the TLR1 gene family in birds and mammals under global- and local-clock models. [file 1471-2148-11-149-S1.DOC]

**Table S1.** Genbank accession number, primer sequences and size of PCR products for the TLR1 family in duck and turkey

| **Gene/BAC** | **GenBank** | **Primers** | **Sequence (5` to 3`)** | **Size (bp)** |
| --- | --- | --- | --- | --- |
| Mallard Duck |  |  |  |  |
| DBS1203G04 | FJ477859 | dTLR1F | ATCCCTGGCAAAAGCATTGTG | 126 |
|  |  | dTLR1R | GATGGGCAAAGTACAGCTCG |  |
| DBS1405N01 | FJ477862 | dTLR2F | CTGTGCCTCCATAAGCGGG | 181 |
|  |  | dTLR2R | GCCACATCGTTGTTCTCGTC |  |
| Turkey |  |  |  |  |
| TLR1A | FJ477857 | tTLR1AF1 | AGGTTGGACTTCTTATTGAGGCATAC | 903 |
|  |  | tTLR1AR1 | AGATGAATCCCAAACTAGCAGAAAAA |  |
|  |  | tTLR1AF2 | ATTTGCCCTCCCCTTCTTTA | 541 |
|  |  | tTLR1AR2 | TGATTTCATACGGCTGGTCA |  |
|  |  | tTLR1AF3 | AAAACCCGTTCAAGTGTTCG | 667 |
|  |  | tTLR1AR3 | GTGGTACCTCGCAGGGATAA |  |
| TLR1B | FJ477858 | tTLR1BF1 | TATTGAGCATCTCCAAGGAA | 803 |
|  |  | tTLR1BR1 | TCATACGGCTGGTCATGA |  |
|  |  | tTLR1BF2 | CATGGTATGTCCTTCATCTGACAG | 1214 |
|  |  | tTLR1BR2 | TGGTACCTCGCAGGGATAAC |  |
|  |  | tTLR1BF3 | TACGAGCTGTACTTTGCCCA | 299 |
|  |  | tTLR1BR3 | AACAAGGAGAAGAAGAAACTGCAT |  |
| TLR2A | FJ477860 | tTLR2AF1 | TTAAAAGGGTGTGCCAGGAG | 270 |
|  |  | tTLR2AR1 | GTCCAAACCCATGAAAGAGC |  |
|  |  | tTLR2AF2 | CCTGAAGAACAAGCCCTGAG | 1505 |
|  |  | tTLR2AR2 | GGACGCAAACTCTTTCTTGG |  |
|  |  | tTLR2AF3 | AACTGCCATTTCTCAAGGAG | 958 |
|  |  | tTLR2AR3 | TATACTGAAACGAGCTCCTAACCTGT |  |
|  |  | tTLR2A3GSP2 | TTCTCGCACTTTCGCCTCTT | 320 |
|  |  | tTLR2AR4 | TCCTTCCAGAGACGACTTCA(N) |  |
|  |  | tTLR2A3GSP1 | TTTGTGCCTGGGAAGTGGAT |  |
|  |  | tTLR2A3GSP2 | TTCTCGCACTTTCGCCTCTT |  |
| TLR2B | FJ477861 | tTLR2BF1 | TGGAAAGTGTGGATGATCTAC | 779 |
|  |  | tTLR2BR1 | AGCATCTGTAAATAAGCACTGT |  |
|  |  | tTLR2BF2 | GGAACTGCCACTTCTCAAGG | 988 |
|  |  | tTLR2BR2 | AATGGAAATAAATCCAGTAGAGGATG |  |

3GSP: gene specific primers for 3’-end sequence of Turkey TLR2A.

**Table S2.** The sequence identities between the orthologues and paralogues of the TLR1 gene family in birds and mammals

| Species^1^ | Group | Average±SD^2^(%) | | | | Average ± SD^3^ (%) | | | |
| --- | --- | --- | --- | --- | --- | --- | --- | --- | --- |
|  |  | Full | N | Central | C | Full | N | Central | C |
| Birds | TLR1A | 88.50±3.67 | 85.33±3.67 | — | 86.50±3.56 | 80.50±4.23 | 78.50±4.97 | — | 83.83±3.37 |
|  | TLR1A/TLR1B | 68.25±1.89 | 19.75±1.89 | — | 97.50±1.29 | 71.50±1.73 | 31.50±2.52 | — | 96.75±2.99 |
|  | TLR1B | 84.10±3.75 | 80.17±4.88 | — | 86.50±3.78 | 78.60±4.86 | 69.00±7.54 | — | 83.67±4.68 |
|  | TLR2A | 85.00±4.82 | 84.67±5.13 | 82.17±5.38 | 86.33±4.45 | 80.50±5.58 | 79.00±7.04 | 74.50±6.60 | 86.00±4.00 |
|  | TLR2A/TLR2B | 88.00±0.82 | 99.25±0.50 | 51.50±7.33 | 99.00±0.82 | 82.50±0.58 | 99.25±0.58 | 34.50±2.89 | 98.50±1.29 |
|  | TLR2B | 85.33±4.87 | 84.33±4.72 | 84.17±5.12 | 87.00±4.34 | 81.67±5.75 | 79.33±6.25 | 76.83±8.33 | 86.67±3.67 |
| Mammals | TLR1 | 83.67±6.46 | 81.94±7.50 | — | 86.17±5.36 | 78.50±7.65 | 74.88±9.28 | — | 82.42±6.15 |
|  | TLR1/TLR6 | 75.83±2.17 | 61.75±3.41 | — | 96.83±1.34 | 62.02±3.43 | 46.67±3.20 | — | 95.67±2.27 |
|  | TLR6 | 83.55±6.53 | 82.05±7.54 | — | 86.20±5.17 | 78.65±7.65 | 75.76±9.10 | — | 83.15±5.76 |

^1^The four avian species are chicken, turkey, duck and zebra finch and twelve mammalian species are cattle, dog, marmoset, horse, hedgehog, human, rhesus monkey, mouse, orangutan, chimpanzee, rat and pig. ^2,3^ represent the average and standard deviation of coding and protein sequence identities respectively. "N ", "Central" and "C " are defined based on the gene conversion results listed in Table 1.

**Table S3.** The comparison of codon-usage bias in gene-conversion sequence versus non gene-conversion sequence of the TLR1 gene family in birds and mammals

| **Group^1^** | **Non conversion region^2^** | | **Conversion region^2^** | |
| --- | --- | --- | --- | --- |
|  | **Average ± SD** | **PI** | **Average ± SD** | **PI** |
| Avian TLR1A/B | 46.25±4.65 | 65.81±7.76 | 387.75±16.52 | 99.47±0.76 |
| Avian TLR2A/B | 66.25±3.20 | 56.11±4.91 | 221.50±6.14 | 99.44±0.57 |
|  |  |  | 352.50±6.24 | 98.71±1.34 |
| Mammalian TLR1/6 | 202.83±11.42 | 57.34±3.39 | 302.25±19.82 | 96.44±1.51 |

^1^The four avian and twelve mammalian species are the same as in Table S2.

^2^"Non conversion region": N region of TLR1A/TLR1B and TLR1/TLR6, Central region of TLR2A/TLR2B as referenced in Table 1; "Conversion region": C region of TLR1A/TLR1B, TLR2A/TLR2B, TLR1/TLR6 and N region of TLR2A/TLR2B as referenced in Table 1; "Average ± SD" means the average and standard deviation of conserved amino acids between the paralogues from the same species in each group; "PI" represents the percentage identical codon usage for conserved amino acids between the paralogues from the same species in each group.

**Table S4.** Maximum likelihood estimates of divergence times (Mya) for gene conversion and duplication of the TLR1 gene family in birds and mammals under global- and local-clock models

| Class | Species | Genes | Global Clock (Mya) | Local Clock (Mya) |
| --- | --- | --- | --- | --- |
| Gene conversion | *Gallus gallus* | TLR1A/B | 7 | 7 |
|  | *Meleagris gallopavo* | TLR1A/B | 10 | 10 |
|  | *Anas platyrhynchos* | TLR1A/B | 18 | 20 |
|  | *Taeniopygia guttata* | TLR1A/B | 0 | 0 |
|  | *Gallus gallus* | TLR2A/B N-region | 2 | 1 |
|  | *Meleagris gallopavo* | TLR2A/B N-region | 0 | 0 |
|  | *Anas platyrhynchos* | TLR2A/B N-region | 1 | 1 |
|  | *Taeniopygia guttata* | TLR2A/B N-region | 4 | 4 |
|  | *Gallus gallus* | TLR2A/B C-region | 0 | 0 |
|  | *Meleagris gallopavo* | TLR2A/B C-region | 5 | 4 |
|  | *Anas platyrhynchos* | TLR2A/B C-region | 14 | 15 |
|  | *Taeniopygia guttata* | TLR2A/B C-region | 6 | 4 |
|  | *Primates* | TLR1/6 | 42 | 44 |
|  | *Callithric jacchus* | TLR1/6 | 15 | 18 |
|  | *Erinaceus europaeus* | TLR1/6 | 7 | 6 |
|  | *Equus caballus* | TLR1/6 | 36 | 45 |
|  | *Canis familiaris* | TLR1/6 | 9 | 8 |
|  | *Sus scrofa* | TLR1/6 | 10 | 10 |
|  | *Bos taurus* | TLR1/6 | 12 | 11 |
|  | *Rattus norvegicus* | TLR1/6 | 6 | 5 |
|  | *Mus musculus* | TLR1/6 | 6 | 5 |
|  | *Equus caballus* | TLR2/P | 4 | 6 |
| Gene duplication | *Avian* | TLR1A/B | 359 | 359 |
|  | *Avian* | TLR2A/B | 341 | 356 |
|  | *Mammalian* | TLR1/6 | 282 | 270 |
|  | *Homo sapiens* | TLR1/6/10 | 359 | 359 |

Primates: human, chimpanzee, orangutan and rhesus monkey; "TLR2/P" represents the C-region and pseudogene of TLR2; "Avian" and "Mammalian" represent the four avian and twelve mammalian species listed in Table 1.
