# Supplementary figures and images for: Molecular evolution of the vertebrate TLR1 gene family - a complex history of gene duplication, gene conversion, positive selection and co-evolution
Source: BMC Evol Biol. 2011 May 28;11:149. doi: 10.1186/1471-2148-11-149 (PMC3125219; doi:10.1186/1471-2148-11-149)

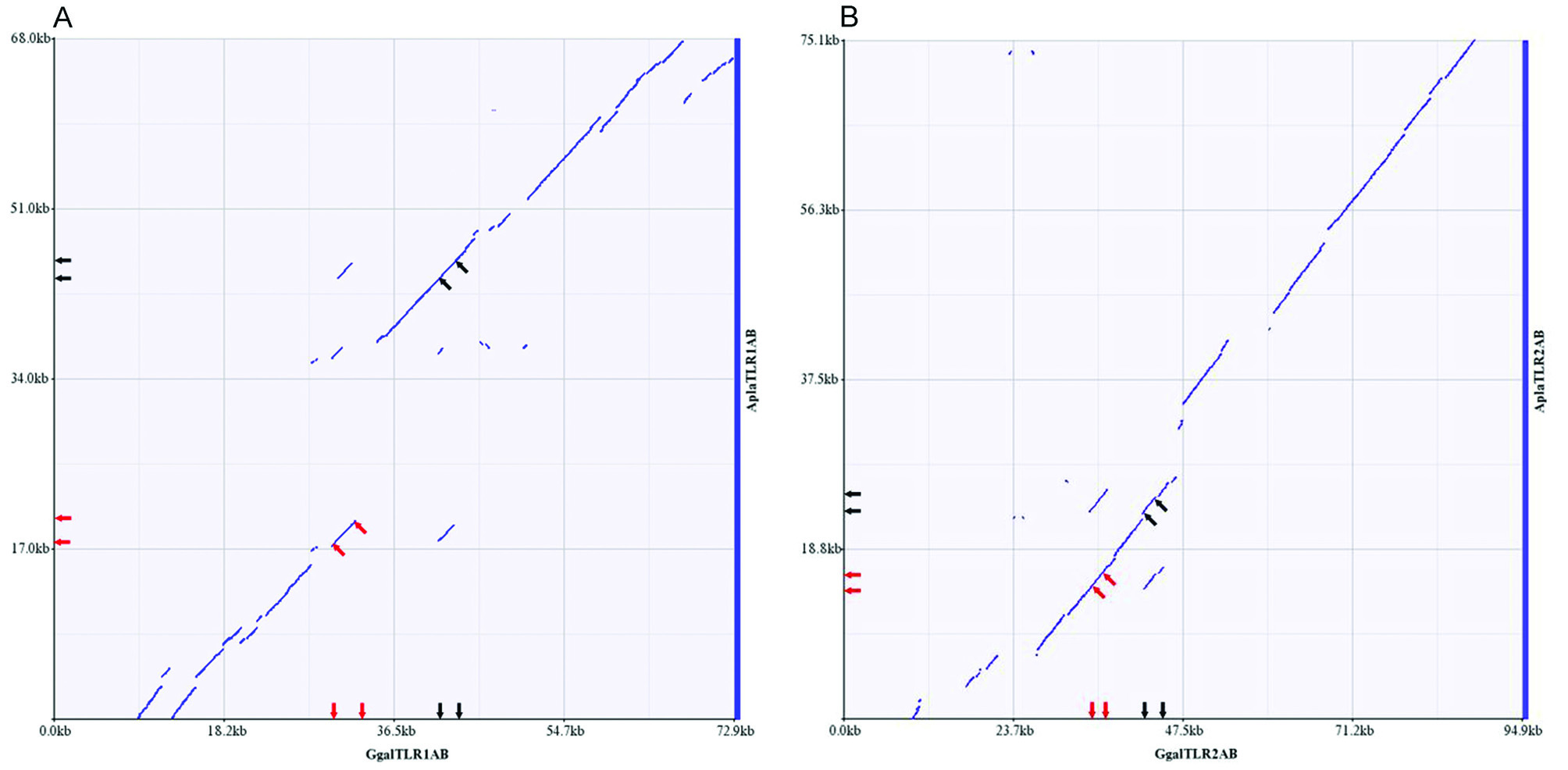

Supplement: Additional file 2 — Figure S1. Dot-plot comparisons of genomic sequences for the duck and chicken TLR1- and TLR2-like genes. Additional file 2 shows the genomic sequence identities of the TLR1-gene family between the duck and chicken. Dot-plot comparisons of genomic sequences for the duck and chicken TLR1- and TLR2-like genes. A: the black and red arrows represent the coding sequences of TLR1A and TLR1B, respectively. Duck TLR1A and TLR1B coding sequences are located at positions 43,905-45,551 bp and 17,445-19,397 bp on the Y-axis, respectively; Chicken TLR1A and TLR1B coding sequences are located at positions 40,473-42,929 bp and 30,001-31,959 bp on the X-axis, respectively. B: the red and black arrows represent the coding sequences of TLR2A and TLR2B, respectively. Duck TLR2A and TLR2B coding sequences are located at positions 14,425-16,806 bp and 22,976-25,327 bp on the Y-axis, respectively; Chicken TLR2A and TLR2B coding sequences are located at positions 34,480-36,851 bp and 42,151-44,741 bp on the X-axis, respectively. [file 1471-2148-11-149-S2.JPEG]

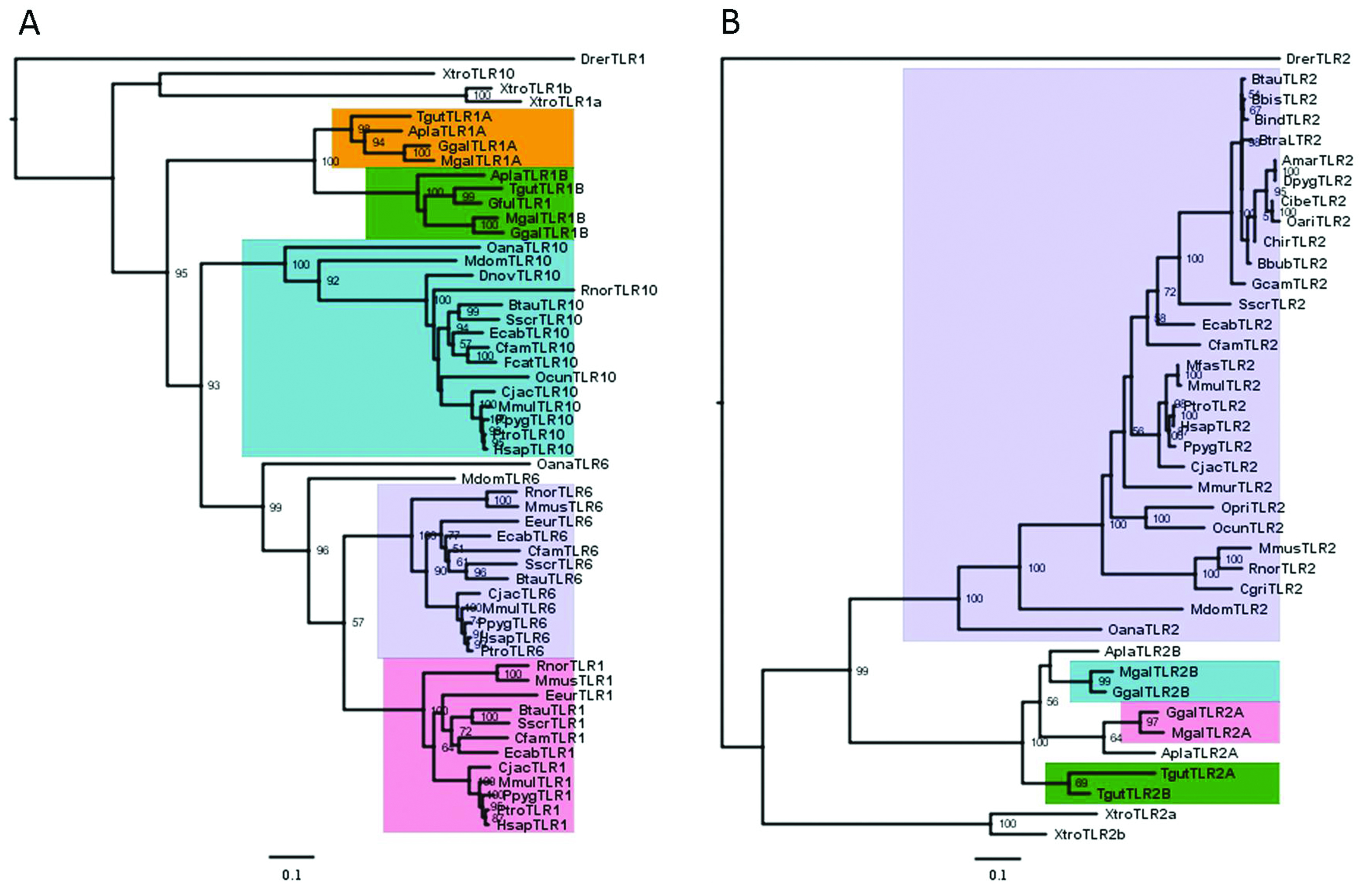

Supplement: Additional file 3 — Figure S2. ML trees of the vertebrate TLR1 and TLR2 subfamilies based on the multiple alignments of full length peptide sequences. Additional file 3 shows the orthologous relationships of TLR1 and TLR2 subfamilies base on the multiple alignments of full length peptide sequences. ML trees of the vertebrate TLR1 and TLR2 subfamilies based on multiple alignments of full length peptide sequences. The sequences are listed in Table 1 and Additional file 4. These trees have been rooted with Danio rerio. The bootstrap values of 1,000 pseudo-replicates are shown as percentages at nodes. Bootstrap values are only shown for nodes with large than 50% support. A: TLR1 subfamily, avian TLR1A/B, and mammalian TLR1/6/10 clades are highlighted in orange, green, pink, lilac and cyan, respectively. Phylogenetic analysis derived from full length peptide sequences suggested that mammalian TLR1 subfamily diverged after the split of mammals and birds. In mammals, one duplication event may have occurred after the split of birds and mammals, but before the divergence of Montremes and Theria, which led to the TLR10 and TLR1/6 lineages. Subsequently, a further duplication event may have occurred during the divergence of Montremes/Theria to Laurasiatheria/Euarchontoglires giving rise to the TLR1 and TLR6 genes. In birds, a single duplication event prior to the split of Passeriforme and Galloanserae gave rise to the TLR1A and TLR1B genes. B: TLR2 subfamily, chicken TLR2A/B, turkey TLR2A/B, zebra finch TLR2A/B and mammalian TLR2 clades are highlighted in pink, cyan, green and lilac, respectively. The phylogenetic tree suggested that many recent independent duplications gave rise to the TLR2A and TLR2B genes in birds. [file 1471-2148-11-149-S3.JPEG]

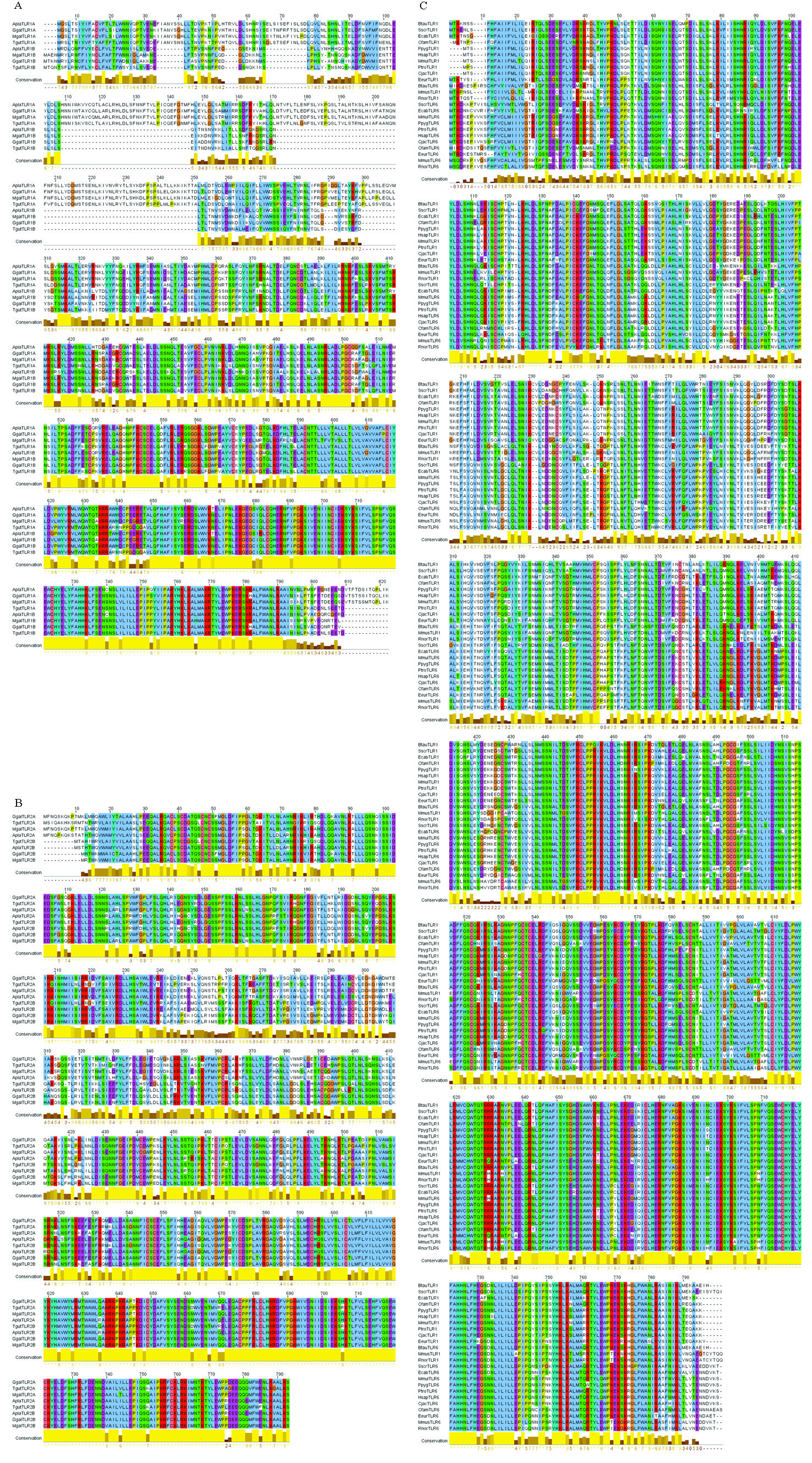

Supplement: Additional file 5 — Figure S3. Multiple sequence alignments of the TLR1 gene family with full length amino acid sequences. Additional file 5 shows the alignments of the TLR1 gene family with full length amino acid sequences. Multiple sequence alignments of the TLR1 gene family with full length amino acid sequences. Alignments were made using Jalview 2.5.1 [32]. A: group 1, avian TLR1A/B. B: group 2, avian TLR2A/B. C: group 3, mammalian TLR1/6. [file 1471-2148-11-149-S5.JPEG]

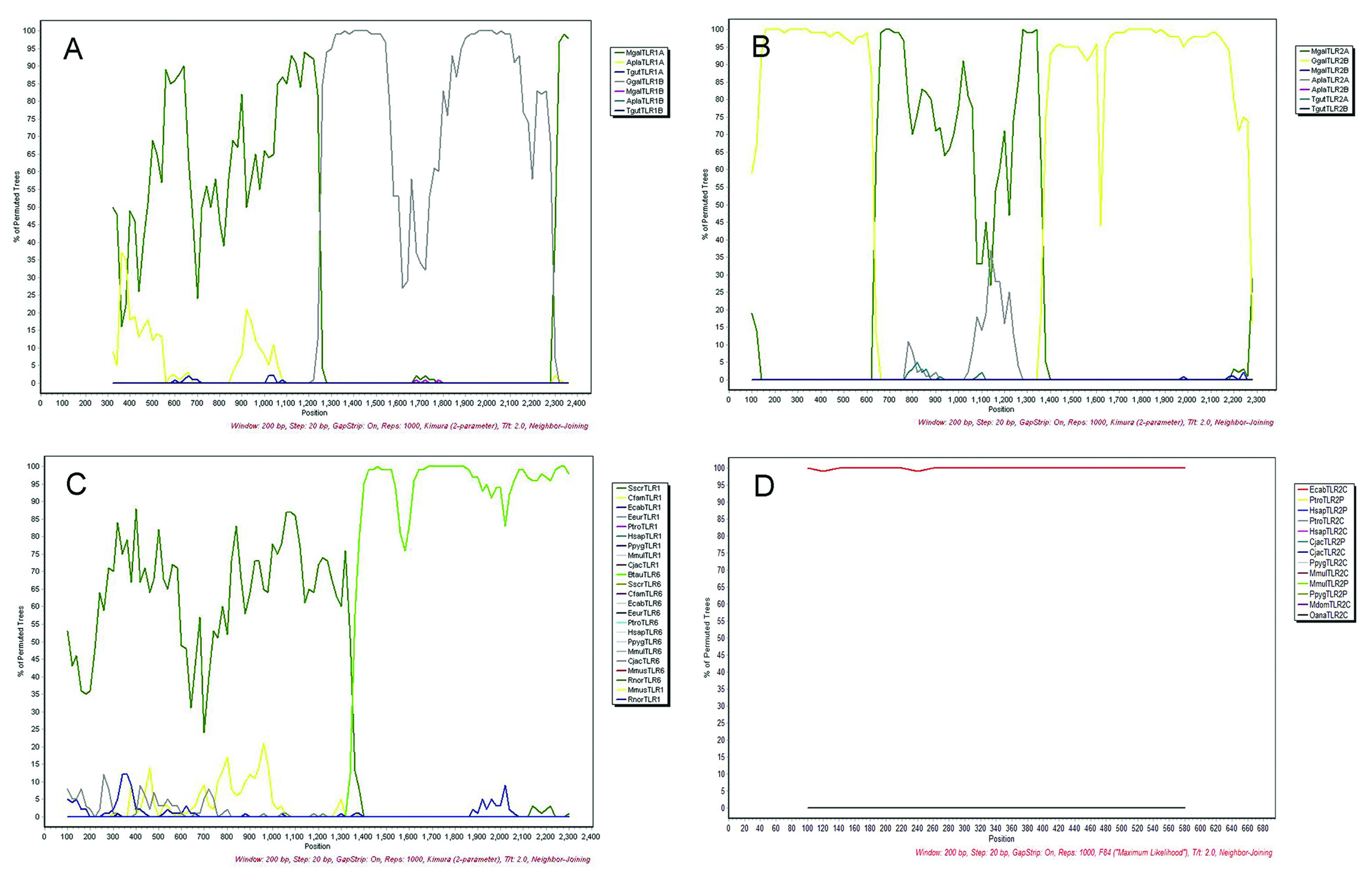

Supplement: Additional file 6 — Figure S4. Sequence similarity plots of coding and pseudogene sequences of the TLR1 gene family in birds and mammals. Additional file 6 shows the sequence identity of coding and pseudogene sequences of the TLR1 gene family in birds and mammals. Sequence similarity plots of coding and pseudogene sequences of the TLR1 gene family in birds and mammals. Bootscan plots were calculated using SIMPLOT (Lole et al. 1999) with a sliding window size of 200 bp, step size of 20 bp, 1000 pseudo-replicates and neighbour-joining tree analysis. The avian and mammalian sequences are listed in Additional file 4, and the N, Central and C regions are defined in Table 1. The query sequences for panels A, B, C and D are GgalTLR1A, GgalTLR2A, BtauTLR1 and EcabTLR2P. The vertical axis is the % of permuted trees from 1000 bootstrap replicates. The horizontal axis indicates the nucleotide positions in base pairs. In panel D the suffixes "C" and "P" represent the homologous regions in TLR2 for functional genes and pseudogenes, respectively. A: TLR1A compared with TLR1B in birds. The N region of the query sequence (GgalTLR1A) is more similar to that of its orthologue (MgalTLR1A) than its paralogue (GgalTLR1B). However, the case in the C region is the reverse. B: TLR2A compared with TLR2B in birds. Both the N and C regions of the query sequence (GgalTLR2A) are more similar in the orthologue (MgalTLR2A) than its paralogue (GgalTLR2B), whereas, the reverse is true in the Central region. C: TLR1 compared with TLR6 in mammals. The N region of the query sequence (BtauTLR1) is more similar to its orthologue (EeurTLR1) than its paralogue (BtauTLR6). However, the case in the C region is the reverse. D: TLR2 pseudogenes compared with TLR2 functional genes in mammals. The query sequence (EcabTLR2P) is more conserved with the C region (which is defined according to the corresponding region of birds as shown in Table 1) of its paralogue (EcabTLR2C) than its orthologues (TLR2P of the other 5 species). In sum [file 1471-2148-11-149-S6.JPEG]

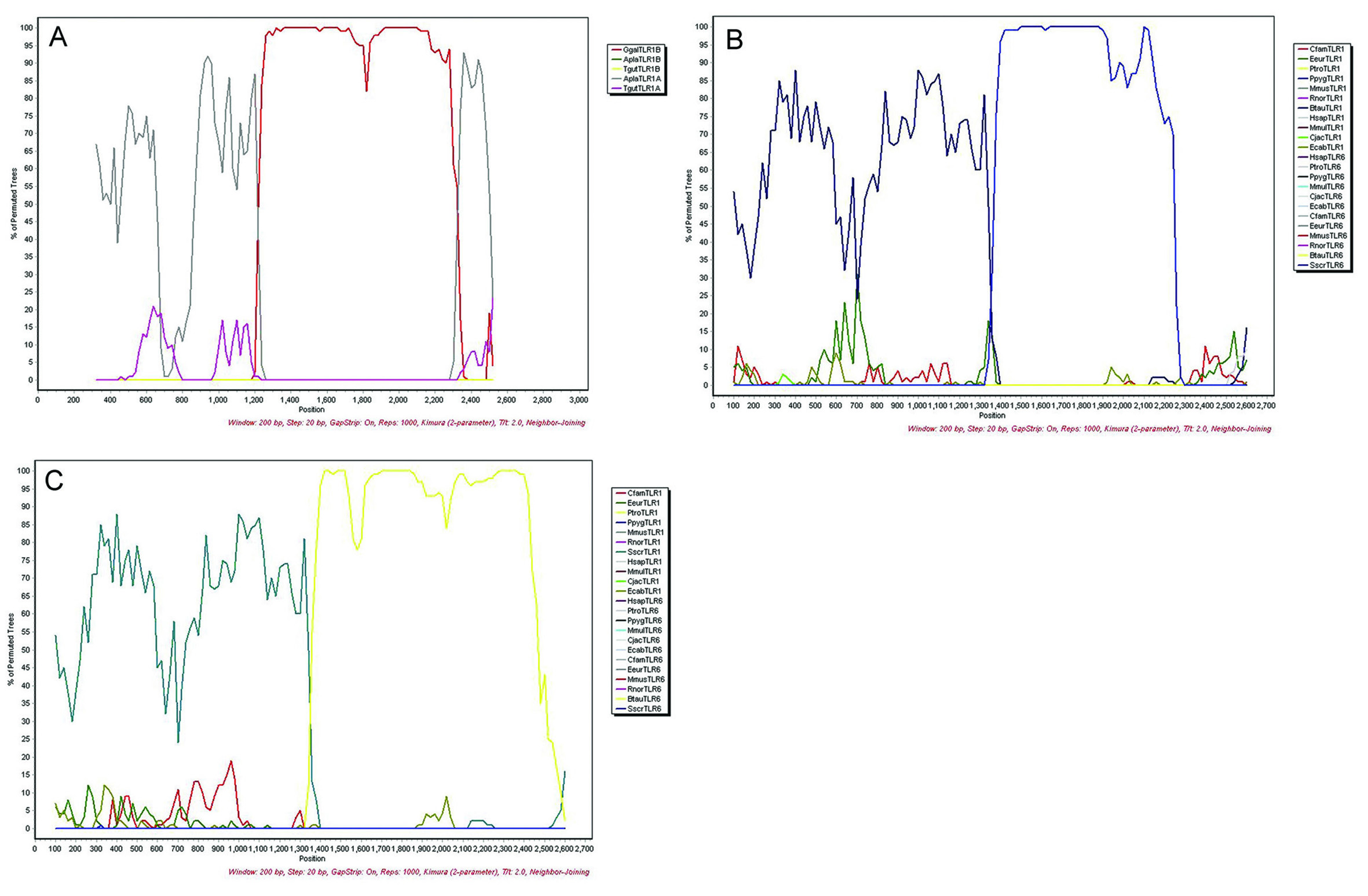

Supplement: Additional file 7 — Figure S5. Sequence similarity plots of the TLR1 subfamily using coding and non-coding 3`-UTR sequences in birds and mammals. Additional file 7 shows the sequence identify of the TLR1 subfamily with coding and non-coding 3`-UTR sequences in birds and mammals. Sequence similarity plots of the TLR1 subfamily using coding and non-coding 3`-UTR sequences in birds and mammals. Plots were calculated using the SIMPLOT, as described in Figure S4. A: TLR1A compared with TLR1B in birds with GgalTLR1A (coding sequence: 1-2462 bp) as query. The N- and 3`-UTR regions of the query sequence are more similar to the corresponding regions of its orthologue (MgalTLR1A) than its paralogue (GgalTLR1B). However, the reverse is true in the C region. B and C: TLR1 compared with TLR6 in mammals, with SscrTLR1 (coding sequence: 10-2407 bp) and BtauTLR1 (coding sequence: 10-2394 bp) as queries, respectively. The N- and 3`-UTR regions of the query sequence in panel B (SscrTLR1) are more like the corresponding regions of its orthologue (BtauTLR1) than its paralogue (SscrTLR6), and the reverse is true in the C region. For panel C, the N region of the query sequence (BtauTLR1) is more similar to its orthologue (EeurTLR1) than its paralogue (BtauTLR6). However unike any other comparison both the C- and 3`-UTR regions of the query sequence (BtauTLR1) are more like to the corresponding regions of its paralogue (BtauTLR6) than any of its orthologues (TLR1 of the other 12 species). In summary, sequence similarity of coding and 3`-UTR sequences of the TLR1 subfamily indicates that orthologues are more similar to each other than the paralogue from the same species in both of the N- and 3`-UTR regions. However, the reverse is true in the C region. These results suggest gene conversion usually occurred in the coding, C-region of members of the TLR1 subfamily. The exception was BtauTLR1, where gene conversion appears to have extended from the coding C-region into the non-coding 3'-UTR for ~200 bp. [file 1471-2148-11-149-S7.JPEG]

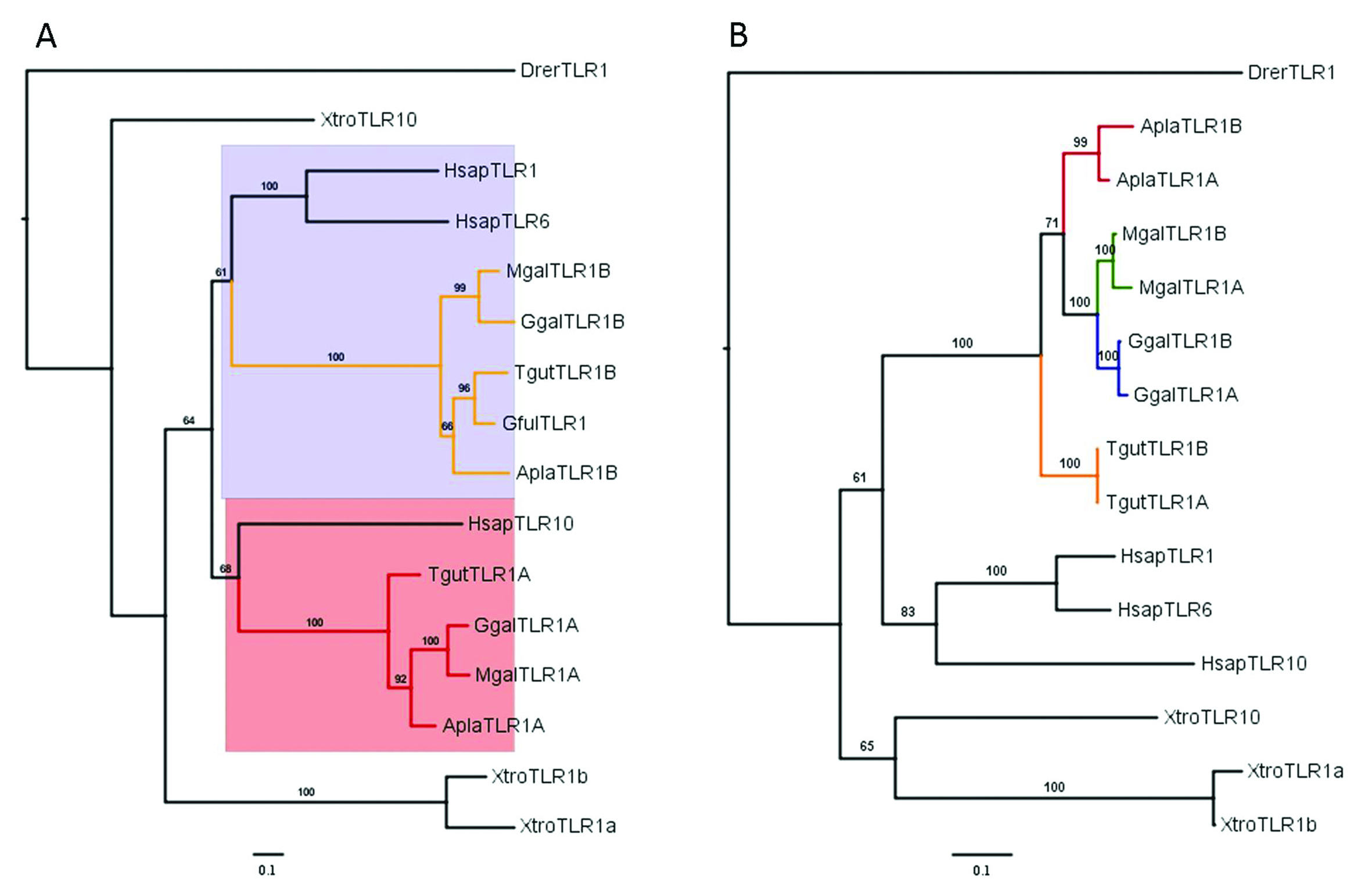

Supplement: Additional file 8 — Figure S6. ML trees of avian TLR1A/B based on amino acid sequences from either N or C terminal regions. Additional file 9 shows the orthologous relationships of TLR1 subfamily in birds based on amino acid sequences from either N or C terminal regions. ML trees of avian TLR1A/B based on amino acid sequences from either N or C terminal regions. These trees have been rooted with Danio rerio. Xenopus tropicalis and Homo sapiens are included as outgroups and the sequences are listed in Table 1. The bootstrap values of 1,000 pseudo-replicates are shown as percentages at nodes. Bootstrap values are only shown for nodes with large than 50% support. A: ML tree based on N-terminal amino acid sequences. The avian TLR1A/B branches are shown in red and orange respectively. The clade containing avian TLR1A and human TLR10 is highlighted in pink, while the clade containing avian TLR1B and human TLR1/TLR6 is in lilac. B: ML tree based on C-terminal amino acid sequences. The TLR1A/B nodes in duck, chicken, turkey and zebra finch are in red, blue, green and orange, respectively. [file 1471-2148-11-149-S8.JPEG]

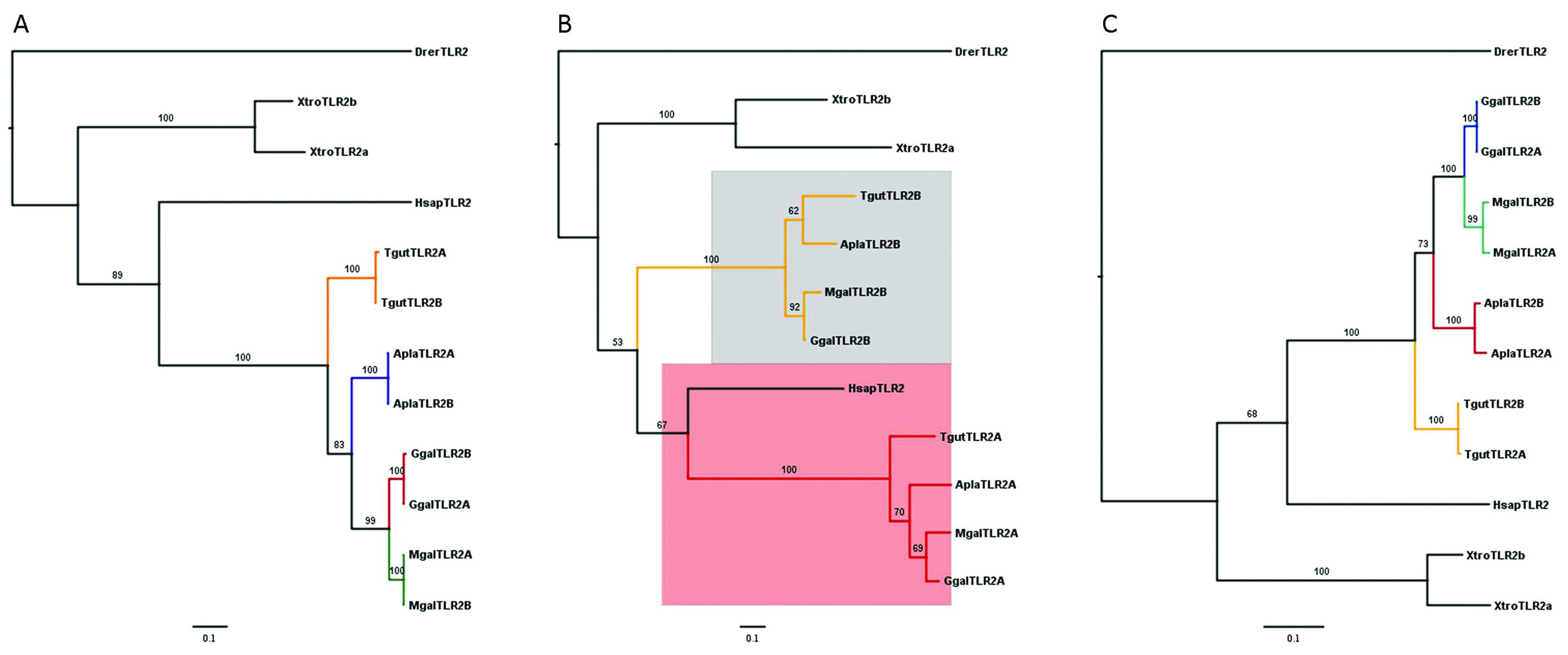

Supplement: Additional file 9 — Figure S7. ML trees of avian TLR2A/B based on amino acid sequences from either N, central or C terminal regions. Additional file 9 shows the orthologous relationships of TLR2 subfamily in birds based on amino acid sequences from either N, central or C terminal regions. ML trees of avian TLR2A/B based on amino acid sequences from either N, central or C terminal regions. These trees have been rooted with Danio rerio. Xenopus tropicalis and Homo sapiens are included as outgroups, and the sequences are listed in Table 1. The bootstrap values of 1,000 pseudo-replicates are shown as percentages at nodes. Bootstrap values are only shown for nodes with large than 50% support. A: ML tree based on N-terminal amino acid sequences. The TLR2A/B nodes in duck, chicken, turkey and zebra finch are shown in red, blue, green and orange, respectively. B: ML tree based on amino acid sequences in the central region of TLR2. The avian TLR2A/B nodes are shown in red and orange, respectively. The avian TLR2A and human TLR2 clade is highlighted in pink, while the avian TLR2B clade is in lilac. C: ML tree based on C-terminal amino acid sequences. The TLR2A/B nodes in duck, chicken, turkey and zebra finch are highlighted in red, blue, green and orange, respectively. [file 1471-2148-11-149-S9.JPEG]

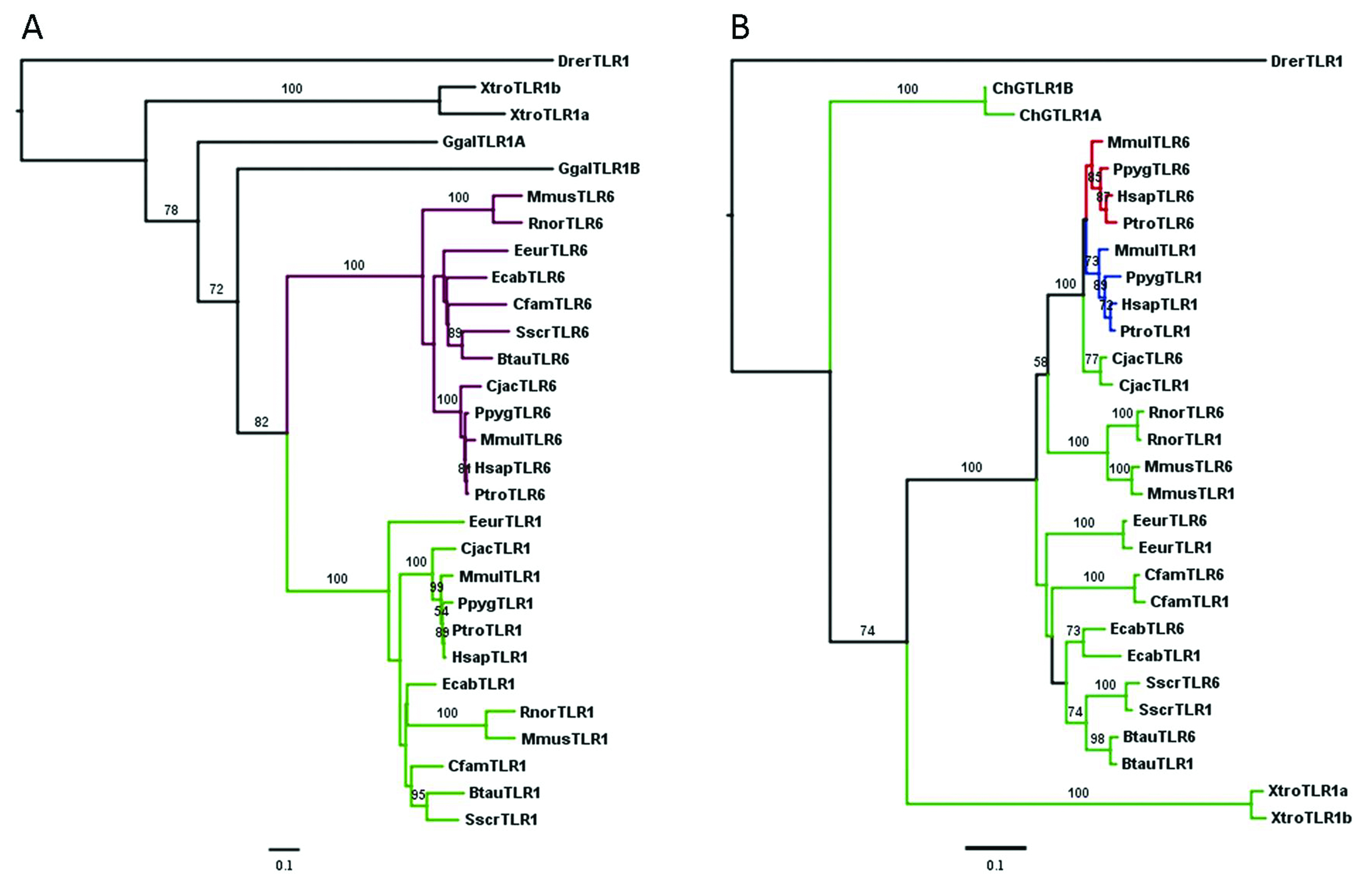

Supplement: Additional file 10 — Figure S8. ML trees of TLR1/6 in mammals based on amino acid sequences either N or C terminal regions. Additional file 10 shows the orthologous relationships based on amino acid sequences from either N or C terminal regions. ML trees of TLR1/6 in mammals based on amino acid sequences from either N or C terminal regions. These trees have been rooted with Danio rerio. Xenopus tropicalis and Gallus gallus are included as outgroups and the sequences are listed in Table 1. The bootstrap values of 1,000 pseudo-replicates are shown as percentages at nodes. Bootstrap values are only shown for nodes with large than 50% support. A: ML tree based on N-terminal amino acid sequences. The mammalian TLR1/6 nodes are shown in green and purple, respectively. B: ML tree based on C-terminal amino acid sequences. The TLR1 and TLR6 nodes in human, chimpanzee, orangutan and rhesus monkey are shown in blue and red, respectively while the TLR1/6 nodes in cattle, dog, hedgehog, horse, marmoset, mouse, pig, and rat are shown in green. [file 1471-2148-11-149-S10.JPEG]

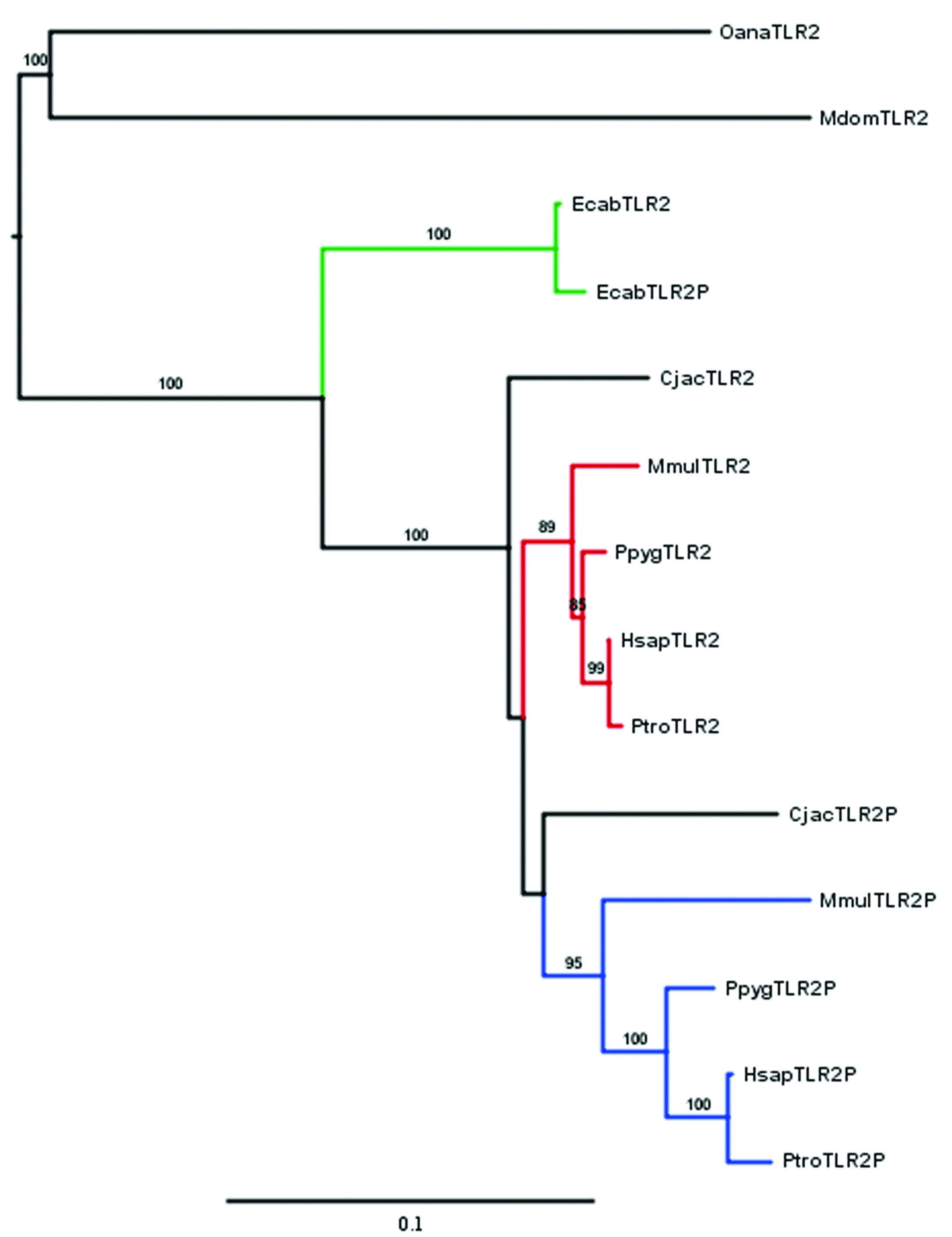

Supplement: Additional file 11 — Figure S9. ML tree of functional and pseudogenised TLR2 genes in mammals based on gap-free, multiple DNA sequence alignments. Additional file 11 shows the orthologous relationships of TLR2 subfamily in mammals based on gap-free, multiple DNA sequence alignments. ML tree of functional and pseudogenised TLR2 genes in mammals based on gap-free, multiple DNA sequence alignments. The tree has been rooted with Ornithorhynchus anatinus. The bootstrap values of 1,000 pseudo-replicates are shown as percentages at nodes. Bootstrap values are only shown for nodes with large than 50% support. The suffixes "C" and "P" represent the homologous regions in TLR2 for functional genes and TLR2 pseudogenes, respectively. The nodes of functional and TLR2P in human, chimpanzee, orangutan and rhesus monkey are shown in red and blue, respectively while the node of functional and TLR2P in horse is shown in green. [file 1471-2148-11-149-S11.JPEG]

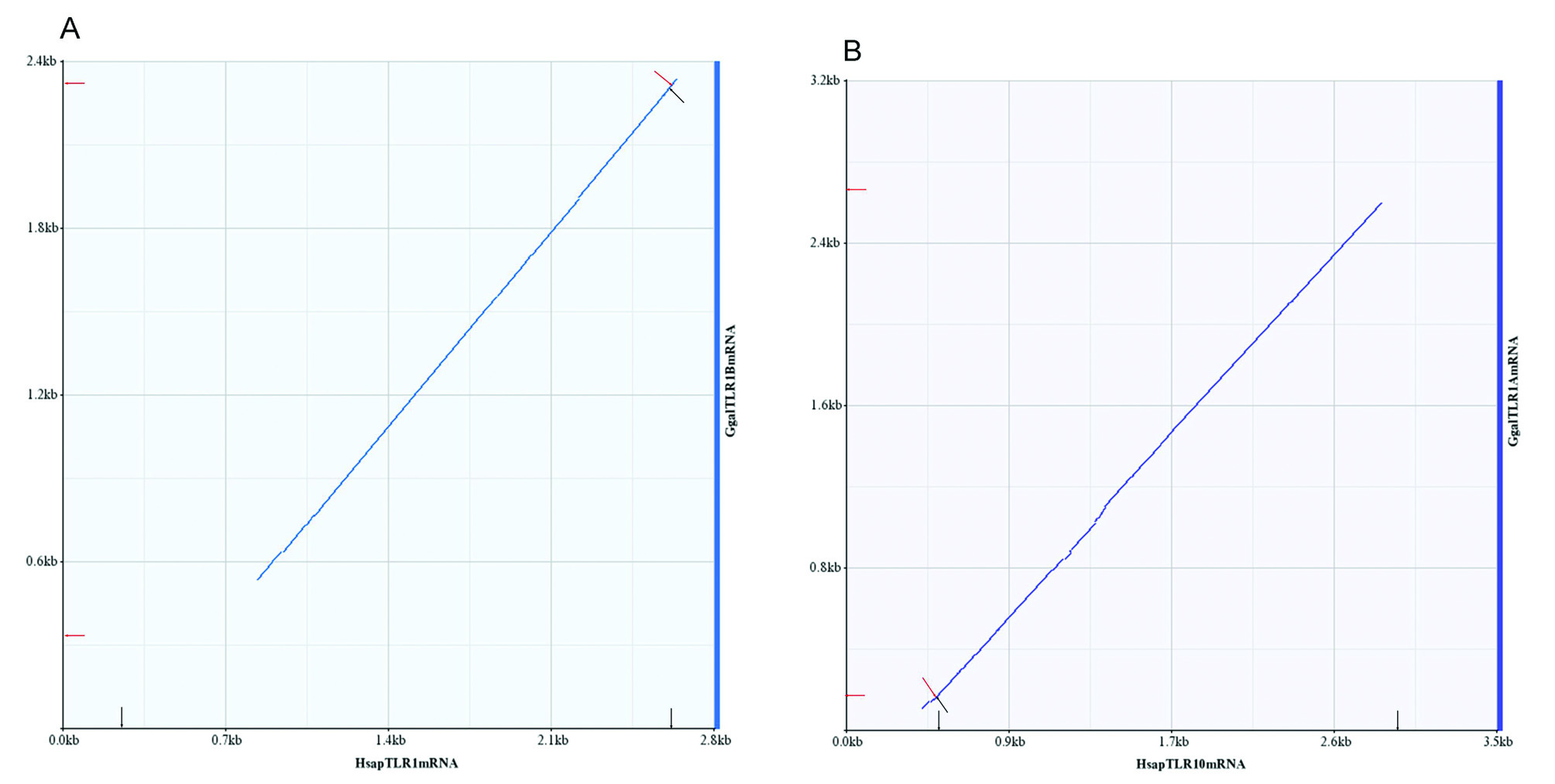

Supplement: Additional file 12 — Figure S10. Dot-plot comparisons of TLR1 subfamily mRNA sequences in human and chicken showing homology of coding and non-coding sequences. Additional file 12 shows the sequence identities of TLR1 subfamily mRNA sequences in coding and non-coding regions of the human and chicken. Dot-plot comparisons of TLR1 subfamily mRNA sequences in the human and chicken showing homology of coding and non-coding sequences. The X-axis represents the sequence from human and the Y-axis the sequence from chicken. The regions between the black and the red arrows are the coding sequences from human and chicken, respectively. A: human TLR1 (coding sequence: 231-2591 bp) compared with chicken TLR1B (coding sequence: 334-2292 bp). B: human TLR10 (coding sequence: 485-2920 bp) compared with chicken TLR1A (coding sequence: 166-2622 bp). [file 1471-2148-11-149-S12.JPEG]

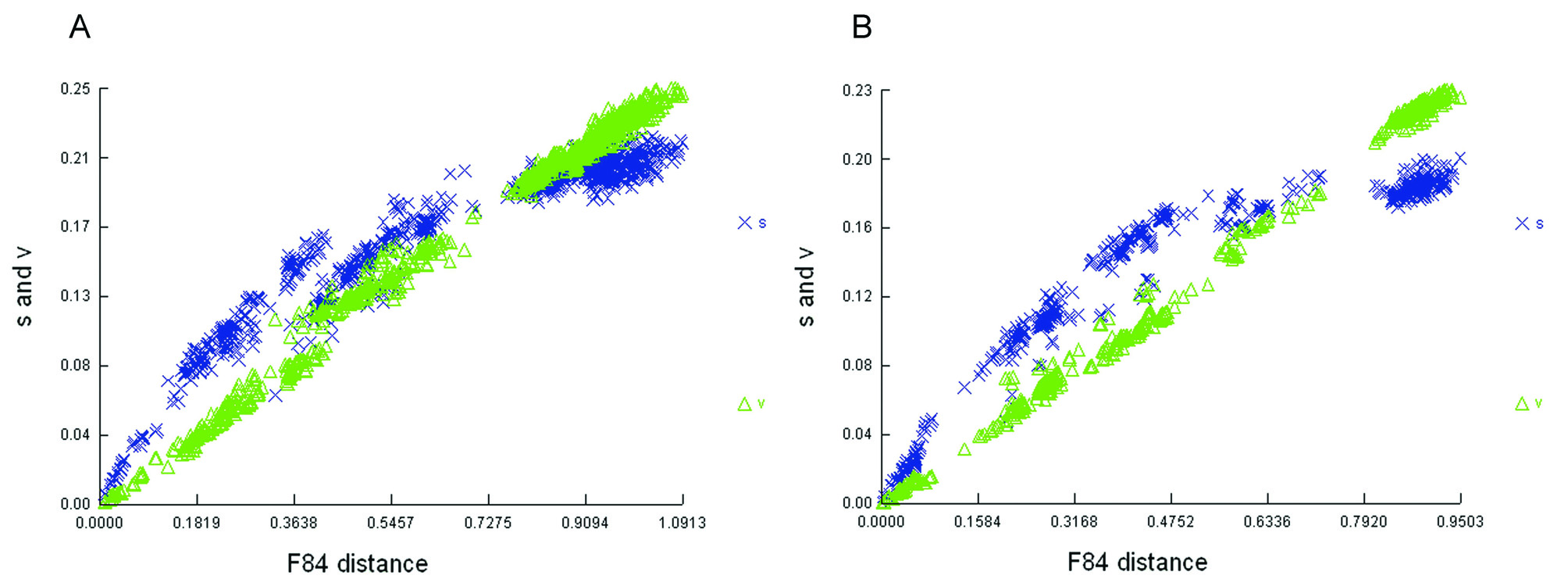

Supplement: Additional file 13 — Figure S11. Plots of transitions/transversions versus genetic distance for TLR1 and TLR2 subfamilies. Additional file 13 shows plots of transitions/transversions versus genetic distance of TLR1 and TLR2 subfamilies. Plots of transitions/transversions versus genetic distance for TLR1 and TLR2 subfamilies. The estimated number of transitions (s) and transversions (v) for each pairwise comparison is plotted against the genetic distance (d) calculated with the F84 model of nucleotide substitution using DAMBE (Xia and Xie 2001). [file 1471-2148-11-149-S13.JPEG]
