# Supplementary material for: Molecular evolution of the vertebrate TLR1 gene family - a complex history of gene duplication, gene conversion, positive selection and co-evolution
Source: BMC Evol Biol. 2011 May 28;11:149. doi: 10.1186/1471-2148-11-149 (PMC3125219; doi:10.1186/1471-2148-11-149)
Supplement: Additional file 4 — The coding and pseudogene sequences of the vertebrate TLR1 gene family. Additional file 4 provides gene name, species, common name, source, length and sequence of the TLR1 gene family from 34 species. [file 1471-2148-11-149-S4.DOC]

>DrerTLR1 Danio rerio Zebra Fish NM_001130593 2388

ATGAAGCCGTCTTCAGGCTGGTGGTTAGTGTCTGTTTATCTCACATGTTTCCATCCGTCTCTCATTCCCGCCATTCAGAGGATCATAGTCAATTACTCCTCTCAGAATCTGTCCTCGGTCCCAGATGACCTTAAACCATCCACCGAGGACCTGGATCTGTCGCTAAACCACATCCAGTCACTAAACTGCAGAGACTTCAACACAACACCACGTCTGCGATTCCTCAATCTGTCCTGGAATATTCTGGAAAACATCGACAGAGACACATTCACATCTACTCCAGCTCTGGAGATGTTGGATTTGTCCCACAACGGACTCCAGAACCTCTCCGAACAGCCGTATCTGCTTCACTTGGGATGTTTGGAGCTTTTAGACCTGTCATCCAATCGGTTTTCAGCCATGGCTTTAGGAGAGGAGTTCTCGATGCTAAAACGCCTGCAGTGGTTGGGATTGAGCGCCAAATCAATCAGCATCCAAGACTTTACTCATATTTCCAATCTCACCTTGAGAACACTCTTCATCAATGCTGACGGGTTATTGACGTATGAGGGGAATAGTTTAGATGACGTACACGCAGAAAAGGCAGTCATCGCCCTTTCAAGTACTAACGTGGATATCGCTATCGCTAATGATGTTTTTGCACGTTTTAAAGAGGTTGAATTCACTAAGGTGGACGGCAAGATGGAGGTAGTCCAGCAGATGCGCAGTCGAGCGCTCATGCGCACTGTTCGATTGGAAATCTCCAATGTGAAAACCACATGGGAATTTCTGACAAGTTCGGTAAACACGATACTATCGTCCACCATTCGTGAGCTTTCCTTAACAGATCTTACCCTGACAGAAATGAAGGATGGTGCTAATCAATCCTCAACCCACATACTGGAGTCATTCTCTACAAAAAGAGCTTCAGTAACCACGTTCATCTTTGACCAAAAAATGCTGTATGACTTCTTTATAAACACACCTGCGAGGAAAGTAAGTCTAACCGAGTCGCCTATAATCTTTATGACCTGTCCTGGGACGATCAGCAAGATCCAGGAGTTGGATTTGTCTGATTGCGCCCTTACAGAAAAAATCTTCTCGGTTAATCCCGAGACAGAATGTGGTACGCTGGTAAACCTGACAAGGCTGGTTTTGAGGGGCAACAACCTAAAGCATCTAAGTCCACTGACTTCAAGAATTAACCTGATGGACTCGCTTCAATACATCGACCTCAGTCAGAACACACTTACCTATTCGGAGAATCAAGGGAGGTGTTTCTGGCCTCCCAGAGTCCTTCATGTAGACTTGTCTAGAAATGGATTCGACGAAGTCGTCTTCAAGTGTTTGCCAGATTCTGTGCAAGTCCTAAACCTTCGGCACAACCGAGTGTCCACAGTTCCTGCAGACATCCACACTTTCGATACCTTGCAAGTCATAGACTTAACATTCAACCGCTTGCTGGATCTTCCCACCTGCCGATCATTCCCAAGCCTACAGAAGCTCCTGATAAGATCCAACTCCATCCATTCTCCTGTACCAGGATCCCTCAAGACTTGTCAACATCTCCAAGATCTCGATTTGAGCCACAACCCTTTCATTTGCACTTGTGCGTTGCGTGATTTTGCCTCTCTGATCAATGCACAGGGAATAAAAACCTTTAAATCAACTCTTAAACACTGGCCAGACGGATACAGATGCAGTTACCCTGAATCCTGGAGCAACTCCACATTGGAAGACTTCTACCTTCCTGAGATCTCCTGCAATGCATGGATTCTGGCTATCACGATTCTAATCCCGACGATCAGTTTGATTGTTGCTGTTAGCCTTCTGTGCAATCGTTTGGATATACCGTGGTACGTCAGGATGATGTGGAAGTGGACTCGCGCCAAGCATTACTCTATAACCTCGCAGCTTAAAGAGGAGGACGTTGAAAGGTTGCACTTCCATGCCTTTGTATCGTACAGCCAAAAAAACGCAGGATGGGTCAAATCTCAATTTCTTCCCAAGCTTGAAGGCGACTGTGGGTTGCGCATGTGTCACCACGAGCGGGACTTCATCCCGGGAAAAACCGTCGTGCAAAACATCCTCCGATGCATCGAGCAGAGCAGAAGGTGTGTTTTCGTGCTGTCGTCTCATTTCGTCCAGAGCGAATGGTGCCACTATGAACTCTACTTCGCCAACCATCAGAAGCTAACGCGAGGCATGGACAGCATACTTCTCATTCTTCTGGAGCCTCTGCCACTGTATCTCATTCCCTCAAAGTACTACCAGCTCAAGACCATGATGTCCAGACGCACTTACCTGGAGTGGCCACAAGAGGGAGCCAAGCAGAAACTGTTTTGGGCAAATCTAAGAGCGGCTCTACAGGCTGAGCTTCCTAATACGCCAGACAGAGAAGAAGAGTGA

>XtroTLR1A Xenopus tropicalis Western Clawed Frog scaffold_394:1,006,666-1,009,056 (xenTro2) 2391

ATGAGAGTCCATGCTGCTGAATACAACTTGTATTTCCTCTTCGCAATTATCATTTACATACTCGCTCAGTTTGAAAGTAATAAAGCCTTGATCATCCAGAGGAAGCTGCATAAATGTTTCTCAAGCTGTCCATCTGTCTGTACTCTGCTGGATTTTACTAAAGAGAACATAACTCATCTTAAAACAAGTGACTTCAATTGTTTTTTACAGTTAAGACTTCTAAATCTTTCACATAATTTAATTGGAGAATTAGACTGTTCAGTGTTTAAGTTCAATCCTTCGCTAGAGTACTTGGATATATCTAACAACAGATTGCATACGATAAAATGTCACTCATTACAATATATTAAAAACATTAAGCGCATGGACCTTTCATACAATAACTTTAAAACCATGGATTTTTGCAGTGAATTTACAGCACTGTCGCAACTGAAACATCTTGGACTGAGTTCTAAGAAGATCCAAAAAGATAGTTTCATAAATATTGCATCTATGGATCTTGATTTTGTGTTTTTGGGGATTGAAAATGAAATTGAATATGAAAATGGAAGCTTACAGTTTCTGAATACAAACAAACTGCATATCAACTTGCAGCCAAACCTAAGTCTGGCAAGTTCTTACCTTTTGAGTGATGCTTTGGACACTTCTATGACTCTCGAGGTCTCTGGGGCACAATGTGATGTACACTGTGATTATTTTACGAAATCTTTTTTCACGATTAATAAAAACTCCAAAGTAGTCAATCTAATTATCAGCAATAGCACAATGCCGGGAAATGAAATGCTTAAAATAATTCCACCTATTTGGGATTCGTCAGTAGAGCATCTACACATCAAAATGTTCAGACTCATAAAGGAGTTTAAGTATGTTAAAATGGACTTTACACATCATTCAATAAAATCTTTTACATTAGAGTATTTTACTAACGATGTGTTTTTTTTCCAAGGAACTCATCCCTTTAACATATTTGCAGAAATGCTTGTTGAAAATGTTACATTCTCAAACGCAGGAATGATACACTTTTTTTGTCCTCCGGTGCCCAGCATTGTCAGGTTTCTAAACCTTCCAAGCAACAGAATTACTGATGAGATTTTTCTAAACTGTTCCAGTCTCAACGAACTGGAATTACTTAATTTGCAAAATAACAAATTAGAGCAAATGTCAAAAATAAGTTCTATGACTCTCAAAATGAAAAATCTCAAGCATTTGGACCTCAGCAAAAATGGACTTCATATTGACAATGAGAAACAATGCAACTGGATGGATTCCCTTGTATTCCTAAATTTATCCGAGAGTGGGCTTACAAATTCGGTCTTTGGTTGCCTTCCCATCAACCTCCAGATACTAGATCTCTCTAAAAATCAAATCTCCAGCATCCCCATAGAAGTAAAGAATTTTGTGTCCCTAAAAGAACTACATTTAGCATCAAACCGTCTAACAGATATCCCTGACTGCCACAATATTGGCAATAACTTGGGGGTCCTAAAAGTTGATGAAAATTTTATAAACTTACCACCAAAAGAATTCCTCCAGAACTGTGAATATGTTCAATATCTAAGTGCTGGACAAAATCCGTTTCAGTGCAACTGTGACCTGAGAGAGTTTGTTAAGATGGGAGTCATGTTTCCAAAAAGGCTTGTTGGATGGCCAGAGTCCTATAAATGTGCAGATCCTGAAAACGTCAAAGGAATTTTCCTTCAAGATTTTCATCTATCTGAAATTTCTTGCAATACATCATTATTTCTAGGAGTTGTACTGGGCACAATTTTTGTAGTGTCAATCATTGTGGTTTCTGCATGTTTTTATTTTGACGTGCCATGGTATATTCGTATGTTGTTCCGATGGTTTAGAACAAAGCACAGGTTAAGGAATGTTAACCAGCAAGACATTCAGAATGACAAACTATTCCATGCATTTATTTCTTACAGTCAGGAAGACTCAGACTGGGTAAAGAATATGTTGCTACCAAATCTTGAAAGAAAAGACGGATCAATAAAAATCTGCCACCATGAAAGGCATTTTATTCCTGGCAAAGCAATTATTGAAAACATAATTGACTGCATTGAAAAAAGTTTCAAGTCCATTTTTGTTTTGTCTCCTAATTTTATTCAGAGTGATTGGTGTCACTATGAGCTGTATTTTGCACAGCACACTTTATTTGGGAAAAATTCCAATAATTTGATTCTGATACTACTGGACCCCATCCCACAGTATTCAATTCCCAATAAGTATAACAAATTAAGATCGATAATGAAGCATAGGACATACCTTGAATGGCCAAAGGAAAAAGGCAAACATGCCCTCTTTTGGGCTAACCTCAGGGAAGCAATTCATGTAAACCTTTCTATTAAAGAGGAAGACATGGCAGACCCTGAAATAACAATAACATGA

>XtroTLR1B Xenopus tropicalis Western Clawed Frog scaffold_394:983,067-985,412 (xenTro2) 2346

ATGTTAAGCATTTACATACTAGTTCAGTCTGAAAGCAGCAAAGCCTCGTTCATCCACAGGAGGCTGCCTAGAGGTCTCTCAAACTGTCCCTCTGCGGATACTCAGCTGGACTTTACTAAAAGGAACATAACTCAGCTTCAAACAAGTGACTTTAGTTGTTTTTTAGATTTGAAATTTTTAAATCTTTCATATAATTCAATTGAAGAACTAGACTGTTCGGTGTTTCAGTTCAATCCTTCTCTAGAGTATTTGGACATATCAAACAACAGATTGCATACGATACAATGTCAATCATTACAATATATTAAACACATTAAGCATCTGGACCTTTCATACAATAACTTTAAACAAATGCATTTTTGCAAAGAATTTACAGCACTGTCCCAACTGAAACATCTTGGCCTGAGTGCCGAGCAGATCCAAACTAACAGTTTTTTAAATATTGCACCTATGCAACTTGAATATGTATTTTTGGGGATGGAAGACTTGACGGAATATGAAAATGGAAGCTTACAATTTCTGAATACAAATAAACTGCTTATCAATTTACCAACAAACCTAAATCTAGCAAGTTCTTACCTTTTGAGTGATGCTTTAAACACCTCCACAACTCTAGAGGTCTCTGGGGCACAATGTGATGTACACTGTGATTATTTCACAAAGTCCTTTTCCACGATTACTAAAAACTCCAAAGTAGTCAATCTAATCATCAGCAATGGTGCAATGCCGGGAAATGAAATATTTAAAATTATGCCGCCTATTTGGGATTCATCAATAGAGCATTTATACATCAAAATCTTCAGACTTCAAAATGAGCTCAAGTATGTTAAAATGGACTTTTTGCATCATTCAATAAAATCTCTTACAGTAGAGGATTTGACTAACGAGGTGGTGTCCTTTCGTGATACTCATCCCCTACAAATGTTTGCAGAAATGCTTGTGGAAAATATAACATTCTCTAGAGCAGAACTGTATTTCTTTTTTTGTCCTCCAGCGCCCAGCATTTTTAGAGTTATAAACCTTCCAAGCAACAGACTTACAGATGATATTTTTCAAAATTGTGCCAATCTCAAAGAATTAGAATTACTGAATTTACAAAATAACAAATTAGAGCAAATGTCAAAAATAAGTTCTATGACTCTCACAATGCCAAATCTCAAGCATTTGGACCTCAGTAGAAATGGACTTCATATTGACAATGAGAAACAATGCAAATGGATGGATTCTCTTGTATTCCTAAACTTATCTGAGAGTGGGCTTACAAATTCGGTCTTTGGTTGCCTTCCCATCAACCTCCAGATACTAGATCTCTCTAAAAATCAAATCTCCAGCATCCCCATAGAAGTTAAAAATTTTGTGTCCCTAAAAGAACTACATTTAGCATCAAACCGTCTAACAGATATCCCTGACTGCCACAATATTGGCAATAACTTGGGGGTCCTAAAAGTTGATGAAAATCTTATAAACTTACCATCAAAAGAATTCCTCCAGAACTGTGAATATGTTAAATATCTAAGTGCTGGAAAAAATCCGTTTCAGTGCAATTGTGACCTGAGAGAGTTTGTTAAGATGGGAGTCATGTTTCCAAAAAGGCTTATTGGATGGCCAGAGTCCTATAAATGTGCAGATCCTGAAAACCTCAGAGGAATTTTCCTTCAAGATTTTTATTTACCTGAAATTTCATGCAATATTTCAATGCTTCTAGGAGTTGTACTGGGTACAATATTTATTTTATCAATCATTGTGGTTTCTGCATGTTTTTATTTTGACGTGCCATGGTATATTCGTATGTTGTTCCGATGGTTTAGAACAAAGCACAGGTTAAGGAATGTTAACCAGCAAGACATTCAGAATGACAAACTATTCCATGCATTCATTTCTTATAGTCAGGAAGACTCAGACTGGGTAAAGAATATGTTGCTACCAAATCTTGAAAGAAAAGACGGATCAATAAAAATCTGCCACCATGAAAGGCATTTTATTCCTGGTAAAGCAATCATTGAAAACATAATTGACTGCATCGAAAAAAGTTTCAAGTCCATTTTTGTTTTGTCTCCTAATTTTATTCAGAGTGATTGGTGTCACTATGAGCTGTATTTTGCACAGCACACTTTATTTGGGAAAAATTCCAATAATTTGATTCTGATACTACTGGACCCCATCCCACAGTATTTAATTCCCAATAAGTATAACAAATTAAGATCGATAATGAAGCATAGGACATACCTTGAATGGCCAAAGGAAAAAGGCAAACATGCCCTCTTTTGGGCTAACCTCAGGGAAGCAATTCATGTAAACCTTTCTATTAAAGAGGAAGACATGGCAGACCCTGAAGTAAGAACATAA

>XtroTLR10 Xenopus tropicalis Western Clawed Frog scaffold_394:997,310-999,838 (xenTro2) 2529

ATGGCTGCAATATTCTTTCTGCTCATTTACTTATCTTCAAGTGGACAGTGTGTCAGGCAGGAAATATTTGAAAGCCATATAGCAAATTACTCCAACAAATTTCTTTTTGCTGTTCCAAAGAACCTTTCATCACTGACGACTGTGCTGGATATCTCATTTAATTCTATAGCAACAATGGAAACATCTGAATTTAACTATATATTTGATCTACAAGTGTTGAATGCATCTTACAATAAAATGAAGTATCTAAATAGCAGTATTTTTAAATTTAACACCCAACTGCAGTATCTTGACTTTTCCCACAATGTACTAAGAAATATCTCTGGTACATTTCCAAGTCATATTCAACATTTGGACATTTCTTTCAATAATTTTCAGACTTTATCTGTGTGCAGTGGATTTGGAAACATGGTTCTACTGGAATATCTTGGGCTTGGTGCGGATCAGATTCTAAAATCTGATTTTGAGGGGATTGTGCATTTGCACCTGAAAGAAGTGTTCATTGAGTTAAACTCCCTAAATGATTATGAAAATGGCAGCTTATTATTACTGAACACAAAGAAGCTTACGCTTGACTTTGGTGTACTATCCAATGAAGACCGCTACAATGTGTTATTTGATGCTGTCAACACAACCAGCGTTCTAGAACTGTCGCATTTACAGAGATGGAATATTCACATTGATCCAAAGAAATATATTTTCAATATTGTACAAAATTCAAAAGTAACAGATCTAACAATGAGGCATGTTAATGTCGAATGGAAAATGATTGTCCTGGCACTTCAGTATATCTGGCATTCCTCTGTTGAAACACTGACGTTCTATGATTTCTCTCTCAAAGGATTAATAGCAAAAACATCCTTTGATTATTCTAATACATCAGTGAAAGCAGTTAATGTGCAAGGTGTGAATGTTGGAGTTTTTCAGTTTGACCAAAGCTCTGTATATAGGGTCTTTTCTAATATGAATATTGAAAACCTGACGTTAAACTTTGCTAGCTTACTGTTTATGGTGTGTCCTCTCAATTCCAGCACCTTTCAGAGTATTGATTTTTCACACAATGCCCTGACAGACGACCTTTTCAAAGACTGCTTTACATTAATAAATCTAAAAAGATTAAAATTGAGAAAAAATAAACTTGAAAAAATATACAAACTGAGTGCTATGACAGAGAATATGCCCTCTTTGGAGTATTTGGATGCAAGTCATAATTTATTGGCCTATGATGAGGAGGACTGCCACTGGTCTCAAAATATTGTCGAGCTGAATTTGGCATCAAATTTTTTGACAGCATCTATTTTTAAATGTTTGCCAATTAGTATTAAAATTTTGATACTTGAGAGCAATGACATCACACATGTTCCAAGCGGTGTAACCCATTTAGATGACTTGGTAGAACTTAACCTTTCATTCAATCGGCTGGGTGATTTACCAGACTGCAGTAATATTAGTAGTCTTAGTCTTCTTGGTGTTGAGGGTAACCAGATCCTATATCCCTCAATTGAATCTATTGAGAGTTGCAGTCGAGTAAAACACATAAGTGCTGGCCAGAACCCATTTCAGTGTAATTGTGAATTAAGAAGGTTCATTAACCAAGAAAAAGAGGCGCCAGGAACATTGATTGGGTGGCCAGAAGCTTATGTTTGCAAGTATCCTGACCATCTCAGGGGAACCATGTTAAAAGATTTTTACATATCAGAAATAACATGCAATGTATTCATTCTGATACCTGTTATAATCTTGCCAATCATTATTTCCATAGCCCTTATTATTGGCCTTTGTAAATACCTTGATGGGCCATGGTTTTTGAAAATGATCTGGCAATACACCAGAACCAAGCAGAGAACAAGAACTAGTAAACAAGGTTACCAGTCACTCCAAAGGGATTTTGACTTTAATGCATTCATCTCTTACAGTGAACATGATGCATCTTGGGTAAAGAACATATTCTTACCTAGCATTGAAAGAAGCAATGACTGTATACGAATCTGCCAGCATGAGAGAAATTTTATCCCTGGTAAAAGCATAATTGAGAACATAATAAACTGCATTGAGAAAAGCTATAAATCAATTTTTATTTTATCTCCCAATTTTGTTCAGAGTGAGTGGTGCCACTATGAACTCTACTTTGCTCACCACAAATTGTATACTGAAAACAATGACAACCTAATCCTGATTCTGCTTGAACCGATCCCCCAGTACCTGATTCCCTCTAAATACTACAAACTGAAAACTCTAATGGCTCAAAGAACATACTTAGAATGGCCATCTGAGAAAAGCAAGCATGGCCTTTTTTGGGCTAATTTAAGGGCAGCCATTAGTATAGATTTAACTCATGCAGAGAGTGAAATTCCTTACTCAATTTCAAGTGAAAATCCTTGCTCAATTTCAAGTGAAAATCCTCACTCAATTTCAAGTGAAAATCCTTACTCAATTTCAAGTGATAATCCTTGCTTAGGGACCAGTGAAAATCCTTGCTCAATTTCAAGTGAAAATCCTTACTCAATTTCAAGTGAAAATCCGTGCTAA

>GgalTLR1A Gallus gallus Chicken NM_001007488 2457 ATGGGATCCCTTACAAGTATCTACGTTTTTGCTTGTGTGTTTTTATCCATACTTTGGAATAATATCCAGCCAACTGTGGAAAATAAAATCACTGCAAACTATTCAGGACATTTACTAACTGAAGTTCCAAAAAACATTCCAGTCCATACTCACATATTAGATTTATCACATAATAGCATCTCTGAAATTACTAACTTCAGATTTACTTCTCTTTCTGACCTTCAAGTATTAAACCTTTCTCATAATCTAATTACAGAGCTTGACTTTAGTGCCTTCATGTTTAATCAAGATTTAGAATACTTAGATTTATCTCACAATAACATTTGGACAGCTTACTGCCAATTGCTTGCACGTCTTCGACATTTAGATCTTTCTTTCAACAAGTTTACTGTCTTGCCAATCTGTCAGGAATTTGGGATCATGTTCCATTTAGAATATCTAGGATTAAGTGCCATGATGATACGAAGGTCAGACTTCAGATATGTGGCACATTTGCAGCTGGACACGGTCTTCCTAACCTTAGAAGACTTTTCTCTGTATGAGCCTCTAAGTCTGACAGCTTTAAATACACGGAGCCTTCACATTGTTTTTGCAACAAACCAAAACTTCAATTTTTCCCTCTTATATGATGGAATGAGCACTTCAGAAAAGTTAAAAATAGTTAATTTAAGATATACCTTGAGCCACAAAGATTTTCCCTCCCCTTCTTTAGAGCTTCAGAAGAAAATCAAAACAACAGATCTAACGCTTGACACTGTGGACTTAGAATGGACTGTCATTCTGCAAATTTTTCTGCTAGTTTGGGATTCATCTGTGGAGCACTTAACTGTGAGAAATTTGATTTTTCGGGGACCAGTGGTGGAGCTGACTGAATATAAACATGTACCCCTTTTAAGATCTCTGGAACAATTACTGTCCTTGGGTAGCTCCATGAAGGCACTAACTTTGGAGCGTGTTCGAAATAAGTTGTATTATTTCAACCAGGAGATTTTGTACAGACAGTTTTCAGAGATGAATATTGATAGTTTGACAATACATGATGCATGTATGCCGCACATGCTTTGCCCAAAAAAACGAAGCTCATTTCAGTATATAAATTTTTCCCGCAATGCCCTGACAGACGAATTGTTCCAGAATTGTGACACTCTGGCAAATTTGAAAATACTTATTTTGCATAGGAATAAATTTGAGAGCCTTTCCAAGGTGAGCTTCATGACCAGCCGTATGAAATCACTGAGGTATCTGGACATGAGCAGCAACTTGCTGCGTAACAGTAGAGCTGAGGGGCGCTGTCAGTGGGCTGATTCTCTGGCAGAGCTGGACCTGTCCTCCAACCAGCTGACAGAGGCTGTGTTTGAGTGCTTGCCAGCTAATATCAACAAAGTGGACCTACAAAACAACCAGATTGCCAATGTCCCCAAGGGGATCACTGAGCTGCACTCATTGCAGGAGCTGAACCTGGCATCCAACCGGCTGGCCGACCTGCCAGGGTGCAGAGCCTTTACAGGCCTGGAGATCCTGAACATAGAGAGGAATTTGATCCTCACCCCATCTGCTGACTTCTTTGAGACCTGCCCGAGCGTGAAGGAGCTGCAAGCTGGGCAAAACCCGTTCAAGTGTTCGTGTGAGCTGCAGGACTTCCTGCGCCTGGAGAGGCAGTCTGGGGGCAAGCTGTCCGGCTGGCCAGAGGCGTACGTGTGCAAGTACCCAGAAGACTTGAGCGGAACGCAGCTTGAGGACTTCCACTTGACCGAGCTGGCTTGCAACACGACTCTGCTGCTTGTGACAGCTCTGCTGCTGACCCTGGTGCTGGTGGCTGTGGTGGCCTTTCCGTGCATCTACCTGGATGTGCCGTGGTACGTGCGGATGCTGTGGCAGTGGACGCAGACGAAGAGGAGAGCTTGGCACGACTGCCCCGAGGAGCGGGAAACCGCTCTGCAGTTCCACGCCTTCATTTCCTACAGCGAACGCGATTCCCTGTGGGTGAAGAATGAGCTGATCCCCAATTTGGAGAAGGGGGAGGGCTGCATCCAGCTGTGTCAGCATGAGAGGAACTTTATCCCTGGCAAAAGCATTGTGGAGAACATCATTAACTGCATTGAGAAGAGCTACAAGTCCATCTTTGTGTTGTCGCCCAACTTTGTGCAGAGCGAGTGGTGTCACTACGAGCTGTACTTTGCCCATCACAGGTTGTTTAGTGAGAATTCCAACAGCTTAATCCTGATCTTGCTGGAGCCGATCCCTTCATACGTTATCCCTGCGAGGTACCACAAGCTGAAGGCTCTCATGGCAAAGAGAACGTACCTGGAGTGGCCAAAGGAGAGGAGCAAGCATGCCCTTTTCTGGGCTAACCTGAGGGCAGTTGTTAACATTAAGCTGCCGACATCCTTTGAAACAGATGAGGAGCAAAGTGATGTCACATCTACCAGTAGTATAACCCAATGTCTGATTAAGTGA

>GgalTLR1B Gallus gallus Chicken DQ518918 1959

ATGACAAAGAATATGAGATATCTCAGGAACTGTTTTATTTACAACTGTCTGTTTGTATTCACTTTCTGGGACAATATTGGCCTGGCTAAGAAAAATGAACTCTTTGCATCTGTTCCTAACAATTTTCCAGAAGATGGTTTGGACAAAAAAAATATGAGTTTTCCACACTCATACGCAAATAATCAGCACTACAAAGCTGATTATGGTTGGGTTATGATAGAAAATACTACGGAAAGCCTATCATTGTCAGAAATCGCAGATGACAATGTAAGAAAATTAATAACTTTACTATCTAAATTTAGAAAAGGCTCCAGGCTACGAAATCTGACACTGACAAATATGTCAGTGGACTGGAAAGATATTATTAAAGTACTTCAGGTTGTATGGCACTCATCCATTGAATACTTCAATATCAACAACTTAACACAACTGGGGAACGTCGTAAGTACTCGCTTCGACTATTCAAAAACATCCATGAAAGCATTTGCAGTGAATAAGGTTTTAATCACAGATCTCTACTTTTCACAGGATGACGTTTATAACATATTTGCAAACATGAATATTGCAGCCTTGACAATAGCTGAATCTGAGTTAATTCATATGCTGTGTCCTTCATCTGACAGTCCCCTTAGATATATAAATTTCTCAAAGAATGATTTAACAGATCTGCTATTTCAGAACTGTGACAAACTAATTCAACTGGAGACATTTATTTTGCATAGGAATAAATTTGAAAGCCTTCCCAAGGTGAGCTTCATGACCAGCCGTATGAAATCACTGAGGTATCTGGACATGAGCAGCAACTTGCTGCGTAACAGTAGAGCTGAGGGGCGCTGCCAGTGGGCTGATTCTCTGGCAGAGCTGGACCTGTCCTCCAACCAGCTGACAGAGGCTGTGTTTGAGTGCTTGCCAGCTAATATCAACAAAGTGGACCTACAAAACAACCAGATTGCCAGTGTCCCCAAGGGGATCACTGAGCTGCACTCATTGCAGGAGCTGAACCTGGCATCCAACCGGCTGGCCGACCTGCCAGGGTGCAGGGCCTTTACAGGCCTGGAGATCCTGAACATAGAGAGGAATTTGATCCTCACCCCATCTGCCGACTTCTTTGAGACCTGCCCGAGCGTGAAGGAGCTGCAAGCTGGGCAAAACCCGTTCAAGTGTTCATGTGAGCTGCAGGACTTCCTGCGCCTGGAGAGGCAGTCTGGGGGCAAGCTGTCCGGCTGGCCAGAGGCGTACGTGTGCAAGTACCCAGAAGACTTGAGCGGAACACAGCTGAAGGACTTCCACTTGACCGAGCTGGCTTGCAACACAACTCTGCTGCTTGTGACAGCTCTGCTGCTGACCCTGGTGCTGGTGGCTGTGGTGGCCTTTCTGTGCATCTACCTGGATGTGCCGTGGTACGTGCGGATGCTGTGGCAGTGGACGCAGACGAAGAGGAGAGCTTGGCACGACTGCCCCGAGGAGCGGGAAACCGCTCTGCAGTTCCACGCCTTCATTTCCTACAGCGAACGCGATTCCCTGTGGGTGAAGAATGAGCTGATCCCCAATTTGGAGAAGGGGGAGGGCTGCATCCAGCTGTGTCAGCATGAGAGGAACTTTATCCCTGGCAAAAGCATTGTGGAGAACATCATTAACTGCATTGAGAAGAGCTACAAGTCCATCTTTGTGTTGTCGCCCAACTTTGTGCAGAGCGAGTGGTGTCACTACGAGCTGTACTTTGCCCATCACAAGTTGTTTAGTGAGAACTCCAACAGCTTAATCCTGATCTTGCTGGAGCCAATCCCTCCATACGTTATCCCTGCGAGGTACCACAAGCTGAAGGCTCTCATGGCAAAGAGAACGTACCTGGAGTGGCCAAAGGAGAGGAGCAAGCATGCCCTTTTCTGGGCTAACCTGAGGGCAGCTATTAGCATCAATCTATCAGTGGCTGACGAACAGAACAGAACAGAAGTGTGA

>MgalTLR1A Meleagris gallopavo Turkey FJ477857 2457

ATGGGATCTCTTACAAGCATCTATGTCTTTGCTTGTGTGTTTTTATCCATACTTTGGAATAATATCCAGCCAACTGTGGAAAATAAAATCATTGCAAACTATTCAGGACATCTACTAACTAAAGTTCCAAAAAACATTCCAGTCCATACTCACGTATTAGATTTATCACATAATAATATCTCTGAAATTACTAACTTGAAATTTATTTATCTTCCTGACCTTCAAGTATTAAACCTTTCTCATAATCTAATTACAGAGCTTGACTTCAGTGTCTTCATATTTAATCAAGATTTAGAATACTTAGATTTATCTCACAATAACATCTGGACAGCTTACTGTCAAATGCTTGCACGTCTTAGACATTTAGATCTTTCTTTCAACAAGTTCACTGTCTTGCCAGTCTGTCAGGAATTTGGGATCATGTTTCATTTGGAATACCTCGGATTAAGTACCAGGATGATACGAAGGTCAGACTTCAGATATGTGACACATTTGCAGCTGAACACTGTCTTCCTAACCTTAGAAGACTTTTCTCTGTATGAGCCTCTAAGTTTGACAGCTTTAAATACAAAGAACCTTCACATTGTTTTTGCAGCAGATCAAAACTTCAATTTTTCCCTCTTATATGACGGAATGAGCACTTCAGAGAAGTTAAAAATATTTAATTTAAGATACACCTTGAGCCACAAAGATTTGCCCTCCCCTTCTTTAAAGCTTCTGAAGAACATCAAAGCAACAGATCTAATGCTTGAAATTGGGGACTTACAATGGACCATTCTTCTGCAAATTTTTCTGCTAGTTTGGGATTCAACTGTGGAGCACTTAACTGTGAGAAATCTGATTTTTCGGGGACCAGTGATGGAGCTGACTGAATATAAATTTGCTCCCCTTTTAAGACCTCTGGAACAATTACTCTCTTTGGGTAGCTCCATGAAAGCACTTACTTTGGAGCATGTTCGAAATAAGGTGTATTATTTCAACCAGGAGCATTTGTACAGATTGTTTTCAGAGATGAATATTGATAGTTTGACAATATATGATGCATGTATGCCACACATGCTTTGCCCCCAAAAAACAAGCTCATTTCAGTATATAAATTTTTCTCGCAATGCCCTGACAGATGAATTGTTCCAGAATTGTGACACCCTGGCAAATTTGAAATTACTTATTTTGAATAGGAATAAATTTAAGAGCCTTTCCAAGGTGAGCTTCATGACCAGCCGTATGAAATCACTGAGGTATCTGGACATGAGCAGCAACTTGCTGCGTAACAGTGGAGCTGAGAAGCGCTGCCAGTGGACTGATTCTCTGTCGGAGCTGGACCTGTCCTCCAACCAGCTGACAGAGTCGGTGTTTGAGTGCTTGCCCGTTAATATCAACAAACTGGACCTACAAAACAACCAGATTGCCAGTGTCCCCACGGGGATTGCTGAGCTGCAGTCCTTGAAGGAGCTGAACCTGGCGTCCAACAGGCTGGCCGACCTGCCAGGGTGCAGGGCCTTTGCGGGCCTGGAGATCCTGAACATAGAGAGGAATTTGATCCTCACGCCAAGTGCCGACTTCTTTGAGAGCTGCCCGAGCGTGAAGGAGCTACAAGCTGGGCAAAACCCGTTCAAGTGTTCGTGTGAGCTGCAGGACTTCCTGCGCCTGCAAAGGCAGTCTGGGGGCAAGCTGTCCGGCTGGCCAGAGGCGTACGTGTGCAAGTACCAAGAAGAATTGATGCGAACGCAGCTGAAGGACTTCCGCCTGAACGAGCTGGCTTGCAACACGACTCTGACCTTCGTGACAGTCTCGCTGCTGACCCTGGTGCTGGTGGCTGTGGTGGCCTTTCTGTGCATCTACCTGGATGTGCTGTGGTACGTGCGGATGCTGTGGCAGTGGACGCAGGCAAAGCGGAGAGCTTGGCACGACTGCCCCGAGGGGCGGGAAACCGTTCTGCAGTTCCACGCCTTCATATCGTACAGAGAGCGCGATTCGTTGTGGGTGAAGAATGAGCTGATCCCCAATCTGGAGAAGGGGGAGGGCTGCATCCAGCTGTGTCAGCACGAGAGGAACTTTATCCCTGGCAAAAGCATCGTGGAGAATATCATTAACTGCATTGAGAAGAGCTACAAGTCCATCTTTGTGTTGTCCCCCAACTTTGTGCAGAGCGAGTGGTGTCACTACGAGCTGTACTTTGCCCATCACAAGTTGTTTAGCGAGAACTCCAACAGTTTAGTCCTGATCTTGCTGGAGCCGATCCCTCCATATGTTATCCCTGCGAGGTACCACAAGCTGAAGGCTCTCATGGCCAAGAGAACCTACCTGGAGTGGCCGAAGGAGAGGAGCAAGCGTGCCCTTTTCTGGGCTAACCTGAGGGCAGCTATTAGCATTAAGCTGCCAACATCCTTTGAAACTGATGAGGAGCAAAGTGAGGTCACATCTACAAGTAGTATGACCCAACCTCTTATTAAGTGA

>MgalTLR1B Meleagris gallopavo Turkey FJ477858 1959

ATGGCAGAGAACATGAGATATCTCAGGAACTTTTTTATTTACAACTGTCTGTTTGCATTCACTTTTTGGAACAATATTGGCCTGGCTATGAAAAATGAACTCTTTACATCTGTTCCTAACAATTTTCTACAGGATGGTTTGGACAAAAAAAACTTGAGCCTTCCACACTCATATACAAATAATCAGCAGTCCAAAGCTGATTTTGGTTGGGTTGTGATAGAAAATACTACGGAAAGCCTATCATTGTCAGAAATCAAAAAAGACAATGTAAGAAAATTAATAACTTTATTATCTAATTTTAGAAAAGGTTCCAGGCTTCAAAATCTGACACTAACGAATATGTCAGTGGACTGGAAAGATTTTATTAAAGCACTCCAGACTGTATGGCACTCATCCATTGAATACTTCAGTATCAACAACTTAATACAAGAGGGGAACGTCAGAAGTTCTCGCTTCGACTATTCAGATACTTCCATGAAAGCACTTGCACTGAATAATGTTAGAATCACAGACCTATACTTTTCACAGGATGACATTTATAACATATTTGCAAACATGAACATTGAAGCCTTGACAATAGCTGATTCTGAGTTAATTCACATGGTATGTCCTTCATCTGACAGTCCATTTAGGTATGTAAATTTCTCAAAGAATGATTTAACAGATCTGCTTTTTCAGAACTGTGACAAACTAATTCAACTGGAGACATTTATTTTGAATAGGAATAAATTTAAGAGCCTTTCCAAGGTGAGCTTCATGACCAGCCGTATGAAATCACTGAGGTATCTGGACATGAGCAGCAACTTGCTGCGTAACAGTGGAGCTGAGAAGCGCTGCCAGTGGACTGATTCTCTGTCGGAGCTGGACCTGTCCTCCAACCAGCTGACAGAGTCGGTGTTTGAGTGCTTGCCCGTTAATATCAACAAACTGGACCTACAAAACAACCAGATTGCCAGTGTCCCCACGGGGATTGCTGAGCTGCAGTCCTTGAAGGAGCTGAACCTGGCATCCAACAGGCTGGCCGACCTGCCAGGGTGCAGGGCCTTTGCGGGCCTGGAGATCCTGAACATAGAGAGGAATTTGATCCTCACGCCAAGTGCCGACTTCTTTGAGAGCTGCCCGAGCGTGAAGGAGCTACAAGCTGGGCAAAACCCGTTCAAGTGTTCGTGTGAGCTGCAGGACTTCCTGCGCCTGGAAAGGCAGTCTGGGGGCAAGCTGTCCGGCTGGCCAGAGGCGTACGTGTGCAAGTACCCAGAAGACTTGAGCGGAACGCAGCTGAAGGACTTCCACCTGACCGAGCTGGCCTGCAACACGACTCTGCTGCTCGTGACAGCTCTGCTGCTGACCCTGGTGCTGGTGGCTGTGGTGGCCTTTCTGTGCATCTACCTGGATGTGCTGTGGTACGTGCGGATGCTGTGGCAGTGGACGCAGGCAAAGCGGAGAGCTTGGCACGACTGCCCCGAGGGGCGGGAAACCGTTCTGCAGTTTCACGCCTTCATTTCGTACAGCGAGCGCGATTCGTTGTGGGTGAAGAACGAGCTGATCCCCAATCTGGAGAAGGGGGAGGGCTGCATCCAGCTGTGTCAGCACGAGAGGAACTTTATCCCTGGCAAAAGCATCGTGGAGAATATCATTAACTGCATTGAGAAGAGCTACAAGTCCATCTTTGTGTTGTCCCCCAACTTTGTGCAGAGCGAGTGGTGTCACTACGAGCTGTACTTTGCCCATCACAAGTTGTTTAGCGAGAACTCCAACAGTTTAGTCCTGATCTTGCTGGAGCCGATCCCTCCATATGTTATCCCTGCGAGGTACCACAAGCTGAAGGCTCACATGGCCAAGAGAACCTACCTGGAGTGGCCGAGAGAGAGGAGCAAGCGTGCCCTTTTCTGGGCTAACCTGAGGGCGGCTATTAGCATTAATCGTTCAGTGCCTGTTGAACAGAACAGAACAGAATTGTGA

>AplaTLR1A Anas platyrhynchos Beijing Duck (Mallard) FJ477859 2457 ATGGGATCCCTTACAAGTATCTATATCTTTGCTTGTGTCTTTACACTCACACTTTGGAATAATGTCCAGCCAACTGTGGAAAACGAATTCATTGCAAACTATTCAAGCCTTTTGCTAACTGATGTTCCAAAAACCATTCCACTCCATACTCGTGTATTAGATTTATCACATAATAGGATCTCTGAACTTAGTATCTCTGAATTTATTTCTCTTTCTGACCTTCAGGTATTAAATCTTTCTCATAATCTAATTACAGAGCTTGACTTTAATGTCTTCATTTTTAATCAAGATTTAGAATACTTAGATCTATCTCATAATAACATTTGGAAAGTTTATTGTCAAACGCTTGCATGTCTTAGACATTTAGATCTTTCTTTCAATAATTTTACTGTCTTGCCCATCTGTCAGGAATTTGGGACCATGTTTCATTTGGAATATCTAGGATTAAGTGCCACAATGATACGAAGGTCAGACTTCAGATACATCACACATTTACAGCTGAACACTGTCTTCCTTACCTTGGAAAACTTTTCTCTGTACAAGCCTCAAAGTCTAACAGCCTTAAATACAAAGAGCCTTCACATTGTTTTTTCAGCAAACCAAAACTTCAATTTTTCCCTCTTGTATGATGGGATGAGCACTTCAGAAAACTTAAAAATAGTTAATGTAAGATATACCTTGAGCTACAAAGATTTCCCCTCTCCTGCTTTAACGCTTCTGAAGAAAATCAAGACAACGGCTCTGATGCTTGACACTGTGGATTTAGAATGGCCTATCATTTTGCAAATTTTCCTGCTTATTTGGTATTCACCTGTGGAGCATCTGACTGTGAGAAATTTGATTTTTCGGGGACCAATAGGAGGGTTGACTGCATATAAATTTGTACCCTTCTTAAGCTCTCTGGAACAAGTAATGTCTTTGGGTGTCTCCATGAAAGCGTTAACTTTGGAGCGTGTTCGTAATAAGGTTTATTATTTCAACCAGAAGATTCTATACAGGTGGTTTTCAGAGATGAATATTGCCAGTTTGACAATATATGATGCATACATGCCACACATGCTTTGTCCGCAGAGAGCAAGCTTATTTCAATATTTAAATTTTTCTCGCAATGCCCTGACAGATGAATTGTTCCAGAATTGTAGTACTCTGGCAGAGTTGAAGTTATTTATTTTGAGGAGGAATAAATTTGAGAGCCTTTCCAAGGTGAGCTCCATGACCAGGTACATGAAATCACTGAGGTATCTGGACATGAGCAGCAACTTGCTGCATACCGATGGAGCCGAGGAGCACTGCCAATGGACTGAGTCTCTGAAGGAGCTGGACCTGTCCTCAAACCAGCTGACAGAGTCCGTGTTCGGGTGCTTGCCGGTCAATGTCAACAAACTGGACCTACACAACAACCAGATCAGCAGCGTCCCCCAGGGGATTGCTGAGCTGAAGTCCTTGAAAGAGCTGAACCTGGCGTCGAACAGGCTGGCCGACCTGCCGGGGTGCGGTGGCTTCTCGGCCCTGGAGATCCTGAATATGGAGATGAACTCGATCCTCACCCCTTCCGCCGACTTCTTTGAGAGCTGCCAGAGGGTGCGGGAGCTGGAAGCCGGGCACAACCCGTTCAAGTGCTCCTGTGAACTGCAGGCCTTCGTGCGTCTGGAGAGGCAGTCTGGGGGGAAGCTGTCCGGCTGGCCGGAGGCATACGTGTGCGAGTACCCCGAGGACCTGAAGGGAACGCAGCTGAAGGACTTCCACTTGACGGAGCTCGCTTGCAACACGACCCTGTTGCTTGTGACAGCTCTGCTGCTGACGCTGGTGCTGGTGGGGGTGGTGGCCTTTCTGTGCATCTACCTGGACGTGCCGTGGTACGTGCGCATGCTGTGGCAGTGGACGCAGACGAAGCGCAGAGCTTGGCACGAGTGCCCCGAGGAGCGGGAAACCGTCCTGCAGTTCCACGCCTTCATTTCCTACAGCGAGCGCGATTCCGTGTGGGTGAAGACCGAGCTGATCCCGAACCTGGAGAAGGGGGAGGGCAGCGTCCAGCTGTGCCAGCACGAGAGAAACTTTGTTCCCGGCAAGAGCATTGTGGAGAATATCATTAACTGCATAGACAAGAGCTACAAGTCAATCTTTGTGTTGTCTCCCAACTTTGTGCAGAGCGAGTGGTGTCACTACGAGCTCTACTTTGCCCATCACAAGTTGTTCAGTGAGAACTGCAACAGCTTGATCCTGATTTTGCTGGAGCCTATTCCTCAGTATATTATCCCTGCGAGGTACCACAAGCTGAAGGCTCTCATGGCAAAGAGAACGTACCTGGAGTGGCCAAAGGAGAGGAGCAAGCGTGCCCTTTTCTGGGCTAACCTGAGGGCAGCTATTAACGTTAACCTGCCAATGTCATTTGAAGGAAATGAGGAGGAAAATGATGTTACTTTTACTGATAGTATAACTCAGCCTCTGATTAAGTGA

>AplaTLR1B Anas platyrhynchos Beijing Duck (Mallard) FJ477859 1953 ATGAGAGATCTCCAGAACTTTTTTGTTTATGAGTGTCTCTTTGTTTTAACTTTTTGGAACAATATCAGCCTGTCTGTGGAAGATGAACTCTTTACACCTGTTTCTAATAATTTTCCAGAAGGTGGTTCTGAGAAAAACATCATGAGCCTTCCACTGTTGTATAATCATCATCATCAGCAGTCCAAAGCTGATTACAATTGGGTTGTAATAGAAAACACTACAGAAAGCCTGTCATTGTCACAAATCACTAATAGCAATGTAAAAAAATTAATAACTTTGTTGTCTGATTTCGGAAAAGGCTCCAGGCTACAAAATCTGACACTGACGAATGTGTCAGTGGACTGGAATATTTTTCTTGAACTTCTTCAGACTATATGGCAATCATCCATTGAATATTTCAATATCAACAATTTTACACAATTGTCGAACATTGAAAAATATAACTTCAGGTATTCGGGTACTTCCATGAAAGCACTGGCACTGAAGAACATTTTAGTCACAGATCTGTACTTTTTTCAGGATGACCTGTACCGGATACTTGCAGACATGAACATTGAAGCCTTGACAGTAGCAGGATCCGAGATGATACATATGCTATGTCCTTCATCTAACAGTCCCTTTAGATACCTAAATTTTATAAACAATGATTTAACCGATCTGCTTTTTCAAAACTGTGACACATTAATTCAACTGGAGATATTTATCTTACATAAGAATAAATTTGAGAGCCTTTCCAAGGTGAGCTCCATGACCAGGTACATGAAATCACGGAGCTATCTGGACATGAGCAGCAACTTGCTGCGTAACGATGGAGCCGAGGAGCGCTGCCAACGGAGCAAGTCTCTGAAGGAGCTGGACCTGTCCTCAAAGCAGCTGACAGAGTCTGTGTTCGGGTGCTTGCCGCTCAATGTCAACAAACTGGACCTACACAACAAGCAGATCAGCAGCGTCCCCCAGGGGATTGCTGCGCTGAAGTCCTTGAAAGAGCTGAAGCTGGCGTTGAACAGGCTGGCCGACCTGCCGGGGTGCAGTGGCTTCTGGGCCCTGGAGATCCTGAATATGGAGATGAACTCGATCCTCAGCCCTTCCGCCGACTTCTTGGAGAGTTGCCAGAGGGTGCGGGAGCTGGAAGCCGGGCACAATCCGTTCAAGTGCTCCTGTGAACTGCAGGCCTTCGTGCGTCTGGAGAGGCAGTCTGGGGGGAAGCTGTCCGGCTGGCCGGAGGCATACGTGTGCGAGTACCCCGAGGACCTGAAGGGAATGCAGCTGAAGGACTTGCACTTGACGGAGCTCGCTTGCAACACGACCCTGTTGCTTGTGACGGCTCTGCTGCTGACGCTGGTGCTGGTGGGGGTGGTGGCCTTTCTGTGCATCTACCTGGATGGGCCGTGGTACGTGCGTGTGCTGTGGCAGTGGACGCAGACGAAGCGCAGAACTTGGCACGAGTGCCCCGAGGAGCGGGAAACCGTCCTGCAGTTCCACGCCTTCATTTCCTACAGCGAGCGCGATTCCGTGTGGGTGAAGACCGAGCTGATCCCGAACCTGGAGAAGGGGGAGGGCAGCATCCGGCTGTGCCAGCACGAGAGAAACTTTGTCCCTGGCAAAAGCATTGTGGAGAATATCATTAACTGCATAGACAAGAGCTACAAGTCAATCTTTGTGTTGTCTCCCAACTTTGTGCAGAGCGAGTGGTGTCACTACGAGCTCTACTTTGCCCATCACAAGTTGTTCAGTGAGAACTGCAACAGCTTGATCCTGATTTTGCTGGAGCCTATTCCTCCGTATATTATCCCTGCGAGGTACCACAAGCTGAAGGCTCTCATGGCAAAGAGAACGTACCTGGAGTGGCCAAAGGAGAGGAGCAAGCGTGCCCTTTTCTGGGCTAACCTGAGGGCAGCTATTAACATTAACCTGCCAGTGGCTGAGGGGCAAAGGTGTGGAGAAAGGGATTAA

>TgutTLR1A Taeniopygia guttata Zebra Finch chr4:48,368,985-48,371,363 (taeGut1) 2418 ATGAGATCCCTCACAAATATATATGTCTTTACTTATGCCTTTACATTCACACTATGGAATAATATCCAGCCAACCGTAGAAGACGAATTTATTGCAAATTATTCAAACAGTTTGCTAACTAATGTTCCAAAAACCACTCCAGTTCATACCCAAGTATTAGATTTATCACATAATAGGATCTCTGGACTTAGTACCTCGGAATTTATTAATCTTTCTGACCTTCAAGTATTAAATCTTTCTCATAATCTAATTACGATGCTTGACTTTAGTGTCTTTATTTTTAATGAAAATTTAGAATACTTAGATTTATCTCATAATAACATTTCAAAAGTTTCCTGTCTAACTCTTGCGTATCTTAGACATTTAGATCTTTCTTTCAATAAGTTTACTGCCCTGCCCATCTGTCAGGAATTTGGGAACATGTTTCATTTGAAGTACCTTGGATTAAGTGCTACTATGATACGAAGGTCAGACTTCAGGTATATCAAACATCTGCAGCTGCACACAGTCTTCCTGAACTTGGGAAACTTTTCACTGTATGAACCTCAGAGTCTGACAGTCTTGTCTACAAGGAACCTCCACATTGCTTTTGCAGCAAACCAAAACTTTGTTTTTTCCCTCTTGTACGATGGAATGAGCACTTCAGAAAACTTAAAAATAGTTAACTTAAGATATACCTTGAGTTACAAAGATTTCCCCTCTCCACCTTTAATGCTTCCGAAGAAAATCAAGACAATAGCTCTCACTCTTGATTCTGTGGACTTACAATGGGCTATCATCCTGCAAATTTTTGTGCTTATTTGGTATTCACCTGTGGAACAATTGACTGTGAGAAATTTGACTTTTAGAGGACCACTGGAGGAGCCAACTGAATATGCATTTCAACCCTTATTAAGCTCTGTGGAACAATTAATCTCTTTGGATGGCTCCATGAAAGCATTAACTTTAGAGCATGTTCGTAATAAGGTTTATTATTTCAACCAGGAGATCCTATACAGACAGTTTTCAGAGATGAATATTGCCAATTTGACAATAGCTGATGCATATATGCCACACATGCTTTGCCCCAATAGAACAAGCTCATTTCAGTATATAAATTTTTCTCACAATGCCCTGACAGATGAGTTGTTCCAGAATTGTGGCACTCTCATGGATCTGAAATTACTTATTTTGAAGAAGAATAAATTTGAGAGCCTTCGCAAAGTAAGCTTCATGACCAGCCGTATGAAATCACTGAAATACCTGGACATGAGCAACAACCTGCTGCGCCACGATGGAGCTGACGTGCAGTGCCAGTGGGCTGAGTCTCTGACAGAGCTGGACCTGTCCTCCAATCAGTTGGCGGATGTTGTGTTTGAGTGCTTGCCAGTCAACATCAAAAAACTCAGCCTACAAAATAATCAGATCAGCAATGTCCCCAGTGGGGTGGCGGAGCTGAAATCCTTGGAAGAGCTGAACCTGGCATCGAACAGGCTGGCCGACCTGCCGGGGTGCAGTGGCTTTACGTCCCTGCAGTTCCTGAACGTAGAGATGAATTCGATCCTCACCCCATCTGCTGACTTCTTCAAGAGCTGCCCAAAGGTCAGGGAGTTGCAGGCCGGGCACAACCCGTTCAAGTGTTCCTGTGAACTGCAAGCCTTTATCCGCCTGGAGAGGCGGTCGGGGGGGAAGCTGTTTGGCTGGCCGGTGGCCTACGTGTGCGAGTACCCCGAAGGCTTGCGAGGAACAGAGCTCAAGGACTTCCACCTGAGCCTGCTGGCCTGCAACACGACGCTCCTGCTTGTGACAGCTCTGCTGCTGACGCTGCTGCTGGTGGCTGTGGTGGCCTTTCTGTGCATCTACCTGGATGTGCCGTGGTACGTGCGGATGACGTGGCAGTGGACGCAGACGAAGCGCAGAGCTCGGCACAACCCTCCTGGGGATCAGGGGGCCGTTCTGCAATTCCACGCGTTCATTTCCTACAGCGAGCGCGATTCGCTGTGGGTGAAGAACGAGCTGATCCCGAACCTGGAGAAGGGGGAGGGCTGCATACAACTGTGCCAGCACGAGAGAAACTTTGTCCCCGGCAAGAGCATTGTGGAGAACATCATTAACTGCATTGAGAAGAGCTACAAGTCGATCTTTGTGTTGTCTCCCAACTTTGTGCAGAGTGAGTGGTGTCACTATGAGCTGTACTTTGCCCATCACAAGTTATTCAGTGAGAATTCCAACAGTTTAATCCTCATTTTACTGGAGCCAATCCCTCCGTACCTTATCCCTGCCAGGTATCACAAGCTGAAAGCTCTCATGGCAAAGCGCACCTACATGGAGTGGCCGAAGGAGAGGAGCAAGCGTGCCCTATTCTGGGCTAATCTCAGGGCAGCCATTAACATTAACCTGCCAAAAGCTGATGAAAACTTGTCTGAGGAAACAGATTAA

>TgutTLR1B Taeniopygia guttata Zebra Finch ENSTGUG00000009041 1962 ATGACACAAAATACAAGCTTTTTAAGAAACGTTTTTCTTTACAAGTGTCTGTTTGCATTAACTTTTTGGAGCTACATCAGCCTGTCTGTAGAAGATGAACTCTTTACATCTGTTTCTAACAATTTTCCAGAAGATGATTCTAACCAAAAAATCAAGAGCCTGCCACTCCTGTATACAAACAGTAATCAGTCCAAAGCTGATTTTGATTGGGTTGTGATACAAAATACTACAGAAAGTCTGTCATTGTCAGAAATCACAAATGACAATGTAAAAAAATTGATAGCTTTATTATCTAATTTCACACAAGGATCCAGGTTACAAAATCTGACACTGACAAATGTGTCAGTGGATTGGAATGCTCTTATGGAAATTTTTCAGACTGTATGGCACTCATCCATTCAATACTTCAATACTAACAATGTAACACAGCTGTCAGACATTGAAAGTTATGACTTTGACTATTCAGGTACTTCTATGAAAGCACTGACAATGAAGAAAATTATAATCACAGACATGTACTTCACACAGGATGACCTATACAAAATATTTGCAGACATGAATATTGAACACATGACAATAGCTGATTCAGAGATGATTCATATGCTTTGTCCTTCATCTAGGAGTCCCTTTAGATACCTAAATTTTTTAAAGAATGATTTAACAGATTTTCTTTTTCAAAACTGTGACAACCTACTTCAGCTGGAGATATTAATCTTGCAGAAGAATAAATTTGAGAGCCTTCGCAAAGTAAGTTTCATGACCAGCCGTATGAAATCACTGAAATACCTGGACATGAGCAACAACCTGCTGCGCCACGATGGAGCTGACGTGCAGTGCCAGTGGGCTGAGTCTCTGACAGAGCTGGACCTGTCCTCCAATCAGTTGGCGGATGTTGTGTTTGAGTGCTTGCCAGTCAACATCAAAAAACTCAGCCTACAAAATAATCAGATCAGCAATGTCCCCAGTGGGGTGGCGGAGCTGAAATCCTTGGAAGAGCTGAACCTGGCATCGAACAGGCTGGCCGACCTGCCGGGGTGCAGTGGCTTTACGTCCCTGCAGTTCCTGAACGTAGAGATGAATTCGATCCTCACCCCATCTGCTGACTTCTTCAAGAGCTGCCCAAAGGTCAGGGAGTTGCAGGCCGGGCACAACCCGTTCAAGTGTTCCTGTGAACTGCAAGCCTTTATCCGCCTGGAGAGGCGGTCGGGGGGGAAGCTGTTTGGCTGGCCGGTGGCCTACGTGTGCGAGTACCCCGAAGGCTTGCGAGGAACAGAGCTCAAGGACTTCCACCTGAGCCTGCTGGCCTGCAACACGACGCTCCTGCTTGTGACAGCTCTGCTGCTGACGCTGCTGCTGGTGGCTGTGGTGGCCTTTCTGTGCATCTACCTGGATGTGCCGTGGTACGTGCGGATGACGTGGCAGTGGACGCAGACGAAGCGCAGAGCTCGGCACAACCCTCCTGGGGATCAGGGGGCCGTTCTGCAATTCCACGCGTTCATTTCCTACAGCGAGCGCGATTCGCTGTGGGTGAAGAACGAGCTGATCCCGAACCTGGAGAAGGGGGAGGGCTGCATACAACTGTGCCAGCACGAGAGAAACTTTGTCCCCGGCAAGAGCATTGTGGAGAACATCATTAACTGCATTGAGAAGAGCTACAAGTCGATCTTTGTGTTGTCTCCCAACTTTGTGCAGAGTGAGTGGTGTCACTATGAGCTGTACTTTGCCCATCACAAGTTATTCAGTGAGAATTCCAACAGTTTAATCCTCATTTTACTGGAGCCAATCCCTCCGTACCTTATCCCTGCCAGGTATCACAAGCTGAAAGCTCTCATGGCAAAGCGCACCTACATGGAGTGGCCGAAGGAGAGGAGCAAGCGTGCCCTATTCTGGGCTAATCTCAGGGCAGCCATTAACATTAACCTGCCAAAAGCTGATGAAAACTTGTCTGAGGAAACAGATTAA

>GfulTLR1 Gyps fulvus Griffon vulture DQ480086 1953 ATGACAGAAAATATGAGATCTCTCAGAAACTTTTTTCTTTACAAGTGTCTGTTTGCATTAACTTTTTGGAATTGTGTCAGCCTGTCTGTGGAAAATGAACTCTTCACATCTGTTTCTAACGAAGATGGTTCTGACAAAAAAATCAAGAGCCTGCCACTCCTCTATACAAATAGTCATCAGTCCAAAGCTAATTTTGACTGGGTTGTGATACAAAATACTACAGAAAGCCTATCGTTGTCAGAAATCACAAATGACAATGTAAAAAAATTAGTAGCATTATTATCTAATTTCAGACAAGGCTCCAGGTTACAAAATCTGACACTGACAAATGTGTCAGTTGACTGGAATGCTCTTATTGAAACTTTTCAGACTGTATGGCACTCACCCATTGAATACTTCAGTGTTAACGGTGTAACACAATTGTCGGACATCGAAAGCTATGACTTTGACTATTCAGGTACGTCTATGAAAGCGGTCACAATGAAGAAAGTTTTAATCACAGATCTGCACTTCTCACAGAATGACCTATACAAAATATTTGCAGACATGAATATTGCAGCCTTGACAATAGCTGAATCAGAGATGATACATATGCTGTGTCCTTCGTCTGACAGTCCCTTTAGATACTTAAATTTTTTAAAGAACGATTTAACAGATCTGCTTTTTCAAAAATGTGACAAATTAATTCAACTGGAGACATTAATCTTGCCGAAGAATAAATTTGAGAGCCTTTCCAAGGTAAGCTTCATGACTAGCCGTATGAAATCACTGAAATACCTGGACATCAGCAGCAACTTGCTGAGTCACGATGGAGCTGATGTGCAATGCCAATGGGCTGAGTCTCTGACAGAGTTGGACCTGTCCTCAAATCAGTTGACGGATGCCGTGTTTGAGTGCTTGCCAGTCAACATCAGAAAACTCAACCTCCAAAACAATCACATCACCAGTGTCCCCAAGGGAATGGCTGAGCTGAAATCCTTGAAAGAGCTGAACCTGGCATCGAACAGGCTGGCTGACCTGCCGGGGTGCAGTGGCTTTACGTCGCTGGAGTTCCTGAACGTAGAGATGAATTCGATCCTCACCCCATCTGCCGACTTCTTCCAGAGCTGCCCACGGGTCAGGGAGCTGCAAGCCGGGCACAACCCGTTCAAGTGTTCCTGTGAACTGCAAGACTTTATCCGTCTGGCGAGGCAGTCTGGGGGGAAGCTGTTTGGCTGGCCAGCGGCGTATGTGTGCGAGTACCCGGAAGACTTGCAAGGAACGCAGCTGAAGGACTTCCACCTGACTGAACTGGCTTGCAACACGGTGCTCTTGCTGGTGACAGCTCTGCTGCTGACGCTGGTGCTGGTGGCTGTCGTGGCCTTTCTGTGCATCTACTTGGATGTGCCGTGGTACGTGCGGATGACGTGGCAGTGGACGCAGACAAAGCGGAGGGCTTGGCACAGCCACCCCAAAGAGCAGGAGACCGTTCTGCAGTTTCACGCGTTCATTTCCTACAGCGAGCGCGATTCGTTGTGGGTGAAGAACGAGCTGATCCCGAACCTGGAGAAGGGGGAGGGCTGTGTACAACTGTGCCAGCACGAGAGGAACTTTATCCCCGGCAAGAGCATTGTGGAGAACATCATTAACTGCATTGAGAAGAGCTACAGGTCGATCTTTGTGTTGTCTCCCAACTTTGTGCAGAGCGAGTGGTGTCACTATGAGCTGTACTTTGCCCATCACAAATTATTCAGTGAGAATTCCAACAGCTTAATCCTCATTTTACTGGAGCCGATCCCTCCGTACATTATCCCTGCCAGGTATCACAAGCTGAAGGCTCTCATGGCAAAGCGAACCTACCTGGAGTGGCCAAAGGAGAGGAGCAAGCGTCCCCTTTTCTGGGCTAATCTGAGGGCAGCTATTAGCATTAACCTGCTAATGGCTGATGGAAAGAGGTGTGGGGAAACAGATTAA

>BtauTLR1 Bos taurus Cow chr6:60,355,759-60,358,137 (bosTau4) 2379 ATGACTAAAAAAAATTCTAGCATCTTCCATTTTGCCATCATCTTTATATTAATACTTGAGATCAGAACTCAATTATCTGATGAAAGTGAATTTTTAATTGACAGGTCAAAAAGAGGTCTCACCTATGTTCCCAAAAACTTATCCCTGGAAACAACCATCTTAGATATATCATACAACTATATTTCTGAGCTTCAGATGCCTGACATCCTCTCACTATCAAAGCTGAAGATTTTGATAATTTCTCATAATAGAATCCAGTATCTTGACTTGAGTGTTTTTAAATTCAACCAGGAACTGGAATACTTGGATTTGTCCCACAACAATTTGGAGAAGATTTCTTGCCACCCTACTCTGAACCTCAAGCACTTAGACCTCTCATTTAATCCATTTGATGCCCTGCCCATATGCCAAGAGTTTGGCAACATGTCTCAACTAGAATTTCTGGGGTTGAGTGCCACACAGTTACAGAAATCCAGTGTGCAGTCAATCACTCATTTGCACATCAGCAAGGTTTTATTGGTCTTAGGAGATACTTATGGGGAAAGAGAAGATGCCGAGAGCCTTCAAGACCTTAAGACACAGAGTCTGCACATTGTTTTCCCCACAGGAAAGGAATTCCATTTTATTTTGGACGTGTCAGTCGGCACCACAGTGAGTCTGGAACTGTCTAATATCAAATGTGTGCTTGATGATAATGGGTGTCCTTATTTCGAAAATGTTCTGTCAAAACTTCAAAAGAACTCAAGGTTATCAAATCTTACTTTAAACAACATTGAAATAACTTGGAATTCCTTCTTCACGATCCTCCAGTTGGTTTGGCGTACAAACATTGAGTACTTCTCCATTTCAAATGTGAAACTACAAGGTTACCTTGACTCTAGAGATTTTGATTATTCTGACACTTCACTGAAGGCCTTGTCTATACACAAAGTTGTCCATGATGTGTTCAGTCTTCCACAAGGTTATGTCTATAAAATATTGTCAAATATGAACATCCAGCATCTCACAGTGTCTGCTGCACACATGGTCCACATGGTCTGCCCATCCCAAATTAGCCCATTTCTGTATTTGAATTTTTCCAATAATCTCTTAACAGACACAGTTTTCATAAACTGTACAAATTTGGCTAATTTGAAGACACTTATCCTACAAAAGAATCAGTTAAAAGAACTTGTAAACATAGTTCATATGACCCAGGAAATGAAGTCTCTACAACAACTGGATGTTAGCCAGAATTCCCTGATGTATGATGAAAGTGAAGGAAATTGCCCTTGGGCCAGAAATTTATTAAGTTTAAATATGTCTTCAAATATACTTACTGACTCTGTTTTCAGATGTTTACCTCCTCAGATCAAGGTTCTTGATCTTCACAATAACAGAATAAGGAGCATCCCTAAAGATGTCACTGGTCTAGAAACTTTGCAAGAACTCAACCTTGCTTCCAATTCTTTAGCCCACCTTCCTGGATGTGGTATCTTTAGCAGCCTTTCCATACTGATCATTGACTATAACTCAATTTCCAATCCATCAGCTGATTTCTTCCAGAGCTGCCAGAAGATTAGGTCCCTCAAAGCGGGGAACAATCCATTCCAATGTTCCTGTGAGCTAAGAGACTTCATCCAAAGTATAGGCCAAGTATCAAGTGACGTGGTAGAGGGCTGGCCTGAGTCTTATAAGTGTGACTATCCGGAAAGCTACAAGGGAACCCCTCTAAAGGACTTCCAGGTATCTGAGCTATCCTGCAACACAGCTCTGCTGATCATCACCATTGTGGTCCCTGGGCTGGTGCTGGCTGTTGCTGTGACTGTCCTCTGTATCTACCTGGATCTGCCCTGGTACCTCAGGATGGTGTGTCAGTGGACCCAGACCCGGCGCAGGGCCAGGAATGTACCCTTGGAAGAACTCCAAAGAACTCTCCAGTTCCATGCTTTTATTTCATATAGTGGGCACGATTCTGCCTGGGTGAAGAATGAATTAATACCTAACCTAGAAAAAGAAGATATAAGAATTTGTCTCCATGAGAGAAACTTTGTTGCTGGCAAGAGCATTGTGGAAAATATCATCAACTGCATTGAGAAAAGTTACAAATCCATCTTCGTTTTGTCTCCCAACTTTGTCCAGAGCGAATGGTGCCATTATGAACTCTACTTTGCCCACCACAATCTCTTCCATGAAGGATCTGATAACTTAATCCTGATCTTGCTGGATCCCATTCCACAGTATTCCATTCCTAGCAGCTACCACAAGCTAAGAGCTCTCATGGCACAGAGAACTTATTTGGAATGGCCCAAGGAGAAGAGTAAACACGGACTTTTTTGGGCTAACCTAAGAGCATCCATTAATATTAAACTGATGGAAAAAGCAGCAGAAATACATTAA

>PtroTLR1 Pan troglodytes Chimpanzee XM_001139029 2361 ATGCCTAGCATCTTCCATTTTGCCATTATCTTCATGTTAATACTTCAGATCAGAATACAATTATCTGAAGAAAGTGAATTTTTAGTTGATAGGTCAAAAAACGGTCTCATCCACGTTCCTAAAGACCTGTCCCAGAAAACAACAATCTTAAATATATCGCAAAATTATATATCTGAGCTTTGGACTTCTGACATCTTATCACTGTCAAAACTGAGGATTTTGATAATTTCTCATAATAGAATCCAGTATCTTGATATCAGTGTTTTCAAATTCAACCACGAATTGGAATACTTGGATTTGTCCCACAACAAGTTGGTGAAGATTTCTTGCCACCCTACTGTGAACCTCAAGCACTTGGACCTGTCATTTAATGCATTTGATGCCCTGCCTATATGCAAAGAGTTTGGCAATATGTCTCAACTAAAATTTCTGGGGTTGAGCACCACACACTTAGAAAAATCTAGTGTGCTGCCAATTGCTCATTTGAATATCAGCAAGATCTTGCTGGTCTTAGGAGAGACTTATGGGGAAAAAGAAGACCCTGAGGGCCTTCAAGACTTTAACACTGAGAGTCTGCACATTGTGTTCCCCACAAACAAAGAATTCCATTTTATTTTGGATGTGTCAGTCAAGACTGTAGCAAATCTGGAACTATCTAATATCAAATGTGTGCTAGAAGATAACAAATGTTCTTACTTCCTAAGTATTCTGGCGAAACTTCAAACAAATCCAAAGTTATCAAGTCTTACCTTAAACAACATTGAAACAACTTGGAATTCTTTCATTAGGATCCTCCAGCTGGTTTGGCATACAACCGTATGGTATTTCTCAATTTCAAACGTGAAGCTACAGGGTCAGCTGGACTTCAGAGATTTTGATTATTCTGGCACTTCCTTGAAGGCCTTGTCTATACACCAAGTTGTCAGCGATGTGTTCAGTTTTCCGCAAAGTGATATCTATGAAATCTTTTCGAATATGAACATCAAAAATTTCACAGTGTCTGGTACACGCATGGTCCACATGCTTTGCCCATCCAAAATTAGCCCGTTCCTGCATTTGGATTTTTCCAATAATCTCTTAACAGACACGGTTTTTGAAAATTGTGGGCACCTTACTGAGTTGGAGACACTTATTTTACAAATGAATCAATTAAAAGAACTTTCAAAAATAGCTGAAATGACTACACAGATGAAGTCTCTGCAACAATTGGATATTAGCCAGAATTCTGTAAGCTATGATGAAAAGAAAGGAGACTGTTCTTGGACTAAAAGTTTATTAAGTTTAAATATGTCTTCAAATATACTTACTGACACTATTTTCAGATGTTTACCTCCCAGGATCAAGGTACTTGATCTTCACAGCAATAAAATAAAGAGCGTTCCTAAACAAGTCGTAAAACTGGAAGCTTTGCAAGAACTCAATGTTGCTTTCAATTCTTTAACTGACCTTCCTGGATGTGGCAGCTTTAGCAGCCTTTCTGTATTGATCATTGATCACAATTCAGTTTCCCACCCATCGGCTGATTTCTTCCAGAGCTGCCAGAAGATGAGGTCAATAAAAGCAGGGGACAATCCATTCCAATGTACCTGTGAGCTAAGAGAATTTGTCAAAAATATAGACCAAGTATCAAGTGAAGTGTTAGAGGGCTGGCCTGATTCTTATAAGTGTGACTACCCAGAAAGTTATAGAGGAAGCCCACTAAAGGACTTTCACATGTCTGAATTATCCTGCAACATAACTCTGCTGATCGTCACCATCGTTGCCACCATGCTGGTGTTGGCTGTGACTGTGACCTCCCTCTGCATCTACTTGGATCTGCCCTGGTATCTCAGGATGGTGTGCCAGTGGACCCAGACCCGGCGCAGGGCCAGGAACATACCCTTAGAAGAACTCCAAAGAAATCTCCAGTTTCATGCATTTATTTCATATAGTGGGCACGATTCTTTCTGGGTGAAGAATGAATTATTACCAAACCTAGAGAAAGAAGGTATGCAGATTTGCCTTCATGAGAGAAACTTTGTTCCTGGCAAGAGCATTGTGGAAAATATCATCACCTGCATTGAGAAGAGTTACAAGTCCATCTTTGTTTTGTCTCCCAACTTTGTCCAGAGTGAATGGTGCCATTATGAACTCTACTTTGCCCATCACAATCTCTTTCATGAAGGATCTAATAACTTAATCCTGATCTTGCTGGAACCCATTCCACAGTACTCCATTCCTAGCAGTTATCACAAGCTCAAAAGTCTCATGGCCAGGAGGACTTATTTGGAATGGCCCAAGGAAAAGAGCAAACGTGGCCTTTTTTGGGCTAACTTAAAGGCAGCCATTAATATTAAGCTGACAGAGCAAGCAAAGAAATAG

>CfamTLR1 Canis familiaris Dog EU551145 2370 ATGAAAACTAATCCTAGCATCTTCCAATTTGCCATCATCTTCATATTAATACTTGAGATCAGAATACAATTGTCTGAAGAAAGTGATTTTCTAGTTAACAGATCAAAAGCAGGTCTCTTTCACATTCCCAAAGACCTATCCCTGAAAACAACAATCTTAGATATATCACAAAACTATATATCTGAGCTTCAGACTTCTGACATCCTATCACTATCAAAGCTGAGGATTTTGATTGTTTCTTATAATAGAATTCAATATCTTGATATCAGTGTTTTCAAATTCAACCAGGAATTGGAATACTTGGATCTGTCCCACAATGAGTTGGGGAGGATTTCTTGCCATCCTACCGTGAACCTCAAGCACTTAGACCTTTCATTTAATGCATTTGATGATCTACCCATATGCAAAGAGTTTGGCAACATGTCTCAACTAGAGTTTCTGGGGTTGAGTGCCACACAGTTACAGAAATCTAGCATGCTACCAATTGCTTCTTTGCATATCAGAAAGGTTTTACTGGTCTTAGGAGACACTTATGGGAAAAAAGAAGACCCTGAGAGCCTTCAAAAGCTTAACACAGAAAGTCTTCACATTGTTTTCCCTATAAGAAAGGAATTCAGTTTTACTCTGGATGTATCAGTCAGCACTGCAGTAAGTCTCGAATTGTCTAATATCAAATGTGTGCCAGATGGTCATGGATGGTCTTATTTCCAAAATGTTCTGTCAAAACTTCAAAAGAATTCAAGGTTATCAAGTCTTACTTTAAACAACATTGAAACAACTTGGAATTTTTTCATTATGCTCCTTCAGTTGGTTTGGCATACAAGCATAGAGTATTTCTCAATTTCAAATGTAAAACTACAAGGTTACCCTGACTTCAGAGATTTTGATTATTCTGACACTTCACTGAAGGCCTTATCTATACACCAAGTCGTTAGTAATGCATTCAATTTGCCACAAAGTTATATCTATAAAATCTTTTCAAATATGAACATCCAAAATTTCACAGTGTCTGGTACGCACATGGTCCACATGGTTTGCCCATCTCAAATTAGTCCATTTCTGCATTTGGATTTTTCTAATAATCTCTTAACAGACATTGTTTTTAAAAATTGTAGAAACTTGATTAAACTGGAGACACTTAGTTTACAAATGAATCAATTAAAAGAACTTGCAAGTATAGCTCAAATGACCAACGAGATGAAGTCTCTACAACAATTGGATATTAGCCAGAATTCTCTAAGGTATGATGAAAATGAAGGAAACTGCTCTTGGACTAGAAGTTTATTAAGTTTAAATATGTCTTCAAATATACTTACTGACTCTGTTTTCAGATGTTTACCTCCCAAGGTCAAGGTGCTTGATCTTCACGATAACAGAATAAGGAGCATTCCTAAACCAATCATGAAGCTAGAAGATTTGCAAGAACTCAATGTTGCTTCCAATTCTTTAGCCCACTTTCCTGACTGTGGTACTTTTAATAGGCTTTCTGTACTGATCATTGACTCTAATTCAATTTCCAATCCATCAGCTGATTTCCTCCAGAGCTGCCATAACATTAGGTCCATAAGCGCAGGGAATAATCCATTCCAGTGTACATGTGAGCTGAGAGAATTTGTCCAAAGTCTAGGCCAGGTAGCAAGCAAAGTAGTAGAGGGTTGGCCTGATTCTTATAAGTGTGACTCTCCAGAAAACTATAAGGGAACCCTACTGAAGGACTTTCACGTGTCTCCGTTATCCTGCAACACAACTCTGCTGCTTGTCACCATTGGGGTCGCTGTGCTAGTGTTCACTGTTACTGTGACTGCGCTCTGTATCTACTTTGATCTGCCCTGGTATCTTAGGATGGTGTTTCAGTGGACCCAGACCCGGCGCAGGGCAAGAAACACACCCTTAGAAAATCTCCAAAGAACCATCCAGTTCCATGCTTTTATTTCATATAGCGGGCATGATTCTGCCTGGGTGAAGAGTGAATTACTACCAAACCTAGAAAAAGAAGAACTAAGGATTTGTCTCCATGAGAGAAACTTTATTCCTGGCAAGAGCATTGTGGAAAATATCATAAACTGCATTGAGAAAAGTTACAAGTCCATCTTTGTTCTGTCTCCCAACTTTGTTCAGAGTGAGTGGTGCCATTATGAACTGTACTTTGCCCACCACAATCTCTTTCATGAAGGATCTAATAACTTAATCTTGATCTTGCTGGAACCTATTCCACAGTATTCCATTCCTAGCAGCTATCACAAGCTCAAAAATCTCATGGCACAAAGGACTTATTTGGAATGGCCCAAGGAGAAGAGCAAACATGGACTTTTTTGGGCTAACCTAAGAGCGTCTATTAATATTAAATTGAGGGAGCAAGCAAAAAAATAG

>EeurTLR1 Erinaceus europaeus Hedgehog ENSEEUG00000015862 2373 ATGACTAAAACTTACTCTATTGTCTTCCATTTAATCATCATCTTCATGCTAATAGTTAAGATCAGAACTCTACTATCTGATGGAAGTGATGTTTTAGCTGACAGATCAAACAGGACTCTCATTCATATTCCCAAGGATCTACCCCCCAGTACGACAATCCTAAATGTTTCACACAACTATATATCTGAGCTTTGGGCTTCTGACATCCTGTCACTCTCAAAGCTGAAGATCTTGATAATGTCTCATAACAGAATCCAGAATCTTGATATTAGTGTTTTCAGATTCAACCAGGAATTGGAATACTTGGATTTATCTCACAACAAATTGGAGACAATTTCTTGCCACTCCACTGCGAACCTTAAACATTTGGATCTCTCCTTCAATGCATTTGTTTTCCTACCCATATGCAAAGAGTTTGGTAACATGTCCCAGCTAGAATTTCTAGGGTTGAGTGCAAGCCAGTTACAAAAGTCTAGGTTGCTGTCAATTTCCCATCTGCACATCAGTAAGGTTTTACTGGTCTTAGGAGACTCCTATGGGGAAAAAGAAATCCCTGACAGCCTTCAAGACCTGAACACAGAAAGTCTTCACATTGTTTTTCCCCTGGGGAAGGAGTTCCATTTTAATTTGGATGTGTCAATCAGTCAGGCAGTAAGTCTGGAACTGTCTAATATCCAGTATGTGCTGGAAGATGGTGGTGCATGTTCTTTCCAAAATGCTCTGAGGAAACTTCAGAAGAATCCAAGGCTATCAAATCTTACTTTAAATAACATTGACACCACTTGGAATTCCTTCATGATGATTCTCCAGTTGGTTTGGCACACAGGCGTAGAATATTTCTCAATTAAAAATGTGAAACTACAAGGTTGGTTTCATCCCAGAGAGTTTAATTATTCTGACACTTCACTGAAATCCTTGACTATACATCAGGTTGTCAATAATGCATACAGTTTAGAACAAAATTCTATCTACAAAATCTTTGCAAATATGAACATCCAACATTTCACAGTGTCTGGTACACCTATGGTCCACATGCTTTGCCCATTACAAACTAGCCCATTTCTGTATTTGGATTTTTCTAATAATCTCTTGACAGACATGATTTTCAAAGATTGTGGAAATTTGACCAAATTAGAGACTCTTATTTTACAAATGAATCAACTACAAGAATTTACAAAGATAGTTTACATGACCAAGAAGATGAAGTCTCTACAACTACTGGATATTAGCCAGAATTCTCTAAGGATTGATGAAAATGAAGGAAATTGTTCTTGGACAGAAAGTTTATCAAGTTTAAATTTATCTTCAAATATACTTACTGAGTCTGTTTTCAGATGTCTACCTCCCAGGGTCAAGGTCCTTGATCTTCACAGTAACAGAATAAGGAGCATCCCAAGAGATGTCAACAATCTGGAAGCTTTGCAAGTACTCAATGTTGCTTCCAATTTTTTAACCAACCTTCCTGGATGTGGTGCCTTTAGCAGCCTTTCTGCACTGATCATTGACTATAACTCAATTTCCAGTCCATCAGTTGATTTCTTCCAGAGCTGCCAGAACATTAGGTCAGTAAAAGCAGGGAACAACCCATTCCAGTGTACATGTGAGCTCAGAGAGTTTGTCCAGAGAATGGGCCAAGTGTCAAGGGAAGTGGTAGAGGACTGGCCTGGTTCTTACCAGTGTGACTATCCAGAAAGCTTTAAGGGAACTGCACTAAAGGACTTCCACATGTCTCAGCTGTCCTGCAACACCACTCTGTTGATTGTCACCATTGTGGTCATTGTGCTGGTGTTGGGTAGTACCACGGTCATGCTCTGTATCTACTTTGATGTGCTCTGGTATCTGAGGATGATGTGCCATTGGACCCAGACCCGGCAAAGGGCTAGGAACACTCCCTTAGCTGAACTCCAGAGAAACCTCCAGTTCCATGCTTTCATTTCATATAGTGAACATGATTCCGCCTGGGTGAAGAGTGAATTACTACCAAACTTAGAAAAAGAAAATATACGAATTTGTCTTCATGAGAGAAACTTTGTCCCTGGGAAGAGCATCATAGAAAACATTATCAACTGCATTGAGAAAAGTTACAAGTCCATCTTTATTCTGTCTCCCAACTTTGTACAGAGTGAGTGGTGCCATTATGAGCTCTACTTTGCCCACCACAATCTCTTTCATAAAGGGTCTGATAACTTAATCTTGATCTTGCTAGAACCCATTCCACAGTATTCCATTCCTAACAGTTATCACAAGCTTAAAGCTCTCATGGCAAGAAGGACTTATTTGGAATGGCCCAAGGAGAAGAGAAAACATGGACTTTTTTGGGCTAGCTTAAGAGTATCCATTAATATTAAATTGACAGAGCAAGCAAAGAAGTAT

>EcabTLR1 Equus caballus Horse XM_001498644 2361 ATGACTAAAACTAATTCTGGCATCTTCCACTTTGCCATCATCTTTGTGTTAATACTTGAGATTGGAATCCAATCATCTGATGAAAGGGAATTTTTTGTTGACAGATCTAAAGCAGGTCTCACCCATGTTCCCAAAGATCTATCTCTGAAAACAACAATCTTAGATATATCACAAAACTATATATCTGAGCTTCGGACTTCTGACATCCAATTACTATCAAAGCTGAGGATTTTGATAATTTCTCATAATAAAATCCAGTATCTTGATATCAGTGTTTTCAAATTCAACCTGGAATTGGAATACTTGGATTTGTCCCACAACAAGTTGGGGAAGATTTCTTGCCACCCTACTGTGAACCTCAAGCACTTAGACCTCTCATTTAATGCATTTGATGCCCTGCCCATATGCAAAGAGTTTGGCAACATGTCTCAACTAGAATTTCTGGGGTTGAGTGCCACACAGTTACAAAAATCTAGTGTGCTGCCAATTGCTCATTTGCATATCAGTAAGGTTTTACTGGTCTTAGGAGACCCTTATGGGGAAAAAGAAGACTCTGAGAGCCTTCGACACCTTAACACAGAGAGTCTGCACATTGTTTTCCCGACAAGAAAGGAATTCCATTTCATTTTGGATGTGTCCCTCAGCACTGCAGTAAATCTGGAACTGTCTAATATCAAATGTGTGCTGGATGATAACCGATGTTCTTATTTCCTAAATGTTCTGTCAAAACTTCAAAAGAATCCAAGGTTATCAAGTCTTACTTTAAACAACATTGAAACAGCTTGGGATTCTTTTATTATGATCCTCCAGTTGGTTTGGCATACAAGTGTAGAAAATTTCTCGATTAAAAATGTGAAACTACAAGGTCACCTTGGCTTCAGAGATTTTGATTATTCTAACACTTCACTGAAGGCCTTGTCTATATACCAAGTTGTCAGTGATGTGTTCAGTTTTCCACAAAGTTCTATCTACAAAATCTTTTCAAATATGACCATCCAAAATTTCACAGTATCTGGTACACACATGATCCACATGCTTTGCCCATCTCAAATTAGCCCATTTCTGCATTTGGATTTTTCCAATAATCTCTTAACAGACACGATTTTTAGAGATTGTGGAACATTGACTAAGCTGGAGACATTTAGTTTACAAATAAATCAATTAAAAGAACTTACAAATATAGCTCATATGACCAAGGAGATGAAGTCTCTACATCAATTGGATATTAGCCAGAATTTTTTAAGGTATGATGAAAATGAAGGAAATTGCTCTTGGACTAGAAGTTTATTAAGTTTAAATATGTCTTCAAATATACTTACTGACTCTGTTTTCAGATGTTTACCTCCCAGGATCAAGGTACTTGATCTTCACAATAACAGAATAAGGAGCATCCCTAAACAAATCATGAAACTAGAATCTTTGCAAAAACTCAATGTTGCTCTCAATTCTTTAACCAACCTTCCTGGATGTGGTGCCTTTAACAGCCTTTCTACACTGATCATTGACCATAATTCAATTTCCAACCCATCAGTTGACTTCTTCCAGAGCTGCCAGAAGATTAGGTCAATAAAAGCAGGGAACAATCCATTCCAATGTACGTGTGAGCTAAGAGAATTTATCCAAAGTATAGGCCAAGTATCAAGTGATGTGGTAGAGGGTTGGCCTGATTCTTATAAGTGTGAGTATCCAGAAAGCTATAAGGGAACCCCACTAAAGGACTTTCATCTGTCTCAATTATCCTGCAACACAGCTCTGCTTGTTGTCACCATTGTGGTCCCTGTGCTGGTGTTGGCTGTTACTGTGAGCATCCTCTGTATCTACCTGGATCTGCCCTGGTATCTCAGGATGGTGTGTCAGTGGACCCAGACCCGGCGCAGGGCTAGGAACATACCCTTAGAAGAACTCCAAAGAACTCTCCAGTTCCATGCTTTTATTTCATATAGTGGGCACGATTCTGCCTGGGTGAAGAGTGAATTACTACCAAACTTAGAAAAGGAGGATATACGGATTTGTCTCCATGAGAGAAACTTTATTGCTGGCAAGAGCATTGTGGAAAATATCATAAACTGCATTGAGAAAAGTTACAAGTCCATCTTTGTTTTGTCTCCCAACTTTGTTCAGAGTGAGTGGTGCCATTATGAGCTCTACTTTGCCCACCACAATCTCTTTCATGAAGCTTTTAATAACTTAATCCTGATCTTGCTGGAACCCATTCCACAATATTCCATTCCCAGTAGCTATCACAAGCTGAAAATTCTCATGGCAAAGAGGACTTATTTGGAATGGCCCAAGGAGAAGAGCAAACATGGACTTTTTTTGGCTAACCTAAGAGCAGCCATTAATATTAAGTTGATGGAGTAA

>HsapTLR1 Homo sapiens Human BC089403 2361 ATGACTAGCATCTTCCATTTTGCCATTATCTTCATGTTAATACTTCAGATCAGAATACAATTATCTGAAGAAAGTGAATTTTTAGTTGATAGGTCAAAAAACGGTCTCATCCACGTTCCTAAAGACCTATCCCAGAAAACAACAATCTTAAATATATCGCAAAATTATATATCTGAGCTTTGGACTTCTGACATCTTATCACTGTCAAAACTGAGGATTTTGATAATTTCTCATAATAGAATCCAGTATCTTGATATCAGTGTTTTCAAATTCAACCAGGAATTGGAATACTTGGATTTGTCCCACAACAAGTTGGTGAAGATTTCTTGCCACCCTACTGTGAACCTCAAGCACTTGGACCTGTCATTTAATGCATTTGATGCCCTGCCTATATGCAAAGAGTTTGGCAATATGTCTCAACTAAAATTTCTGGGGTTGAGCACCACACACTTAGAAAAATCTAGTGTGCTGCCAATTGCTCATTTGAATATCAGCAAGGTCTTGCTGGTCTTAGGAGAGACTTATGGGGAAAAAGAAGACCCTGAGGGCCTTCAAGACTTTAACACTGAGAGTCTGCACATTGTGTTCCCCACAAACAAAGAATTCCATTTTATTTTGGATGTGTCAGTCAAGACTGTAGCAAATCTGGAACTATCTAATATCAAATGTGTGCTAGAAGATAACAAATGTTCTTACTTCCTAAGTATTCTGGCGAAACTTCAAACAAATCCAAAGTTATCAAGTCTTACCTTAAACAACATTGAAACAACTTGGAATTCTTTCATTAGGATCCTCCAGCTGGTTTGGCATACAACTGTATGGTATTTCTCAATTTCAAACGTGAAGCTACAGGGTCAGCTGGACTTCAGAGATTTTGATTATTCTGGCACTTCCTTGAAGGCCTTGTCTATACACCAAGTTGTCAGCGATGTGTTCGGTTTTCCGCAAAGTTATATCTATGAAATCTTTTCGAATATGAACATCAAAAATTTCACAGTGTCTGGTACACGCATGGTCCACATGCTTTGCCCATCCAAAATTAGCCCGTTCCTGAATTTGGATTTTTCCAATAATCTCTTAACAGACACGGTTTTTGAAAATTGTGGGCACCTTACTGAGTTGGAGACACTTATTTTACAAATGAATCAATTAAAAGAACTTTCAAAAATAGCTGAAATGACTACACAGATGAAGTCTCTGCAACAATTGGATATTAGCCAGAATTCTGTAAGCTATGATGAAAAGAAAGGAGACTGTTCTTGGACTAAAAGTTTATTAAGTTTAAATATGTCTTCAAATATACTTACTGACACTATTTTCAGATGTTTACCTCCCAGGATCAAGGTACTTGATCTTCACAGCAATAAAATAAAGAGCATTCCTAAACAAGTCGTAAAACTGGAAGCTTTGCAAGAACTCAATGTTGCTTTCAATTCTTTAACTGACCTTCCTGGATGTGGCAGCTTTAGCAGCCTTTCTGTATTGATCATTGATCACAATTCAGTTTCCCACCCATCAGCTGATTTCTTCCAGAGCTGCCAGAAGATGAGGTCAATAAAAGCAGGGGACAATCCATTCCAATGTACCTGTGAGCTAGGAGAATTTGTCAAAAATATAGACCAAGTATCAAGTGAAGTGTTAGAGGGCTGGCCTGATTCTTATAAGTGTGACTACCCGGAAAGTTATAGAGGAACCCTACTAAAGGACTTTCACATGTCTGAATTATCCTGCAACATAACTCTGCTGATCGTCACCATCGTTGCCACCATGCTGGTGTTGGCTGTGACTGTGACCTCCCTCTGCATCTACTTGGATCTGCCCTGGTATCTCAGGATGGTGTGCCAGTGGACCCAGACCCGGCGCAGGGCCAGGAACATACCCTTAGAAGAACTCCAAAGAAATCTCCAGTTTCATGCATTTATTTCATATAGTGGGCACGATTCTTTCTGGGTGAAGAATGAATTATTGCCAAACCTAGAGAAAGAAGGTATGCAGATTTGCCTTCATGAGAGAAACTTTGTTCCTGGCAAGAGCATTGTGGAAAATATCATCACCTGCATTGAGAAGAGTTACAAGTCCATCTTTGTTTTGTCTCCCAACTTTGTCCAGAGTGAATGGTGCCATTATGAACTCTACTTTGCCCATCACAATCTCTTTCATGAAGGATCTAATAGCTTAATCCTGATCTTGCTGGAACCCATTCCGCAGTACTCCATTCCTAGCAGTTATCACAAGCTCAAAAGTCTCATGGCCAGGAGGACTTATTTGGAATGGCCCAAGGAAAAGAGCAAACGTGGCCTTTTTTGGGCTAACTTAAGGGCAGCCATTAATATTAAGCTGACAGAGCAAGCAAAGAAATAG

>CjacTLR1 Callithrix Jacchus Marmoset ENSCJAG00000000831 2361 ATGACTAGCATCTTCCATTTTGCCATTATCTTCATGTTAATACTTCAGATCAGAACACAATTATCTGAAGAAAGTGAATTTTTAGTTGATAGGTCAAATAACGGTCTCATCCACATTCCTAAAGACTTATCCCAGAAAACAACAGTCTTAAATATATCACAAAATTATATATCTGAGCTACAGACTTCTGACATCTTATTACTGTCGAAGCTGAGGGTTTTGATAATTTCTCATAATAGAATCCAGTATCTTGATATCAGTGTTTTCAAATTCAACCAGGAATTGGAATACTTGGATTTGTCCCACAACAAGTTGGTTGAGATTTCTTGCCACCCTACTGTGAACCTCAGGCACCTGGACCTGTCATTTAATGCATTTGATGCCCTGCCTATATGCAAAGAGTTTGGCAGTATGTCTCAACTAAAATTTCTGGGGTTGAGTGCTACACACTTAGAAAAGACTAGCGTGCTGCCAATTGCTCACTTGAATATCAGTAAGGTCTTGCTGGTCTTAGGAGAGCCTTATGGGGACAAAGAAGACCCCGAGGGCCTTCAAGACTTTAACACTGAGAGCCTGCACATTGTTTTCCCCGCAAATAAAGAATTCCATTTTATTTTGGATGTGTCAGTCAGGACTGTAGCAAATCTAGAACTATCTAATATCAAGTGTGTGCTGGAAGATAACGAATGTTCTTACTTCCTAAATATTCTGGCAAAACTTCAAACAAATCCAAAGTTGTCAAGTCTTACTTTAAACAACATTGAAACAACTTGGAATTCTTTCATTAGGATCTTCCAGCTGGTTTGGCATGCAGCCATACAGTATTTCTCAATTTCAAACGTGAAACTACAGGGTCAGTTGGATTTCAGAAATTTTAATTATTCTGGCACTTCCCTGAAGGCCTTGTCAATACACCAAGTTGTCACTGATGTGTTCAGTTTTCCACAAAGTGATATCTACAGCATCTTTTCAAATATGAACATCAAAAATTTAACAGTGTCTGGTACACACATGGTCCACATGCTTTGCCCATTCAAAAGTAGCCCGTTCCTGTATTTGGATTTTTCCAATAATCTCTTAACAGACACGGTTTTTGAAAATTGTGGTCACCTTACTGAGTTGGAGACACTTATTTTACAAACGAATCAATTAAAAGAACTTTCAAAAATAGCTCTTATGACTAAACAGATGAAGTCTCTGCAACAATTGGATATTAGCCAGAATTCTCTAAGCTATGATGAAAATGAAGAAGATTGCTCTTGGACTAAAAGTTTATTAAGTTTAAATATGTCTTCAAATATACTTACTGACTCCGTTTTCAGATGTTTACCTCCCAGGATCAAGGTACTTGATCTTCACAGCAATAAAATAAAGAGCATTCCTAAACAAGTCATAGAACTGGAAGCTTTGCAGGAACTCAATGTTGCTTTCAATTCTTTAACGGACCTTCCTGGATGTGGCAGCTTTAGCAGGCTTTCTGTGTTGATCATTGATCACAATTCAGTTTCCCACCCATCAGCTGATTTCTTCCAGAGCTGCCAGAAGATGAGGTCAATAAATGCAGGGAACAATCCATTCCACTGTACCTGTGATCTAAGAGAATTTGTCAAAAATATAGGCCAGGTATCAAGTGAAGTAGTAGAGGGCTGGCCCGATTCTTATAAGTGTGACTACCCAGATAGTTATAGAGGAACCCCACTGAAGGACTTTCACCTGTCTGACTTATCCTGCAACATAACTCTGCTGATCGTCACCATTGGAGCCACCATGCTGGTGTTGGCTGTGACTGTGACCTCCCTCTGCATCTACTTGGATCTGCCCTGGTATCTCAGGATGGTGTGCCAGTGGACCCAGACCCGACGCAGGGCCAGGAATGTACCCTTAGAAGAACTCCAAAGAAATCTCCAGTTTCATGCTTTTATTTCATATAGTGGGCACGATTCTTTCTGGGTGAAGACTGAATTATTACCAAACCTAGAGAAAGAAGGTATGCAGATTTGCCTTCATGAGAGAAACTTTGTTCCTGGCAAGAGCATTGTGGAAAATATCATCAACTGCATTGAGAAGAGTTACAAGTCCATCTTTGTCTTGTCTCCCAACTTTGTCCAGAGTGAGTGGTGCCATTATGAACTCTACTTTGCCCACCACAATCTCTTCCATGAAGGATCTAATAACTTAATCCTCATCTTGCTGGAACCCATTCCGCAGTACACCATTCCTACCAGCTATCACAAGCTCAAATCGCTCATGGCCAGGAGGACTTATTTGGAATGGCCCAAGGAAAAGAGCAAACATGGGCTTTTCTGGGCTAACCTAAGGGCAGCTATTAATATTAAGCTGACAGAGCAAGCAAAAAAGTAG

>MmusTLR1 Mus musculus Mouse AY009154 2388 ATGACTAAACCAAATTCCCTCATCTTCTACTGTATCATTGTTTTAGGACTGACACTTATGAAAATCCAATTATCTGAGGAATGTGAGCTTATCATAAAGAGGCCAAACGCAAACCTTACCAGAGTGCCCAAGGACCTACCCTTGCAAACAACTACTTTAGATCTATCACAAAACAATATATCTGAGCTTCAGACTTCTGACATCCTCTCATTGTCCAAGCTGAGGGTCCTGATAATGTCCTACAACAGACTCCAGTATCTTAATATCAGTGTTTTCAAATTCAACACAGAGCTGGAATATTTGGATTTGTCCCACAATGAGCTAAAGGTGATCTTGTGCCACCCAACAGTCAGCCTCAAGCATTTGGACCTCTCCTTTAATGCCTTTGATGCCCTGCCTATATGCAAAGAATTTGGCAACATGTCCCAACTACAGTTCCTGGGGTTGAGCGGTTCTCGGGTACAAAGTTCAAGTGTGCAGCTGATTGCTCATTTGAACATCAGTAAGGTTTTGCTGGTGTTAGGAGATGCTTATGGGGAAAAAGAAGACCCCGAATCTCTTCGGCACGTTAGCACTGAGACTCTGCATATTGTTTTCCCGTCGAAAAGAGAATTCCGTTTTCTTCTGGATGTGTCCGTCAGCACTACGATCGGTTTGGAACTGTCTAACATCAAGTGTGTGCTTGAAGACCAGGGCTGCTCTTATTTCTTACGTGCTTTGTCAAAGCTTGGAAAGAATCTGAAGCTCTCAAATCTTACCCTGAACAATGTGGAAACAACGTGGAATTCCTTCATTAATATCCTCCAGATAGTTTGGCATACGCCAGTCAAATATTTCTCAATTTCAAATGTGAAGCTACAAGGTCAACTTGCCTTCAGGATGTTCAATTATTCTGACACTTCTCTGAAGGCTTTGTCGATACATCAAGTTGTCACTGATGTCTTCAGCTTCCCCCAAAGTTACATATACAGTATCTTTGCCAATATGAACATCCAAAACTTTACAATGTCTGGAACACACATGGTCCACATGCTGTGCCCGTCCCAAGTTAGCCCATTTCTGCATGTGGACTTTACAGATAACCTTTTAACAGACATGGTTTTTAAAGACTGTAGAAACTTAGTTAGATTGAAAACACTTAGTTTACAAAAGAATCAGTTAAAAAACCTTGAGAATATAATCCTCACATCTGCAAAGATGACATCCCTACAAAAACTAGACATTAGCCAGAATTCTCTAAGGTACAGCGATGGGGGAATCCCATGCGCCTGGACCCAGAGTTTGTTAGTTTTAAATTTGTCTTCGAATATGCTTACAGGCTCTGTCTTCAGATGCTTACCTCCCAAAGTCAAGGTCCTTGACCTTCACAACAACAGGATAATGAGCATCCCTAAAGATGTCACCCACCTGCAGGCTTTGCAGGAACTCAATGTAGCATCCAACTCCTTAACTGACCTTCCTGGGTGCGGGGCCTTCAGCAGCCTTTCTGTGCTGGTCATCGACCATAACTCAGTTTCCCATCCCTCTGAGGATTTCTTCCAGAGCTGTCAGAATATTAGATCCCTAACAGCGGGAAACAACCCATTCCAATGCACATGTGAGCTGAGGGACTTTGTCAAGAACATAGGCTGGGTAGCAAGAGAAGTGGTGGAGGGCTGGCCTGACTCTTACAGGTGTGACTACCCAGAAAGCTCTAGGGGAACTGCACTGAGGGACTTCCACATGTCTCCACTATCCTGTGATACTGTTCTGCTGACTGTCACCATCGGGGCCACTATGCTGGTGCTGGCTGTCACTGGGGCTTTCCTCTGTCTCTACTTTGACCTGCCCTGGTATGTGAGGATGCTGTGTCAGTGGACACAGACCAGGCACAGGGCCAGGCACATCCCCTTAGAGGAACTCCAGAGAAACCTCCAGTTCCATGCTTTTGTCTCATACAGTGGGCATGATTCTGCCTGGGTGAAGAACGAATTACTACCCAACCTAGAGAAAGATGACATCCAGATTTGCCTCCATGAGAGGAACTTTGTCCCTGGCAAGAGCATTGTGGAGAACATCATCAATTTCATTGAGAAGAGTTACAAGTCCATCTTTGTGCTGTCTCCCCACTTCATCCAGAGTGAGTGGTGTCATTATGAACTCTATTTTGCCCATCACAATCTCTTCCATGAAGGCTCTGATAACTTAATCCTCATCTTGCTGGCACCCATTCCCCAGTACTCCATCCCTACCAATTACCACAAGCTCAAAACTCTCATGTCACGAAGGACCTATCTGGAATGGCCCACAGAGAAGAACAAGCATGGACTTTTTTGGGCAAACCTAAGAGCATCCATTAATGTTAAGCTGGTTAACCAGGCAGAAGGAACGTGTTACACACAGCAATAA

>PpygTLR1 Pongo pygmaeus Orangutan ENSPPYG00000014668 2361 ATGACTAGCATCTTCCATTTTGCCATTATCTTCATGCTAATACTTCAGATCAGAATACAATTATCTGAAGAAAGTGAATTTTTAGTTGATAGGTCAAAAAACGGTCTCACCCATGTTCCTAAAGACCTATCCCAGAAAACAACAGTCTTAAATATATCTCAAAATTATATATCTGAGCTTTGGACTTCTGACATCTTATCACTGTCAAAGCTGAAGATTTTGATAATTTCTCATAATAGAATCCGGTATCTTGATATCAGTGTTTTCAAATTCAACCAGGAATTGGAATACTTGGATTTGTCCCACAACAAGTTGGCGAAGATTTCTTGCCACCCTACTGTGAACCTCAAGCACTTGGACCTGTCATTTAATGCGTTTGATGCCCTGCCTATATGCAAAGAGTTTGGCAATATGTCTCAACTAAAATTTCTGGGGTTGAGCACCACACACTTAGAAAAATCTAGTGTGCTGCCAATTGCTCATTTGAATATCAGCAAGGTCTTGCTGGTCTTAGGAGAGGCTTATGGGGAAAAAGAAGACCCTGAGGGCCTTCAAGACTTTAACACTGAGAGTCTGCACGTTGTGTTCCCCACAAACAAAGAATTCCATTTCATTTTAGATGTGTCAGTCAAGACTGTAGCAAATCTGGAACTATCTAATATCAAATGTGTGCTAGAAGATAACAAATGTTCTTACTTCCTAAATATTCTGGCAAAACTTCAAACAAATCCAAAGTTATCAAGTCTTACCTTAAACAACATTGAAACAACTTGGAATTCTTTCATTAGGATCCTCCAGCTGGTTTGGCATACAACCGTATGGTATTTCTCAATTTCAAACGTGAAGCTACAGGGTCAGCTGGACTTCAGAGATTTTAATTATTCTGGCACTTCCTTGAAGGCCTTGTCTATACACCAAGTTATCAGCGATGTGTTCAGTTTTCCACAAGGGGCTATCTACAAAATCTTTTCGAATATGAACATCAAAAATTTCACAGTGTCTGGTACACGCATGGTCCACATGCTTTGCCCATCCAAAATTAGCCCGTTCCTGCATTTGGATTTTTCCAATAATCTCTTAACAGACACGGTTTTTGAAAATTGTGGGCACCTTACTGAGTTGGTGACACTTATTTTACAAATGAATCAATTAAAAGAACTTTCAAAAATAGCTGAAATGACTACACAGATGAAGTCTCTGCAACAATTGGATATTAGCCAGAATTCTGTAAGCTATGATGAAAAGAAAGGAGATTGTTCTTGGACTAAAAGTTTATTAAGTTTAAATATGTCTTCAAATATACTTACTGACACTATTTTCAGATGTTTACCTCCCAGGATCAAGGTACTTGATCTTCACAGCAATAAAATAAACAGCATTCCTAAACAAGTCGTAAAACTGGAAGCTTTGCAAGAACTCAATGTTGCTTTCAATTCTTTAACTGACCTTCCTGGATGTGGCAGCTTTAGCAGCCTTTCTGTATTGATCATTGATCACAATTCAGTTTCCCACCCATCAGCTGATTTCTTCCAGAGCTGCCAGAAGATGAGGTCAATAAAAGCAGGGAACAATCCATTCCAATGTACCTGTGAGCTAAGAGAATTTGTCAAAAATATAGACCAAGTATCAAGTGAAGTGGTAGAGGGCTGGCCTGATACTTATAAGTGTGACTACCCAGAAAGTTATAGAGGAACCCTACTAAAGGATTTTCACTTGCCTGAATTATTCTGCAACATAACTCTGCTGATCATCACCATCGTTGCCACCATGCTGGTGTTGGCTGTGACTGTGACCTCCCTCTGCATCTACTTGGATCTGCCCTGGTATCTCAGGATGGTGTGCCAGTGGACCCAGACCCGGCGCAGGGCCAGGAACATACCCTTAGAAGAACTCCAAAGAAATCTCCAGTTTCATGCATTTATTTCATATAGTGGGCACGATTCTTTCTGGGTGAAGAATGAATTATTACCAAACCTAGAGAAAGAAGGTATGCAGATTTGCCTTCATGAGAGAAACTTTGTTCCTGGCAAGAGCATTGTGGAAAATATCATCAGCTGCATTGAGAAGAGTTACAAGTCCATCTTTGTTTTGTCTCCCAACTTTGTCCAGAGTGAGTGGTGCCATTATGAACTCTACTTTGCCCATCACAATCTCTTTCATGAAGGATCTAATAACCTAATCCTGATCTTGCTGGAACCCATTCCACAGTACTCCATTCCTAGCAGTTATCACAAGCTCAAAAGTCTCATGGCCAGGAGGACTTATTTGGAATGGCCCAAGGAAAAGAGCAAACGTGGGCTTTTTTGGGCTAACCTAAGGGCAGCCATTAATATTAAGCTGACAGAGCAAGCAAAGAAATAG

>SscrTLR1 Sus scrofa Pig AB219564 2391 ATGACTAAAGAGAATCTTAGCATCTTCCATTTTGCCATCATCTTCATATTAATACTTGAGATCAGAATTCAATTATCTGAGGAAAGTGAAGTTTTAGTTGACAGATCAAAAACAGGTCTCACCCATGTTCCCAAAGACCTATCCTTGGAAACGACAATCTTAGATCTATCACAAAACTCTATCTCTGAGCTTCAGACTTCTGACATCCTCTCACTATCAAAGCTGAGGGTTTTCATAATTTCTCATAATAGAATACAGTATCTTGATGTCAGTGTTTTCAAATTCAACCAGGAACTGGAATACTTGGATTTGTCCCACAACAAGTTGGAGAAGATTTCGTGCCACCCTATGCTGAACCTCAAGCACTTGGACCTCTCATTTAATGCATTCGATGCCCTGCCCATATGCCAAGAGTTTGGCAGTATGTTTCAACTAGAATTTCTGGGGTTGAGTGCCACACAGTTACAAAAATCCAGTGTGTTGCCAATCGCTCATTTGCATATCGGTAAGGTTTTACTTGTCTTAGGAGACTCTTACGGGGAAAGAGAAGACCCTGAGAGCCTTCAAGACCTTAACACACAGAGTCTGCACATTGTTTATCCCCCAGGAAAGGAATTTCATTTTATGTTGGACGTGTCAGTCAGCACCGCAGTAAATCTGGAACTGTCCAATATCAGATGTGTGCTGGATGCTAACGGATGTCATCACTTCCAAAATGTTCTATTGAAACTTCAAAAGAACTCAAAGTTATCCAACCTGACTTTGAACAACATTGAAACCACTTGGAATTCTTTCATCACCACCCTCCAGTTTGTCTGGCGTACAAGCATCGAGTATTTCTCCATTTCCAGTGTGAAACTACAAGGTCAGCTGGACTTCAGAGATTTTGATTATTCTGACACGTCACTGAAGGCCTTGTCTCTACACCAAGTTGTCAGTGAGGTGTTCAGTTTCCCACAAAGTTACATCTATAAAATCTTTTCAAATATGAACATTCAGTATCTCACAGTGTCTGCCACACACATGGTCCACATGGTTTGCCCATCCCAAATTAGCCCATTTCTATATTTGGATTTTTCCAATAATGCCTTAACAGACATGGTTTTTAAAAATTGTGCAAACTTGGCTAATTTGAACACACTCAGTTTACAAATGAATCAGTTAAAAGAACTTGTGAATGTCATCCATATGACCAAAGAAATGCAGTCTCTACAGCAATTGGATGTTAGCCAGAATACCCTGAGGTATGATGAAAATGAGGGAAGTTGCACTTGGACCGGAAGTTTATTAAGTTTAAATTTATCTTCAAATATACTCACTGACTCTGTTTTCAGATGTTTACCTCCCAGGATCAAGGTTCTTGATCTTCACAATAACAGAATAAGGAGCATCCCTAAGGATGTCGCCCATCTGGAAGCTCTGCAAGAACTCAATGTTGCTTCCAATTCTTTAGCTCACCTGCCTGGATGTGGTTCCTTTAGCAGCCTTTCCATTCTGATCATTGACTATAATTCAATTTCCAACCCATCAGCTGACTTCTTCCAGAGCTGCCAGAAGATTAGGTCCCTCAAAGCAGGGAACAATCCATTCCAATGTACATGTGAGCTAAGAGACTTCATCCAAAGTCTAGGTCAAGTATCGAGTGACGTGGTAGAGAGTTGGCCTGATTCTTACGAGTGTGAGTATCCAGAAAGCTATAAGGGGACTCTGCTCAAGGACTTCCGTGTATCTGAATTATCCTGCAACACAGCTCTGCTGATTGTCACCATCGGAGTCACTGGGCTGGCACTGGCTCTTACCATGACCGGCCTCTGTGTCTACTTTGATCTGCCCTGGTATCTCAGGATGCTGTGTCAGTGGACCCAGACTCGGCGCAGGGCTAGGAATGTACCCTTAGAAGAACTCCAAAGAACTCTCCAGTTCCATGCCTTCATTTCATATAGTGGGCATGATTCTGCTTGGGTAAAGAATGAATTACTACCAAATGTAGAAAAAGAAGGTATAAAGATTTGTCTCCATGAGAGAAACTTTGTTCCTGGCAAGAGCATCATGGAAAATATCATAAACTGCATTGAGAAAAGCTACAAGTCCATCTTTGTTTTGTCTCCCAACTTTGTCCAGAGCGAATGGTGCCACTATGAACTCTACTTTGCCCACCACAACCTCTTCCATGAAGGGTCTGATAACTTAATCCTGATCTTGCTGGATTCCATTCCACAGTATTCCATCCCCAGCAGCTATCACAAACTCAAAGCTCTCATGGCACAGCGAACTTATTTGGAATGGCCCAAGGAGAAGAGCAAACATGGACTTTTTTGGGCTAATCTGAGAGCATCCATTAATATTAAATTGATGGAGAAAGCAGAAGAAATAAGTTACACACAGATCTAA

>RnorTLR1 Rattus norvegicus Rat ENSRNOG00000042926 2388 ATGACTAAAACACAGTCCACCATCTTCTATTGTATTGTTGTCTTAGGGCTGATACTTATCAAAATCCAGTTATCTGAGGAAAGTGAGTTGATCATTAAGAGGCCAAATGCAAACCTTACCAGAGTGCCCAAGGACCTACCCTTGCAAACAACTACTTTAGATGTATCACAAAACAATATATCTGAGCTTCAGACTTCTGACATACTCTTGTTGTCCAAACTGAGGGTCTTCATTATGTCCTACAACAGACTCCAGTATCTTAATATCAGTGTTTTCAAATTCAACACGGAACTGGAATATTTGGATTTGTCCCACAATGAGTTAAGGCTGATCTCTTGCCACGCAACAGCCGACCTCAAACATTTAGACCTCTCCTTTAATGCATTTGATGCCCTGCCCATATGCAAAGAGTTTGGCAACCTGTCCCAACTACAGTTTCTGGGATTGAGCGGTTCTCAGATACAAAATTCAAGTGTGCAGCTGATTGCTCATCTGAACATCAGTAAGGTTTTGCTGGTGTTAGGAGACACTTATGGGGAAAAAGAAGATCCCAAGTGTCTTCAGCACATTAGCACTGAGACTCTGCATATCGTTTTCCCTTCCAAAAGAGAATTCCATTTTCTTCTGGACATGTCTGTCAGCACGGCCATCAGTTTGGAACTGTCTAACATCAAGTGTGTGCTTGAGGACAAGAACTGCTCTTACTTCCTAGGTACCTTAGAAAGACTTAGAAAGACTCAGAGGCTCTCAAATCTTACCCTGAACAACGTGGACACAACATGGAATTCCTTCATTAACATCCTTCAGCTGGTTTGGCATACACCAGTCAAGTCTTTCTCAATTTCAAACGTGAAACTAAAAGGTCATTTTAACTTCAGAAGATTCCATTATTCTGACACTTCTCTGAGGGCTTTGTCGATACATCAAGTTGTCACTGATGTGTTCAGCTTCCCCCAAAGTAACATATACAGCATCTTCTCCAATATGAACATCCAAAGTTTTACAGTATCTGGAACACGCATGGTCCACATGCTTTGCCCAGACCAAATTAGCCCATTTCTGTATTTGGACTTTACAGATAACCTTTTAACAGACATAGTTTTTGAAGACTGTAGAAATTTAATTAGATTGAAAACACTTAGTTTACAAAAGAATCAGTTAAAAACACTTGAAAATATAATTCTTATGTCTATGGAGATGACATCCCTACAAAAACTAGACATTAGCCAGAATTCTCTAAGGTACAGTGATGCGGGAAGCCCATGCTCCTGGACCCAGAGTTTGTTAGTTTTAAATTTGTCTTCAAACATGCTTACGGACTCTGTCTTCAGGTGCTTACCTCCCAAGGTCAAGGTCCTTGACCTTCACAACAACAGGATAGTGAGCATCTCTAAAGATGTCACCCACCTGCAAGCTTTGCAGGAACTCAATGTCGCATCCAATTTTTTAACTGACCTTCCTGGATGTGGAGCCTTCAGTAGCCTTTCTGTGCTGGTCATCGACCATAACTCAGTTTCCCACCCCTCCTCTGATTTCTTCCAGAGCTGTCAGAATATCAGGTCCATAACAGCGGGGAACAACCCATTCCGATGCACATGTGAGCTGAGGGAGTTTGTCAAAAACATAGGTCAGGCATCAAGAGAAGTGGTGGAGGGCTGGCCTGACTCTTACAGGTGTGATTACCCAGACAGCATTAAGGGAACCCCACTGCAGGACTTCCACATGTCTCCACTGTCCTGCGATACAATTCTACTGACTGTCACCATTGGGGCCACTCTGCTGCTGCTGGCTGCCATTGGGGCTTCCCTCTGTCTCTACTTTGATCTGCCCTGGTATCTCAGGATGCTATGGCAGTGGACACAGACCAGGCACAGGGCCCGGAACATCCCCTTAGAGGAACTGCAGAGGAACCTCCAGTTCCATGCTTTTGTCTCATACAGTGGGCATGATTCTGCCTGGGTGAAGAATGAATTACTACCAAACCTAGAGAAAGATGACATTCGGGTTTGCCTCCATGAGAGAAACTTTGTCCCTGGCAAGAGCATTGTGGAGAACATCATACACTTCATTGAGAAGAGTTACAAGTCCATCTTTGTGCTGTCTCCCCACTTCATCCAGAGTGAGTGGTGCCATTATGAACTCTACTTTGCCCATCACAATCTCTTCCACGAAGGGTCTGATAACTTAATCCTGATCTTGCTGGAACCAATTCCACAGTACTCCATCCCTACCAATTACCACAAGCTCAAAACTCTCATGGCACGGAGGACCTATTTGGAATGGCCCACAGAAAAGAGCAAGCATGGACTCTTTTGGGCAAATCTAAGAGCATCCATTAATGTTAAGCTGGTCAACCAGGCAGAAGCAACATGTTACACACAGCAATAA

>MmulTLR1 Macaca mulatta Rhesus Monkey XM_001088852 2361 ATGACTAGCATCTTCCATTTTGCCATTATCTTTATGTTAACACTTCAGATCAGAATACAATTATCTGAAGAAAGTGAATTTTTAGTTGATAGGTCAAAAAACAGTCTCATCCACGTTCCTAAAGACCTATCCCAGAAAACAACAATCTTAAATATATCACAAAATTATATATCTGAGCTTTGGACTTCTGACATCTTATCACTGTCAAAGCTGAGGATTTTGATAATTTCTCATAATAGACTCCAGTATCTTGATATCAGTGTTTTCAAATTCAACCAGGAATTGGAATACTTGGATTTGTCCCACAACAAGTTGGCGAAAATTTCTTGCCACCCTACTGTGAACCTCAAGCACTTGGACCTGTCCTTTAATGCATTTGATGCCCTGCCTATATGCAAAGAGTTTGGCAATATGTCTCAACTAAAATTTCTGGGGTTGAGTACTACACACTTAGAAAAATCTACTGTGCTGCCAATTGCTCATTTGAATATCAGCAAGGTCTTGCTGGTCTTAGGAGAGCATTATGGGGACAAAGAAGACCCTGAGGGCCTTCAAAACTTTAACACTGAGAGTCTGCACATTGTGTTCCCAACAAGCAAAGAATTCAATTTTATTTTGGATGTGTCAGTCAGGACTGTAGCAAATCTGGAACTATCTAATATCAAATGTGTGCTAGAAGATAACGAATGTTCTTACTTCCTAAATATTCTGGCAAAACTTCAAACAAATCCAAAGTTATCAAGTCTTACTTTAAACAACATTGAAACAACTTGGAATTCTTTCATTAGGATCCTCCAGCTGGTTTGGCATACAACCGTATGGTATTTCTCAATTTCAAACGTGAAGCTACAGGGTCAACTGGACTTCAGAGATTTTGATTATTCTGGCACTTCCCTGAAGGCCTTGTCTGTACACCAAGTCGTCAGCGATGTGTTCAATTTTCCACAAAGGGATATCTATGAAATCTTTTCAAATATGAACATCAAAAATTTCACAGTGTCTGGTACACGCATGATCCACATGGTTTGCCCATCCAAAATCAGCCCGTTCCTGCATTTGGATTTTTCCAATAATCTCTTAACAGACACGGTTTTTGAAAATTGTGGGCACCTTACTGAGTTGGAGACACTTATTTTACAAATGAATCAATTAAAAGAACTTTCAAAAATAGCTGAAATGACTACACGGATGAAGTCTCTGCAACAATTGGATATTAGCCAGAATTCTGTAAGCTATGATGAAAAGAAAGGAGATTGCTCTTGGACTAAAAGTTTATTAAGTTTAAATATGTCTTCAAATATACTTACTGACACTATTTTCAAATGTTTACCTCCCAGGATCAAGGTACTTGATCTTCACAGCAATAAAATAAAGAGCATTCCTAAACAAGTCATAAAACTGGAAGCTTTGCAAGAACTCAATGTTGCTTTCAATTCTTTAACTGACCTTCCTGGATGTGGCAGCTTTAGCAGCCTTTCTGTATTGATCATTGATCACAATTCAGTTTCCCACCCATCAGCTGATTTCTTCCAGAGCTGCCAGAAGATGAGGTCAATAAAAGCAGGAAACAATCCATTCCAGTGTACCTGTGAGCTAAGAGAATTTATCAAAAATATAGAGCAAGTATCAAGTGAAGTGGTAGAGGGCTGGCCTGATTCTTATAAGTGTGACTACCCAGAAAGTTATAGAGGAACCCCACTAAAGGACTTTCACATGTCTGAATTATCCTGCAACATAACTCTGCTGATCGTCACCATCGGTGCCACCATGCTGGTGTTGGCTGTGACTGTGACCTTCCTCTGCATCTACTTGGATCTGCCCTGGTATCTCAGGATGGTGTGCCAGTGGACCCAGACCCGGCGCAGGGCCAGGAATGTACCCTTAGAAGAACTCCAAAGAAATCTCCAGTTTCATGCATTTATTTCATATAGTGGGCACGATTCTTTCTGGGTGAAGAATGAATTATTACCAAACCTAGAGAAAGAAGGTATGCAGATTTGCCTTCATGAGAGAAACTTTGTTCCTGGCAAGAGCATTGTGGAAAATATCATCAACTGCATTGAGAAGAGTTACAAGTCCATCTTTGTTTTGTCTCCCAACTTTGTCCAGAGTGAGTGGTGCCATTATGAACTCTACTTTGCCCATCACAATCTCTTTCATGAAGGATCTAATAACTTAATCCTGATCTTGCTGGAACCCATTCCGCAGTACTCCATTCCTAGCAGCTATCACAAGCTCAAAAATCTCATGGCCAGGAGGACTTATTTGGAATGGCCCAAGGAAAAGAGCAAACATGGGCTTTTTTGGGCTAATCTAAGGGCAGCCATTAATATTAAGCTGACAGAGCAAGCAAAAAAATAG

>DnovTLR6 Dasypus novemcinctus Nine-Banded Armadillo DQ113655 2370 ATGACCAAAGACAAAGAACCTATTGTCAAAAACTTTCATTTAGTTTGCATTGTGACCTTAATAGTTGGAACCATAATCCAGTTCTCTGATGCAGATGAATTTGCAATAGACATGTCGAAAACAGGCCTTACTCATGTCCCAAAAGACCTGCCACCAAAAACTAAAGTCTTAGATATGTCTCAAAACTACTTATCTGAGCTACAGATCTCTGATATCAGCTTTCTGTCAGGACTGAAAATTTTGGTACTTTCCCACAATAGACTCCAGCTCCTTGATTTAAGTGTTTTCAAGTTTAACCAGGATTTGGAATATTTGGATTTATCTCACAATCAGTTGAAGAAGATGTCCTGCCACCCTTTTGTAAATCTCAAGCATTTAGACCTCTCATTCAATGACTTTGATTCTCTGCCCATATGTAAGGAATTTGGCAATTTAACACAACTGGATTTCTTGGGATTAAGTGCTACTAAATTACAACAATTAGATCTGCTGCCAATTGCTCATTTGCATCTAAATTGCATCCTTCTGGATTTAAAGGGCTACTATGTGAAACAAAATGAGACGGAAAGTCTTCAAATTCTGAATACAAAAAAACTTCACCTTGTTTTTCATACGGGAAGTTTTTTCTCTGTCCAAGTGAATATGTCAGTTAACACTTTAGGGTGTTTAGAAATGACTAATATTAAATTGAATGATAAGAACTGTTACTTTTTAATGAAATTTTTATTAGAACTCACCAAAGGCCCAAGCTTACTGAATTTTACTCTGAACCATATGGAAACAACTTGGAAATGCTTGGTTAGAGTTTTTCAGTTCCTTTGGACCAAACCTGTAGAATATCTCAATATTTACAATTTAACAATAGTTGATGACATCAATAAAGAATATTTTATTTATTGTAAAACTGCATTGAAAGCATTGAAAATTGAACATATTACAAAAACAGTTTTTATCTTTTCATGGTCATCGCTATACACACTGTTTTCTGAGATGAACATTATGATGTTAAGCATAACTGATACACCGTTTATACACATGCTTTGTCCAAAGACACGAAGCACATTTAAGTTTTTGGATTTTACCCAGAATGTTTTCACAGATAGTATTTTTGAAAATTGCTCTACTTTAGTTGAATTGGAGACTCTTATCTTACAAAAGAATGGGTTAAAAGATCTTTTCAAAATAGGTCTCATGACTAAGGGCATGCCGTCTTTGGAAATACTGGATCTTAGCTGGAATTCTTTGGTATTTAATAGACAGAGGAAATGCATTTGGGTTGGAAGTATATTAATGTTAAATATGTCTTCAAATTTACTTACAGATCTGGTTTTCAGATGTTTACCTCCCAGGGTCACAGTACTTGATCTTCACAATAACAGAATAATGAGCATCCCTAAAGATGTCACCAGTCTGGAAACTTTGCAAGAACTAAATATTGCTTTCAATTCTTTAACTGACCTTCCTGGATGTGGAACCTTCAGCAGCCTTTCTGTACTAATCATTGACTATAATTTAATTTCCCACCCATCTACTGATTTCATCCAGAGCTGCCAGAACATTACATCAATAAAAGCAGGGAAAAATCCATTCCAATGTACATGTGACTTAAGAGAATTTATCAAAACCATAAGTCAAATGTCCAGTGAAGTGGTTAAGGACTGGCCTGATTCTTATAAGTGTGACTACCCAGAAAGTTATAAGGGAACCCTCCTACAGGACTTTCACATTTCTCAGTTATCCTGCAGCACCTCTCTGCTGACTGTCACCATTGGGGCCACCATGCTAGTGTTGGTTGTTACCGTGACCTTCCTCTGCATCTACTTGGATCTGCCCTGGTATATCAGGATGGTGTATCAGTGGACACAGACCCGACGCAGGGCTAGGAACATACCCCTGGAAGAACTCCAGAGAACTCTCCAGTTCCATGCTTTTATTTCATACAGCGGGCATGATTCTGCCTGGGTGAAGACTGAATTACTACCAAACCTAGAGAAAGAAGATATACAGATTTGCCTTCATGAGAGAAATTTTGTTCCTGGTAAGAGCATCATTGAAAATATCATAAACTTCATTGAGAAGAGTTACAAGTCCATCTTTGTTTTGTCTCCCAACTTTGTCCAGAGTGAGTGGTGCCATTATGAACTCTACTTTGCCCATCACAATCTCTTTCATGAGGCTTTTGATAACTTAATCCTCATCTTGCTGGAACCCATTCCACAGTACTCCATTCCTAACAACTATCACAAGCTCAAATCTCTTATAGCACAGAGGACTTATTTGGAATGGCCCAAAGAAAAGAGCAAACATGGACTCTTTTGGGCTAACCTAAGAGCAGCCATTAATATTAAGTTGATGGAAGAAAAGAAATAG

>CgarTLR6 Ctolemur garnettii Bushbaby ENSOGAG00000016497 2385 ATGGGCCAAGACAAAGAACTTATTGTCAGAAGCTTCCATTTTGTTTGCATTATGACCGTAATCATTGGAACCATAAGCCACTTCTCTGATGAAAGTGAATTTGCAATAACCATGTCAAAAATAGGCCTTACTCATATTCCAAAAGACCTACCACCAAAAACCAAAGTCTTAGACATATCTCAAAACAACATATCTGAGCTTCAGATCTCTGATATCAGCTTTCTGTCAGGGTTGAAAGTTTTGAGACTTTCCTATAATAGAATCCAGTGGCTTGATTTTAGTATTTTCAAGTTCAACCAGGATTTGGAATATTTGGATTTATCTCATAATCAGTTGCGAAAGATATCCTGCCATCCTATTGTGAGTTTCAAGCATTTGGACCTCTCATTCAATGACTTTGATGCCCTGCCCATCTGTAAGGAATTTGGCAATTTGACACAACTGAATTTCTTGGGATTAAGTGCTATGAAGTTACAACAATTTGATCTGCTGCCAATCGCCCACTTGCATCTAAGTTACCTCCTTCTGGATTTAGGGAATTACCATGTAAAGGAGTCAGAAAGTCTTCAAATTCTAAATATAAAAACACTTCACCTTGTTTTTCATCCAAATAGTTTATTCTCTGTCCAAGTGAACATATCAGTTAATACTTTAGGATGCTTAAAACTGACTAATATTAAGTTGGATGATGAGAACTGTGAAGTTTTAATTAAGTTTTTGTCAGAGCTCACTAGAGTTTCAACCTTACTGAATTTTACCCTCAACCACATGGAAACAACTTGGAAGTGCTTGGTTAGAGTCTTGCAGTTTCTTTGGCCCAGGCCTGTGGAATATCTCACAATTTACAACTTAACAATAGTTGAAAGCATTGATGAAGAAGATTTTACTTATTCTAAAACAGCATTGAAAGCACTGACAATAGAACATGTCACAAACAAAGTGTTTCTTTTTCCGCAGACCGCATTATACACAGTGTTTTCTGAGATGAACATTATGATGTTAACCATATCAGATACGCCTTTTATACACATGCTTTGCCCTCAGGCACCAAGCACATTTAAATTTTTGAACTTTACCCAGAATGTTTTCACAGACAGTGTTTTTCAAAATTGTTTCACATTAGTTAGATTGGAGACACTTATCCTACAAAAGAATGGATTAAAAGACCTTTTCAGTGTAGGTCAGATGACTAAGAACATGCCATCTTTGCAAATACTAGATGTTAGCTTCAATTCTTTGGAATCTGATAGGTGTGAGGAAAATTGCACTTGGGTTGAGAGTATAGTGATGTTAAATTTGTCTTCAAATAAACTTACTGACTGTGTTTTCAGATGTTTACCTCCCAGGCTTGAGGTACTTGATCTTCACAGTAACAGAATAGAGAGCATCCCTAAAGATGTCACTCATCTGGAAGCTTTGCAAGAACTCAATGTTGCTTTCAATTCTTTAATTGACCTCCCTGGATGTGACACCTTTAGCAGCCTTTCTGTACTGGTCATTGACCACAATTCCATTTCCCAGCCATCAGATGAATTCTTCCAGAGCTGCCAGAAGATCAGGTCAATAAAAGCGGGGAACAATCCATTCCAATGTATGTGTGAACTAAGAGAATTTGTCAAAAATGTAGGCCAAGTATCAAGTGAAGTGGTAGAGGACTGGCCTGATTCTTATAAGTGTGACTATCCTGAAAGCTATAAGGGAATACCGCTAAAGGATTTTCACATGTCTGAATTATCCTGCAATACAGCTCTGCTGATGGTCACCACTGGGGCCATCATGCTGGTGGTGGCTGTTACTGTGACCTTCCTCTGCATCTATTTGGATCTGCCCTGGTATCTTAGGATGGTGTTCCAGTGGACCCGGACCCGATACAGGGCTAGGAACATACCGTTAGAGGAACTCCAAAGAGCTCTCGAGTTCCATGCCTTTATTTCATATAGCGAACATGATGCTGCCTGGGTGAAGAATGAATTGGTACCTTGCCTAGAAAGAGAAGATATACGGATTTGCCTCCATGAGAGAAACTTTGTTCCTGGCAAGAGCATTGTGGAAAATATCATCAACTGCATTGAGAAGAGTTACAAGTCTATCTTTGTTTTGTCTCCCCACTTTGTCCAGAGTGAGTGGTGCCATTATGAACTCTACTTCGCCCACCACAATCTCTTCCATAAAGGATCTAACAATTTAATCCTGATCTTGCTAGAACCTATTCCACAGAACATGATTCCCAGCAAGTATCATAAGCTGAAGTCTCTCATGATGCAACGGACTTATTTGGAATGGCCCAAGGAGAAAAGCAAGCATGGACTTTTTTTGGCTAACATTAGAGCTGCTTTTAATGTGAAATTAATACTAGACACTGAAAATGATCATGTGAAAATTTAA

>BtauTLR6 Bos taurus Cow chr6:60,372,842-60,375,193 (bosTau4) 2382 ATGATCAAAGACAAAGAATCTCCCATCAGAAGCTGTCATTTTGTTTACATTGTGGCCTTAGTATTTGGAACCATAATCCAGTTCTCTGATGAAAGTGAATTTGTGGTAGACATGTCAAAAACAAGCCTTATTCATGTTCCCAAAGACCTGCCACCAAAAACCAAAGTCTTAGACTTGTCTCAAAACAACATATCTGAGCTTCACCTGTCTGATATCAGCTTTCTCTCAGGGCTGAGAGTTCTGAGACTTTCCCATAATAGAATCCAGGGCCTTGATATTAGTATTTTCAAGTTCAACCATGATTTGGAATATTTGGATTTATCTCATAATCAGTTGCAGAAGATATCCTGCCATCCAATCACCACGACTCTCAAGCATTTAGACCTCTCATTCAATGACTTCGATGCCCTGCCCATCTGTAAGGAATTTGGCAACTTGACCCAACTGAATTTCTTAGGATTAAGTGCTACAAAGTTACAACAATTAGATTTACTACCCATTGCTCACTTGCACCTAAGTTGTATCCTTCTGGATTTGGAAGACTATATGAAAGAAAATAAGAAAGAAAGTCTTCAAATTCTGAATACAAAGAAACTTCACCTTGTTTTTCACCCAAATAGCTTTTTCTCTGTCCAAGTGGACATATCAGGGAATAGTTTAGCGTGCTTACAACTGACTAATATTAAATTGAATGATTACAATTGTCAAGTTTTACTTAAATTTTTATCAGGACTCACTGGAGGACCAACCTTACTAAATTTTACCCTCAACCACGTGGAAACAACTTGGAAATGTTTGGTTAAAGTTTTTCAGTTCCTTTGGCCCAAACCTATAGAATATCTCAATATTTACAATTTAACAATAGTTGAAAGCATTGATGAAGAAGTTTTTACTTATTATAAAACGACATTGAAGGCACTGAAAATAGAACATATTACAAACAAAGTTTTTATTTTTTCACAGACAGCATTATACACAGTGTTTTCTGAGATGAACATTCTGATGTTAACCATATCAGACACACGCTTTATACACATGCTTTGTCCTCAGGAACCAAGCACATTTAAGTTTTTGAACTTTACCCAGAATAGTTTCACAGATAGTGTCTTTCAAAATTGTGATACTTTAGCTAGATTGGAGACACTTATCTTACAAAAGAATGAATTAAAAGACCTTTTCAAAACAAGTCTCATGACTAAGGATATGCTTTCTTTGGAAACACTGGATGTTAGCTGGAATTCTTTGGAATATGACAGAAGTAATGGAAATTGCTCTTGGGTTGGGAGTATAGTGGTGTTAAATTTATCTTCAAATGCACTCACTGACTCTGTTTTCAGATGTTTACCTCCTCGGATCAAGGTTCTTGATCTTCACAATAACAGAATAAGGAGCATCCCTAAAGATGTCACTGGTCTAGAAACTTTGCAAGAACTCAACCTTGCTTCCAATTCTTTAGCCCACCTTCCTGGATGTGGTATCTTTAGCAGCCTTTCCATACTGATCATTGAACATAATTCAATTTCCAATCCATCAGCTGATTTCTTCCAGAGCTGCCAGAAGATTAGGTCCCTCAAAGCGGGGAACAATCCATTCCAATGTTCCTGTGAGCTAAGAGACTTTATCCAAAGTGTAGGCCAAGTATCCAGTGACGTGGTAGAGGGCTGGCCTGAGTCTTATAAGTGTGACTATCCGGAAAGCTACAAGGGAACCCCTCTAAAGGACTTCCAGGTATCTGAGCTATCCTGCAACACAGCTCTGCTGATCATCACCATTGTGGTCCCTGGGCTGGTGCTGGCTGTTGCTGTGACTGTCCTCTGTATCTACCTGGATCTGCCCTGGTACCTCAGGATGGTGTGTCAGTGGACCCAGACCCGGCGCAGGGCCAGGAATGTACCCTTGGAAGAACTCCAAAGAACTCTCCAGTTCCATGCTTTTATTTCATACAGTGAACATGACTCTGCCTGGGTGAAGAATGAATTAATACCTAACCTAGAAAAAGAAGATATAAGAATTTGTCTCCACGAGAGAAACTTTGTTGCCGGCAAGAGCATTGTGGAAAATATCATCAACTGCATTGAGAAAAGTTACAAATCCATCTTTGTTTTGTCTCCCAACTTTGTCCAGAGCGAGTGGTGCCATTATGAACTCTACTTTGCCCACCACAATCTCTTCCATGAAGGATCTGATAACTTAATCCTGATCTTGCTGGATCCCATTCCACAGTATTCCATTCCTAGCAGCTACCACAAGCTAAGAGCTCTCATGGCACAGAGAACTTATTTGGAATGGCCCAAGGAGAAGAGTAAACACGGACTTTTTTGGGCTAACCTAAGAGCATCCATTAATATTAAACTGATGGAAAAAGCAGCAGAAATACATTAA

>PtroTLR6 Pan troglodytes Chimpanzee XM_001139197 2391 ATGACCAAAGACAAAGAACCTATTGTTAAAAGCTTCCATTTTGTTTGCCTTATGATCATAATAGTTGGAACCAGAATCCACTTCTCCGACGGAAATGAATTTGCAGTAGACAAGTCAAAAAGAGGTCTTATTCATGTTCCAAAAGACCTACCGCTGAAAACCAAAGTCTTAGATATGTCTCAGAACTACATAGCTGAGCTTCAGGTCTCTGACATGAGCTTTCTATCAGAGTTGAAAGTTTTGAGACTTTCCCATAACAGAATCCAGCTACTTGATTTAAGTGTTTTCAAGTTCAACCAGGATTTAGAATATTTGGATTTATCTCATAATCAGTTGCAAAAGATATCCTGCCATCCTATTGTGAGTTTCAGGCATTTAGATCTCTCATTCAATGATTTCAAGGCCCTGCCCATCTGTAAGGAATTTGGCAACTTGTCACAACTGAATTTCTTGGGATTGAGTGCTATGAAGCTGCAAAAATTAGATTTGCTGCCAATTGCTCACTTGCATCTAAGTTATATCCTTCTGGATTTAAGAAATTATTATATAAAAGAAAATGAGACGGAAAGTCTACAAATTCTGAATGCAAAAACCCTTCACCTTGTTTTTCACCCAACTAGTTTATTCGCTATCCAAGTGAACATATCAGTTAATACTTTAGGGTGCTTACAACTGACTAATATTAAATTGAATGATGACAACTGTCAAGTTTTCATTAAATTTTTATCAGAACTCACCAGAGGTCCAACCTTACTGAATTTTACCCTCAACCACATAGAAACGACTTGGAAATGCTTGGTCAGAGTCTTTCAATTTCTTTGGCCCAAACCTGTGGAATATCTCAATATTTACAATTTAACAATAATTGAAAGCATTCATGAAGAAGAGTTTACTTATTCTAAAACGACATTGAAAGCATTGAAAATAGAACATATCACGAACAAAGTTTTTCTGTTTTCACAGACAGCGTTGTACACCGTGTTTTCTGAGATGAACATTATGATGTTAACCATTTCAGATACACCTTTTATACACATGCTGTGTCCTCATGCACCAAGCACATTCAAGTTTTTGAACTTTACCCAGAACGTTTTCACAGATAGTATTTTTGAAAAATGTTCCACGTTAGTTAAATTGGAGACACTTATCTTACAAAAGAATGGATTAAAAGACCTTTTCAAAGTAGGTCTTATGACTAAGGATATGCCTTCTTTGGAAATACTGGATGTTAGCTGGAATTCTTTGGAATCTGGTAGACATAAGGAAAACTGCACTTGGGTTGAGAGTATAGTGGTGTTAAATTTGTCTTCAAATATGCTTACTGACTCTGTTTTCAGATGTTTACCTCCCAGGATCAAGGTACTTGATCTTCACAGCAATAAAATAAAGAGCGTTCCTAAACAAGTCATAAAACTGAAAGCTTTGCAAGAACTCAATGTTGCTTTCAATTCTTTAACTGACCTTCCTGGATGTGGCAGCTTTAGCAGCCTTTCTGTATTGATCATTGATCACAATTCAGTTTCCCACCCATCAGCTGATTTCTTCCAGAGCTGCCAGAAGATGAGGTCAATAAAAGCAGGGGACAATCCATTCCAATGTACCTGTGAGCTAAGACAATTTGTCAAAAGTATAGACCAAGTATCAAGTGAAGTGTTAGAGGGCTGGCCAGATTCTTATAAGTGTGACTACCCAGAAAGTTATAGAGGAACCCCACTAAAGGACTTTCACATGTCTGAATTATCCTGCAACATAACTCTGCTGATCATCACCATCGGTGCCACCATGCTGGTGTTGGCTGTGACTGTGACCTCCCTCTGCATCTACTTGGATCTGCCCTGGTATCTCAGGATGGTGTGCCAGTGGACCCAGACCCGGCGCAGGGCCAGGAACATACCCTTAGAAGAACTCCAAAGAAATCTCCAGTTTCATGCTTTTATTTCATATAGTGAACATGATTCTGCCTGGGTGAAAACTGAATTGGTACCTTACCTAGAAAAAGAAGATATACAGATTTGTCTTCATGAAAGAAACTTTGTCCCTGGCAAGAGCATTGTGGAAAATATCATCAACTGCATTGAGAAGAGTTACAAGTCCATCTTTGTTTTGTCTCCCAACTTTGTCCAGAGTGAATGGTGCCATTACGAACTCTATTTTGCCCATCACAATCTCTTTCATGAAGGATCTAATAACTTAATCCTCATCTTACTGGAACCCATTCCACAGAACAGCATTCCCAACAAGTACCACAAGCTGAAGGCTCTCATGATGCAGCGGACTTATTTGCAGTGGCCCAAGGAGAAAAGCAAACGTGGGCTCTTTTGGGCTAACATTAGAGCCGCTTTTAATATGAAATTAACACTAGTCACTGAAAACAATGATGTGAAATCTTAA

>CfamTLR6 Canis familiaris Dog chr3:76,346,360-76,348,753 (canFam2) 2394 ATGATCAAAGACAAAGACTCTATCACTGGAAGCTTCCATTTTGTGTACATTGTGACCTTAATAGTTGGAACCATAATCCAATTCTCTGATGAAAGTGAATTTACAGTAGACATGTCAAATATGAACCTTACTCATGTCCCAGAAGACCTGCCACCAAAAACCAAAATCTTAGATATGTCTCAGAACAATATATCTGAGCTTCACCTCTCTGACATGAGCTATCTCTCAGGACTAAAAATATTGAGAATTTCTCATAATAGAATCTGGTGGCTTGATTTTAGCATTTTCAAGTTCAACCAGGATTTGGAATATTTGGATTTATCTTACAATCAGTTACGGAACATGTCCTGCCATCTTATCAGGAGTCTCAAGCATTTAGACCTCTCATTCAATGACTTTCATGTCCTGCCCATCTGTAAGGAATTTGGCAACTTGACACAACTACAATTCTTAGGTTTAAGTGCTACAAAGTTACGGCAATTAGATCTGCTACCAATTGCTCATTTGCATCTAAGTTATATCCTTCTGGATTTACAAGGTTATTATGCAAAAGAAAGTGAAAAAGGAAGTCTTCAAATTCTAGATACAAAAACACTTCATCTTGTTTTTCATCCAAATCAGTTATTCTCTGTACAAGCAAACATGTTAGTTAATAATTTAGGGTGCTTACAACTGACTAATATTAAGTTGAATAATGACAACTGTCAAGTTTTAATTCAATTTTTATCAGAACTCACCAGAGGTCCAACTTTACTGAATTTTACTCTCCAACATGTGAAAACAACTTGGAAATGCTTGGTTAGAATTTTTAAATTTCTTTGGCCCAAACCTGTACAATATCTCAATATTTATAATTTAACAATAGTTGAAAGCATTAATAAGGAATATATTCATTATCCTAAAACAGCACTGAAAGCATTGACAATAGAGCATGTTAAAAATGAAGTTTTTCTTTTTTCACAGACAGCGTTATACACAATTTTTTCTGAGATGAACATTATGATGTTAACCATATCAGACACACCTTTTATACACATGCTTTGTCCTCCACCATCAAACACATTTAAGTTTTTGAACTTTACCCAGAATGTTTTCACAGATAGTGTTTTTCAAAGTTGTTCCCACCTAGTTAGATTGGAAACACTTATCTTACGAAAGAATAAATTGAAAGACCTTTACAAAGTAGGTCTCATGACGAAGCATATGACATCTTTGGAAATATTGGATGTTAGTGTGAATTCTTTGGAATATGATAGATATGATGGAAATTGCACTTGGGTTGGGAGTATAGTGGTGTTAAATTTGTCTTCAAATATACTTACTGACTCTGTTTTCAGATGTTTACCTCCCAAGGTCAAGGTGCTTGATCTTCACGATAACAGAATAAGGAGCATTCCTAAACCAATCATGAAGCTAGAAGATTTGCAAGAACTCAATGTTGCTTCCAATTCTTTAGCCCACTTTCCTGACTGTGGTACTTTTAATAGGCTTTCTGTACTGATCATTGACTCTAATTCAATTTCCAATCCATCAGCTGATTTCCTCCAGAGCTGCCATAACATTAGGTCCATGAGCGCAGGGAATAATCCATTCCAATGTACATGTGAGCTGAGAGAATTTGTCCAAAGTCTAGGCCAGGTAGCAAGCAAAGTAGTAGAGGGTTGGCCTGATTCTTATAAGTGTGACTCTCCAGAAAACTATAAGGGAACCCTACTGAAGGACTTTCACGTGTCTCCGTTATCCTGCAACACAACTCTGCTGCTTGTCACCATTGGGGTCGCTGTGCTAGTGTTCACTGTTACTGTGACTGCGCTCTGTATCTACTTTGATCTGCCCTGGTATCTTAGGATGGTGTTTCAGTGGACCCAGACCCGGCGCAGGGCAAGAAACACACCCTTAGAAGAACTCCAAAGAACCATCCAGTTCCATGCTTTTATTTCATACAGTGAACATGATTCTGCCTGGGTGAAGAATGAACTGGTACCCTGCCTAGAAAAAGAAGAACTAAGGATTTGTCTCCATGAGAGAAACTTTATTCCTGGCAAGAGCATTGTGGAAAATATCATAAACTGCATTGAGAAAAGTTACAAGTCCATCTTTGTTCTGTCTCCCAACTTTGTTCAGAGTGAGTGGTGCCATTATGAACTGTACTTTGCCCACCACAATCTCTTTCATGAAGGATCTAATAACTTAATCTTGATCTTGCTGGAACCCATTCCACAGAACTGCATTCCCAGCAAGTATCACAAGCTGAGGGCTCTCATGACGCAGCGGACTTACTTGGAATGGCCCAAGGAGAAGAGCAAACATGGACTTTTTTGGGCTAATATTAGAGCTGCTTTTAATATGAAGTTAACACTAATTGCTGAAAACAATAACGCAGAAGCTTCTTAA

>EeurTLR6 Erinaceus europaeus Hedgehog ENSEEUG00000015874 2385 ATGACCAGAGACAAAGAATCTACTATGAGAAGTTTTGCTTATACTGTGATCGTAACAGTTGGGATCGTAATCCAGTCCTCTGATGAAAATGAATTTACAGTAGATTTGTCCAAAAGAGGCCTCACTCATATTCCAAGAGATTTGCCATCCCAAACCGAAGTCTTAGATATGTCTCAAAACAACGTATCTGAACTTCACCTTTCTGATATGAGCCTTATCTCAAGACTCAAAGTTTTAAGACTTTCCCATAACAGGATCCAGTGCCTTGATTTTAGTGTTTTCAGATTCAACCAGGATTTGGAATATTTGGATTTATCCCACAATCAGTTGAAAAAATTGTCCTGCCATGCCATCCAGAGTCTCCAGCATTTAGACCTCTCCTTCAATGACTTTGATAGTTTGCCCATTTGTAAGGAATTTGGCAACTTGACACAACTGAATTTCTTGGGATTAAGTACCTCAAAGTTGCAACAGTTAGACCTGCTACCAGTTTCTCACTTGCATGTAGACAGCATCCTTCTGGATTTACAAGGCTATTCTATGAAAGGAAACGAGACTGGGAGTCTTCAAATTTTCAATACAAAAAAACTTCACTTTGTTTTTCATCCAAATGATTTGTTCTCTGTGCTAGTGAACATATCTGTTAACATGATAGAGTGCTTACAGCTGACTAATATCAAACTGAATGATGACAACTGTCAAGTTTTAATAAATTTCTTATCAGAACCACTTATCAGAGGCCCAAACTTACTGAATTTGACCCTAGATCATGTGGAAACAACTTGGAAATGTTTAGTTAGAGTTTTTCAGTCCCTTTGGTCCAAGCCTATAGAATACCTGAATATTTACAATTTGACAGTAGTTGAAAGAATTGATGAAGAAGAGTTCACTTACTCCAAAACATCATTGAAGGCACTGAAAATAGAGCATATTACAAATAGAGTTTTTCTTTTTTCACAGACTGCATTGTACACAGTGTTTTCTGAGATGAACATTATGATGTTAACCATATCAGAGGCACCTTTAATACACATGCTTTGTCCTCAGGCACCAAGCATGTTCAAGTTTTTGAACTTTACCCACAATGTTTTTACAGATAGTATTTTTCAAAACTGTTCCACACTAGGTAGATTAGAAACACTTATCTTACAGAAGAATGAATTGAAAGAACTTTTTAAAGTAGGTCTAATGACTAAGGATATGCAGTCTTTGGAAATACTGGATGTAAGCTGGAATTCTCTGAACTATGATAGATATGATGGAATTTGTACGTGGGCTCAGAGTATAGTGATGTTAAATTTATCTTCAAATATACTTACTGAGTCTGTTTTCAGATGTCTACCTCCCAGGGTTAAGGTCCTTGATCTTCACAGTAACAGAATAAGGAGCATCCCAAGAGATGTCAACAATCTGGAAGCTTTGCAAGTACTCAATGTTGCTTCCAATTTTTTAACCAACCTTCCTGGATGTGGTGCCTTTAGCAGCCTTTCTGCACTGATCATTGACTATAACTCAATTTCCAGTCCATCAGTTGATTTCTTCCAGAGCTGCCAGAACATTAGGTCAGTAAAAGCAGGGAACAACCCATTCCAGTGTACATGTGAGCTCAGAGAGTTTGTCCAGAGAATGGGCCAAGTGTCAAGGGAAGTGGTAGAGAACTGGCCTGGTTCTTACCAGTGTGACTATCCAGAAAGCTTTAAGGGAACTGCACTAAAGGACTTCCACATGTCTCAGCTGTCCTGCAACACCACTCTGTTGATTGTCACCATTGTGGTCATTGTGCTGGTGTTGGGTAGTACCACGGTCATGCTCTGTATCTACTTTGATGTGCTCTGGTATCTGAGGATGATGTGCCATTGGACCCAGACCCGGCAAAGGGCTAGGAACACTCCCTTAGCTGAACTCCAGAGAAACCTCCAGTTCCATGCTTTCATTTCATATAGTGAACATGATTCCGCCTGGGTGAAGAATGAGCTGGTACCCTGCCTAGAAAAAGAAAATATACGAATTTGTCTTCATGAGAGAAACTTTGTCCCTGGGAAGAGCATCATAGAAAACATTATCAACTGCATTGAGAAAAGTTACAAGTCCATCTTTGTTCTGTCTCCCAACTTTGTACAGAGTGAGTGGTGCCATTATGAGCTCTACTTTGCCCACCACAATCTCTTTCATGAAGGGTCTGATAACTTAATCTTGATCTTGCTGGAACCCATTCCACAGAACAACATTCCTAGTAAGTATCACAAGCTGAAGGCTCTCATGACACAACGAACTTATTTGGAATGGCCTAAGGAGAAGAGCAAACATGGACTTTTCTGGGCTAACATCACAGCTGCTTTTCATATGAAATTAACACTAGTGAATGAAAATGATGCAGAAACTTAG

>EcabTLR6 Equus caballus Horse XM_001498630 2391 ATGACCAAAGACAACAAATGTGTTGTTAGAATCTTTAATTTTGTTTACATCGTGACCTTAATAGTTGGAACCATAATCCAATTCTCTGATGAAAGTAAATTTGCAGTAGACATGTCAAAAATAGGCCTTACTCATGTTCTGAAAGACTTGCCACCAGAAACCAAGGTCTTAGACATGTCTCAAAACTGCATATCTGAGCTTCACCTGTCTGACATGAGCTTTCTCTCAGGGCTGAAAGTCTTGAGACTTTCCCGTAATAGCATCCGGTACCTTGATTTTAGTATTTTCAAGTTCAACCCGGATTTAGAATATTTGGATTTATCTCACAATCAGTTGCAGAAGATTTCCTGCCATCCTATCATGAGTCTCAAGCATTTAGATCTCTCATTCAATGACTTTGAAGTCCTGCCCATTTGTAAGGAATTTGGCAACTTGACTCAACTGGATTTCTTGGGATTAAGTGCTACAAGGTTACAGCAATTAGATCTGCTACCAATTGCTCACTTGCATCTAAGTTGCATCCTTCTTGATTTAGAAGGTTATTATGTGAAAGAAAACCAGACAGAAAGTCTTCAAATTCCAAATACAAAAACACTTCAGCTTGTTTTCCACCCATATAATTTATTCTCTGTCCAAGTGAACATATCACTTAATAGTTTAGGGTGCTTACAACTGACTAATATTAAATTGAATGATGACAACTGTCAAGTTTTGATTAAATTTTTGTCAGACCCCATTAGACAGCCAACCCTACTGAATATTACCCTCAACCATGTGGAAACAACATGGAAATGCTTGGTTAGAGTTTTTCAATTCCTTTGGCCCAAACCTGTAGAATCTCTCCATATTTGCAATTTAACAATAGTTAAAAGCATTGGTAAAGAAGATTTTACCTATTCTAAAACGGCATTGAAAGCATTGAAAATAGAACATATTACAAACAGAGTTTATATTTTTTCACAGCAAGTGTTGTACACAGTGTTTTCTGAGATGAACATTATGATGTTAACCATATCAGATACACCTTTTATACATATGGTTTGTCCTCAGGCACCGAGCACATTTAAGTTTTTGAACTTTACCCAGAATGTTTTCACAGATAGTATTTTTCAAAACTGTTCCACTTTAGTTAGATTGGAGACACTTATCTTACAAAAGAACGAATTAAAAGATCTTTTCAAAGTAGGCCTCATGACTAAGAATATGCCATCTTTGGAAATACTGGATGTTAGCTGGAATTCTCTGGAATATCATGGACCTGATGGAAATTGCCCTTGGGTTGAGAGTCTAGTGGTGTTAAATTTGTCTTCAAATATACTTACTGACTCTGTTTTCAGATGTTTACCTCCCAGGGTCAAGGTACTTGATCTTCACAATAACAGAATAAGGAGCATCCCTAAAGATATCACCAGCCTGGAAGCCCTGCAAGTACTCAATGTTGCTTTCAATTCTTTAGTCGACCTTCCTGGATGTGGTGCCTTTAACAGCCTTTCTATACTGATCATTGACTATAATTTAATTTCCGACCCATCAGCCGATTTCTTCCACAGTTGCCAGAAGATTAGGTCAATAAAAGCAGGGAACAATCCATTCCAATGTACGTGTGAACTAAGAGAATTTATCCAAAGTATAGGCCAAGTATCAAGTGATGTGGTAGAGGGTTGGCCTGATTCTTATAAGTGTGAGTATCCAGAAAGCTATAAGGGAACCCCACTAAAGGACTTTCATCTGTCTCAATTATCCTGCAACACAGCTCTGCTTGTTGTCACCATTGTGGTCCCTGTGCTGGTGTTGGCTGTTACTGTGAGCATCCTCTGTATCTACCTGGATCTGCCCTGGTATCTCAGGATGGTGTGTCAGTGGACCCAGACCCGGCGCAGGGCTAGGAACATACCCTTAGAAGAACTCCAAAGAACTCTCCAGTTCCATGCTTTTATTTCATACAGTGAACGTGATTCTGCCTGGGTGAAGAATGAACTAGTACCTTGCTTAGAAAAAGAAGATATACGGATTTGTCTCCATGAGAGAAACTTTGTTCCTGGCAAGAGCATTGTGGAAAATATCATAAACTGCATTGAGAAAAGTTACAAGTCCATCTTTGTTTTGTCTCCCAATTTTGTTCAGAGTGAGTGGTGCCATTATGAACTCTACTTTGCCCACCACAATCTCTTTCATGAAGGATCTGATAACTTAATCCTGATCTTGCTGGAACCCATTCCACAGAACAACATTCCCAGCAAGTATCACAAGCTCAGGGCTCTCATGACACAGAGGACTTATTTGGAATGGCCCAAGGAGAAGAGCAAACGTGGACTTTTTTGGGCTAACATTAGAGCTGCTTTTAATATGAAAGTAGCACTAGTCATTGAAAACAATGATGTGAAAACTTAA

>HsapTLR6 Homo sapiens Human NM_006068 2391 ATGACCAAAGACAAAGAACCTATTGTTAAAAGCTTCCATTTTGTTTGCCTTATGATCATAATAGTTGGAACCAGAATCCAGTTCTCCGACGGAAATGAATTTGCAGTAGACAAGTCAAAAAGAGGTCTTATTCATGTTCCAAAAGACCTACCGCTGAAAACCAAAGTCTTAGATATGTCTCAGAACTACATCGCTGAGCTTCAGGTCTCTGACATGAGCTTTCTATCAGAGTTGACAGTTTTGAGACTTTCCCATAACAGAATCCAGCTACTTGATTTAAGTGTTTTCAAGTTCAACCAGGATTTAGAATATTTGGATTTATCTCATAATCAGTTGCAAAAGATATCCTGCCATCCTATTGTGAGTTTCAGGCATTTAGATCTCTCATTCAATGATTTCAAGGCCCTGCCCATCTGTAAGGAATTTGGCAACTTATCACAACTGAATTTCTTGGGATTGAGTGCTATGAAGCTGCAAAAATTAGATTTGCTGCCAATTGCTCACTTGCATCTAAGTTATATCCTTCTGGATTTAAGAAATTATTATATAAAAGAAAATGAGACAGAAAGTCTACAAATTCTGAATGCAAAAACCCTTCACCTTGTTTTTCACCCAACTAGTTTATTCGCTATCCAAGTGAACATATCAGTTAATACTTTAGGGTGCTTACAACTGACTAATATTAAATTGAATGATGACAACTGTCAAGTTTTCATTAAATTTTTATCAGAACTCACCAGAGGTTCAACCTTACTGAATTTTACCCTCAACCACATAGAAACGACTTGGAAATGCCTGGTCAGAGTCTTTCAATTTCTTTGGCCCAAACCTGTGGAATATCTCAATATTTACAATTTAACAATAATTGAAAGCATTCGTGAAGAAGATTTTACTTATTCTAAAACGACATTGAAAGCATTGACAATAGAACATATCACGAACCAAGTTTTTCTGTTTTCACAGACAGCTTTGTACACCGTGTTTTCTGAGATGAACATTATGATGTTAACCATTTCAGATACACCTTTTATACACATGCTGTGTCCTCATGCACCAAGCACATTCAAGTTTTTGAACTTTACCCAGAACGTTTTCACAGATAGTATTTTTGAAAAATGTTCCACGTTAGTTAAATTGGAGACACTTATCTTACAAAAAAATGGATTAAAAGACCTTTTCAAAGTAGGTCTCATGACGAAGGATATGCCTTCTTTGGAAATACTGGATGTTAGCTGGAATTCTTTGGAATCTGGTAGACATAAAGAAAACTGCACTTGGGTTGAGAGTATAGTGGTGTTAAATTTGTCTTCAAATATGCTTACTGACTCTGTTTTCAGATGTTTACCTCCCAGGATCAAGGTACTTGATCTTCACAGCAATAAAATAAAGAGCGTTCCTAAACAAGTCGTAAAACTGGAAGCTTTGCAAGAACTCAATGTTGCTTTCAATTCTTTAACTGACCTTCCTGGATGTGGCAGCTTTAGCAGCCTTTCTGTATTGATCATTGATCACAATTCAGTTTCCCACCCATCGGCTGATTTCTTCCAGAGCTGCCAGAAGATGAGGTCAATAAAAGCAGGGGACAATCCATTCCAATGTACCTGTGAGCTAAGAGAATTTGTCAAAAATATAGACCAAGTATCAAGTGAAGTGTTAGAGGGCTGGCCTGATTCTTATAAGTGTGACTACCCAGAAAGTTATAGAGGAAGCCCACTAAAGGACTTTCACATGTCTGAATTATCCTGCAACATAACTCTGCTGATCGTCACCATCGGTGCCACCATGCTGGTGTTGGCTGTGACTGTGACCTCCCTCTGCATCTACTTGGATCTGCCCTGGTATCTCAGGATGGTGTGCCAGTGGACCCAGACTCGGCGCAGGGCCAGGAACATACCCTTAGAAGAACTCCAAAGAAACCTCCAGTTTCATGCTTTTATTTCATATAGTGAACATGATTCTGCCTGGGTGAAAAGTGAATTGGTACCTTACCTAGAAAAAGAAGATATACAGATTTGTCTTCATGAGAGGAACTTTGTCCCTGGCAAGAGCATTGTGGAAAATATCATCAACTGCATTGAGAAGAGTTACAAGTCCATCTTTGTTTTGTCTCCCAACTTTGTCCAGAGTGAGTGGTGCCATTACGAACTCTATTTTGCCCATCACAATCTCTTTCATGAAGGATCTAATAACTTAATCCTCATCTTACTGGAACCCATTCCACAGAACAGCATTCCCAACAAGTACCACAAGCTGAAGGCTCTCATGACGCAGCGGACTTATTTGCAGTGGCCCAAGGAGAAAAGCAAACGTGGGCTCTTTTGGGCTAACATTAGAGCCGCTTTTAATATGAAATTAACACTAGTCACTGAAAACAATGATGTGAAATCTTAA

>CjacTLR6 Callithrix Jacchus Marmoset ENSCJAG00000000829 2391 ATGACCAAAGACAGCGAACCTATTGTGAAAAGCTCCCGTTTTGTTTGCCTTATGATAATAATAGTTGGAACCATCATCCAGTTCTCCGACGAAAGTGAATTTGCAGTAGACAAGTCAAAAAGAGGCCTTATTCATGTTCCAAACGACCTACCGTTGAACACCAAAGTCTTAGATATGTCTCAGAACTACATAACCGAGCTTCGGGTTTCTGATGTGAGCTTTCTGTCAGAGTTGAAAGTTTTGAGACTTTCCCATAACAGAATCCAGCTACTTGATTTGAGTGTCTTCAAGTTCAATCAGGATTTGGAATACTTGGATTTATCTCATAATCAGTTGCAAAAGATCTCTTGCTATCCTATTGTGAGTTTCAGGCATTTAGATCTCTCATTCAATGACTTCAAGGCCCTGCCCATCTGTAAGGAATTTGGCAACTTGTCGCAACTGAATTTCTTGGGATTGAGTGCTATGAGGTTACAAAGATTAGATTTGCTTCCAGTTGCTCACTTGCATCTAAGTTATATCCTTCTGGATTTAAGAAATTATTATGTGAAAGAAAATGAGACAGAAAGTCTACAAATTCTGAATGCAAAAACACTTCACCTTGTTTTTCACCCAAATAGTTTATTCTCTATCCAAGTGAGTATATCAGTTAATACTCTAGGGTGCTTACAACTGACTAATATTAAATTGAATGATGACAACTGTCAAGTTTTCATTCATTTTTTATCAGAACTCACCAGAGGTCCGACCTTACTGAATTTTACCCTCACCCACATGGAAACAACTTGGAAATGCCTGGTTAGAGTCTTTCAATACCTTTGGCCCAAACCTGTGGAATATCTCAATATTTACAATTTAACAATAATTGAAAGCATTCGCAAAGAATATTTTACTTATTCTAAAACGACGTTGAAAGCATTGAAAATAGAACATATCACAAACCAAGTTTTTCTTTATTCACAGACAGCATTGTATACAGTATTTTCCGAGATGAACATTATGATGTTAACCATTTCGGATACACCATTTATACACATGCTGTGTCCTCATGCACCAAGCACATTCAAGTTTTTGAACTTTACCCAGAATGTTTTCACAGATAGTATTTTTGAAAAATGTTCCACGTTAATTAAATTGGAGACACTTATTTTACGAAAGAATGGATTAAAAGACCTTTTCAAAGTAGGTCTCATGACTAAGGATATGCCATCTTTGGAAATGTTGGATGTTAGCTGGAATTCTCTGGAATCTGGTAGACATGAGGAAAACTGCACTTGGGGTGAGAGTATAGTGGTGTTAAATTTGTCTTCAAATATACTTACTGACTCCGTTTTCAGATGTTTACCTCCCAGGATCAAGGTACTTGATCTTCACAGCAATAAAATAAAGAGCATTCCTAAACAAGTCATAGAACTGGAAGCTTTGCAGGAACTCAATGTTGCTTTCAATTCTTTAACGGACCTTCCTGGATGTGGCAGCTTTAGCAGGCTTTCTGTGTTGATCATTGATCACAATTCAGTTTCCCACCCATCAGCTGATTTCTTCCAGAGCTGCCAGAAGATGAGGTCAGTAAATGCAGGGAACAATCCATTCCATTGTACCTGTGATCTAAGAGAATTTGTCAAAAATATAGGCCAAGTATCAAGTGAAGTAGTAGAGGGCTGGCCCGATTCTTATAAGTGTGACTACCCAGATAGTTATAGAGGAACCCCACTGAAGGACTTTCACATGTCTGACTTATCCTGCAACATAACTCTGCTGATTGTCACCATCGGGGCCACCATGCTGGTGTTGGCTGTGACTGTGACCTCCCTCTGCATCTACTTGGATCTGCCCTGGTATCTCAGGATGGTGTGCCAGTGGACCCAGACCCGACGCAGGGCCAGGAATGTACCCTTAGAAGAACTCCAAAGAAATCTCCAGTTTCATGCTTTTATTTCATATAGTGAACATGATTCTGCCTGGGTGAAAAATGAATTGGTACCTTACCTAGAGAAAGAAGGTATACAGATTTGTCTCCATGAGAGAAACTTTGTTCCTGGCAAGAGCATTGTGGAAAATATCATCAACTGCATTGAGAAGAGTTACAAGTCCATCTTTGTCTTGTCTCCCAACTTTGTCCAGAGTGAGTGGTGCCATTATGAACTCTACTTTGCCCACCACAATCTCTTCCATGAAGGATCTAATAACTTAATCCTCATCTTGCTGGAACCCATTCCACCGAACAGCATTCCCAACAAGTACCACAAGCTGAGGGCTCTCATGACACAGCGGACTTATTTGGAATGGCCCAAGGAGAAAAGCAAACATGGGCTTTTTTTGGCTAACATTAGAGCTGCTTTTCATATAAAATTAACACTAGTCGCTGAAAACAATGATGTGAAATCTTAA

>MmusTLR6 Mus musculus Mouse BC055366 2388 ATGAGCCAAGACAGAAAACCCATCGTGGGGAGTTTCCACTTTGTTTGCGCCCTGGCCTTAATAGTCGGAAGCATGACCCCGTTCTCTAATGAACTTGAGTCTATGGTAGACTATTCAAACAGGAACCTTACTCATGTCCCCAAAGACCTGCCACCAAGAACAAAAGCCCTGAGTCTGTCTCAAAACTCTATATCTGAGCTTCGGATGCCTGATATCAGCTTTCTGTCAGAGCTGAGAGTTCTGAGACTCTCCCACAACAGGATACGGAGCCTTGATTTCCATGTATTCTTGTTCAATCAGGACTTAGAATACCTGGATGTCTCACACAATCGGTTGCAAAACATCTCTTGCTGCCCTATGGCGAGCCTGAGGCATCTAGACCTCTCATTCAATGACTTTGATGTACTGCCTGTGTGTAAGGAATTTGGCAACCTGACGAAGCTGACTTTCCTGGGATTAAGTGCTGCAAAGTTCCGACAACTGGATCTGCTCCCAGTTGCTCACTTGCATCTAAGCTGCATTCTTCTGGACTTAGTGAGTTATCATATAAAAGGCGGGGAAACAGAAAGTCTTCAGATTCCCAATACCACCGTTCTCCATTTGGTCTTTCATCCAAATAGCTTGTTCTCTGTTCAAGTGAACATGTCTGTAAACGCTTTAGGACATTTACAACTGAGTAATATTAAATTGAATGATGAAAACTGTCAAAGGTTAATGACATTTTTATCAGAACTCACCAGAGGTCCAACCTTATTGAATGTGACCCTCCAGCACATAGAAACAACCTGGAAGTGCTCGGTTAAACTTTTCCAATTCTTTTGGCCCCGACCGGTGGAGTACCTCAATATTTACAACTTAACGATAACTGAGAGAATCGACAGGGAAGAATTTACTTACTCGGAGACAGCACTGAAGTCACTGATGATAGAGCACGTCAAAAACCAAGTGTTCCTCTTTTCAAAGGAGGCGCTATACTCGGTGTTTGCTGAGATGAACATCAAGATGCTCTCTATCTCAGACACCCCTTTCATCCACATGGTGTGCCCGCCATCCCCAAGCTCATTTACATTTCTGAACTTTACCCAGAATGTTTTTACTGACAGTGTTTTTCAAGGCTGTTCCACCTTAAAGAGATTGCAGACACTTATCTTACAAAGGAATGGTTTGAAGAACTTTTTTAAAGTAGCTCTCATGACTAAGAATATGTCCTCTCTGGAAACTTTGGATGTTAGTTTGAATTCTTTGAACTCTCATGCATATGACAGGACATGCGCCTGGGCTGAGAGCATATTGGTGTTGAATTTGTCTTCGAATATGCTTACAGGCTCTGTCTTCAGATGCTTACCTCCCAAGGTCAAGGTCCTTGACCTTCACAACAACAGGATAATGAGCATCCCTAAAGATGTCACCCACCTGCAGGCTTTGCAGGAACTCAATGTAGCATCCAACTCCTTAACTGACCTTCCTGGGTGTGGGGCCTTCAGCAGCCTTTCTGTGCTGGTCATCGACCATAACTCAGTTTCCCATCCCTCTGAGGATTTCTTCCAGAGCTGTCAGAATATTAGATCCCTAACAGCGGGAAACAACCCATTCCAATGCACATGTGAGCTGAGGGACTTTGTCAAGAACATAGGCTGGGTAGCAAGAGAAGTGGTGGAGGGCTGGCCTGACTCTTACAGGTGTGACTACCCAGAAAGCTCTAAGGGAACTGCACTGAGGGACTTCCACATGTCTCCACTGTCCTGTGATACTGTTCTGCTGACTATCACCATCGGGGCCACTATGCTGGTGCTGGCTGTCACTGGGGCTTTCCTCTGTCTCTACTTTGACCTGCCCTGGTATGTGAGGATGCTGTGTCAGTGGACACAGACCAGGCACAGGGCCAGGCACATCCCCTTAGAGGAACTCCAGAGAAACCTCCAGTTCCATGCTTTTGTCTCATACAGTGAGCATGATTCTGCCTGGGTGAAGAACGAATTACTACCCAACCTAGAGAAAGATGACATCCGGGTTTGCCTCCATGAGAGGAACTTTGTCCCTGGCAAGAGCATTGTGGAGAACATCATCAATTTCATTGAGAAGAGTTACAAGGCCATCTTTGTGCTGTCTCCCCACTTCATCCAGAGTGAGTGGTGCCATTATGAACTCTATTTTGCCCATCATAATCTCTTCCATGAAGGCTCTGATAACTTAATCCTCATCTTGCTGGAACCCATTCTACAGAACAACATTCCCAGTAGATACCACAAGCTGCGGGCTCTCATGGCACAGCGGACTTACTTGGAATGGCCTACTGAGAAGGGCAAACGTGGGCTGTTTTGGGCCAACCTTAGAGCTTCATTTATTATGAAGTTAGCCTTAGTCAATGAGGATGATGTGAAAACTTGA

>MdomTLR6 Monodelphis domestica Gray Short-Tailed Opossum chr5:185,788,282-185,790,693 (monDom5) 2412 ATGTGCACTACAAAACTATGGTTTATCCCAGACCCACCTATCATCAACACATTCCATTTTGTCCTCATCTTTGTATTGATCCTTGGGAGTGTAATCCAACACTCTTCGCAAAATGAATTTATAGGCAACTATTCAAACAGCCATCTCAGTCATGTTCCACATCACCTGTCACCAAAAACAACAGTCTTAGATTTATCACTAAACAATATAACTGAGATTCAGATTGAGGACTTCAAACTTCTACCAAAGCTGAGAGTTTTAATACTTTCTCATAATAGGATCCAGCACCTTAATATCAGTGTTTTTAAATTCAACCAGGATCTGGAATACTTGGATTTATCCCACAATAACTTATGGGATATTTCTTGCCATCTTCTCATGAATCTTATACATTTAGACCTCTCATTCAATGAATTTGAATTCCTTCCCATTTGTGAGGAATTTAGCAACCTGTCCCAATTAGATTTTCTGGGACTGAGTGCTAAAAGGATACAAAAATCAGACTTACTACCTGTTAGTCATTTAAATCTAAGTAACATCCTGCTAGATCTAGAAAGATTTGGGGGAGCAAAAGACAAATTGGAGACCCTTCCAGTTTTGAACACAAAGAAACTACAGATTATCTTCCCTCCAAATAGAGCATTCTTTATGCAATTGAATATATCAGTGAATAACTTAAGAAGTTTACAAATTACTAATATTAATTTAAATAATGATAAATGTTCAGTTTTCACTGATTTTCTATCAAAAGTAATTCATAATTCAAAATCTCTGAATCTTACTCTTTACCATATTGAAGACACTTGGAAATGTTTTGTGGAAATTCTTCAATTTCTTTGGCACAAGCCTATAGAATATCTTTATATCTACAACTTAACATTGGTGGGAAAAATTGGTTTTGAGTTATTCACATACAATGAAACTTTGCTGAAAGGACTGAAAATAGAATTTATCAACATCAAAGTTTATATATTTCTACAGTCAAATATATATAATATCTTTTCAAACATGAACATAAATATGTTAACGATAACTGATTCCCGAATGCTGCATATGTCTTGCCCCTTCAAGCCTAGCCCATTTCAGTATTTGAACTTTGCTAACAATCTTTTCCCAGATACTATTTTCCCAGACTGTAAAAACTTAAGCCTCTTACAAACACTTATTTTACAAAAGAACAAATTAACACACCTTTCTAAAATAGCTGGTATGACAGAAAAAATGAAGTCGCTGAAACATTTAGATTTAAGCCAGAATTCCATCAGTTATGATGAAGAGAAGAAAAACTGCAGTTGGTCTGAAAGTCTGTTGGTTCTGAATCTGTCTTCCAACGAACTCACTGAGTCTGTCTTCAGATGTTTACCTCCTAGCATCACAGTACTTGATCTCTACAGGAACAAAATAAAGAGCATCCCTAAAAACATATCCAATCTGGAAAGTTTGCAAGAATTAAATATTGCTTTTAATTTTCTAGTTGATCTTCCAGGGTGCAGTACCTTCAGTAGCCTTTCTGTATTGATCATTCAACATAATTCAGTTTCCCACCCATCTGCTCACTTCTTCAATAGCTGCCAGAATATTAAATCACTACAGGCAGAAGACAACCCATTCCTGTGTACCTGTGAACTAAGAGAATTTAGCAAAAATATGGCCACGCTCTCGAGCCAAGTGGTGAGTGGATGGCCACATTCATATAAGTGTGCCTTTCCAGATAACTATAAGGGAACACATTTAAAGGACTTTCATTTGTCTCCCTTGTCATGTAATATAGTCCTGTTGCTTATCACGATTGTTATCGTTGTGTTGGTTTTTGTGATTGTTACAACTTTCTTCTGTGTTTACTTTGACTTGCCATGGTATCTGAGGATGATATTTCAGTGGGCTCGGATGCGACGCAGAGCTAGGAACATCCCCTTAGACGAGCTTGAGAAAGAGGTCCAGTTCCATGCTTTTATATCCTACAGTGAACTGGACTCTCCCTGGGTGAAGCACGAGCTGCTTCCCAACCTTGAGCAGGAAGAGATAAGGATTTGTCTTCATGAGAGAAACTTTGTCCCTGGAAAGAGTATTGTTGAAAACATCATAAACTGTATCGAGAAAAGCCACAAATCCATCTTTGTTTTGTCTCCTAACTTCATTCAGAGTGAGTGGTGCCATTATGAGCTTTACTTTGCTCACCACAAACTTTTTCATGAACGTTCTGATAACTTAATCCTCATTTTACTGGAGCCAATTCCCCAGTACAACATTCCCGCCAAGTATCACAAGCTGAAAGCGCTCATGGCACGCAGAACCTATCTGGAATGGCCCAAGGAGAAGGGCAAACAGGCGCTTTTCTGGGCTAACATCAGAGCAGCATTTAACAGGAAATTAACATTAGTTGAAGAAGCTAATGATGTGGAGATAATTTAA

>PpygTLR6 Pongo pygmaeus Orangutan ENSPPYG00000014669 2391 ATGACCAAAGACAAAGAACCTATTGTTAAAAGCTTCCATTTTGTTTGCCTTATGATCATAATAGTTGGAACCAGAATCCAGTTCTCCGACGGAAGTGAATTTGCAGTAGACAAGTCAAAAAGAGGTCTTATTCATGTTCCAAAAGACCTGCCGCTGAAAACCAAAGTCTTAGATATGTCTCAGAACTACATAGCTGAGCTTCAGGTCTCTGACATGAGCTTTCTGTCAGAGTTGAAAGTTTTGAGACTTTCCCATAACAGAATCCAGCTACTTGATTTAAGTGTTTTCAAGTTCAACCAGGATTTAGAATATTTGGATTTATCTCATAATCAGTTGCAAAAGATATCCTGCTATCCTATTGTGAGTTTCAGGCATTTAGATCTCTCATTCAATGACTTCAAGGCCCTGCCCATCTGTAAGGAATTTGGCAACTTATCACAACTGAATTTCTTGGGATTGAGTGCTATGAAGCTGCAAAAATTAGATTTGCTGCCAGTTGCTCACTTGCATCTAAGTTATATCCTTCTGGATTTAAGAAATTATTATATAAAAGAAAATGAGACAGAAAGTCTACAAATTCTGAATGCAAACACCCTTCACCTTGTTTTTCACCCAACTAGTTTATTCTCTATCCAAGTGAACATATCAGTTAATACTTTAGGGTGCTTACAACTGACTAATATTAAACTGAATGATGACAACTGTCAAGTTTTTATTAAATTTTTATCAGAACTCACCAGAGGTCCAACTTTACTGAATTTTACCCTCAACCACATAGAAACAACTTGGAAATGCCTTGTTAGAGTCTTTCAATTTCTTTGGCCCAAACCTGTGGAATATCTCAATATTTACAATTTAACAATAATTGAAAGCATTTGTGAAGAAGATTTTACTTATTCTAAAACGACATTGAAAGCATTGAAAATAGAACATATCACGAACCAAGTTTTTCTGTTTTCACAGACAGCGTTGTACACCGTGTTTTCTGAGATGAACATTATGATGTTAACCATTTCAGATACACCTTTTATACACATGCTGTGTCCTCATGCACCAAGCACATTCAAGTTTTTGAACTTTACCCAGAACGTTTTCACAGATAGTATTTTTGAAAAATGTTCCACGTTAGTTAAATTGGAGACACTTGTCTTACAAAAGAATGGATTAAAAGACCTTTTCAAAGTAGGTCTCATGACTAAGGATATGCCTTCTTTGGAAATACTGGATGTTAGCTGGAATTCTTTGGAATCTGGTAGACATAAGGAAAACTGCACTTGGGTTGAGAGTATAGTGGTGTTAAATTTGTCTTCGAATATGCTTACTGACTCTGTTTTCAGATGTTTACCTCCCAGGATCAAGGTACTTGATCTTCATAGCAATAAAATAAAGAGCATTCCTAAACAAGTTGTAAAACTGGAAGCTTTGCAAGAACTCAATGTTGCTTTCAATTCTTTAACTGACCTTCCTGGATGTGGCAGCTTTAGCAGCCTTTCTGTATTGATCATTGATCACAATTCAGTTTCCCACCCATCAGCTGATTTCTTCCAGAGCTGCCAGAAGATGAGGTCAATAAAAGCAGGGGACAATCCATTCCAATGTACCTGTGAGCTAAGAGAATTTGTCAAAAATATAGACCAAGTATCAAGTGAAGTGGTAGAGGGCTGGCCTGATTCTTATAAGTGTGACTACCCAGAAAGTTATAGAGGAACCCTACTAAAGGACTTTCAAATGTCTGAATTATCCTGCAACATAACTCTGCTGATCATCACCATCGTTGCCACCATGCTGGTGTTGGCTGTGACTGTGACCTCCCTCTGCATCTACTTGGATCTGCCCTGGTATCTCAGGATGGTGTGCCAGTGGACCCAGACCCGGCGCAGGGCCAGGAACATACCCTTAGAAGAACTCCAAAGAAATCTCCAGTTTCATGCATTTATTTCATATAGTGAACATGATTCTGCCTGGGTGAAAAATGAATTGGTACCTTACCTAGAAAAAGAAGATATACAGATTTGTCTTCATGAGAGAAACTTTGTTCCTGGCAAGAGCATTGTGGAAAATATCATCAACTGCATTGAGAAGAGTTACAAGTCCATCTTTGTTTTGTCTCCCAACTTTGTCCAGAGTGAGTGGTGCCATTACGAACTCTATTTTGCCCATCACAATCTCTTTCATGAAGGATCTAATAACTTAATCCTCATCTTACTGGAACCCATTCCACAGAACAGCATTCCCAACAAGTACCACAAGCTGAAGGCTCTCATGACGCAGTGGACTTATTTGCAGTGGCCCAAGGAGAAAAGCAAACGTGGGCTCTTTTGGGCTAACATTAGAGCCGCTTTTAATATGAAATTAACACTAGTCACTGAAAACAATGATGTGAAATCTTAA

>OanaTLR6 Ornithorhynchus anatinus Platypus XM_001512923 2373 ATGCCTGGGACGGAAGACCAGGCCATCAGAACCTTCCTCTTCGCGGGCATCTGGACCGCGGCCCTCGGAAGTCAAACCCAGGCATCTGTCGCGAGCGACTCCTTCGCTGACTACTCCAGCCAACAGCTCGAGAAGGTACCCAGCCCCCTCTCTCCAGACCTGACAACCTTAGATGTGTCCCGAAATAACATATCCGTGCTTGAGGCCGCTGACTTCCGGTCTCTAGCGAAGCTGAGGGTTTTAGTCCTTTCTCATAATAGAATCCGCCACCTGGACACCAGTGTATTTGAATTTAATCCAGCCTTGGAGTTTGTGGATGTAGCCCACAATGAATTGGAGAGCCTCTCTTGCCACCCAGCTGTGGCCTTCAGGCACTTGGATCTCTCTTTCAACCTGTTCGAGCGGGTGCCCGTCTGCAGAGAGTTCGGCCACTGGTTGCAGCTGGATTTCCTGGGGCTGAGTGCCCGGCGGATCCGGAAAGCTGATTTGCAGCCACTGGCTGACTTGCATCTCAGAGAGGTTTTGCTCCACTTGAGGGTCCCAGAGGGGCCGGTCAGCCTGTGCTGCTTGCACACAGAGAAGCTGCACCTCGTTTTCTCTCCCTGGCTGAATCTCCCATCCCACTGGAGTCTGTCTAACAGCACTCCTGGAAGTCTAGAGATAACGAACATCCAGCTGGTCAATGGGCGGTGTTCCGATCTCACCGAGGTTTTATCAGAAGCGATGCAAGATCTGAAATTGCTCAATCTGACGCTGAACAATGTCGAAACCACTTGGGAATGCCTGGTGGCCGTCTTCCAGCTCGTTTGGCCCCGGAATGTGGAATACCTCAACATCTACAACCTGACCCTCTTTGAAGAAATCAATGAAGAAGCATTCGACACCACTGAGACGTCACTGAAAGGAGTGAAATTGGAACTCATGACCAACAAGGTTCTCTATTTCTCCCAGGATAACATCTACGGAATATTTGCAAAGATGAACATCAGCAGTCTGACAATAACCAATTCCCCTTTCATACATATGCTTTGCCCTACCGGGGCAAGCCGGTGGCTGCATCTGGACATGTCCAATAACGTGCTGACGGATACGATTTTTGAAACCTGTACCACCCTCGTTCACTTGGAAACTCTCATTTTTCGAGGAAATCAGTTAAAGAACCTGGTGCCACTGAGTCGGATGACTAAGACGATGCGGTCTCTCCAACACCTGGACCTGAGTTTGAATTTTTTGCAGTTTGATGGGGACGCGGAAAGCTGCGATTGGTCTGCGAGTCTGTCGACGTTAGATTTATCATCGAATAAGCTGACTGGATCCGTCTTCAGGTGTCTGCCCCCGCGAGTCACGGTCCTTGACCTGCACGGTAATCAGATCCGAAGCATCCCCAAGGAGGCCGTGGCCCTGCCGGCTCTGCGAGAGTTAAACGTGGCACTTAACTTCCTGGCAGACCTTCCGGACTGCGGTGCTTTCCGGAGCCTCACCGTCCTGCGCGTTGATGGCAATTCCATCCCCAGCCCATCGCCCGCCGCCTTTCGGTCATGCCAGAACCTCAGGAGGCTCTGGGCCGGAAACAATCCCTTCGCGTGTGGGTGTAATCTCAGACAGTTCATCGGCCTAGCGGCCTCTTCCTCCAATTTGCTGGAAGGTTGGCCGGATGCCTACGTCTGTGCTTCCTCAGAAGGCCATGATGGGACTCTGCTGAAGGACGTCCACCTGCCCGAATTAGCCTGCAACATCCCCCTTCTGGTGGGGACCGTGCTGGTGACCCTGTCGGTCGTGGCGGCGGCCGGGGCGGCCCTGTGCCGCTACCTGGATGTGCCCTGGTACCTGAGGATGACGTGGCAGTGGGCGCAGACCCGGCGCAGGGCCCGGCGGGCGACCCCCGTGAAACTCGAGAGGATGGCCCAGTTCCACGTGTTCGTCTCCTACAGCGAGAGGGACGCGACCTGGGTCAAGGCCGAACTGATCCCCAACCTGGAGAGTGCCGCCATAAGCGTCTGTCTGCACGAGAGGAACTTCGTCCCCGGCAAGAGTATCGTGGAGAACATCATCAGCTGCATCGAGAAGAGCCGCAAATCCATCTTCGTCCTGTCCCCCCACTTTGTCCAGAGCGAGTGGTGCCACTACGAGCTCTGCTTCGCTCACCACCGGCTCTTCCAAGAGGGTTCCGACAGCCTCATCCTCATCGTGCTGGCCCCGATCCCGCGGCACGGCATCCCCGCCCGCTATCACAAGCTTAAGTCCCTCATGGCCCGGAAGACCTATCTGGAATGGCCCCAGGAGAGGAGCAAGCAGGGCCTGTTCTGGGCCAACCTGAGGGTTGCGGTGAACCTTAAACTGACTGATCGACTGGATGATGCTCTGACTCGCTGA

>SscrTLR6 Sus scrofa Pig AB085936 2391 ATGACCAAAGACAAAAAACCTACTGTCATAAGCCTTCATTCTGTGTATGTCATGACCTTAGTATGGGGAACCCTAATCCAGTTCTCTGAAGAAAGTGAATTTGTGGTAGACAAGTCAAAAATAGGCCTTACTCGTGTTCCAAAAGACCTGCCACCCCAAACCAAAGTCTTAGATGTGTCTCAAAACTTCATAACTGAGCTTCACCTCTCTGACATCAGCTTTCTCTCGCAGCTGACGGTTTTGAGACTTTCCCAGAATAGGATGCAGTGCCTTGATATCAGTGTTTTCAAGTTCAATCAGGATTTGGAATATTTGGATTTATCTCACAATCAGTTGCAGACAATCTTGTGCCATCCCATCACCAGCCTCAAGCATTTGGACCTCTCATTCAATGACTTTGAAGCCCTGCCCATATGTAAGGAGTTTGGCAACTTGACACAACTGAATTTCTTGGGATTAAGTGCTACAAAGTTACAGCAATTAGATCTACTACCAATTGCTCACTTGCATCTAAGTTGCATCCTTCTGGATTTGGAACGTTATTACATGAAAGAAAATGAGAAAGAAAGTCTTCAAATTCTGAACACAGAGAAACTTCACCTGGTCTTTCATCCAAATAGCTTCTTCTCTGTCCAAGTAAACATATCGGTTAAGAGTGTAGGGTGTTTACAACTGGCTAATATTAAACTGGGTGATGACAACTGTCAGGTTTTCATTACATTTTTATTGGAACTCACTCAAGGGCCAACCTTACTAAATTTTACGCTCAACCATGTGGAAACAACTTGGAAATGTTTGGTTGGAATTTTTCAATTCCTTTGGCCCAAACCTGTAGAATATCTCAGTATTTACAATTTAACAATAGTTGAAAGCATTGATGAAGAAGATTTTATTTATTATGAAACAACATTGAAAGGAGTGAAAATAGAACATATTACAAAGAGAGTTTTTATTTTTTCACAGACAGCATTATACAGAGTGTTTTCCGATATGAATATCAGGATGTTAACCATAGCAGACACACATTTTATACACATGCTTTGTCCTCAGGTACCAAGCACATTTAACTTTTTGAACTTTACCCAGAATGTTTTTACAGATAGTGTTTTTCAAAATTGCAAAACTTTAGCTAGATTAGAGACACTCATCTTACAAAAGAATAAATTAGAAGACCTTTTCAAAATAAGCCTCATGACTAAGGATATGCTATCTTTGGAAATACTGGATGTTAGCTCGAATTCTTTGGAGTATGATAGACATGGTGAAAATTGCACTTGGGTTGGGAGTATAGTGGTGTTAAATTTATCTTCAAATATACTCACTGACTCTGTTTTCAGATGTTTACCTCCCAGGATCAAGGTTCTTGATCTTCACAGTAACAGAATAAGGAGCATCCCTAAGGATGTCGCCCATCTGGAAGCTCTGCAAGAACTCAATGTTGCTTCCAATTCTTTAGCTCACCTGCCTGGATGTGGTTCCTTTAGCAGCCTTTCCATTCTGTCCATTGACTATAATTCAATTTCCAACCCATCAGCTGACTTCTTCCAGAGCTGCCAGAAGATTAGGTCCCTCAAAGCAGGGAACAATCCATTCCAATGTACATGTGAGCTAAGAGACTTCATCCAAAGTCTAGGTCAAGTATCGAGTGACGTGGTAGAGAGTTGGCCTGATTCTTACGAGTGTGAGTATCCAGAAAGTTATAAGGGGACTCTGCTCAAGGACTTCCGTGTATCTGAATTATCCTGCAACACAGCTCTGCTGATTGTCACCATCGGAGTCACTGGGCTGGCATTGGCTCTTACCATGACCGGCCTCTGTGTCTACTTTGATCTGCCCTGGTATCTCAGGATGCTGTGTCAGTGGACCCAGACTCGGCGCAGGGCTAGGAATGTACCCTTAGAAGAACTCCAAAGAACTCTCCAGTTCCATGCCTTCATTTCATATAGTGAACACGATTCTGCCTGGGTAAAAAATGAACTGGTACCTTGTCTAGAAAAAGAAGGTATAAAGATTTGTCTCCATGAGAGAAACTTTGTTCCTGGCAAGAGCATCATGGAAAATATCATAAACTGCATTGAGAAAAGCTACAAGTCCATCTTTGTTTTGTCTCCCAACTTTGTCCAGAGCGAGTGGTGCCACTATGAACTCTACTTTGCCCACCACAACCTCTTCCATGAAGGGTCTGATAACTTAATCCTGATCTTGCTGGATCCCATTCCACAGAACAGCATTCCTGGCAAGTATCACAAACTCAAAGCTCTCATGGCACAGCGAACTTATTTGGAATGGCCCAAGGAGAAGAGCAAACATGGACCTTTTTGGGCTAATATTAGAGCTGCTTTCAATATTAAATTAAAACTAGTCGCTGAAGAGGATGATGTGAAAACTTGA

>RnorTLR6 Rattus norvegicus Rat NM_207604 2388 ATGTCCCAAGACAGAGAACCCATCGTGGAGAGTTTCCATTTTGTTTGCACCCTGGCCTTAATAGTCGGAAGCATGACCCAGTTCTCTGATGAACTTGAGTCTGTAGTAGACTATTCAAACAAGAACCTTACTCATGTCCCAAAAGACCTGTCACCAAGCACAAAATCCTTGAGTCTGTCTCAAAACTCCATATCTGATCTTCAGATGTCTGATATCAGCTTTCTGTCAGAGCTGAGAGTTCTGAGACTCTCCCACAACAGAATACGGAGACTTGACTTTGGTGTGTTCTTGCTCAATCGGGACTTAGAATACCTGGATGTCTCTCACAATCAGTTACAAAACATCTCTTGCTGTCCTATGGTGAACTTGAAACATCTAGACCTCTCATTCAATGATTTTGAAGTGCTGCCCGTGTATAAGGAATTTGGCAACTTGAGGAAGCTGAGTTTCTTGGGATTAAGTGCTGCAAAGTTCCGACAACTGGATCTGCTCCCAATTTCTCACTTGCACCTGAGCTGTGTTCTTCTGGACTTGGTGAATTATCAGATAAAAGATGGTGAAACAGAAAGTCTTCAGGTTCCAAATACCAACGTTCTCCATTTGGTCTTTCATCCGAATAGCCTGTTCTCTGTGCAAGTGAACATATCTGTAAATGCTTTAGGATGCTTACAACTGAGTAATATTAAATTGAATGATGAAAACTGTCAAAGCTTAATTATATTTTTATCAGAACTCACCAGAGGTCCAACCTTATTGAATCTGACCCTCCAGCACATAGAAACAAACTGGAAGTGCTTTGTTAGACTTTTACAATTCCTTTGGCCCAGACCTGTGGAGTATCTCAATATTTACAACTTAACGATAACTGAGAGCATAAGCAGGGAGACATTTATTTACGTGGAGACAGTGTTGAAGTCACTGAAGATAGAGCATGTCACAAACCAAGTGTTCCTCTTTGTGAAGGATGCACTATATTCTGTGTTTGCAGAAATGAACATCAGGATGCTCACACTGTCAGACACGCCATTCATCCACATGGTGTGCCCTGAGTTCCCAAGCACATTTGCATTTCTGAACTTTACCCAGAACGTTTTTACTGACAGCATTTTTCAAGGCTGTTCCACCTTGAAGAGACTGGAGACACTTATCTTGCAAAGGAATGGTTTAAAGAACCTTTTTAAGGTAGCTCTCATGACCAAGACTATGTCCTCTCTGGAAACATTGGATGTCAGTTTGAATTCTTTGAACTCTCATGTGTATGACAGAACATGTGCTTGGGCCGAGAGCATACGGGTGTTGAATTTGTCTTCGAATGTACTTTCGGACTCTGTCTTCAGGTGCTTACCTCCCAAGGTCAAGGTCCTTGACCTTCACAACAACAGGATAGTGAGCATCCCTAAAGATGTCACCCACCTGCAAGCTTTGCAGGAACTCAATGTCGCATCCAATTTTTTAACTGACCTTCCTGGATGTGGAGCCTTCAGTAGCCTTTCTGTGCTGGTCATCGACCATAACTCAGTTTCCCACCCCTCCTCTGATTTCTTCCAGAGCTGTCAGAATATCAGGTCCATAACAGCGGGGAACAACCCATTCCGATGCACATGTGAGCTGAGGGAGTTTGTCAAAAACATAGGTCAGGCATCAAGAGAAGTGGTGGAGGGCTGGCCTGACTCTTACAGATGTGATTACCCAGACAGCATTAAGGGAACCCCACTGCAGGACTTCCACATGTCTCCACTGTCCTGCGATACAATTCTACTGACTGTCACCATTGGGGCCACTCTGCTGCTACTGGCTGCCATTGGGGCTTCCCTCTGTCTCTACTTTGATCTGCCCTGGTATCTCAGGATGCTATGGCAGTGGACACAGACCAGGCACAGGGCCAGGAACATCCCCTTAGAGGAACTGCAGAGGAACCTCCAGTTCCATGCTTTTGTCTCATACAGTGAGCATGATTCTGCCTGGGTGAAGAATGAATTACTACCAAACCTAGAGAAAGATGACATTCGGGTTTGCCTCCATGAGAGAAACTTTGTCCCTGGCAAGAGCATTGTGGAGAACATCATACACTTCATTGAGAAGAGTTACAAGTCCATCTTTGTGCTGTCTCCCCACTTCATCCAGAGTGAGTGGTGCCATTATGAACTCTACTTTGCCCATCACAATCTCTTCCACGAAGGGTCTGATAACTTAATCCTGATCTTGCTGGAACCCATCCAACAGAACAACATTCCCAGTAGATACCACAAGCTGAGGGCTCTCATGGCACAGCGGACTTACTTGGAATGGCCTATTGAGAAGGGTAAACGTGGGCTGTTTTGGGCCAACCTTAGAGCTTCTTTTATTATGAAGCTAGCCTTAGTTAATGAGAATGATGTGAAAACTTGA

>MmulTLR6 Macaca mulatta Rhesus Monkey XM_001089296 2391 ATGACCAAAGACAAAGAACCTGTTGTTAAAAGCTTCCATTTTGTTTGCCTTATGATCATAATAGTTGGAACTAGAATCCAGTTCTCTGACGGAAGTGAATTTGCAGTAGACAAGTCAAAAAGAGGTCTTACTCATGTTCCAAAAGACCTACCGCCGAAAACCAAAGTCTTAGATATGTCTCACAACTACATAGCTGAGCTTCAGGTCTCTGACATAAGCTTTCTGTCAGAGTTGAAAGTTTTGAGACTTTCCCATAACAAAATCCAGCTACTTGATTTAAGTGTTTTCAAGTTCAACCAGGACTTGGAATATTTGGATTTATCTCATAATCAGTTGCAAAAGATATCCTGCCATCCTATTATGAGTTTCAGGCATTTAGATCTCTCATTCAATGACTTCGAGGCCCTGCCCATCTGTAAGCAATTTGGCAACTTGTCACAACTGAATTTCTTGGGGTTGAGTGCTATGAAGTTACAAAAATTAGATTTACTGCCAATTGCTCACTTGCATCTAAGTTACATCCTTCTGGATTTAAGAAATTATTATATAAAAGAAAATGAGACAGAAAGTCTACAAATTCTGAATGCAAAAACACTTCACCTTGTTTTTCACCCAACTAGTTTATTCTCTATCCAAGTGAACATATCAGTTAATACTTTAGGGTGCTTACAACTGACTAATATTAAATTGAATGATGACAACTGTCAAGTTTTCATTAAATTTTTATTAGAACTCACTAGAGGTCCAACCTTGCTGAATTTTACCCTCAATCACATAGAAACAACTTGGAAATGCCTGGTGAGAGTCTTTCAATTTCTTTGGCCCAAACCTGTGGAATATCTCAATATTTACAATTTAACAATAATTGAAAGCATTCATGAAGAAGATTTTACTTATTCTAAAACGACATTGAAAGCATTGAAAATAGAACATATCACGAACCAAGTTTTTATCTTTTCGCAGACAGCATTGTACACCGTGTTTTCTGAGATGAACATTATGATGTTAACCATTTCAGATACACCTTTTATACACATGCTGTGTCCTCGTGCACCAAGCACATTCAAGTTTTTGAACTTTACCCAGAATGTTTTCACAGATAGTATTTTTGAAAAATGTTCCACGTTAGTTAAATTGGAGACACTTATCTTACAAAAGAATGGATTAAAAGACCTTTTCAAAGTAGGTCTCATGACTAAGGATATGCCATCTTTGGAAATACTGGATGTTAGCTGGAATTCTTTGGAATCTGGTAGACATAGGGAAAACTGCACTTGGGTTGAGAGTATAGTGGTGTTAAATTTGTCTTCAAATATACTTACTGACTCCGTTTTCAGATGTTTACCTCCCAGGATCAAGGTACTTGATCTTCACAACAATAAAATAAAGAGCATTCCTAAACAAGTCGTAAAACTGGAAGCTTTGCAAGAACTCAATGTTGCTTTCAATTCTTTAACTGACCTTCCTGGATGTGGCAGCTTTAGCAGCCTTTCTGTATTGATCATTGATCACAATTCAGTTTCCCACCCATCAGCTGATTTCTTCCAGAGCTGCCAGAAGATGAGGTCAATAAAAGCAGGGAACAATCCATTCCAGTGTACCTGTGAGCTAAGAGAATTTGTCAAAAATATAGAGCAAGTATCAAGTGAAGTGGTAGAGGGCTGGCCTGATTCTTATAAGTGTGACTACCCAGAAAGTTATAGAGGAACCCCACTAAAGGACTTTCACATGTCTGAATTATCCTGCAACATAACTCTGCTGATCATCACCATCGGTGCCACCATGCTGGTGTTGGCTGTGACTGTGACCTTCCTCTGCATCTACTTGGATCTGCCCTGGTATCTCAGGATGGTGTGCCAGTGGACCCAGACCCGGCACAGGGCCAGGAATGTACCCTTAGAAGAACTCCAAAGAAATCTCCAGTTTCATGCATTTATTTCATATAGTGAACATGATTCTGCCTGGGTGAAAAATGAATTGGTACCTTACCTAGAGAAAGAAGGTATGCAGGTTTGCCTTCATGAGAGAAACTTTGTTCCTGGTAAGAGCATTGTGGAAAATATCATCAACTGCATTGAGAAGAGTTACAAGTCCATCTTTGTTTTGTCTCCCAACTTTGTCCAGAGTGAGTGGTGCCATTATGAACTCTACTTTGCCCATCACAATCTCTTTCATGAAGGATCTAATAACCTAATCCTCATCTTACTGGAACCCATTCCACAGAATAGCATTCCCAACAAGTACCACAAGCTGAGGGCTCTGATGACTCAGAGGACTTATTTGCAATGGCCCAAGGAGAAAAGCAAACGTGGGCTCTTTTGGGCTAACATTAGAGCCACTTTTAATGTGAAATTAACACTAGTCACTGAAAACAATGATGTGAAATCTTAA

>DnovTLR10 Dasypus novemcinctus Nine-Banded Armadillo ENSDNOG00000009949 2397 ATGATACTGATCAGAAACATTTGCATATTTTGTAGTATTGTTCTGTCAGTGCAGGGTGGGAACCCAAAACTGCCAGAAGAAAGTGAATTGACTACCAACTACTCCAGTATGTCTCTGAGAAAGGTCCCTGCAGATTTGACTCCAACCACAACCACGCTGGATTTATCCTACAATCTTCTTTTTCAGCTTCAGAGTTCAGATTTTCGTTCTGTCTCCAAACTGAAAGTTCTGATTCTGTGCCATAACAGAATCCAACAGCTGGCTATCAAGGTCTTCGAATTCAATAAGGAGTTAAGATATTTAGATTTGTCTTACAACAGACTGAAAGCTGTAACTTGCTATTCACTGGCAGGTCTCAGACATTTAGACCTTTCTTTCAATGACTTTGACACTGTGCCTATCTGTGAGGAGATTGGCAGCCTGTCAAAACTGGAAATCCTAGGCTTGAGTGGGGCAAAAATACGAAAGTCAGACTTCCAGAAAATTGCTCATTTGCATCTAAATACTGTGTTCTTAGGCTTGAAAACTCTTTCTCATTATGAAGAAGGCAGTCTGCCCCTCCTAAACACAACAAAACTTCACATTTTTGTACCAATGGACACAAATTTCTGGGTTCTTTTGCGTGATGGAATGAAGACTGCAAAAATATTAGAAATGACAAATATAGATGGCAAAAGCCAATTTGCAAGTTATGAAACTCAACCCAGTCTTATTTTAGAAAATGCTAAAACATCTACTTTGTTACTTAATGAAGTTGATTTACTCTGGGATGACCTTTTCCTCATCTTCCAGCTTGTTTGGCATTCATCAGTGGAATACTTCCAGATCCAAAATGTGACTTTTGGAGGTAAGGTTTACCTCAACCACAATTCATTTAACTACTCAAATACTGTAATGAGAACAATAAAATTGGAGCATGTACACTTCAGAGTTTTTCATATTCCACAAGATAGAGTTTATCTGCTTTTTACTAAAATGGATTTGGAAAACCTGACAATATCAGATGCACAAATGCCACACATGCTTTTTCCTGATTACCCTACAAGATTCCAATATTTAAATTTTGCAAGCAATATCTTAACAGATGACCTGTTTAAAAATCCTATCCAATTGCCTCATTTGAAAACGCTCATTTTGACGGGTAATAAACTGGAAACACTTTCCTTAGTGAGTTCCTTTGCTAACAGCACACCCTTGAAGCACTTAGATCTGAGCCAAAACCTATTGCAACATGAAAATGATGAAAATTGTTCCTGGCCAGAAACTGTGGTCANNNNNNNNNNNNNNNNNNNNNNNNNNNNNNNNNNNGTTTTCAGGTGCTTGCCCCCAAATATTCAAATACTTGACTTGAATAATAACAAAATTCAAACTGTCCCTAAAGAGATTGCTCATCTGACGTCTTTACAAGAACTACACATTGCATTTAATTTTCTAACTGATCTCCCAGGGTGCAGTCACTTCAGAAAACTCTCAATTCTGGACATTGAAATGAACTTAATTCTCAGCCCTTCTCTAGATTTTTTTCAGAATTGCCAGCAAGTGAGAACTCTCAATGCAGGAAGAAATCCATTCCGGTGTACCTGTGAATTAAGAGACTTCATTCAGCTTGAAAAAAATTCAGAGGGCATGATGGTTGGATGGTCAGATTCATACATCTGTGAATATCCTTTGAATCTAAGGGGGACTCAGTTAAAGGATGTTCATCTACCCGAGTTATCGTGCAACACAGCTCTGTTGATTGTCACCATCGTGGTCATCATGCTGACTCTGGGGGTGGCAGTGGCTTTCTGCTGTGTCCACTTTGATTTCCTGTGGTATCTCAGGATGCTGTGTCAATGGGCAAAGACATGGCATGAAGTTAGGATGACAACTCAAGAACAGCTAAAGAGAAATATCCAATTCCATGCATTTATTTCATACAGTGAACACAATTCTCTCTGGGTGAAGAATGAATTGATTCCCAACCTAGAGAAAGATGATGGTTCTGTGTCGATTTGCCTTCATGAGAGACACTTTGATCCTGGCAAGAGCATTGCTGAAAATATCAGAAGCTGCATTGAAAAAAGCTATAAGTCCATCTTTGTTTTGTCTCCTAGCTTTGTCCAGAGTGAGTGGTGCCATTATGATGCCTGCTTTGCCCACTGCAATCTCTCCCATGAAAATCCCAATTACTTAATCTTCATTTTGCTGGAACCCATTCCACTCTACTGCATTCCCACCAAGTATCATAAGCTGAGAGCGCTCATGGAAAAGAAAACATACTTGGAATGGCCCAAGGATAGGCGTAAATGTAGGCTGTTTTGGGCAAACCTTCAGGCTGCTGTTAATGTTAATTTATCAGAAACCAGAGAGATGTTGGAACTACCGACATTCACAGAGCTCGATGAAGAGTCCCGAGGTTCTGCCATGTCTCTGATAAGAACAGACTACCTTTAA

>FcatTLR10 Felis catus Cat chrB1:190,319,954-190,322,286 (felCat4) 2260 ACCAACTGCTCCAACATGTCCCTTAGAAAGGTTCATGCAGACTTGACCCCAACCACAACCACACTGGATTTATCCTACAACCTCCTTTCTCAGCTTCAGAGTTCAGATTTTCGTTCTGTCTCTAAACTGAAAGTTTTGATTCTGTGCCACAACAGAATCCAAGAGCTGGATATCAAGACCTTTGAATTCAACAGAGAGTTAAGATATTTAGATTTGTCTTACAACAGATTGAAGATTGTAACTTGGTATTCACTGGCAGGTCTCAGACATTTAGATCTTTCTTTCAATGACTTTGACAGTGTGCCTATCCACGAGGAGGCTGGCAACATGTCACATCTGGAAATCCTGGGTTTGAGTGGGGCAAAAATACGAAAATCAGATTTCCAGAAAATTGCTCATTTGCATCTAAATACAGTCTTCTTAGGATTAAGAAGTCTTTCTTATTATGAAGAAGGTAACCTGCCCATCTTAAACACAACAAAACTTCATATTGTTTTACCAATGAACACAAATTTCTGGGTTCTTTTGCGTGATGGAATCAAGACTTCAAAAATACTAGAAATGACAAACATAGATGGCAAAAGCCAATTTTCAAGTTATGAAACTCAACAAAATCTTACTTTAGCGAATTCCAAGACATCTATTCTATTGCTTAATAAAGTTGATTTACTCTGGGACTACCTTCTCCTCATCTTCCAGTTTGTTTGGCATACATCAGTAGAATGCTTCCAAGTCCAACATGTGACTTTTGGAGGCAAGGTTTATCTTGACCATAATTCATTTGATTACTCAAATACTGCAATGAGAGCTATAAAATTGGAGCACATACAGTTCAGAATTTTTTATATTCCACAGGAAAGGGTCTACTTGCTTTTTACCAAAATGGATATAGAAAACCTGACTATATCAGATGCACAAATGCCACACATGCGGTTTCCTAATTATCCCACAAGATTCAAACATTTAAATTTTGCTGATAATATCTTAACAGATGACCTGTTTAAGCAACCTATCCAATTGCCTCATTTGAAAACTTTAATTTTGAAGGGCAATAAATTGGAGACACTTTCTTTAGTGAGTTTCTTTGCCAACAACACATCCTTGAAGCACTTAGATCTCAGCCAGAATCTGTTACAATATGAAAATGATGAAAATTGCTTTTGGCCAGAAACCTTGATCACTATGAACCTGTCATCCAACAAATTTGCTGATTCTGTTTTCAGGTGCTTGCCCAGAAGTATTCAAATACTTGACCTGAATAATAACAAGATTCAAACTGTCCCTAAAGAGATTATTCATCTGAAGTCTTTGCGAGAACTAAATATCGCATTTAACTTTCTAACTGATCTTCCTGGGTGCAGTCATTTCAGAAAACTCTCAATTCTGAACATTGAAATGAACTTAATTCTCAGCCCATCTCTGGATTTTTTCCAGAGCTGTCAGGAAGTTAAGACTCTGAATGCAGGAAGAAACCCATTCCGGTGTACCTGTGAATTAAGAGATTTTATTCAGCTGGAAAAATATTCACAGGGCATGATGATTGGATGGTCAGATTCATATATCTGTGAATACCCTTTGAATCTAAAGGGGACTCGGTTAAAGGATGTTCATCTTCCTGAATTATCTTGCAACACAGCTCTGTTGATTGTCACCATTGTGGTTATCATGCTAGTTCTGGGGACTGCTATGGCCTTCTGCTGCCTCTACTTTGATCTGCCCTGGTATCTCAGGATGCTAGGTCAGTGGACACAGACATTGCAGAGGATTAGGAAGACAACCCAAAGAACAACTTCAAGAAGAAATGTCCAGTTCTATGTGTTTATTTCATACAATAAACATGACTCTACCTGGGTGAAGCATGAATTAATTCCCAATCTAGAGAAAAGAGAGAACTTTGATTTCCTTCATGAAGAAACTTTTGATCCTGGTAAGAGCATACCTGGAAATATCACAAACTGCATAGAAAAAACTNNNNNNNNTCTCTTCCATGAAAATTCTGATTACATAATTTTTATCTTGCTAGAACCCATTCCACTCTACTGTATTCCTACCAGGTATCCTAAGCTGAAAGCTCTTATGGAAAAGAAAGCATACTTGGAATGGCCCAAAGATAGGCGTAAATGTGGACTTTTTTGGGCAAAACTTAGAGCTTCTATTAATGTTAATTTATTAGAAACCAGAGAGATGTGTGAACTACAGACATTTGTGGAGCTGAATGAAGAGTCTCAAGGTTCTGTAATCTCTCTGATAAGAACAGACTGCCTATAA

>BtauTLR10 Bos Taurus Cow BC123541 2439 ATGAGATATATCAGAAGCATTTACATATTTTGTAGTATTGTTACGTCTGTGCGTAGTGGGGCTTCAGAGCTGCCCGAAGAAAGGGAATTGACTACCAACTTCTCCAGCATGTCTTTAACAAAGGTTCCTGAAGGCTTGACCCCAATTACAACCACATTGGATTTGTCTTATAATCTCCTTTTTCAACTCCAGCATTCAGATTTCCGTTCTCTCTCTAAACTGAAAGTTTTGATTCTATGCCACAACAGAATCCAAGAGCTGGATATCAAGACCTTTGAATTCAACAAGGAGTTAAGCTACTTAGATGTGTCTAACAACAGACTGAAGAGTGTAACCTGGTTCTCACTGGCAGGTCTCAGACATTTAGATCTGTCCTTCAATGACTTTGACACTCTGCCTATCAGCGTAGAAACTGGCAACATGTCACACCTGGAAACCCTAGGTTTGAGTGGGGCAAAAATACAAAAATCAGATTTTCAGAAAATTGCTCATTTGCAGCTAAATACCGTCTTATTAGGATTGAGAACTCTATCTCATTATGAAGAAGGTAGCTTGCCCATCTTAAACACAACAAGACTTCACATTGTGTTACCAGTGAACACAAATTTCTGGGTTCTTTTGCATGATGGAATCAAAACTTCAAAAATATTAGAAGTCATCAATATAGATTTGCAAAAGAGCCAGTTTACGAGTTATGAATCTCAACAAATTCCTATTTTAGAAAATGCCAAGACATCCATTCTGTTACTTAATAAAGTTGACTTATCCTGGGACGATCTTTTCCTCATCTTCCAATTGGTTTGGCATACATCAGTAGAGTACTTTCAGATTCAACATGTGACCTTTGGAGGTAAGGTTTATCTTGACCACAATTCATTTGACTACTCAAATACTGTAATGAGAACTATAAAACTGGAACATGTACATTTTAGAATTTTTAATATCCCCCAGGAGAGCATCTACTTGCTTTTTACCAAAATGGATATAGAAAACCTGACAATATCAGATGCACAAATGCCTCACATGCTGTTCCCTATGTACCCTACAAGATTCCAATATTTAAATTTCGCTAATAATATCTTAACAGATGATGTGTTTAAAAAATCTATCCAATTGCCTCATTTGAAAACTCTCATCTTGAAGGACAATAAATTGGAGACACTTTCCTTAGTGAGTCACTTTGCCAGCAACACATCCCTGAGGCACTTAGATCTGAGTGAAAATCTGTTACAACATGAAAATGATGAAAATTGCTTGTGGCCAGAAACCTTGGTCACCATGAACTTGTCATTCAACAAATTTGCTGATTCTGTTTTTGGGTGCTTGCCTAGAAACATTCAAATACTGGACCTGAATAGTAACAAAATTCAAACTGTCCCTAAAGCGATTACTCACCTGACATCTTTGCGAGAGTTAAATCTTGCATTTAATTTTCTAACTGATCTTCCTGGGTGCAGTCATTTCAGAAGACTCTTAGTTCTGAACGTTGAAATGAACTTAATTCTCAGCTCATCTCTGGATTTTTTCCAGAGCTGCCAGGAAGTTAAAACTCTAAATGCAGGAAGAAATCCATTCCGATGTACTTGTGAATTAAGAGATTTCATTCAGCTTGGAAAATACTCAGAAGGCATGATGGTTGGATGGTCAGATTCATACATCTGTGAATACCCTTTGAATCTAAAGGGGACTCAGTTAAAGGATGTTCATCTTCCAGAAATATCTTGCAACACAGGTTTGCTGATTGTCACCATTGTGGTTGTCATGCTCGTTCTGGGGATGGCTGTGGCCTTCTGCTGCCTCCACTTTGATTTGCCCTGGTATCTCAGGATGCTGCATCAATGGACTCAGACATGGCTCAGGGTTAGGAAGACAACTCAGGAACAACTCAAAAGAAGTGTCCAATTCCATGTGTTTATTTCATACAGTGAACATGATTCTGCCTGGGTGAAGTATGAATTGATTCCCAGTCTAGAGAAAGAAGATGGTTCTGTTCTGATTTGCCTTCATGAGGGAAACTCTGACCCTGGCAAGAGCATGACTGAAGATACCATAAACTGCATTGAGAAAAGTTACAAGTCCATCTTTGTTTTGTCTCCCAGCTTTGTCCAGACTGAATGGTGCCATTATGAACCCTACTTTGCCCACCACAATCTCTTCCATGAAAGTCTTGATTACATAATTCTTATCTTACTGGAACCCATTCCACTCTACTGCATCCCTACCAGATATCCTGAGCTGAAAGCTCTCATGGAAAAGAAAGCATACTTGGAATGGCCCAAGGATAGGCGTAAATGTGGCCTTTTTTGGGCAAACCTTCGAGCTGCTCTTCATGTTAATTTATTAGACACCAGAGGGACATGTGAACTACAGACATTCACAGAACTGAATGAAGGGTTTGGAGGTTCTGCAATCTCTCTGATCAGAACAGACTGCCTGTAG

>PtroTLR10 Pan troglodytes Chimpanzee XM_001138435 2436 ATGAGACTCATCAGAAACATTTACATATTTTGTAGTATTGTTATGACAGTAGAGGGTGATGCTCCAGAGCTGCCAGAAGAAAGGGAACTGATGACCAACTGCTCCAACATGTCTCTAAGAAAGGTTCCCGCAGACTTGACCCCAGCCACAACAACACTGGATTTATCCTATAACCTCCTTTTTCAACTCCAGAGTTCAGATTTTCATTCTGTCTCCAAACTGAGAGTTTTGATTCTATGCCATAACAGAATTCAACAGCTGGATCTCAAAACCTTTGAATTCAACAAGGAGTTAAGATATTTAGATTTGTCTAATAACAGACTGAAGAGTGTAACTTGGTATTTACTGGCAGGTCTCAGGTATTTAGATCTTTCTTTTAATGACTTTGACACCATGCCTATCTGTGAGGAAGCTGGCAACATGTCACACCTGGAAATCCTAGGTTTGAGTGGGGCAAAAATACAAAAATCAGATTTCCAGAAAATTGCTCATCTGCATCTAAATACTGTCTTCTTAGGATTCAGAACTCTTTCTCATTATGAAGAAGGTAGCCTGCCCATCTTAAACACAACAAAACTGCACATTGTTTTACCAATGGACACAAATTTCTGGGTTCTTTTGCGTGATGGAATCAAGACTTCAAAAATATTAGAAATGACAAATATAGATGGCAAAAGCCAATTTGTAAGTTATGAAATGCAACGAAATCTTAGTTTAGAAAATGCTAAGACATCGATTCTATTACTTAATAAAGTTGATTTACTCTGGGACGACCTTTTCCTTATCTTACAATTTGTTTGGCATACATCAGTGGAACACTTTCAGATCCGAAATGTGACTTTTGGTGGTAAGGCTTATCTTGACCACAATTCATTTGACTACTCAAATACTGTAATGAGAACTATAAAATTGGAGCATGTACATTTCAGAGTGTTTTACATTCAACAGGATAAAATCTATTTGCTTTTGACCAAAATGGACATAGAAAACCTGACAATATCAAATGCACAAATGCCACACATGCTTTTCCCTAATTATCCTACGAAATTCCAATATTTAAATTTTGCCAATAATATCTTAACAGACGAGTTGTTTAAAAGAACTATCCAACTGCCTCACTTGAAAACTCTCATTTTGAATGGCAATAAACTGGAGACACTTTCTTTAGTGAGTTGCTTTGCTAACAACACACCCTTGGAACACTTGGATCTGAGTCAAAATCTATTACAACATAAAAATGATGAAAATTGCTCATGGCCAGAAACTGTGGTCAATATGAATCTGTCATACAATAAATTGTCTGATTCTGTCTTCAGGTGCTTGCCCAAAAGTATTCAAATACTTGACCTAAATAATAACAAAATCCAAACTGTACCTAAAGAGACTATTCATCTGATGGCCTTACGAGAACTAAATATTGCATTTAACTTTCTAACTGATCTCCCTGGGTGCAGTCATTTCAGTAGACTTTCAATTCTGAACATTGAAATGAACTTCATTCTCAGCCCATCTCTGGATTTTGTTCAGAGCTGCCAAGAAGTTAAGACTCTAAATGCGGGAAGAAATCCATTCCGGTGTACCTGTGAATTAAAAAATTTCATTCAGCTTGAAACATATTCAGAGGTCATGATGGTTGGATGGTCAGATTCATACACCTGTGAATACCCTTTAAACCTAAGGGGAACTCGGTTAAAAGACGTTCATCTCCACGAATTATCTTGCAACACAGCTCTGTTGATTGTCACCATTGTGGTTATTATGCTAGTTCTGGGGTTGGCTGTGGCCTTCTGCTGTCTCCACTTTGATCTGCCCTGGTATCTCAGGATGCTAGGTCAATGCACACAAACATGGCACAGGGTTAGGAAAACAACCCAAGAACAACTCAAGAGAAATGTCCGATTCCACGCATTTATTTCATACAGTGAACATGATTCTCTGTGGGTGAAGAATGAATTGATCCCCAATCTAGAGAAGGAAGATGGTTCTATCTTGATTTGCCTTTATGAAAGCTACTTTGACCCTGGCAAAAGCATTAGTGAAAATATTGTAAGCTTCATTGAGAAAAGCTATAAGTCCATCTTTGTTTTGTCTCCCAACTTTGTCCAGAATGAGTGGTGCCATTATGAATTCTACTTTGCCCACCACAATCTCTTCCATGAAAATTCTGATCATATAATTCTTATCTTACTGGAACCCATTCCATTCTACTGCATTCCCACCAGGTATCATAAACTGAAAGCTCTCCTGGAAAAAAAAGCATACTTGGAATGGCCCAAGGATAGGCGTAAATGTGGGCTTTTCTGGGCAAACCTTCGAGCTGCTATTAATGTTAATGTATCAGCCACCAGAGAAATGTATGAACTGCAGACATTCACAGAGTTAAATGAAGAGTCTCGAGGTTCTACAATCTCTCTGATGAGAACAGACTGCCTATAA

>CfamTLR10 Canis familiaris Dog XM_545953 2424 ATGAAACGTATCAGAAGCATTTATATATTTTGTAGTATTGCTATATCAGTGCAGGGCTGGGCTTCGAAGCTGCCAGAAGAAAGGGAGTTGACAACCAATTGCTCCAACATGTCCCTAAGAAAAATTCCTGCAGACTTGACCCCAACCACAACCACACTGGATTTATCCTACAACCTCCTTTCTCAACTCCAGAGTTCAGATTTTCGTTCTGTCTCTAAACTGAAGGTTTTGATTCTCTGCCATAACAGAATTCAAGAGCTGAATATCAAGATCTTTGAATTTAACAGAGAGTTAAGATATTTAGATTTGTCTTACAACAGATTGAAGATTGTAACTTGGTATTCACTGGCAGGTCTCAGGCATTTGGATCTTTCTTTCAATGACTTTGACACCGTGCCTATCTGTGAGGAGACTGGCAACATGTCACATTTGGAAATCCTAGGTTTGAGTGGGGCAAAAATACAAAAATCAAATTTCCAGAAAATTGCTCATTTGCATCTAAAAACTGTCTTCTTAGGATTGAGAAGTCTTTCTCACTATGAAGAAGGTAGCCTGCCCATCTTAAACACAACAAAACTTCACATTGTTTTACCAATGAACACAAATTTCTGGGTTCTTTTGCATGATGGAATCAAGACTTCAAAAATACTAGAAATGACAAATATAGATGGCAAAAGCCAATTTGCAAGTTATGGAACTCAAAAAAATCTTACTTTAGAGAATTCCAAGACATCTATTTTATTACTTAATAAAGTTGATTTACTCTGGGATGACCTTCTCCTCATCTTCCAATTTGTTTGGCATACGTCAGTAGAATGCTTCCAAATCCAACATCTGACTTTTGGAGGCAAGGTTTATCTCGACCACTATTCATTTGATTACTCAAACACTGTAATGAGAACTATAAAATTAGAGCATGTACAGTTCAGAATTTTTTATATTCCACAGGAGAGAGTCTACTTGCTCTTTACCAAAATGGATATAGAAAACCTGACTATATCAGATGCACAAATGCCATATATGCTGTTTCCTATATATCCTACAAGATTCCAATATTTAAATTTCGCTAATAATATCTTAACGGATGACCTGTTCAAGCAACCTATCCAATTACCTCATTTGAAAACTCTCATTTTGAAGGGCAATAAATTGGAGACGTTTTCTTTAGTGAGTTTCTTTGCCAACAACACATCCTTGAAGCACTTAGATCTGAGCCAGAATCTGTTACAACATGAAAATGGTGAAAATTGCTTTTGGCCAGAAACCTTGATCACTATGAACCTGTCATCCAACAAATTTGCTGATTCTGTTTTCAGGTGCTTGCCCAGAAATATTCAAATACTTGACTTGAATAATAACAAAATTCAAACTGTCCCTAAAGACATTATTCATCTGAAGTCTTTGCAAGAGTTAAATCTTGCATTTAATTTTCTAACTGATCTTCCAGGATGCAGTCATTTCAGAAAACTTTCAATTCTGAACATTGAAATGAACTTAATTCTCAGCCCATCTCTGGATTTTTTTCAGAGCTGTCAGGAAGTTAAGATTCTGAATGCAGGAAGAAATCCATTCCGGTGTACTTGTGAATTAAGAGATTTCATTCAGCTTGAAGAATATTCAGAGGGCATGATGATTGGATGGTTAGATTCATATATCTGTGAATATCCTTTGACTCTAAAGGGGACTCTGCTAAAGGACGTTCATCTTCCTGAGTTATCGTGTAACACAACTCTGTTGATTGTCACCATTGTGGTTATTATGCTAGTTCTGGGGATGGCTGTGGCCTTCTGCTGCTTCTACTTTGATCTGCCCTGGTATCTCAGGATGCTAGGTCAATGGACATTGCAGAGGATTAGAAAAACAACCCAAGAACAGCTCAAGAGAAATGTCCAGTTCCATGTGTTTATTGCATACAGTGAACATGATTCTACCTGGGTGAAGCACGAATTAATCCCCAATCTAGAGAAGAAAGAGAAATTGATTTGCCTTCATGAGGGAAACTTTGACCCTGGCAAGAGCATTATTGAAAATATCATGAACTGCATTGAGAAAAGCTATAAGTCCATCTTTGTTTTGTCTCCCAACTTTGTCCAGAGTGAGTGGTGCCATTATGAACTTTACTTCGCCCACCACAGTCTCTTCCATGAAAATTCTGATTACATAATTTTTATCTTACTGGAACCCATTCCACTCTACTGCATTCCTACCAAGTATCCTAAGCTGAAAGCTCTTATGGAAAAGAAAGCATACTTGGAATGGCCCAAGGATAGGCGTATATGTGGACTTTTTTGGGCAAATCTTCGAGCTGCTATTAATGCTAACTTATTAGAAACCAGAGAGATGTATGAACTACAGACATTTGTGGGGCTGAATGAAGAGTCTCAAGGTTCTGCAATCTCTCTGATAAGAACTGACTGCCTATAA

>EcabTLR10 Equus caballus Horse XM_001498678 2436 ATGAGACACATCAGAAGCATTTATGTATTTTGTTGTATTGTTATGTCAGCATGTGGCTGGGCTTCAAAGCTGCCAGAAGAAAGAGAATTGACAACCAACTGCTCCAATATGTCTCTAAGAAAGGTTCCTGTGGACTTGACGCCAACCACCACCACCCTGGATTTATCCTACAACCTCCTTTCTCAACTCCAGAGTTCAGATTTTCATTCTGTCTCTGAACTGAAAGTTTTGATTCTATGCCATAACAGAATCCAAGAACTGGATATCAAGACCTTTGAATTCAACAAGGAGTTAAGATATTTAGATTTGTCTTACAACAGACTGAAGATTGTAACTTGGTATTCACTGACAGGTCTCAGACATTTAGATCTTTCTTTCAATGACTTTGACACCATGCCTATCTCTGAGGAGACTGGCAACATGTCACACCTGGAAATCCTAGGTTTGAGTGGGGCAAAAATTCAAAAATCAGATTTCCAGAAAATTGCCCATTTACATCTAAATACTGTCTTCTTAGGATTGAGAACTCTTTCTCATTATGAAGAAGGTAGCTTGCCCATCTTAAACACAACAAAACTTCACATCGTTTTACCAATGAACACAAATTTTTGGGTTCTTTTGCGTGATGGAATCAAGACTTCAAAAATATTAGAAATGACAAATGTAGATGGCAAAAGCCAATTTGCAAGCTATGAAACTCAACAAAATCTTATTTTAGAGAATGCCAAGACATCTATTCTGTTACTTAATAAGGTTGATTTACTCTGGGATGACCTCCTCCTTATCTTCCAATTTGTTTGGCATACATCAGTGGAATACTTCCACATCCAAAATGTGACTTTTGGAGGTAAGGTTTATCTTGACCACAATTCATTTAACTACTCAAATACTGTAATGAGAGCTATAAAATTGGAGCATGTACATTACAGAGTTTTTTACATTCCACAAGAGAGAGTCTACTTGCTTTTTACCAAAATGGGTATAGAAAACCTGACAATATCAGATGCACAAATGCCACATATGGTGTTCCCGACTTGTCCTTCAAAATTCCAATATTTAAATTTTGCTAATAATATCTTAACAGATGACCTGTTTAAAAGATCTATCCACTTGCCTCATTTGAAAACTCTCATTTTGAAAGGCAATAAATTGGAGACACTTTCCTTAGTGAGTTGCTTTGCTAACAACACATCCTTGAAGCACTTAGATCTGAGCCAAAATCTGTTACAACATGAAAATGATGAAAATTGCTGGTGGCCAGAAACCTTGATCACTATGAACCTGTCATCCAATAAATTTGCTGATTCTGTTTTCAGGTGCTTGCCCCAAAGTATTCAAATACTTGACCTGAATAATAACAAAATTCAAACTGTCCCTAAAGAGATGATTCATCTGAAGTCTTTGCGAGAGCTAAATCTTGCATTTAATTTTCTAACTGATCTTCCTGGGTGCAGTCATTTCAGAAGACTCTCAGTTCTGAACATTGAAATGAACTTAATTCTCAGCCCATCTCTGGATTTTTTTCAGAGCTGCCAGGAAGTTAAGACTCTAAATGCAGGAAGAAATCCATTTCGGTGTACTTGTGAATTAAGAGATTTCATTCAGCTTGAAAAATATTCAGAGGGCATGATGGTTGGATGGTCAGATTCATACATCTGTGAGTACCCTTTAAGTCTAAAGGGGACTCAGTTAAAGGATGTTCACCTTCCTGAACTATCTTGCAACACAGCTCTATTGATTGTCACCATTGTGGTTGTCATGCTCATTTTGGGGATGGCTGCAACTTTCTGCTGCCTCCACTTTGACCTGCCCTGGTATCTTAGGATGCTACATCAATGGACACAGACATGGCACAGGGTTAGGAAGGCAACCCAAGGACAACTCAAGAGAAATGTCCAATTCCATGTGTTTATTTCATATAGTGAACGTGATTCCGCCTGGGTGAAGCATGAATTGATCCCCAATCTAGAGAAAGAAGATGGTTCTGTGTTAATTTGCCTTCATGAGGGAAACTTTGACCCTGGCAACAGCATTACTGAAAATATCATAAACCACACTGAGAAAAGCTATAAAATCATCTTAGTTTTGTCTCCAGACTTTGTTCAGAGTGAGTGGTACCGTTATGAACTCGACTTTACCCATCACAGTCTCTGTCATGAAAATTCTAATTACATAATTCTTATTTTACTAGAACCCATTCCACTCTACTGCATTCCTACCAGGTATCCTAAGCTGAAAGCTCTCATGGAAAAGAAAGCATACTTGGAATGGCCCAAGGATAGGCGTAAATGTAGACTTTTTTGGGCAAATCTTCGAGCTGCTATTCATGTTAATTTAGTAGAAACCAGAGAGACGTCTGAACTACAGACATTCATAGAGCTGAATGAAGAGTCTCAAGGGTCTGCAATTTCTCTGATAAGAACAGACTGCCTGTAA

>HsapTLR10 Homo sapiens Human BC089406 2436 ATGAGACTCATCAGAAACATTTACATATTTTGTAGTATTGTTATGACAGCAGAGGGTGATGCTCCAGAGCTGCCAGAAGAAAGGGAACTGATGACCAACTGCTCCAACATGTCTCTAAGAAAGGTTCCCGCAGACTTGACCCCAGCCACAACGACACTGGATTTATCCTATAACCTCCTTTTTCAACTCCAGAGTTCAGATTTTCATTCTGTCTCCAAACTGAGAGTTTTGATTCTATGCCATAACAGAATTCAACAGCTGGATCTCAAAACCTTTGAATTCAACAAGGAGTTAAGATATTTAGATTTGTCTAATAACAGACTGAAGAGTGTAACTTGGTATTTACTGGCAGGTCTCAGGTATTTAGATCTTTCTTTTAATGACTTTGACACCATGCCTATCTGTGAGGAAGCTGGCAACATGTCACACCTGGAAATCCTAGGTTTGAGTGGGGCAAAAATACAAAAATCAGATTTCCAGAAAATTGCTCATCTGCATCTAAATACTGTCTTCTTAGGATTCAGAACTCTTCCTCATTATGAAGAAGGTAGCCTGCCCATCTTAAACACAACAAAACTGCACATTGTTTTACCAATGGACACAAATTTCTGGGTTCTTTTGCGTGATGGAATCAAGACTTCAAAAATATTAGAAATGACAAATATAGATGGCAAAAGCCAATTTGTAAGTTATGAAATGCAACGAAATCTTAGTTTAGAAAATGCTAAGACATCGGTTCTATTGCTTAATAAAGTTGATTTACTCTGGGACGACCTTTTCCTTATCTTACAATTTGTTTGGCATACATCAGTGGAACACTTTCAGATCCGAAATGTGACTTTTGGTGGTAAGGCTTATCTTGACCACAATTCATTTGACTACTCAAATACTGTAATGAGAACTATAAAATTGGAGCATGTACATTTCAGAGTGTTTTACATTCAACAGGATAAAATCTATTTGCTTTTGACCAAAATGGACATAGAAAACCTGACAATATCAAATGCACAAATGCCACACATGCTTTTCCCGAATTATCCTACGAAATTCCAATATTTAAATTTTGCCAATAATATCTTAACAGACGAGTTGTTTAAAAGAACTATCCAACTGCCTCACTTGAAAACTCTCATTTTGAATGGCAATAAACTGGAGACACTTTCTTTAGTAAGTTGCTTTGCTAACAACACACCCTTGGAACACTTGGATCTGAGTCAAAATCTATTACAACATAAAAATGATGAAAATTGCTCATGGCCAGAAACTGTGGTCAATATGAATCTGTCATACAATAAATTGTCTGATTCTGTCTTCAGGTGCTTGCCCAAAAGTATTCAAATACTTGACCTAAATAATAACCAAATCCAAACTGTACCTAAAGAGACTATTCATCTGATGGCCTTACGAGAACTAAATATTGCATTTAATTTTCTAACTGATCTCCCTGGATGCAGTCATTTCAGTAGACTTTCAGTTCTGAACATTGAAATGAACTTCATTCTCAGCCCATCTCTGGATTTTGTTCAGAGCTGCCAGGAAGTTAAAACTCTAAATGCGGGAAGAAATCCATTCCGGTGTACCTGTGAATTAAAAAATTTCATTCAGCTTGAAACATATTCAGAGGTCATGATGGTTGGATGGTCAGATTCATACACCTGTGAATACCCTTTAAACCTAAGGGGAACTAGGTTAAAAGACGTTCATCTCCACGAATTATCTTGCAACACAGCTCTGTTGATTGTCACCATTGTGGTTATTATGCTAGTTCTGGGGTTGGCTGTGGCCTTCTGCTGTCTCCACTTTGATCTGCCCTGGTATCTCAGGATGCTAGGTCAATGCACACAAACATGGCACAGGGTTAGGAAAACAACCCAAGAACAACTCAAGAGAAATGTCCGATTCCACGCATTTATTTCATACAGTGAACATGATTCTCTGTGGGTGAAGAATGAATTGATCCCCAATCTAGAGAAGGAAGATGGTTCTATCTTGATTTGCCTTTATGAAAGCTACTTTGACCCTGGCAAAAGCATTAGTGAAAATATTGTAAGCTTCATTGAGAAAAGCTATAAGTCCATCTTTGTTTTGTCTCCCAACTTTGTCCAGAATGAGTGGTGCCATTATGAATTCTACTTTGCCCACCACAATCTCTTCCATGAAAATTCTGATCATATAATTCTTATCTTACTGGAACCCATTCCATTCTATTGCATTCCCACCAGGTATCATAAACTGAAAGCTCTCCTGGAAAAAAAAGCATACTTGGAATGGCCCAAGGATAGGCGTAAATGTGGGCTTTTCTGGGCAAACCTTCGAGCTGCTATTAATGTTAATGTATTAGCCACCAGAGAAATGTATGAACTGCAGACATTCACAGAGTTAAATGAAGAGTCTCGAGGTTCTACAATCTCTCTGATGAGAACAGATTGTCTATAA

>CjacTLR10 Callithrix jacchus Marmoset ENSCJAG00000000842 2436 ATGAGACTCATCGGAAACATTTACATATTTTGTAGTATTGTTATGACAGTAGAGGGTGAGGTTCCAGAGCTGCCAGGAGAAAGGGAGCTGATGACCAACTGCTCCAACATGTCTCTAAGAAAGGTTCCTGCAGACTTGACCCCAGCCACAACCACACTGGATTTATCCTATAACCTCCTTTTTCAACTCCAGAGTTCAGATTTTCATCCTGTCTCCAAACTGAAAGTTTTGATTCTATGCCATAACAGAATCCAGCAGCTGAATCTCAAAATCTTTGAATTCAACAAGGAGTTAAGATATTTAGATTTATCTAATAACAGACTGAAGAGTGTAAGTTGGTATTCACTGGCAGGTCTCAGGTATTTAGATCTTTCTTTTAATGACTTTGACACCATGCCTATCTGTGAGGAAGCTGGCAACATGTCACACCTGGAAATCCTAGGTTTGAGTGGGGCAAAAATACAAAAATCAGATTTCCACAAAATTGCTCATCTGCATCTAAATACTGTCTTCTTAGGATTCAGAACTCTTTCTCATTATGAAGAAGGTAGCCTGCCCATCTTAAACACAACAAAACTGCACATTGTTTTACCAATGAATACAAATTTCTGGGTTCTTTTGCGTGATGGAATCAAGACTTCAAAAATATTAGAAATGACAAATATAGATGGCAAAAGCCAATTTGTAAGTTATGAAATGCAACAAAATCTGAGTTTAGAAAATGCTAAGACATCGGTTTTATTACTTAATAAAGTTGATTTACTCTGGGACGACCTTTTCTCTATCTTCCAATTTGTTTGGCATACATCAGTGGAATACTTTCAGATCCGAAATGTGACTTTTGGTGGTAAGGTTTATCTTGACCACAATTCATTTGACTACTCAAATACTGTAATGAGAACTATAAAATTGGAGCATGTACATTACAGAATGCCTTACATTCAGCAGGATAAAATCTATTTGCTTTTGACCAAAATGGACGTAGAAAACCTGACAATATCAAATGCACAAATTCCACATATGCTATTCCCTAATTATCCTACAAAATTTCAACATTTAAATTTTGCCAATAATATCTTAACAGACGAGTTGTTTAAAAGAACTATCCAACTGCCTCACTTGAAAACTCTCATTTTGAAGGACAATAAGCTGGAGACACTTTCTTTAGTGAGTCGCTTTGCTAACAACACACCCTTGGAACACATGGATCTGAGTCAAAATCTATTACAACATAAAAATGATGAAAATTGCTCATGGCCAGAAACTGTGGTCACCATGAACCTGTCATACAATAAATTGTCTGATTCTGTTTTCAGGTGCTTACCCAAAAGTATTCAAATACTTGACCTAAATAATAACCAAATCCAAACTGTACCTAAAGAGGCTATTCATCTAATGGCCTTACAAGAGCTAAATATTGCATTTAATTTTCTAACTGATCTCCCTGGGTGTAGTCATTTCAGTAGACTCTCAGTTCTGAACATTGAAATGAACTTAATTCTCAGCCCATCTCTGGATTTTTTTCAGAGCTGTCAGGAAGTTAAGACTCTAAATGCAGGAAGAAATCCATTCTGGTGTACCTGTGAATTAAAACATTTCATTCGGCTTGAAACATATTCAGAGGGCATGATGGTTGGATGGTCAGATTCATACATCTGTGAATACCCTTTAAACCTAAGGGGGACTCGGCTAAAAGATGTTCATCTTCCTGAATTATCTTGCAACACAGCTCTGTTAATTGTCACCATTGTGCTTATCATGCTAGTTCTGGGGTTGGTTGTGGCCTTCTGTTGTCTCCACTTTGATCTGCCCTGGTATCTCAGGATGCTAGGTCAATGCACACAAACATGGCACAGGGTTAGGAAGACAGCCCAAGAACAACTCAAGAGAAATGTCCAATTCCACACATTTATTGCATACAGTGAACATGATTCTCTGTGGGTGAAGAATGAATTGATCCCCAATCTAGAGAAAGAAGATGGTTCTATCTTGATTTGCCTTTATGAAAGAAACTTTGACCCTGGAAAGAGTGTTAGTGAAAATATTGTGAGCTTCATGGAGGAGAGTTACAAGTCCATCTTTGTTCTGTCTCCCAACTTTGTCCAGAGTGAGTGGTGCCATTATGAACTCTACTTTGCCCACCACAATCTCTTCCATGAAAATTCTGATCATATAATTCTTATATTACTGGAACCCATTCCATTCTACTGCATTCCCACCAGGTATCATAAACTGAAAGCTCTCCTGGAAAAAAAAGCATACTTGGAATGGCCCAAGGATAGGCGTAAATGTAGGCTTTTCTGGGCAAACCTTCGAGCTGCTCTTAACATTCACGTATCAGCCACCAGAGAAATGTGTGAACTACAGACATTCGCAGAGTTCAGTGAAGAGTCTCGAGGTTCTACAGTCTCTCTGATGAGAACAGACTGCCTATAA

>MdomTLR10 Monodelphis domestica Gray Short-Tailed Opossum ENSMODG00000020531 2496 ATGGGAATCATCAGAAACATTTATATTTTGTATAGTATTGATCTGTCTGTACTTTGTGGGGCTCTGCTACTACCCGAAATGAATGAATTAACTACCAACTGTTCTCAGGAATATTTTAAAAAGGTTCTGATGGGCATGTCACTAAAAATCACCACAATGGATTTATCAAACACATTCACATGTCAGCCTCAGAACTTGGACCTTAGTCCTTTCTCTAAGCTAAAAGTCTTAATTCTCTCTCATAACAAAATCCAACATCTGGATATCAGCATCTTCCAGTTTAACAAGGAGTTGGAATATTTAGACTTATCTTACAATAGTCTGAGGAATATTTCTTGTTATCCACTTCTGGGTCTCAAACATTTAGATCTTTCATTTAATGACTTCAACAATATTCCCATCTGTCAGGAGTTTGGCAGCATGTCACAACTGGAATTTTTAGGACTGAGTGGGGCACAGATACAAAAATCACATCTGCAGAAAGTTGCTCATTTGCACCTAAACACTGTCTTCCTGGGCTTAAAACATCTTTCGTCCTATGAAGAAGGCAGCTTATCCCTTTTAAACACTGAAAGACTTCGTATAGTCTTTCCAGAGATTGCTGATTACAGGTTTATTTTGCGTGATGGAATCCACACTTCCAAAATCTTGGAAATGGCAAATATCAATATGAACCAATTTACCAACCATGACAATCAGAAGAATCCTCTTCTGGAGAAACCAAAGGGCTCCAAAACATCTCATCTGTTACTCAGTAATGTCTATGGACCCTGGCAGGAATTCCTCCAAGCGCTGCAGTTTGTTTGGCACTCATCAATTCAACAATTACAAATACAAAATTTTACCCTTGGTGCCCAAAGTGAATTTAAAACAAAAATATTTGACTACTCTAATACCTCAATGAAAGTCATAAAGGTAGAGCATGTGAGCATTAAGATATATTCCTTTCCACAGGATAGAATCTACTTGTTCTTTACCCAAATGGGCACTGAGAACTTGACAATATCAGGTGCAGGGATGCCACATATAATTTTCCCTGATTATCCCTCGAAATTTCAGCACTTGAATTTTACCAACAATGCTTTGACAGATGAATTATTCAGAAAGACACTTCTATTGCCACATTTGAAGACTCTTGTTTTGCAAAGGAATAAATTAGAGACACTTTCCACAGTGAGTTCCTTTGCTATCAATACATCCTTGATACACTTAGATCTAAGCCAGAATCTACTACAGCACACTGATGGAAACCATTGCCACTGGCCAGAAACCCTCACTACCATGAATTTGTCATCCAATAAATTTATTGACTCTATTTTCAAATGCATACCAAAAAGCATCCAAGTCCTTGATCTGCATAATAATGATATTAGAACGATCCCCAGAGAAATTGAGAATCTGAAAGCATTACAGGAGCTAAATATTGCATCCAATTCTCTAACTGATCTTCCTGGGTGTCGTAATTTCCAAAGGCTTTCTGCACTGAATGTTGAAATGAATTCCATTATTAGCCCATCTCTTGATTTCTTCCAGTCTTGTCAGGAAGTGCAGTCATTAAAAGCAGGAGACAATCCATTCAGATGTAATTGTGATTTAAGAAACTTCATCAACCTAGAAAAAAAATCCCAGGGCTTGATGATTGGGTGGCCAGATGCATATACCTGTGAATATCCTCTAGAACTAAAGGGAATTCATCTGGAGAATGTCGATCTGCCAGCATTATCCTGTAATACACCTCTATTGATTGCTGTCATTCTGATAATCGGGTTACTTTGGACAGTTGTCATAGCAATTCTCTGCATTCGCTTTGATTTGCCGTGGTACTTGAGGATGATAAGTCAGTGGACTCAGAACCGGCACCGAGTTAGGAATGTGCCCATAGAAGAACTCAAAAGAACAATTCAATTTCATGCTTTTATTTCATACAGTGAAATGGATTCTCTCTGGGTAAAAAGTGAGCTGATACCAAACCTGGAGAAAGAAGATAGGTCTATATCAATTTGTCTCCATGAGAGACACTTTGTTCCTGGCAAGAGTATCATTGAAAATATTATAAATTGCATTGAGAAATGCTACAAATCTATCTTTGTTTTGTCCCCTAACTTTGTTCAGAGTGAGTGGTGCCATTATGAACTTTACTTTGCTCACCACAAAGTATTTCATGAAGGTTCTAATAATTTAATTCTCATATTACTAGAACCAATTCCACAGTACAGCATTCCTAGCAAGTATCATAAGCTAAAAACTCTCATGGCACAAAGAACCTATCTGGAATGGCCCAAGGAGAAGAATAAGCATGGACTCTTCTGGGCTAACCTTAGGGCTACCATTAATATTAATTTGTCAGAATCTGGAAAGATGAACAAATCACAAACAATTTTAGAGTTAATGGAAGGAATCACAAGCATCTTCATGAAATTTGCACTTTTAATACATTTTCCTTCCATCCAGTCATTGAGTATTTTAGTGGAGCAGCTCAGCCCTAAAGTGCTATGA

>PpygTLR10 Pongo pygmaeus Orangutan ENSPPYG00000014667 2436 ATGAGACTCATCAGAAACATTTACATATTTTGTAGTATTGTTATGACACTAGAAGGTGATGCTCCAGAGCTGCCAGAAGAAAGAGAACTGATGGCTAACTGCTCCAACATGTCTCTAAGAAAGGTTCCTGCAGACTTGACCCCAGCCACAACAACACTGGATTTATCCTATAACCTCCTTTTTCAACTCCAGAGTTCAGATTTTCATTCTGTCTCCAAACTGAAAGTTTTGATTCTATGCCATAACAGAATTCAACAGCTGGATTTCAAAACCTTTGAATTCAACAAGGAGTTAAGATATTTAGATTTGTCTAATAACAGACTGAAGAGTGTAACTTGGTATTTGCTGGCAGGTCTCAGGTATTTAGATCTTTCTTTTAATGACTTTGACACGATGCCTATCTGTGAGGAAGCTGGCAACATGTCACACCTGGAAATCCTAGGTTTGAGTGGGGCAAAAATACAAAAATCAGATTTCCAGAAAATTGCTCATCTGCATCTAAATACTGTCTTCTTAGGATTCAGAACTCTTTCTCATTATGAAGAAGGTAGCCTGCCCATCTTAAACACAACAAAACTGCACATTGTTTTACCAATGGACACAAATTTCTGGGTTCTTTTGCGTGATGGAATCAAGACTTCAAAAATATTAGAAATGACAAATATAGATGGCAAAAGCCAATTTGTAAGTTATGAAATGCAACGAAATCTTAGTTTAGAAAATGCTAAGACATCGGTTCTGTTACTTAATAAAGTTGATTTACTCTGGGACGACCTTTTCCTTATCTTACAATTTGTTTGGCATACATCAGTGGAACACTTTCAGATCCGAAATGTGACTTTTGGTGGTAAGGCTTATCTTGACCACAGTTCATTTGACTACTCAAATACTGTAATGAGAACTATAAAATTGGAGCACGTACATTTCAGAGTGTTTTACATTCAACAAGATAAAATCTATTTGCTTTTGACCAAAATGGACATAGAAAACCTGACAATATCAAATGCACGAATGCCACACATGCTTTTCCCTAATTATCCTACAAAATTCCAATATTTAAATTTTGCCAATAATATCTTAACAGACGAGTTGTTTAAAAGAACTATCCAACTGCCTCACTTGAAAACTCTCATTTTGAATGGTAATAAACTGGAGACACTTTCTTTAGTGAGTTGCTTTGCTAACAACACACCCTTGGAACACTTGGATCTGAGTCAAAATCTATTACAACATAAAAATGATGGAAATTGCTCATGGCCAGAAACTGTGGTCAACATGAATCTGTCATACAATAAATTGTCTGATTCTGTCTTTAGGTGCTTGCCCAAAAGTATTCAAATACTTGACCTAAATAATAACCAAATCCAAACTGTACCTAAAGAGACTATTCATCTGATGGCCTTACGAGAGCTAAATATTGCATTTAATTTTCTAACTGATCTCCCTGGGTGCAGTCATTTCAGTAGACTTTCAATTCTGAACATTGAAATGAACTTAATTCTCAGCCCATCTCTGGATTTTGTTCAGAGCTGCCAGGAAGTTAAGACTCTAAATGCGGGAAGAAATCCATTCCGGTGTACCTGTGAATTAAAAAATTTCATTCAGCTTGAAACATATTCAGAGGTCATGATGGTTGGATGGTCAGATTCATACACCTGTGAATACCCTTTAAACCTAAGGGGAACTCGGCTAAAAGACGTGCATCTCCCCGAATTATCTTGCAACACAGCTCTGTTGATTGTCACCATTGTGGTTATCATGCTAGTTCCAGGGTTGGCTGTGGCCTTCTGCTGTCTCCACTTTGATCTGCCCTGGTATCTCAGGATGCTAGGTCAATGCACACAAACATGGCACACGGTTAGGAAAACAACCCAAGAACAACTCAAGAGAAGTGTCCGATTCCACGCATTTATTTCATACAGTGAACATGATTCTCTGTGGGTGAAGAATGAATTGATCCCCAATCTAGAGAAAGAAGATGGTTCTATCTCGATTTGCCTTTATGAAAGCTACTTTGACCCTGGCAAGAGCATTAGTGAAAATATTGTAAGCTTCATTGAGAAAAGCTATAAGTCCATCTTTGTTTTGTCTCCCAACTTTGTCCAGAATGAGTGGTGCCATTATGAATTCTACTTTGCCCACCACAATCTCTTCCATGAAAATTCTGATCATATAATTCTTATCTTACTGGAACCCATTCCATTCTACTGCATTCCCACCAGGTATCATAAACTGAAAGCTCTCCTGGAAAAAAAAGCATACTTGGAATGGCCCAAGGATAGGCGTAAATGTGGGCTTTTCTGGGCAAACCTTCGAGCTGCTATTAATGTTAATGTATCAGCCACCAGAGAAATGTATGAACTGCAGACATTCACAGAGTTAAATGAAGAGTCTCGAGGTTCTACAATCTCTCTGATGAGAACAGACTGCCTATAA

>OanaTLR10 Ornithorhynchus anatinus Platypus Ultra544:4,643,610-4,645,988 (omAna1) 2379 ATGGCGAGGAACCTGGGAATGTTTTTCATTCTCTACAGAGTCACCACGTTCGTGCTGGGAGAAACCGGCCAACCAGCGGCCAAGAACGAACTGATCGCCAATTGCTCCAATGGGCACCTGATCAGCGTTCCCACAGATGTGTCTTCTGGGACCACCACGCTGGACCTCTCCCACAATAACATATCCCAGCTCCAGCCCTTGGATCTGAGATCTCTCGTCAAGCTCAGAGTGCTGCTCCTCTCTCACAACACCATCCGCCATCTGGATGCCAATGTCTTTCGATTCAACGAGGCTTTGGAGCATTTAGACTTATCCTATAACCTCCTAGGAGAGATTTCTTGCCACCCGCTTAGGAGTCTCAGACACCTGGATCTCTCCTTCAACAACTTCCTCACTGTGCCTGTCTGCCCGGAGTTCGGGAGCCTGCCGCACCTGGAATTCCTAGGGCTGAGCGGGGAGCAGATACGGAAATCAGATTTCCAGAAAATTGCACATTTGCTCCTAAATGCCGTCTTTCTAAACTTAAACACTCCAATCCGTTATGAAGAGGGCAGCCTGCCAGTTTTGAACCCTGCCAAACTCCACATTGTCTTACCGGGAGACACCGATTCCCTGCTTATTTTGAAAGATGGCATGAAAACTTCCAAAATCTTTGAGCTCTCAAATCTATGTCTGCTTCCAAGCTGTAACACTCAACGAAATCCCCTTCTGGAAACCCTCCGGGACTCCAAGACAACACAGCTGTCTCTCAGTCGAGTCAACACAGCCTGGAAGGAATTTCTCCAGATCCTCCAGTTTGTGTGGCATTCATCTGTCCAGCAACTCCATATTCGAAACCTGACCTTTATTGATTATCCCAACTCTCATTCTGCCCACTTTAACTATCTCGGGACCGCAATGAGAGCCTTAACGGTGGAACAGGTACATATACAGACTTTCACTTTCCCACAGGACCTAGCCTACCAACTCTTTACTAAAATGGATATCGACAATCTGACTCTATCCGATGCCCAGATGCCACACATGGTGTTTCCCCAGGGCGTGAGGAGATTTCGGTATGTGAATTTTTCCAACAACGCCCTGTCGGATGCATTCTTCATCACGGATGCCAGGTTCCCAGCTTTGGAAACCCTCATTTTGCAAAGAAATATGTTGATGACCCTTTCCGTGGCAAGTTCCTTTGCGGCACACACCCCCTTGAAGTACATTGATCTGAGTCAGAATCACCTGCATCACAGGGACGACACCCCCTGCTTCTGGCCGGAGACGCTGGTGGCCATGAACCTCTCATCCAACAAGCTGGCTGATTCTGTTTTTGGCTGCTTGCCAAAGAGCATCCAAGTTCTTGATCTCCAGAACAATGAGATCAGGTCGGTACCCCGACAGATGGCAGATCTGAAAGGGCTGTTGGAGCTCAACATCGCCTCGAATTTTTTGACCGATCTCCCCGGATGCGGTCGTTTTAACCATCTAGCCGTGCTGAACATCGAGATGAATTCGATTCTCGTCCCATCTCTCGATTTCTTCCAGTCCTGCTCCGCTCTTAAGTCGGTCAAAGCAGGGAACAATCCCTTTCGGTGCTCCTGTGAGTTACGAGATTTCATTCGTTTGGAAAAAAGGTCCAAGGGCCTGATGGTTGGATGGGCTGAGGCATACCGATGTCAGTATCCCGTCTCCCTGAAAGGGACCTTGCTGAGGGACATCCACCTGCCTGAATTAGCCTGCAACATCCCCCTGCTAGTGGGGTCCGTGCTGGTGACCCTGTCGGTCATGGCGGCAGCTGGGGTGGCCCTGTGCCGCTACCTGGATGTGCCCTGGTACCTGAGGATGACGTGGCAGTGGGCGCAGACCCGGCGCAGGGCCCGGCGGGTGCCACCCACGGAGCTCGAGAGGATGGCCCAGTTCCACGTGTTCGTCTCCTACAGCGAGAGGGACGCGACCTGGGTCAAGGCCGAACTAATTCCCAACCTGGAGAGCGCCGCCATAAACATCTGTCTGCACGAGAGGAACTTCGTCCCCGGCAAGAGCGTCGTGGAGAACATCATCAGCTGCATCGAGAAGAGCCGCAAATCCATCTTTGTCCTGTCCCCCCACTTTGTCCAGAGTGAGTGGTGCCACTACGAGCTCTGCTTCGCTCACCACCGGCTCTTCCAAGAGGGCTCCGACAGCCTCATCCTCATCGTGCTGGCCCCGATTCCGCGGCACAGCATCCCCGCCCGCTATCACAAGCTCAAGTCCCTCATGGCCCGGAAGACCTATCTGGAATGGCCCCGGGAGAGGAGCAAGCAGGGCCTTTTCTGGGCCAACCTGAGGGCTGCGGTCCAAGTTCCTATATCGAAATCCGAGACGGATGGACTCGTGAGCGACCCACAGTCCTGA

>SscrTLR10 Sus scrofa Pig AB219565 2436 ATGAAACTTATCAGAAGCATTTACATATTTTGTGGTATTGTCATGTCAGTGCATGGAAGGACTTCAGAGCTGCCAGAAGAAAGGGAATTGACTACCAACTGCTCCAACATGTCTTTAAGAAAGGTTCCCACAGACTTGACCCCAACCACAACCACACTGGATTTATCCTACAACCTCCTTTTTCAACTCCAGCATTCAGATTTTCATTCTTTCTCAAAACTAAAAATTTTGATTCTGTGCCATAACAGAATCCAAGAGCTGGACATCAAGACCTTTGAATTCAACAAGGAGTTACAATATTTAGATGTGTCTTACAATAGACTGAAGACTGTAACTTGGTATTCACTGGCAAGTCTCAGACAGTTGGATCTGTCCTTCAATGACTTTGACAGCATGCCTATCTCCGAGGAGACGGGCAACATGTCACACCTGGAAATCCTGGGTTTGAGTGCGGCCAAAATACGAAAATCAGATTTCCAGAAAATTGCTCATTTGCATCTAAATACTGTCTTTTTAGGATTGAGAACTCTTTCTCATTATGAAGAAGGTAGCCTGCCCATCTTAAACACAACAAAACTTCACATTGTTTTACCAATAAACACAAATTTCTGGGATCTTTTGCGTGATGGAATCAAGACTTCAAAAATATTGGAAATGACGAATCTAGATGGCAAAAGCCAATTTGCAAGTCATGAAACTCAACAAAACCTTATTTTAGAGAATACCAAGACATCCATTCTGTTACTTAATAAAGTTGATTTACCCTGGGATGACCTTTTCCTTATCTTACAATTTGTTTGGCACACATCAGTAGAATACTTCCAGATCCAACATGTGATTTTTGGAGGCAAGGTTTATCTTGATCACAATTCATTTGACTACTCAAATACTGTAATGAGAACTATAAACTTGGAGCATGTACATTTCAGGGTTTTTAATATCCCACAGGAGAGAGTCTACTTGCTTTTTACCAAAATGGCTATAGACAATCTGACAATATTAGATGCACAAATGCCTCACATGTTGTTCCCTACTTATCCTACAAGATTCCAATACTTAAATTTTGCTAGTAATATCTTTACAGATGACGTGTTTAGAAAACCTGTCCAACTGCCTCATTTGAAAACTCTCATTTTGAAGGACAACAAATTGGAGACACTTTCCTTAGTGAGTTGCTTTGTCAGAAACACATCCTTAGAACACTTAGATCTGAGCCAAAATCTGTTACAATATGAAAATGATGAAAAGTGCTTGTGGCCAGAAACTTTGATCACCATGAACTTGTCATTCAATAAACTTGCTGATTCCGTTTTCAGGTGCTTGCCCAGAAATATTCAAATGCTTGACCTGAATAATAACAACATTCACACTGTCCCTAAAGAAATTATTCATCTGACATCCTTACAAGAGTTAAATCTTGCATTTAACTTTCTAACTGATCTTCCTGGGTGCAGTCATTTCAGAAGACTCTCAGTTCTGAACATTGAGATGAACTTAATCCTCAGCCCGTCGGTGGATTTTTTTCAGAGTTGCCAGGAAGTTAAGACTTTAAACGCGGGAGGAAATCCATTCCGGTGTACTTGTGAATTAAGAGATTTCATCCAGCTTGAAAAATATTCAGAGGGCATGATGGTTGGGTGGTCAGATTCATACATCTGCGAATACCCTTTGGATCTAAAGGGGACTCAGCTAAAAGATGTTCATCTTTCTGAATTATCTTGCAACATAGCTTTGTTGATTGTCATCATAGTGATTATCATGCTAGTTCTGGGGATGGCTGTGGCCCTCTGCTGCCTCCGCTTTGATCTGCCCTGGTATCTCAGAATGCTGGGTCAGTGGGCACGGACATGGTACAGGGTTAGGAAAACAACCCAAGGACAACTCAAGAGAAATATTCAATTCCACGTGTTTATTTCATTCTGTGAACATGATTCGGCCTGGGTAAAGCATGAATTGATTCCCAATCTAGAGAAAGAAGATGGTTCTGTCCTGATTTGCTTTCATGAGGGAAACTCTGATCCTGGCAAGAGCATTACTGAAGATATCATAAACTGCATGGAGAAAAGCTCTAAGTCCATCTTTGTTTTGTCTCCCAATTTCGTCCAGAGCGAGTGGTGCCACTATGAACTCTACTTTGGCCACCACCACCTCTTCCATAAAGGGTCTGATGACATAATTCTTATCTTACTGGAACCCATTCCACTCTACTGCATTCCTACCAGGTATCCTGCACTGAAAGCTCTCATGGAAAAGAAAGCATATTTGGAATGGCCCAAGGATAGGCGTAAATGTTGCCTTTTTTGGGCAAACCTTAGAGCTGCTATTCATGTTAATCTATTAGAAACCAGAGAGGTGTGTGAACTACAGACATTCACAGAGCTGAATGAAGGGTCTCGAGGTTCTGCAATATCTCTGATAAAAACAGACTGCCTATAA

>OcunTLR10 Oryctolagus cuniculus Rabbit ENSOCUG00000003701 2433 ATGAGACTTATCAGAAGCATTTACTTGTGTTGTACTATTGTGATATCAGTGGAGGGCTGGGCCCCAAAGCTGCCAGAGGAAAGAGAATTGACAACCAACCTCAGCAACTTGTCTCTGAGAAAGGTTCCCACAGACTTAGCCCCAGCCTCAACCACAGTAGATTTATCCTACAACCTCCTTTCCGAACTCCAGAATTCAGATTTTCATTCCGTCTCCAAACTGAAAATTTTGATTCTGTGCCATAATAGTCTCCAGCAACTGGATATCAAGAGCTTTGAGTTCAACAAAGAGTTACGATATTTAGATGTGTCTCACAACAGACTGAACGTCGTAACCTGGTATCCGTTGGCCAGTCTCAAACATGTAGATCTCTCCTTTAATCGTTTTGACACCATGCCCATCAGTGAGGAGACTGGCCACATGTCACAGCTGGAAACCCTGGGGCTGAGTGGGGCAAAAATCCAAAAATCGGATTTCCAGAAAATTTCTCACCTGCATCTCAATACTGTCATCTTAGGACTGAGAACCCTCTCTCATTATGAAGAAGGCAGCCTGCCCATCTTAAACACAACAAAACTTCATATTGTTCTACCGATGAACGTCAATTTTTGGGCACTCTTGTGTGATGGACTGAAGACTTCAAAAATACTAGAAATGACAAATATAGATGGCAAAAGCCAATTTGTAAGTTATGAAACTCGACACGATCTCATTTTGGAGAATGCCAAGACATCCATTCTGTTATTTAATAAAGTTGATTTGCTCTGGGATGACCTTCTCCTTGTATTCCAGTTTGTTTGGCATACTTCAGTGGAATACTTCCAAATCGAAAATGTGACTTTTGGAGGTATTGTTTATCTTGACCACGAATCCTTTGACTATACAAGCACTGTGATGAGAACTGTAAAATTGGAGCATGTTCATTTCAGAGTTTTTAATATCCCACAGGATAGAATCTACTTGCTTTTCACCAAAATGGACATAGAAAACCTGACAATATCAGATGCCCAAATGCCACACATGCTTTTCCCTAATTCTCCTACAAGGTTCCAGTATTTAAATTTTGCCAATAACATCTTAACGGATGACCTGCTTAAAAAACCTGCCCAACTGCCTGATCTGAAAACTCTCATTCTGAAGGGAAATAAACTGGAGACGCTTTCGTTGGCGAGTTGCTTTGTTAGCCTCACACCGGTAATGCACTTGGATCTGAGCCAAAATCTATTACAACATCAAAATGATGACAATTGCTCATGGCCAGACACCTTGCTTACCATGGATCTGTCGTCCAATAAATTGGCTGGCTCTGTTTTCAGGTGCTTGCCCAGAAGTATTCAAACACTCGATCTGAATAATAACAAAATTCAAACTATCCCTAAGGAGATTATCCATCTGACGTCCTTACGAGAGTTAAACATCGCATTGAATTTTCTAACTGATCTCCCTGAATGCAGTCATTTCAGAAAACTCTCAGTGCTGAACGTTGAAATGAACTTGATTCTCAGCCCCTCTCTGAATTTTTTGCAGAGCTGCCAGGAAGTTCGGACTCTAAATGTGGGGAAAAATCCATTCCAGTGTACTTGTGACTTAAGAGATTTCATTCAGCTTGGGAAACACTCAGAGGGCATGCTGCTTGGATGGTCCGACTCGTACCTCTGTGAATACCCTTTGAATCTAAAGGGCACTCAGCTAAAGGATGTTTACCTTCCCGAATTATCTTGCAACACCGCTCTGTTAATTGCCACCATTGTGGCTATTGTGCTAGTTCTGGGGATGGCTGTGGCCTTTAGCTGCCTCTGCCTGGACCTGCCCTGGTATCTCAGGATGCTAGGTCAATGGATACAAATACGTCTCAGGGTTGGGGAAGCAACCCAAGAACAACTCAGGAGAAATGTCCAGTTCCATGCCTTCATTGCATATAGTGAACGTGATTCTCTCTGGGTGAAGAGTGCATTGATCCCCAGTCTAGAGAAAGAAGATGGTTCTGTCTTGATTTGCATTTATGAGAGAAACTTTGACCCTGGCAAGAGCATGACTGAAAATATCATCGATTGCATTGAGAAAAGCTATAAATCCATCTTTATTTTGTCTCCCGACTTTGTGCAGAGTGAGTGGTGCCATTACGAAGGGATTGCCCACCATGATCTCTTCCGTGACAATTCTGATCATATAATTTTTATCTTACTGGAACCTGTCCCATTCTACTGCATTCCCACTAGGTATCAGAAGCTGAAAGATCTCATGGGAAGGAAAGCACTCTTGGAATGGCCCAAGAGTAGGCATAAGTGTAGGCTTGTTTTGGCAAACCTTCGAGCTGCTATTGATGTGAATTTATCAGATAACAGACAAGCGTGTGAACTACAGACATTCACAGGACTGAACGAACAGGCCCGAGGTTCTGCAAGCTCTCTGATAAGAACCGACTGCCTATAA

>RnorTLR10 Rattus norvegicus Rat XM_223422 2436 ATGAGACTCATCAGCAGCATCTATACCCTTTATAACATCATTATGTCAACAGAAGCCTGGGCTCCAAGACCCACAGATGAAAGGGAACTGATCTCCAACTACTCCAACATGGCTTTAAGGAAGGTTCCCCCAGATGTGACCCCAGCCACAACCATACTGGATTTATCCTACAACCTCCTCTATCAATTCCAGAGCTCAGATTGCCACTCCTTCTCCAAACTGCGTTTTCTGATCCTGTGCCATAACAGCATCCAACAGCTGGACACCAAGATCTTTGACCTTAACAAGGAGCTAAGCTATTTAGACTTGTCTTACAATAGACTACAGTCCATCACTTGGTCTTCCTTGGTAGATCTCAGACATTTAGATCTTTCTTTTAACGACTTCGATACTCTGCCTGCCTGTGAGGAGATTGGCAACATGCGACATCTGGAAACCTTAGGTTTGAGTGGGGCCAAAATCCAAAGATCAGATCTCCAGAAAATTGCTAATTTGCAATTCAGCACTGTGTTCATAGGGCTGAATACTCTTTCTCACTATGAAGAAGGCAGCCTGCCTGTCCTAAACACGACAGTGCTTCACATAGTTTTACCAATGGCTGCTAACTTCTGGGTGCTTCTGTGTGATGGAATGAAGACTTCAAAGGTAGTCGAAATGTCAAACATAGGTGGCAAGAGCCAGTTTGTAAGTTATGAGACTCCGAGAACTCTTTCTGTGGAGAATACCAAGGCGTCCACTCTTATATTCCATAAAGTTGACCTGCTCTGGGATGACCTTCTCCACGTTTTTCAGTTTGTGTGGCGGACTTCAGTGGAATGCTTCCAGATTCAGGACCTGACTTTTGGAGGGATGGCTAGTCGGGACCACAGTTCATTTGACTACTCAAATACCGTAATGAAAGCTGTAATCTTGGAGAGCATACATTTCAGAATCTTTTTCATTCCCCAGAATAAAATCTACTTGTTTTTCACCCAAATGGATTTAGAAAACCTGACGATATCAGACGCACAAATGCCACATCTTCTCTTCCCTAGTCACCCCCAAAGTTTCCAACATTTAAATTTTGCCAATAACATCTTAACAGATGACCTGTTTAAAAACCCCATCCAGATGCCTTATCTGAAAACTCTCGTTTTGAGGGGCAACCAACTGGAGACTCTTTCTCTAGTGAGTCTCTTTGCCAACCACACGTCCTTGCTTCACCTGGATCTGAGCCAGAACCTATTGCAACATGGAAACGGGGAGAAATGCATGTGGCCCGACACCTTGATCCTCTTGAACTTGTCATCCAATAAATTTGATGGTTCTGTTTTCAGATGTTTGCCTAAAAGTATCCAGACACTTGATCTGAACAATAACAAAATTCGAGCAGTCCCTAAAGAGATTATATACCTAAAATCTTTACGAGAGCTAAGTATTGCTTTTAATTTTCTGACTGACCTCCCTGGGTGTGATCACTTCAAACAGCTCTCTAGTCTGAACATTGAAATGAATTTGATTCTCAGCCCATCTCTGGATTTTTTCCAGAGCTGCCAGGAGGTAAAGACTCTAAACGCAGGGCGAAATCCATTCCGGTGTATCTGTGAATTTAGAGACTTCATTTGGCTTGAGAAACATTCCAAGGGCCTGATGTCTGGATGGCCAGACTCCTACGCCTGCGAATACCCTTTGAGTCTAAAGGGGACTCCATTAAAAGATGTTTATCTTCCCGAATTAGCTTGCAACACAACTTTGCTGATTGTCACCATTGTGCTGGTTATCCTGGTTCTGGGTTTGGTTGTAGCATTCTGCATTCTAAAATTTGACATGCTTTGGTATCTCAGGGTGCTGGCCCAGTGCATCCGGACACAGCATAGGATTAGGAAGACAGTTCGAGAGGAGCGTAAGAGAAGTATACAGTTCCATGCATTTATCTCCTACGCTGCACATGATTCTGTCTGGGTGAAGAATGAACTGATTCCCAATCTGGAGACAGAAGATGGCTCTGTCTTGCTTTGTCTTCCCGAGAGGAACTTTGACCCTGACAGGAGCATTGCTGAAAATATTATAAGCTGCACTGAGAAAAGCTATAAGTCCATCTTTGTGCTGTCCCCCAACTTTGTGCAGAATGGGTGGTGCCATAATGAACTCTATCTGGCCCACCACAATCCTTTCCGTGACAATTATGATTACATCATTCCCATCTTACTAGAACCCATTCCCTCCTACTGTGTTCCTACCAAGTTCCCTAAGCTGAAAGCTCTTTTGGAAAAGAAAGTATACTTAGAGTGGCCCAAGGACAGGCGCAAACATGGGCTTTTTTGGGCAAACCTTCGAGCTGCCATTAACGTAAATTTATCAGACACAAGGGGAAGGTGTGAACTACAGACATTCACAAAGCCTAACGCGGATTGCCAAGGTTCTACCATCTCTCTGGTAAGAACAGACAGCTTCTAA

>MmulTLR10 Macaca mulatta Rhesus Monkey XM_001088509 2436 ATGAGACTCATCAGAAACATTTACATATTTTGTAGTATTGTTATGACAGTAGAGGGCGAGGCTCCAGAGCTGCCAGAAGAAAGGGAACTGATGACCAACTGCTCCAACATGTCTCTAAGAAAGGTTCCCGCAGACTTGACCCCAGCCACAACCACACTGGATTTATCCTATAACCTCCTTTTTCAACTCCAGAGTTCAGATTTTCATTCTGTCTCCAAACTGAAAGTTTTGATTCTACGCCATAACAGAATTCAACAGCTGAATCTCAAAACCTTTGAATTCAACAAGGAGTTAAGATATTTAGATTTGTCTAATAACAGACTGAAGAGCGTAACTTGGTATTTACTGGCAGGTCTCAGGTATTTAGATCTTTCTTTTAATGACTTTGACACCATGCCTATCTGTGAGGAAGCTGGCAACATGTCACACCTGGAAATCCTAGGTTTGAGTGGGGCAAAAATACAAAAATCAGATTTCCAGAAAATTGCTCATCTGCATCTAAATAGTGTCTTCTTAGGATTCAGAACTCTTTCTCATTATGAAGAAGGTAGCCTGCCCATCTTAAACACGACAAAACTGCACATTGTTTTACCAATGGACACAAATTTCTGGGTTCTTTTGCGTGATGGAATCAAGACTTCAAAAATATTAGAAATGACAAATATAGATGGCAAAAGCCAATTTGTAAGTTATGAAATGCAACGAAATATTAGTTTAGAAAATGCTAAGACATCAGTTCTATTACTTAATAAAGTTGATTTACTCTGGGACGACCTTTTCCATATCTTACAATTTGTTTGGCATACATCAGTGGAACACTTTCAGATCCAAAATGTGACTTTTGGTGGTAAGGCTTATCTTGACCACAATTCATTTGACTACTCAAATACTGTAATGAGAACTATAAAATTGGAGCATGTACATTTCAGAGTGTTTTACATTCAACAGGATAACATCTATTTGCTTTTGACCAAAATGGACATAGAAAACCTGACAATATCAAATGCACAAATGCCACATATGCTTTTCCCTAATTATCCTACAAAATTCCAATATTTAAATTTTGCCAATAATATCTTAACAGACGAGTTGTTTAAAAGAACTATCCAACTGCCTCACTTGAAAACTCTCCTTTTGAATAGCAATAAACTGGAGACACTTTCTTTAGTGAGTTGCTTTGCTAACAACACACCCTTGGAACACTTGGATCTGAGTCAAAATCTATTACAACATAAAAATGATGAAAATTGCTCATGGCCAGAAACTGTGGTCAACATGAATCTGTCATACAATAAATTGTCTGATTCTGTCTTCAGGTGCTTGCCCAAAGGTATTCAAATACTTGACCTAAATAATAACCAAATCCAAACTGTACCTAAAGAGACTATTCATCTGATGGCCTTACGAGAGCTAAATATTGCTTTTAATTTCCTAACTGATCTCCCTGGGTGCAGTCATTTCAGTAGACTTTCAATTCTGAACGTTGAAATGAACTTAATTCTCAGCCCATCTCTGGATTTTGTTCAGAGCTGCCAGGAAGTTAAGACTCTAAATGCGGGAAGAAATCCATTCCGGTGTACTTGTGAATTAAAAAATTTCATTCAGCTTGAAACATATTCAGAGGTCATGATGGTTGGATGGTCAGATTCTTACACCTGTGAATACCCTTTAAACCTAAGGGGCACTCGGTTAAAAGATGTTCATCTCCCTGAACTATCTTGCAACACAGCTCTGTTGATTGTCACCATTGTGGTTATCATGCTAGTTCTGGGGTTGGCTGTGGCCTTCTGCTGTCTCCACTTTGATCTGCCCTGGTATTTCAGGATGTTAGGTCAATGCACACAAACATGGCACAGGGTTAGGAAGACAACTCAAGAACAACTCAAGAGAAATGTCCGATTCCACGCATTTATTTCATACAGTGAACATGATTCTCTGTGGGTGAAGAATGAATTGATCCCCAATCTAGAGAAAGAAGATGGTTCTATCTTGATTTGCCTTTATGAAAGATACTTTGACCCTGGCAAGAGCATTAGTGAAAATATTGTAAGCTTCATTGAGAAAAGCTATAAGTCCATCTTTGTTTTGTCTCCCAACTTTGTCCAGAATGAGTGGTGCCATTATGAATTCTACTTTGCCTACCACAATCTCTTCCATGAAAATTCTGATCATATAATTCTTATCTTACTGGAACCCATTCCATTCTACTGCATTCCCACAAGGTGTCATAAACTGAAAACTCTCCTGGAAAAAAAAGCATACTTGGAATGGCCCAAGGATAGGCGTAAATGTGGGCTTTTCTGGGCAAACCTTCGAGTTGCTATTAATGTTAACATATCAGCCACCAGAGAAATGTATGAACTGCAGACATTCACAGAGTTAAATGAAGAGTCTCGAGGTTCTACAGTCTCTCTGATGAGAACAGACTGTCTATAA

>DrerTLR2 Danio rerio Zebra Fish ENSDARG00000037758 2367 ATGAGACTCGTAGGAACAATGACTGCCATAATCCTTATTATGTTCATTCTGGCTCAAGGTCTCGAGTGCTCCCAGACATGTAAATGTGATCAGATGTATTTCTGCAACTGTTCCTCAAACAACCTCCATCAGGTTCCAACAGTTCCATCAGATGTGCTTGGCCTTGACCTGTCTTTCAATCAAATTGAGTCAATCAACATGACTGATCTCAGCTCTTACAATGAGCTGATAATCCTCAACTTGCATAAGAACAAACTCAGGCATATACACAGGGATGCATTCAAGTCCCAACATAACCTGGAAGTTCTCGATCTCTCCTTAAACAACCTGAACAATCTCTCTCCGTCTTGGTTTCACAAGTTAAAATCTCTCCAGCAACTTAACCTTGTAGGAAACCCATACTCAACTGTGGGACCTGCTCCAATCTTCAAGTCTCTTGTAAACTTGAGGACACTTCACTTAGGTAGTCCTTCACTGAGAGAGCTACACAAGAATGGCCTAGATGTGCTCACTCATCTGGACGAGATGACATTTTTTGGCAGTAACCTGAGGTCATATGAGAACGGGAGCTTGAAAGCAGCTCGTCCCATCGGTTCAGTCTCTTTGAGTCTACAGAATTTGTTTGAGAGTGATCCAGAACTAGTTTCCAAGGTCCTTCAAGATGTCTCCCACCCTGAAACTCTGCTGATAATCAAAGATGTCACTATGAAAACAAACACATCTACAGAACCCTTCAAAATGGTGAAGGAAGGTGGCACTAAGAGCCTGACTTTCCAAAACTCCTCCACGACCGATCAAGCCTTGACCTCTTTTTTAGAGTTCATGGATGGCTCTCCATTGTCTTTTATTGGACTTGAAGATATACAATTTGTTGGGATAGGAGAGTGGCAGAAAGCTAAATACACACATCATGACAGCCTACGCACAGCCTACTTGCGTAATATTGAAATAGAAGGTTTCTTTGGGTTTAGCAGCATGATAGAGTTGGGATTTCTACTGAAGCACTTCCACAACGTGTCTGTGATCAATGCCACAGTGTTTGTGATTCCTAAGGAAACCACATTCCTGTTGAAAAATCTAGAATACATGGACCTCAGCCAGAATCTCCTGACAGACTTGACTATTCAACCAACACTGTATACAGGATCTGGCGCATATCAAAACCTCAACATGCTCAATGTGAGTCAGAATGTTTTAAAATCTCTCGGGCTGATGTCTAGGTTGGTCACCAATCTTAAAAAGCTCAAATATTTAGACTTAAGTTATAACAGTTTTGTTTCAATGCCAGAAAAGTGTAGCTGGCCTGTTACTCTGAGGTTTCTGAACCTTTCAAGCACAAAGTTAAGCACATTGACTCCTTGCTTGCCTTCGAGTTTGACAGTTCTGGATCTCAGCGAAAATGATCTTAAGGCGTTCAAGCAAAGATTTCCTCATCTCACTACACTTATACTGACAGGCAATCGCTTAATGAAACTGCCAGATGGGAAATTATTCCCAAGCCTAAATACATTGCTGATACAAAGGAACGCCTTACGGATGTTCAACCAAAGCTCTTTGAGGAGTTTTAAAACTCTGCTTTACTTGGAGGCAGGTGCCAACAATTTTGTGTGCTCCTGCAAGTTTGTTTCTTTCTTTAAAAAGGACGTTGAGGATTTGATCACCCTACAGGATGGCCGTCAAAATTACGTATGTAATACTCCCTTCACTCTCAGAGGTAATGCGATTGACAGTGTCAGACTGTCTGTGTTTGAGTGTTATATGATCCCTGCCGTCTCAGTGCTTTGTTTTGGGATTATCACAGCACTCGGTCTTGTTGTGTTAACCTGCCACAAGCTTCACGTCATATGGTACCTGCAGATGACTAAAGCATGGATACAAGCCAAACGGAAACCTGCTGTCGGTCGATTACCTGAAGAGCTCCGCTATGATGCTTTTGTGTCTTACAGCCAACATGACGCTGAATGGGTCGAGGAGATTCTTGTTGCTGAGCTTGAAGACACTCAGCCTTCGTTTTCCCTGTGTTTGCACAAACGGGACTTCAGACCGGGCCGCTGGATCGTGGACAACATCATTGACTCAATTGAAAAGAGCTATCGAACTCTTTTTGTTCTGTCGGAGCACTTTGTGAGCAGTGAATGGTGCCGTTACGAGCTGGACTTCTCACATTTCCGCATCATGGATGAACACAATGACTCCGCTGTCCTGGTGCTTCTGGAACCAATAAAGAAGGAGACGATTCCCAAACGCTTCTGCAAGCTGAGAAAGATTATGAACTCCAGGACGTATCTGGAGTGGCCTGAGGATGAGGACAAGAGAGATGAGTTTTGGAGCAATCTGAGAGCCGCTTTACAGAGAGACGAGTGTTAA

>XtroTLR2A Xenopus tropicalis Western Clawed Frog scaffold_60:1,193,457-1,195,805 (xenTro2) 2349 ATGTATATCTACAGTATTTGTATAATCTACTTACTGGCAATTGCAGAACTTTCCAAAGGGAATACAGACTGCCCCTGTGACGCAGCACATTTCTGCCATTGTTCCTCCATGCACTGGGAGGCTATTCCATCTGGATTGCCAAGAGATGTCAGGGGATTAAACCTGTCCAGCAATGCCATACAGATTGTCACTGAGACTGACCTAAAGCCCTATGACCAACTGCAGACACTCCTGCTGCAGTATAATGCAATACACACTATAAATGACGGCTCATTCCAACCACTTGGAAACCTTGAAGAATTAGATTTATCATACAACAATTTAGTCCTTTTGTCTTCAGTTTGGTTTCGAAATCTGAACAAGTTAAAGCATTTAAATCTCCTAGGTAACCAATACATAACACTTGGGAATGATTCACTATTTGCCAGTCTTTCCTCTTTAAGTTCATTACAATTTGGAAATCAGAACTTTGACGCTATACAGAAGCAAAGCTTTGAGGGATTAGAGAGACTTAATACACTTGAAATAAACGCTGCACAGTTAACACTGTATGAAGAGGGAAGTCTTGGAAGGATGAAGCAAATTTACCATACTATTTTGAGAGTAAATCTTACTTTACTACCTGCACTGCTCAAAGACTTGGTGCATTCCGTGACCCTGCTAGAAATTACCGACACAGAATTTAGCACTCCTGCTGACATGCAAGCCTTTGAAGTTCTTGCTTACACCAGTGTGAAGCAATTCATTTTCAGGAACTGCACAATTGTTGACGTGAGTGGATACAGATTTGTTGAAATTATTCAGACTTATAGAAATATAACAGATGTTGTCATGAAAAACTGCAGACTTTTAGGAAGAGGATTAGATTGTCCAAATCTGAAAAGAACAACCTCTTCTGTTACTACAATTATTATAAATAACATTGAAGTATCATTGTATTATATCTTTTCTGACCTTAGTTCATTGCATCAAATTATACCACAGTTTACTTATGTAACTGTAACGGACTCCCCAGTTTATTTAATTCCATGTCAGTTTTCACAGTCATTCACTTCTCTTCAATACCTGGATGTTAGTGGAGGTAAACTGGATAATACTTATTTAGGAAGCTCAGTGTGTTACCATGAAGGCGGGGGTGCATGTCCCAAGTTGCAGACTTTAAATGTCAGTAGAAATCTTCTGACCTCTGTGCCAAAAGTTGCGCAATTCCTGTCTGGGCTGAATTACCTCACTAACCTGGACATCAGCCAGAATAAATTCAGTGAATTATCAACTTCTAAGTGCCAATGGCCTAAAAACCTAAAATACTTGAACATCTCAAACAATCAGATTAATATTATAACTACTTGCATTCCACCAACTTTACAGATCTTGGATGTGAGTTCAAATTATCTCACAGTTTTTGCAATTGAAATGCCAAATTTGACAGAACTTTACATATCAAACAACAGACTGAGTAAGTTACCAGAAGGCATGTATTTTACAAGTTTAGTATTATTAAGTATAAGCAGAAATGATCTAAATGGTTTTGGCCAGTCAGATTTAGAGTTGTTTTCCAACCGTACCATACTGGATGCAAGAGCCAACAATTATAAATGTTCGTGTCAATTTCTAGATTTTATTCGCTCAAATACAGCTGTATTGGTAGGTTGGCCAGATAACTACAAATGTGCTTCTCCTGCTTCAGTAAAAGATGTACAAATACAAGATGCAAACCTGCCTCCCCTGGTGTGCCATAAAACACTAATTGTAACACTGTCCTGTATTCTCTTGATAGCACTTGTAGCAGCTATTGTGGCTCTTTGCCACTTCCTTCATGTTGTATGGTATGCAAAGATGACCTGGGCCTGGCTAAAAGCCAAAAGAAAGCCCCTGAAAAACTGTGACAGAGAGATTTGTTTTGATGGGTTTGTTTCTTACAGTGAAAGAGACTCTGAATGGGTTGAGAATATGATGGTGCCAAAGCTGGAAAATGCCACCCTGGCAATGAAACTGTGCTTACATAAGCGCGACTTTGTACCCGGCAAGTGGATTATTGATAACATTATAGATGCAATGGAGAAGAGCTATAAAACTGTGTTTGTGCTCTCCGAGCACTTTGTCAGAAGTGAATGGTGTAAGTACGAGCTGGAATTCTCTCACTTCCGTCTCTTTGATGAAAATAACGACTCCGCCATTTTGATTCTTTTGGATCCCATTGAGAAAGAAACTGTCCCAAAACGATTCTGCAAGCTGAGGAAGCTCATGAACACTAAAACATACTTGGAGTGGCCTACGGACGAGGAGCAGCAAGAAGTATTCTGGGATAATTTGAAAACAGCGTTGCAACCAGCAGATTATATAAATTAA

>XtroTLR2B Xenopus tropicalis Western Clawed Frog scaffold_60:1,181,164-1,183,506 (xenTro2) 2343 ATGATGCAATCTATATCCCTGAGCTGCCTTATCCTCTCCCTGGTTGTTACAGTACTTTCCAAAGGGAATACAGACTGCCCCTGTGACGCAGCACATTTCTGCCATTGTTCCTCCATGCACTGGGAGGCTATTCCATCTGGATTACCAAGAGATGTCAGGGGATTAAACCTGTCCAGCAATGCCATACAGATTGTCACTGAGACTGACCTGCAACCCTATGACCAACTGCAGACACTCCTGCTGCAGTATAATGCAATACACACTATAAATGACGGCTCATTCCAACCACTTGGAAACCTTGAAGAATTAGATTTATCATACAACAATTTAAGTCACTTATCATCCGCTTGGTTTAGAAATTTGTACAACTTAAAGCACTTAAATCTCCTAGGTAACCAATACTTAACACTTGGAAATAATTCACTATTTGCCAGTCTTTCCTCCTTAAGATCATTACAGTTCGGAAATCAGGATTTTTCTGCTATTCAGAAGCTTCATTTTGAGAGTTTAGAGAGACTTAATATACTTGAAATAAAGGCGGCACAACTTGGACTGTATGAAGAGGGGAGCCTTGCAGCAATAAAGCAAATAAACCATGCTGTTTTGACAGTGAATATTCAGAACCTACGTGCGCTGCTAAATGACTTTGTACATTCTGTGACCCTGCTAGAAATGAAAGACATGCAGTTTGGCGATGCTGCTGATGTGAAAGCTATGGTGATACTCAATGATACCAGTATAAAGTACTTGGTCTTCAGGAACTGTATTATAACAGACCAAAGTGCATCAAGATTACTTGAAATTTTTCAGACTTACAGAAACATAACAGATTTTATATTGGATGATAGCACACTTTATGGAACAGGGACAGCATCCCCAGTAATTGGAGATGACCCCTTTTCTGTTACTACAGCCATTATAAATAAATTGCACATACCCAAGTTTTTCTTGTTTTCTGACCTGCAAGGAATATATAAACTTGCTTCTAATTTTAAAAATATTGCTTGTATAGACAGCAAAGTTTTCTTAATGCCATGTGCTTTTTCACGGTCTTTTTCTTCTCTTCAGTACCTGGACCTCAGTGGAAATCTCCTTTCGGATAACCTGTTGGCAAGCTCGGCATGTGCCTTTGAAGGCGGGGGTGCATGGCCCATATTGCAGACTTTAAATGTCAGTAGAAATCTTCTGACCTCTTTGCGGAGACTTGCACAAATAACGTCTGGCCTGAAATACCTCACTAACCTGGATGTCAGTCAAAATAATTTTGGTGAATTATCAACTTCTACTTGCCAATGGCCTAAAAGCCTTAAATACCTGAACATCTCAAGCAGTCAGATGAGTAATATAACTTGCATTCCTCCAACTTTACAGATCTTGGATGTGAGTTCAAATTATCTCACAGTATTTACAATTAAAATGCCAAATTTGACAGAACTTTACATATCCAACAACAGACTGAGTAAGTTACCAGAAGGCATGTATTTTCCAAGGTTATTTATGTTAAGTATAGATAGAAACAAACTAAATGACTTCTACCAGTCAGATTTGGATCTGTTTCCCCAACTCACTACACTGGATGGAAGAGACAACAATTATTTATGTTCTTGCCAATTTTTAAGCTTTATGCATTCCCATACAATTGCTTTGGTAGGTTGGCCCGATGACTACATATGTGATTCTCCTTCTTCAGTAAGAGGCAAACGGATACAAGATGCAAACCTGCCTCCCCTAGTGTGCCATAAAACACTAATTGTAACACTGTCCTGTATTCTCTTGATAGCACTTGTAGCAGCTATTGCAGCTCTTTGCCACTTCCTTCATGTTGTATGGTATGCAAAGATGACCTGGGCCTGGCTAAAAGCCAAAAGAAAGCCCCTGAAAAACTGTGACAGAGAGATTTGTTATAATGGGTTTGTTTCTTACAGTGAAAGAGACTCTGAATGGGTTGAGAATATGATGGTGCCAAAGCTGGAAAATGCTGTCCCGCCAATGAAACTGTGCTTACATAAGCGCGACTTTGTACCCGGCAAGTGGATTATTGATAACATTATAGATGCAATGGAGAAGAGCTATAAAACTGTGTTTGTGCTCTCCGAGCACTTTGTCAGAAGTGAATGGTGTAAGTACGAGCTGGAATTCTCTCACTTCCGTCTCTTTGATGAAAATAACGACTCCGCCATTTTGATTCTTTTGGAGCCCATTGAGAACGAAACTGTCCCAAAACGGTTCTGCAAGCTGAGGAAGCTCATGAACACTAAAACATACTTGGAGTGGCCTACGGACGAGGAGCAGCAAGAAGTATTCTGGGATAATTTGAAAACAGCGTTGCAGTCAGAATACTAA

>GgalTLR2A Gallus gallus Chicken AB050005 2382 ATGTTCAACCAAAGTAAACAGAAACCAACAATGAAGCTGATGTGGCAAGCATGGCTGATCTACACAGCCTTGGCTGCGCACCTCCCTGAAGAACAAGCCCTGAGACAGGCCTGTCTTTCATGCGATGCCACTCAGTCATGCAACTGCTCTTTCATGGGTTTGGACTTCATTCCCCCGGGGCTCACAGGCAAAATCACGGTGTTAAACCTGGCCCACAACAGGATAAAGCTGATCCGAACACATGATCTGCAAAAGGCTGTGAACCTGAGAACCCTGCTGCTGCAGTCCAACCAAATCAGCTCCATAGACGAGGACTCGTTTGGCTCTCAGGGGAAACTGGAGCTCTTGGACTTGTCAAATAATAGCCTGGCTCACTTGTCCCCAGTGTGGTTTGGGCCCCTTTTTTCGCTCCAACACCTTCGCATTCAAGGCAATTCCTACAGCGACCTGGGGGAAAGTTCCCCCTTTTCCAGCCTGAGAAACTTGAGCTCCCTCCACCTGGGCAACCCACAGTTCTCCATCATCAGGCAAGGAAACTTTGAGGGCATTGTGTTTCTCAACACGCTGAGGATCGACGGTGACAATCTCAGTCAGTATGAGCCTGGAAGTCTGAAATCGATCAGGAAGATAAATCACATGATCATAAGCATAAGAAGGATTGACGTATTCTCAGCAGTCATCAGGGACCTTCTGCACTCTGCCATTTGGTTAGAAGTTAGAGAGATCAAATTAGATATTGAAAATGAAAAACTGGTTCAGAACTCTACTCTTCCTTTGACGATACAGAAACTTACATTTACCGGTGCTTCATTCACAGATAAATATATTAGCCAAATAGCAGTGCTATTGAAGGAAATTAGATCTTTGAGAGAGTTAGAAGCAATAGATTGTGTGCTTGAGGGGAAAGGAGCATGGGATATGACAGAAATTGCAAGAAGTAAGCAAAGTTCTATTGAAACACTATCAATAACAAATATGACTATTCTGGATTTTTATTTGTTCTTTGACCTGGAAGGTATAGAGACACAGGTAGGTAAGCTGAAAAGACTCAGCATTGCAAGCTCTAAAGTCTTCATGGTACCATGCAGACTAGCAAGATATTTTTCATCACTTTTATATCTTGATTTCCATGATAATTTGCTTGTAAATAATCGCTTAGGAGAGACAATCTGTGAAGATGCATGGCCTTCTTTACAAACTCTAAATCTAAGTAAAAATTCTCTGAAATCCCTAAAACAGGCTGCAAGATATATAAGTAATCTACACAAACTGATTAATCTTGACATTAGTGAAAACAATTTTGGTGAGATTCCAGACATGTGTGAATGGCCTGAAAACCTGAAATATCTGAATCTCTCCAGCACTCAAATTCCCAAATTAACAACTTGCATTCCCTCAACTCTTGAAGTGCTGGACGTTAGTGCTAACAACCTGCAGGATTTTGGACTGCAACTGCCATTTCTCAAGGAGCTGTACCTGACAAAAAACCATCTGAAGACCTTGCCTGAAGCCACAGACATTCCTAACTTAGTGGCCATGTCGATCAGCAGAAACAAGCTCAACAGCTTCTCCAAGGAAGAGTTTGAGTCCTTCAAGCAAATGGAGCTGCTGGATGCCAGCGCCAATAACTTTATCTGCTCCTGTGAATTCCTCTCCTTCATTCACCATGAGGCAGGGATAGCCCAGGTGCTTGTGGGGTGGCCAGAAAGCTACATCTGTGACTCTCCGCTGACAGTGCGAGGGGCACAGGTTGGGAGTGTGCAGCTGTCACTGATGGAGTGCCACCGGTCCCTCCTAGTGTCCTTGATCTGCACCCTGGTGTTCCTGTTCATCCTCATCCTGGTGGTCGTTGGGTACAAGTACCATGCAGTCTGGTACATGAGAATGACCTGGGCATGGCTCCAAGCCAAGCGGAAGCCCAAGCGAGCCCCCACGAAAGACATCTGCTACGACGCTTTTGTCTCCTACAGTGAGAACGACTCCAACTGGGTGGAAAACATCATGGTGCAGCAGCTGGAGCAGGCATGTCCCCCCTTTAGGCTGTGCCTCCATAAGCGGGACTTTGTGCCTGGGAAGTGGATTGTGGACAACATCATTGACTCCATTGAGAAGAGCCACAAGACGCTCTTTGTGCTATCAGAGCACTTTGTGCAGAGCGAGTGGTGCAAGTATGAGCTGGATTTCTCGCACTTTCGCCTTTTTGACGAGAACAACGATGTGGCGATCCTCATCCTGCTGGAGCCCATTCAGAGCCAGGCGATCCCCAAGAGGTTCTGCAAACTGCGGAAGATAATGAACACCAAGACCTACCTGGAGTGGCCTCCTGATGAAGAGCAACAGCAGATGTTTTGGGAAAACTTGAAAGCAGCCTTGAAGTCATAG

>GgalTLR2B Gallus gallus Chicken AB046533 2346 ATGCATACTTGGAAAATGTGGGCAATTTGCACAGCCTTGGCTGCGCACCTCCCTGAAGAACAAGCCCTGAGACAGGCCTGTCTTTCATGCGATGCCACTCAGTCATGCAACTGCTCTTTCATGGGTTTGGACTTCATTCCCCCGGGGCTCACAGGCAAAATCACGGTGTTAAACCTGGCCCATAACAGGATAAAGGTGATCCGAACACATGATCTGCAAAAGGCTGTGAACCTGAGAACCCTGCTGCTGCAGTCCAACCAAATCAGCTCCATAGACGAGGACTCGTTTGGCTCTCAGGGGAAACTGGAGCTCTTGGACTTGTCAAATAATAGCCTGGCTCACTTGTCCCCAGTGTGGTTTGGGCCCCTTTTTTCGCTCCAACACCTTCGCATTCAAGGCAATTCCTACAGCGACCTGGGGGAAAGTTCCCCCTTTTCCAGCCTGAGAAACTTGAGCTCCCTCCACCTGGGCAACCCACAGTTCTCCATCATCAGGCAAGGAAACTTTGAGGGCATTGTGTTTCTCAACACGCTGAGGATCGACGGTGACAATCTCAGTCAGTATGAGCCTGGAAGTCTGAAATCGATCAGGAAGATAAATCACATGATCATAAGCATAAGAAGGATTGACGTATTCTCAGCAGTCATCAGGGACCTTCTGCACTCTGCCATTTGGTTAGATGTAAGAAAACTAGCATTCAGTGTTCCTGAAAAAATACAACTTTTGAGAATTATGTCTTCCTCTTTCGCAAAGAAAATTTCTTTAAAACAGTGCTTATTTACAGATGCTACTGTGCCTGAGATTGTCAGCATTTTAGAAGGCATGCCAAAATTAATGGAGGTGGAGATGAAAGACTGTACACTCTTGGGCACTGGAAAGTGGTATAAACAAATTCATGCAAACCAGTCACAATCTCTGAGAATTCTGACAATAGAGAATTTATCTATAGAAGAGTTTTATTTGTTTACAGATCTTCAGTCTGTACTAGATCTCCTATCTCTTTTTAGGAAAGTCACAGTTGAAAATACCAAGGTGTTTCTGGTGCCATGCAAACTTTCACAACACCTTCTATCATTGGAGTATCTTGACCTTAGCGCAAATTTACTTGGAGATCAGAGTTTGGAGCATTCAGCCTGTCAGGGTGCATGGCCTTCATTACAAACTCTAAATCTAAGTCAGAATTCATTGAGTGACTTAAAAATGACAGGTAAAAGCTTGTTTCATCTAAGAAACCTAAATCTCTTAGACATCAGTGAAAACAATTTTGGTGAGATTCCAGACATGTGTGAATGGCCTGAAAACCTGAAATATCTGAATCTCTCCAGCACTCAAATTCCCAAATTAACAACTTGCATTCCCTCAACTCTTGAAGTGCTGGACGTTAGTGCTAACAACCTGCAGGATTTTGGACTGCAACTGCCATTTCTCAAGGAGCTGTACCTGACAAAAAACCATCTGAAGACCTTGCCTGAAGCCACAGACATTCCTAACTTAGTGGCCATGTCGATCAGCAGAAACAAGCTCAACAGCTTCTCCAAGGAAGAGTTTGAGTCCTTCAAGCAAATGGAGCTGCTGGATGCCAGCGCCAATAACTTTATCTGCTCCTGTGAATTCCTCTCCTTCATTCACCATGAGGCAGGGATAGCCCAGGTGCTTGTGGGGTGGCCAGAAAGCTACATCTGTGACTCTCCGCTGACAGTGCGAGGGGCACAGGTTGGGAGTGTGCAGCTGTCACTGATGGAGTGCCACCGGTCCCTCCTAGTGTCCTTGATCTGCACCCTGGTGTTCCTGTTCATCCTCATCCTGGTGGTCGTTGGGTACAAGTACCATGCAGTCTGGTACATGAGAATGACCTGGGCATGGCTCCAAGCCAAGCGGAAGCCCAAGCGAGCCCCCACGAAAGACATCTGCTACGACGCTTTTGTCTCCTACAGTGAGAACGACTCCAACTGGGTGGAAAACATCATGGTGCAGCAGCTGGAGCAGGCATGTCCCCCCTTTAGGCTGTGCCTCCATAAGCGGGACTTTGTGCCTGGGAAGTGGATTGTGGACAACATCATTGACTCCATTGAGAAGAGCCACAAGACGCTCTTTGTGCTATCAGAGCACTTTGTGCAGAGCGAGTGGTGCAAGTATGAGCTGGATTTCTCGCACTTTCGCCTTTTTGACGAGAACAACGATGTGGCGATCCTCATCCTGCTGGAGCCCATTCAGAGCCAGGCGATCCCCAAGAGGTTCTGCAAACTGCGGAAGATAATGAACACCAAGACCTACCTGGAGTGGCCTCCTGATGAAGAGCAACAGCAGATGTTTTGGGAAAACTTGAAAGCAGCTCTAAAATCGTAG

>MgalTLR2A Meleagris gallopavo Turkey FJ477860 2382 ATGTTCAACCAAAGTAAACAGAAGCCAACAACTAAGCTGATGTGGCAAGTGTGGATGATCTACATAGCCTTGGCTGCACACCTCCCTGAAGAACAAGCCCTGAGACAGGCCTGTCTTTCATGTGATGCCACTCAGTCTTGCAACTGCTCTTCCATGGGTTTGGACTTCATTCCCTCGGGGCTCACGGGCAAAATTACGGCGTTAAACCTGGCCCACAACAGGATAAAACTGATCCGGGCACATGATCTGCAGCAGGCTGTGAACCTGAGAGCCCTGCTGCTGCAGTCCAACCAAATCAGCTCCATAGACGAGGACTCGTTTGCCTCCCAGGGGAAACTGGAGCTCTTGGACCTGTCAAATAACAGCCTGGCTCGCTTGTCCCCGGCGTGGTTTGGGCCCCTTTTTTCGCTCCAGCACCTCCGCATTCAAGGCAATTCCTACAGCGACCTGGGAGAAAGTTCCCCCTTTTCCAGCCTGAGAAACTTGAACTCCCTCCACCTGGGCAATCCACAGTTCTCCGTCATCAGGCAAGGAAACTTTGAGGGCATTACATTTCTCAGCACGCTGAAGATCGACGGAGGCAATCTCAGTCAGTATGAACCTGGAAGTCTGAAATCGATTAGGAAGATAAATCACATGATCATAAGCGTAAGAAGGATTGATGTATTCTCAGCAGTCATCAGGGACCTTCTGCACTCTGCCATTTGGTTAGAAGTTAGAGAGATCAAATTAGATATTGAAAATGAAAAACTGATTCAGAACTCTACTCTTCCTTCAACGATAGAAAAACTTACATTTACTGGTGCTTCATTCACAGATCAATATATTAGCCGAATAGCAGTGTTATTGAAGGAAATCACATCTTTGAGAGAGTTAGAAGCAATAGATTGTGTGCTTGACGGGAAGGGAGAATGGGATACAACAGAAATTGCAAGAAGTAGGCAAAGTTCTGTTGAAACAATATCAGTAACAAATATGACTATTCTGGATTTTTATTTGTTCTTTGATCTGGAAGGTATAGAGACACAAGTAGATAAGCTGAAAAGACTCAGCATTGAAAGCTCCAAAGTCTTCATGGTACCGTGCAGACTGGCAAGATATTTTTCATCTCTTTTATTTCTTGACTTCCATGCAAATTTGCTTGTAAATAATAGCTTAGCTGAGTCAACCTGTCAAGGTGCATGGCCTTCATTACAAACTCTAAATCTAAGTAAAAATTCTCTGAAATCCCTAGAACAGGCTGCAAAATATATAAGTAATCTACACAAGCTGATTAATCTTGATATTAGTGAAAACAATTTTGGTGAGATTCCAGATGTGTGTGGATGGCCTGAAAACCTGAAATACCTGAATCTCTCCAGCACTCAAATTCCCAAATTAACACGTTGCATTCCCTCAACTCTTGAAGTGCTGGACGTTAGTGCTAACAACCTGCAGGATTTTGGTCTGGAACTGCCATTTCTCAAGGAGCTGTACCTGACAAAAAACCATCTGAAGACCTTGCCCGAAGCTGCAGCCATTCCTAACTTAGTGGCCATGTCGATCAGCAGAAACAATCTCAACAGCTTCTCCAAGAAAGAGTTTGCGTCCTTCAAGCAAATGGAGCTGCTGGATGCTAGCGCCAATAACTTTATTTGCTCCTGCGAATTCCTCTCCTTCATTCAGCATGAGGCAGGGATAACCCAGGTGCTTGTGGGGTGGCCAGAAAGCTACATCTGCGACTCTCCGCTGGCGGTGCGAGGGGCACAGGTTAGGAGCGTGAGTCTGTCGCTGATGGAGTGCCACCGGTCCTTCCTAGTGTCTTTGATCTGCATTCTGATGTTCCTGTTCATCCTCGTCCTGGTGGTCATTGGGTACAAGTACCATGCGGTCTGGTACATGAGAATGACCTGGGCATGGCTCCAAGCCAAGCGGAAGCCCAAGCGAGCCCCCACGAAAGACATCTGCTACGACGCTTTTGTCTCCTACAGTGAGAACGACTCCAACTGGGTGGAAAACATCATGGTGCAGCAGCTGGAGCAGGCGTGTCCACCCTTTAGGCTATGCCTCCATAAGCGGGACTTTGTGCCTGGGAAGTGGATTGTGGACAACATCATCGACTCCATTGAGAAGAGCCACAAGACGCTCTTTGTGCTATCAGAGCACTTTGTGCAGAGCGAGTGGTGCAAGTACGAGCTGGATTTCTCGCACTTTCGCCTCTTTGACGAGAACAACGATGCAGCGATCCTCACCCTGCTGGAGCCCATTCAGAGCCAGGCGATCCCCAAGAGGTTCTGCAAACTGCGGAAGATAATGAACACCAAGACCTACCTGGAGTGGCCTCCTGATGAAGAGCAACAGCAGATGTTTTGGGAGAACTTGAAAGCAGCTCTAAAATCCTAG

>MgalTLR2B Meleagris gallopavo Turkey FJ477861 2346 ATGCGCACTTGGAAAGTGTGGATGATCTACATAGCCTTGGCTGCACACCTCCCTGAAGAACAAGCCCTGAGACAGGCCTGTCTTTCATGTGATGCCACTCAGTCTTGCAACTGCTCTTCCATGGGTTTGGACTTCATTCCCTCGGGGCTCACGGGCAAAATTACGGCGTTAAACCTGGCCCACAACAGGATAAAACTGATCCGGGCACATGATCTGCAGCAGGCTGTGAACCTGAGAGCCCTGCTGCTGCAGTCCAACCAAATCAGCTCCATAGACGAGGACTCGTTTGCCTCCCAGGGGAAACTGGAGCTCTTGGACCTGTCAAATAACAGCCTGGCTCGCTTGTCCCCGGCGTGGTTTGGGCCCCTTTTTTCGCTCCAGCACCTCCGCATTCAAGGCAATTCCTACAGCGACCTGGGAGAAAGTTCCCCCTTTTCCAGCCTGAGAAACTTGAACTCCCTCCACCTGGGCAATCCACAGTTCTCCGTCATCAGGCAAGGAAACTTTGAGGGCATTACATTTCTCAGCACGCTGAAGATCGACGGAGGCAATCTCAGTCAGTATGAACCTGGAAGTCTGAAATCGATTAGGAAGATAAATCACATGATCATAAGCGTAAGAAGGATTGATGTATTCTCAGCAGTCATCAGGGACCTTCTGCACTCTGCCATTTGGTTAGATGTAAGAAAACTAGCATTCAGTGCTCCTGAAAAAATACAATTTTTGAGAATTATGTCTTCCTCTTTCACAAAGAAAATATCTTTAAAACAGTGCTTATTTACAGATGCTACTGTACCAGGAATTGTCAGCATTTTAGAAGGCATGCCAAAATTAATGGAAGTGGAAATGAAAGACTGTACACTCTTGGGCACTGGACTGTGGTATACACAAATTCATGCAAAGCAGTCACAATCTCTGAGGGTTCTGACAATAAAAAATTTATCTATAGAAAATTTTTATTTGTTTACAGATCTTCAGTCGGTACTAGATCTACTATCTCTTTTTAGGAAAGTCACAGTTGAAAATACCAAGGTGTTTCTGGTACCATGCAAACTTTCACAACAACTTCTCTCATTGGAGTATCTTGACCTTAGTGCAAATTTACTTGGAGATCAGAGTTTGGAGCATTCAGCCTGTCAGGGTGCATGGCCATCATTACAAACTCTAAATCTAAGTCAGAACTCACTGAGTGACTTAAAAATGACAGGTAAAAGCTTGTTTCATCTAAGAAAGCTAAATCTTTTAGACATCAGTGAAAATAATTTTGGTGAGATTCCAGATGTGTGTGAATGGCCTGAAGACCTGGAATACCTGAATCTCTCCAGCACTCAAATTCCCAAATTAACACGTTGCATTCCCTCAACTCTTGAAGTGCTGGACGTTAGTGCTAACAACCTGCAGGATTTTGGTCTGGAACTGCCACTTCTCAAGGAGCTGTACCTGACAAAAAACCATCTGAAGACCTTGCCCGAAGCTGCAGCCATTCCTAACTTAGTGGCCATGTCGATCAGCAGAAACAATCTCAACAGCTTCTCCAAGAAAGAGTTTGCGTCCTTCAAGCAAATGGAGCTGCTGGATGCTAGCGCCAATAACTTTATTTGCTCCTGCGAATTCCTCTCCTTCATTCAGCATGAGGCAGGGATAACCCAGGTGCTTGTGGGGTGGCCAGAAAGCTACATCTGCGACTCTCCGCTGGCGGTGCGAGGGGCACAGGTTGGGAGCGTGAGTCTGTCGCTGATGGAGTGCCACCGGTCCTTCCTAGTGTCTTTGATCTGCATTCTGATGTTCCTGTTCATCCTCGTCCTGGTGGTCATTGGGTACAAGTACCATGCGGTCTGGTACATGAGAATGACCTGGGCATGGCTCCAAGCCAAGCGGAAGCCCAAGCGAGCCCCCACGAAAGACATCTGCTACGACGCTTTTGTCTCCTACAGTGAGAACGACTCCAACTGGGTGGAAAACATCATGGTGCAGCAGCTGGAGCAGGCGTGTCCACCCTTTAGGCTATGCCTCCATAAGCGGGACTTTGTGCCTGGGAAGTGGATTGTGGACAACATCATCGACTCCATTGAGAAGAGCCACAAGACGCTCTTTGTGCTATCAGAGCACTTTGTGCAGAGCGAGTGGTGCAAGTACGAGCTGGATTTCTCGCACTTTCGCCTCTTTGACGAGAACAACGATGTGGCGATCCTCATCCTGCTGGAGCCCATTCAGAGCCAGGCGATCCCCAAGAGGTTCTGCAAACTGCGGAAGATAATGAACACCAAGACCTACCTGGAGTGGCCTCCTGATGAAGAGCAACAGCAGATGTTTTGGGAGAACTTGAAAGCAGCTCTAAAATCCTAG

>AplaTLR2A Anas platyrhynchos Beijing Duck (Mallard) DBS1405N01 2382 ATGTTCAACCAACCAAAACAGAAATCAACAGCTACACATACCTGGCAAGTGTGGGCCATGTACGTGGTCTTGGCTGTCAGTCTCTCTGAGGAACAAGCCCTGAAGCAGGTCTGTCCTTCATGCGATGCCACTCAGTCTTGCAACTGCTCTTCCATGGGCTTGGACTTCATTCCCTCGGGGCTCACGGACAAAATCACGCTGTTAGACTTGGCCCGCAACAGGATAAAGCACATCCGAGCGCATGATCTGCAGCAGGCTGTGAACCTGAGGACCCTGCTGCTGCAGTCCAACAAAATCAGCTCCATAGACGAGGACTCGTTTGTCTCCCTGGGGAAGCTGGAGCTCTTGGACTTGTCAAATAACCGCCTGGCTCACTTGTCCCCAAGGTGGTTTGATCACCTTGTTTCGCTCCAGCACCTCCACATTCACGGCAATTGCTATAGCGACCTGGGGGAAAGTTCTCCCTTTTCTAGCCTGAGAAACTTGAGCTCTCTCCACCTGGGCAACCCGCGGTTCTCCACAATAAGGCAAGGAAACTTTGAGGGCATTACGCTTCTCGACCAGTTGTGGATCGAGGGCAGCAATCTCAGTGTGTATGAGCCTGGAAGTCTGAAGTCAATTAAGAAGATAAATCACATGATCATAAGCATAAGAAGGGTAGATCTGTTCTCAGCAATTGTTCGTGATCTTCTGCACTCTGCCATTTGGTTAGAAATTAGAGAAATTAAATTGGACATTGAAAATGAAAAACTGGTGCAGAACTCTACACTTCCTTTAACAATACAAAAAATGACTTTTACCAGAGCTTCATTCTCAGATAAATATGCTAGCCAAATAATGGTGTTATTGAAAGAAATTACATCTTTGCGAGAGTTAGAGTCAATAGATAGTGTGCTTGAGGGGAATGGAAATTGGAATACTGAATTAATTAAAGTAAGTGGACAAAGTTCTGTTGAAACAGTATCAGTAACTAATATGACTATTCTGCATTTTTATTTGTTTTTAGACTTGAAAGGTTTGGAGTCACAAATAAGTAAACTGAAAAGACTCAGCATTGCAAGCTCTAAAGTTTTCATGGTACCATGCAGTCTTGCAAGAAATTTTTTATCGCTTGTGTATCTTGACTTTCATGATAATTTGCTTGTAAATAATCGCTTAAGTGAGACAATCTGTAAAGATGCTTGGCCTTCATTGCAAACTTTAAATCTAAGTAAAAACTCTTTAAAATCTCTAAACCAGACTGCAAAATATGTAACTAATCTACCCAAACTGAATAATCTTGACATTAGCCAAAACAATTTTGGTGAGATTCCAGATGTGTGTGAGTGGCCAGCAAAGCTGAAATATTTGAATCTCTCCAGGACTCGTATTTCCAAATTAACAGCTTGCATTCCCTCAACTCTCGAAGTGTTGGACATCAGTGCAAACAACCTGAAGGAGTTTAATCTGCATCTGCCGTTTCTCAAAGAGCTGTACCTTGCAAAAAACCAGCTGAAGGCCTTGCCTGATGCGGCAAGCATTCCTAACTTGGTGGCCATGTCAATCGGAAGAAACAAGCTCAGCAGCTTCTCCAAGGAAGAGTTGGAGTCCTTCAAGAAAATGGAGCTGCTGGATGTCAGCGCCAATAACTTTATCTGTTCCTGTGAATTCCTCTCCTTCATTCAGCACCAGGCAGGGATAGGCCAGATGCTCGTGGGGTGGCCGGAAGGCTACATCTGCGACTCTCCCCTGGCAGTGAGAGGGGCACAGGTTGGAGCCATTCATCTCTCACTGATGGAGTGTCACCAGTCCCTGGTGGTGTCCTTGATCTGTGCTGTGTTTTTCCTGGTCATCCTCGTCCTGGTGGCCATCGGGTACAAGTACCACGCAGTCTGGTACATGAGAATGACGTGGGCATGGCTCCGAGCCAAGCGGAAGCCCAAGAGAGCCCCCCCCAAGGACATCTGCTACGATGCTTTTGTCTCCTACAGTGAGAACGACTCCAACTGGGTGGAAAACGTCATGGTGCAGGAGCTGGAGCAGGCGTGTCCCCCCTTTCGGCTGTGCCTCCATAAGCGGGACTTTGTGCCCGGGAAGTGGATTGTGGATAACATCATCGACTCCATTGAGAAGAGCCACAAAACGCTCTTCGTGCTGTCGGAGCACTTTGTGCAGAGCGAGTGGTGCAAGTACGAGCTGGACTTCTCGCACTTCCGCCTGTTTGACGAGAACAACGATGCGGCCATTCTCATCCTCCTGGAGCCCATCCAGAGCCAAGCTATTCCCAAGAGGTTCTGCAAACTGCGGAAGATAATGAACACAAAGACCTACCTGGAGTGGCCTCGTGAGGAAGAGCAGCAGGAGATGTTTTGGGAAAACTTGAAAGCAGCTTTGAAATCATAG

>AplaTLR2B Anas platyrhynchos Beijing Duck (Mallard) DBS1405N01 2352 ATGTCGACACATACCTGGCAAGTGTGGGCCATGTACGTGGTCTTGGCTGTCAGTCTCTCTGAGGAACAAGCCCTGAAGCAGGTCTGTCCTTCATGCGATGCCACTCAGTCTTGCAACTGCTCTTCCATGGGCTTGGACTTCATTCCCTCGGGGCTCACGGACAAAATCACGCTGTTAGACTTGGCCCGCAACAGGATAAAGCACATCCGAGCGCATGATCTGCAGCAGGCTGTGAACCTGAGGACCCTGCTGCTGCAGTCCAACAAAATCAGCTCCATAGACGAGGACTCGTTTGTCTCCCTGGGGAAGCTGGAGCTCTTGGACTTGTCAAATAACCGCCTGGCTCACTTGTCCCCAAGGTGGTTTGATCACCTTGTTTCGCTCCAGCACCTCCACATTCACGGCAATTGCTATAGCGACCTGGGGGAAAGTTCTCCCTTTTCTAGCCTGAGAAACTTGAGCTCTCTCCACCTGGGCAACCCGCGGTTCTCCACAATAAGGCAAGGAAACTTTGAGGGCATTACGCTTCTCGACCAGTTGTGGATCGAGGGCAGCAATCTCAGTGTATATGAGCCTGGAAGTCTGAAGTCAATTAAGAAGATAAATCACATGATCATAAGCATAAGAAGGGTAGATCTGTTCTCAGCAATTGTTCGTGATCTTCTGCACTCTGCCATTTGGTTAGAAATTAGAGAAATAGCATTCAATATAGCTGAAGAAATGCAACTTTTGAGCGTTTTATCTTTGTCTTTTGCAAAGAAAATTTCTTTTAAACAGGTCTTACTCACAGATGCTACTGTGCCTGGGATTGTCACTATTTTAGAAAAGGTGCCAAGATTAGTTGAGGTGGAGATGAAAGACTGTAGACTCTTGGGCACTGGACGATGGGATTTAGAAATTCAAGCAAACCAATCACAGTCTCTGAGAGTTCTGACAATTGAGAAATTATCTATAGAGGAATTTTATTTGTTTACGGATCTTCAGGCTGTACTAGATCTAGTATCTCTCTTTACCAAAATCACAGTTGAAAATACCAAGGTCTTTTTGGTACCATGCAGGCTTTCAAAACAACTTCTGTCATTAGAATATCTTGACCTTAGTGCAAATTTGCTTGGAGATCAGAGTTTGGAACATTCAGCCTGTCCGGGTGGTTGGCCTTTACTAAAAACTTTAAATTTAAGTCAGAATTCACTGAGTGACTTTAAAATGACAGCTAAAAGCTTGTCTCATCTAAGAAACTTAATTCTTTTAGACATTAGCCAAAACAATTTTGGTGAGATTCCAGATGTGTGTGAGTGGCCAGCAAATTTGAAATATTTGAATCTCTCTAGGACTCAAATTCCCAAATTAACGGCTTGCATTCCCTCAACTCTCGAAGTGTTGGACATCAGTGCAAACAACCTGAAGGAGTTTAATCTGCATCTGCCGTTTCTCAAAGAGCTGTACCTTGCAAAAAACCAGCTGAAGGCCTTGCCTGATGCGGCAAGCATTCCTAACTTGGTGGCCCTGTCAATCAGAGGAAACAAGCTCAACAGCTTCTCCAAGGAAGAGTTGGAGTCCTTCAAGAAAATGGAGCTGCTGGATGCCAGTGCCAATAACTTTATCTGTTCCTGTGAATTCCTCTCCTTCATTCAGCACCAGGCAGGGATAGGCCAGATGCTCGTGGGGTGGCCAGAAGGCTACATCTGCGACTCTCCCCTGGCAGTGAGAGGGGCACAGGTTGGAGCCGTTCATCTCTCACTGATGGAGTGTCACCAGTCCCTGGTGGTGTCCTTGATCTGCGCTGTGTTTTTCGTAGTCATCCTCGTCCTGGTGGCCATCGGGTACAAGTACCACGCAGTCTGGTACATGAGAATGACGTGGGCATGGCTTCGAGCCAAGCGGAAGCCCAAGCGAGCCCCCCCCAAGGACATCTGCTACGATGCTTTTGTCTCCTACAGTGAGAACGACTCCAACTGGGTGGAAAACGTCATGGTGCAGGAGCTGGAGCAGGCGTGTCCCCCCTTTCGGCTGTGCCTCCATAAGCGGGACTTTGTGCCCGGGAAGTGGATTGTGGATAACATCATCGACTCCATTGAGAAGAGCCACAAAACGCTCTTCGTGCTGTCGGAGCACTTTGTGCAGAGCGAGTGGTGCAAGTACGAGCTGGACTTCTCGCACTTCCGCCTATTTGACGAGAACAACGATGCGGCCATTCTCATCCTCCTGGAGCCCATCCAGAGCCAAGCTATTCCCAAGAGGTTCTGCAAACTGCGGAAGATAATGAACACAAAGACCTACCTGGAGTGGCCTCGTGAGGAAGAGCAGCAGGAGATGTTTTGGGAAAACTTGAAAGTAGCTCTAAAATCCTAG

>TgutTLR2A Taeniopygia guttata Zebra Finch ENSTGUG00000005179 2382 ATGTCCATCCAAGCAAAACACAAATCAAGAATGACAAACACCTGGCGAGTGTTGGCCATCTACGTGATCTTAGCTGCAAGTCTCTCTGAGCAACAAGCACTGAAGCAGGCTTGTCCTTCATGTGATGGCAGTCAGCTTTGTAACTGCTCCTCCATGGGCTTGGACTTCATTCCCCCTGGGGTCACAGCCATAATTACAGTGTTAAACCTGGCCCACAACGGGATAAAGCGCATCCAATCCCAGGACCTGCAGCAGGCTGTGAACCTGAGAGCCCTGCTGCTGCAGTCCAACAAAATCAGCTCCATAGATGAGGACTCATTTTGGTCCCTGGAAAAACTGGAGCTCTTGGACTTATCAAATAACAGCTTGGCTCACTTGTCCCCTGTGTGGTTTGGGCACCTTTTTTCACTCCAGCACCTCCATCTTGAAGGCAATTCCTACAGAGACCTGGGGCAGAGCTCCCCCTTTTCTAGCCTGAAGAACTTGAGCTCTCTCCACCTGGGCAACCCGCAGTTCTCTGTGATTCGGCATGGGAACTTTGAGGGTATTGAGCTTCTGCACAAGTTGTGGATTGATGGTAGCAATCTCAGTCAGTATGAGCAGGGAAGTTTGAAATCAATTAAGCAGATCAATCACATGATCCTAAACCTAAGAAATGGTTATATATTCTCAGAAATTGTTAGGGACCTTCTGCACTCTGTCACTTGGCTGGAAGTGACAGAAATCAAATTACCAGTTGAAAGAAAAAGCTTGGTGCAGAATTCTACACGTCCTTTTAGGATACAAAAACTTACGTTTAAAGAAGCTTTCTTCACGGATGAAACTATTAGCCGAACAATAGTGTCACTGAAGGAAATCACCTCTTTAAAAGAGTTAGAGGCAATTAATTGTGTGCTTGAGGGGAAGGGAATATGGAACACTAAAGAAATTGCAAGGAGTGGGCAAAGTTTTGTTGAAACAGTAACAGTAACAAAGATAATGATTCAGAATTTTCATTTGTTTTTTGACCTGGAAGGTATGGAGTCACAAATAAACAAACTAAAAAGACTCAGTATCGCAAGCTCTAATGTTTTCATGGTACCGTGCAAACTTGCAAGACATTTTTCATCATTGCTGTATCTGGACTTTCATGATAATTTGCTTGTAAATAAGCGTTTAGATGAGACAATCTGTGAACATTCTTGGCCTTCATTGCAAACTTTAAATCTAAGTCAGAACTCTCTTAAATCTCTGGAACAGACTGCAAAGTATATATCTCGTCTACCAAAATTGAATAATCTTGACATTAGCCAAAATAATTTTGGTGAGATTCCAGATGTGTGTGACTGGCCAAAATCCCTGAAATATTTAAACCTGTCCAGCACTCAAATTCCCAGAGTAACCACCTGCATTCCTCAAACGCTAGAAGTTTTGGATGTTAGTGGAAACAACCTGAAGGAGTTTGGACTGCGGCTCCCACTTCTGAAAGAGCTGTACCTCACCAGAAACCAGCTGAAGACCCTGCCGGGTGCCGCACCCATTCCAAACTTGGTGTCCTTGTCCGTCAGCAGAAACAAGCTGAACAGTTTCTCCAAGGAGGAGTTTGAGTCCTTCAGGAGAATGAAGCTGCTGGATGCCAGTGGCAACAACTTCATCTGCTCCTGTGAGTTCCTCTCCTTCATCCACCACGAAGCTGGGATATCCCAAGTGCTGGTGGGGTGGCCGGACAAGTACGTGTGCGACTCTCCGCTGGCGGTGAGAGGGGCGCAGGTTGGCGCTGTGCACCTCTCCCTGATGGAGTGCCACAGGTCCCTGGTGGTGTCGTTGATCTGCGTCCTGGTGTTCCTGGTCATCCTGCTGCTGGTGGCCGTCGGCTACAAGTACCACATGGTCTGGTACCTGCGGATGACGTGGGCATGGCTGCAAGCCAAGCGGAAGCCCAAGCGCGCCCCGCCGAAGGACGTCTGCTACGACGCTTTTGTCTCCTACAGCGAGAACGACTCCGACTGGGTGGAGAACACCATGGTGCGGGAGCTGGAGCAGGCCTGCCCTCCCTTCCGGCTCTGCCTGCACAAGCGGGACTTTGTGCCGGGGAAGTGGATTGTGGACAACATCATCGATTCCATTGAGAAGAGCCGTAAAACGCTCTTTGTGCTGTCCGAGCACTTTGTGCAGAGTGAGTGGTGCAAATACGAGCTGGACTTCTCGCATTTCCGCCTCTTTGATGAGAACAACGATGCGGCGATTCTCGTCCTCCTGGAGCCCATCCAGAGCAAAGCCATTCCCAAGAGGTTCTGCAAGCTGCGGAAGATCATGAACACCAAGACCTACCTGGAGTGGCCTGCTGGTGAAGAGCAGCAGCAGGTGTTTTGGGAAAACTTGAAAGGAGCCTTGAAGTCATAG

>TgutTLR2B Taeniopygia guttata Zebra Finch chr4:27,734,003-27,736,354 (taeGut1) 2352 ATGACTGCACACATCTGGCGAGTGTTGGCCATCTACGTGATCTTAGCTGCAAGTCTCTCTGAGCAACAAGCACTGAAGCAGGCTTGTCCTTCATGCGATGGCAGTCAGCTTTGTAACTGCTCCTCCATGGGCTTGGACTTCATTCCCCCTGGGGTCACAGCCAAAATTACAGTGTTAAACCTGGCCCACAACAGGATAAAGCGCATCCAATCCCAGGACCTGCAGCAGGCTGTGAACCTGAGAGCCCTGCTGCTGCAGTCCAACAAAATCAGCTCCATAGATGAGGACTCATTTTGGTCCCTGGAAAAACTGGAGCTCTTGGACTTATCAAATAACAGCTTGGCTCACTTATCCCCTGTGTGGTTTGGGCACCTTTTTTCACTCCAGCACCTCCATCTTGAAGGCAATTCCTACAGAGACCTGGGGCAGAGCTCCCCCTTTTCTAGCCTGAAGAACCTGAGCTCTCTCCACCTGGGCAACCCGCAGTTCTCTGTGATTCGGCATGGGAACTTTGAGGGTATTGAGCTTCTGCACAAGTTGTGGATTGATGGTAGCAATCTCAGTCAGTATGAGCAGGGAAGTTTGAAATCAATTAAGCAGATCAATCACATGATCCTAAACCTAAGAAATGGTTATATATTCTCAGAAATTGTTAGGGACCTTCTGCACTCTGTCACTTGGCTGGAAGTGAGAAGAATAGCATTCAGTATTGCAGCAGAAATGCAAGTATTGAGAGTCATGTCTTCATCTTTTGCAAAGAAAATTTCTTTTAGACAGACATTACTAACAGATGCTACTGTGCCTGAGATTGTCAGTATTTTAGAAGACATGCCACAATTAGTGGAGCTGGAGCTGGTAGACTGTAGACTCTTGGGAACTGGACAATGGAAAATGGAAATTCAAGCAAAGAAATCACAGACACTTAGAATTTTAACAATAAAGAAATTATCTATAGAAGAATTTTACTTGTTTACAGATCTTCACTCTGTGGAAGGTCTACTGTCTCTTTTAACAAGAGTCACAGTCCAAAACACCAAAGTTTTTTTGGTACCCTGCAGAATTTCCCAAAACCTTCTGTCATTAGTATATCTTGACCTTAGTGCAAATTTGCTTGGAGACTTGAGTTTAGAACATTCAGCCTGTCAGGGTGGCTGGCCATCACTACAAGCTCTAAATTTAAGTCAGAACTCACTGAGTGACTTAGAAAGGACAAGTAAAAGTTTATCTCATCTAGGAAACCTAATTGTTTTGGACATTAGCCAAAATAATTTTGGTGAGATTCCAGATGTGTGTGACTGGCCAAAATCCCTGAAATATTTAAACCTGTCCAGCACTCAAATTCCCAAAGTAACAACCTGCATTCCTCAAACGCTAGAAGTTTTGGATGTTAGTGGAAACAACCTGAAGGAGTTTGGACTGCGGCTCCCACTTCTGAAAGAGCTGTACCTCACCAGAAACCAGCTGAAGACCCTGCCGGGTGCCGCACCCATTCCAAACTTGGTGTCCTTGTCCGTCAGCAGAAACAAGCTGAACAGTTTCTCCAAGGAGGAGTTTGAGTCCTTCAGGAGAATGAAGCTGCTGGATGCCAGTGGCAACAACTTCATCTGCTCCTGTGAGTTCCTCTCCTTCATCCACCACGAAGCTGGGATATCTCAAGTGCTGGTGGGGTGGCCGGACAAGTACGTGTGTGACTCTCCGCTGGCGGTGAGAGGGGCGCAGGTTGGCGCTGTGCACCTCTCCCTGATGGAGTGCCACAGGTCCCTGGTGGTGTCGTTGATCTGCGTCCTGGTGTTCCTGGTCATCCTGCTGCTGGTGGCCGTCGGCTACAAGTACCACATGGTCTGGTACCTGCGGATGACGTGGGCATGGCTGCAAGCCAAGCGGAAGCCCAAGCGCGCCCCGCCGAAGGACGTCTGCTACGACGCTTTTGTCTCCTACAGCGAGAACGACTCCGACTGGGTGGAGAACACCATGGTGCGGGAGCTGGAGCAGGCCTGCCCTCCCTTCCGGCTCTGCCTGCACAAGCGGGACTTTGTGCCAGGGAAGTGGATTGTGGACAACATCATCGATTCCATTGAGAAGAGCCGCAAAACACTCTTTGTGCTGTCCGAGCACTTTGTGCAGAGTGAGTGGTGCAAATATGAGCTGGACTTCTCGCATTTCCGCCTCTTTGATGAGAACAATGATGCGGCGATTCTCGTCCTCCTGGAGCCCATCCAGAGCAAAGCCATTCCCAAGAGGTTCTGCAAGCTGCGGAAGATCATGAACACCAAGACCTACCTGGAGTGGCCTCTGGAAGAAGAGCAGCAGCAGATGTTTTGGTTTAATTTGAAAATCGCTCTAAGATCCTAG

>BbisTLR2 Bison bison American bison EU580539 2355 ATGCCACGTGCTTTGTGGACAGCATGGGTCTGGGCTGTCATCATCCTGTCCACGGAAGGAGCCTCTGACCAGGCTTCTTCTCTGTCTTGTGACTCAACTGGTGTCTGCGATGGCCATTCCAGATCTTTAAACTCCATCCCCTCTGGTCTCACGGCAGGTGTGAAAAGCCTTGACCTGTCCAACAACGAGATCACCTATGTCGGCAACAGAGACCTGCAGAGGTGTGTGAACCTGAAGACTCTGAGGCTGGGGGCCAATGAAATTCACACAGTGGAGGAAGATTCTTTTTTTCACCTGAGGAATCTTGAATATTTGGACTTATCCTATAATCGCTTATCTAACTTATCATCCTCCTGGTTCAGGTCCCTTTATGTCTTGAAATTCTTAAACTTACTGGGAAATTTATACAAAACACTTGGGGAAACATCTCTTTTTTCTCATCTCCCAAATCTGCGGACCCTAAAAGTAGGAAATAGTAACAGCTTCACTGAGATTCATGAAAAGGATTTCACTGGACTGACTTTTCTTGAGGAGCTTGAGATCAGTGCTCAAAATCTGCAGATATATGTGCCAAAGAGTTTAAAGTCAATCCAGAACATTAGCCATCTGATTCTACATCTGAAGCAGCCTGTTTTACTCGTGGACATTCTTGTAGATATTGTAAGTTCCTTAGATTGTTTAGAACTGAGAGATACTAATTTGCACACTTTCCATTTTTCAGAAGCATCCATCAGTGAAATGAGTACATCGGTTAAAAAGCTTATATTTAGAAATGTGCAATTCACCGATGAAAGTTTTGTTGAAGTTGTCAAACTGTTTAACTATGTTTCTGGGATCTTAGAAGTAGAGTTTGATGACTGTACCCATGATGGAATTGGCGATTTTAGAGCACTGAGTTTGGACAGAATTAGACACCTAGGTAATGTGGAGACGTTAACAATACGGAAGTTGCATATTCCACAGTTTTTCTTATTTCATGATCTGAGTAGTATATATCCACTCACAGGCAAAGTTAAAAGAGTCACAATAGAAAACAGTAAGGTTTTTCTGGTTCCTTGTTTACTTTCACAACATTTAAAATCATTAGAATATTTGGATCTCAGTGAAAACTTAATGTCTGAAGAAACCTTGAAAAACTCAGCCTGTAAGGATGCCTGGCCCTTCCTTCAAACCTTGGTTTTAAGGCAGAATCGTTTGAAATCACTAGAAAAAACGGGAGAACTTTTGCTTACTCTGGAAAATCTGAATAGCCTTGATATCAGTAAGAATAATTTTCTTTCAATGCCTGAAACTTGTCAGTGGCCAGGCAAAATGAAACAGCTGAACTTATCCAGCACAAGGATACACAGTTTAACCCAGTGCCTTCCCCAGACCCTGGAAATTTTAGATGTTAGCAATAACAATCTCGATTCATTTTCTTTGATTTTGCCGCAACTCAAAGAACTTTATATTTCCAGAAATAAGTTGAAGACTCTACCAGATGCCTCCTTCTTACCCGTGTTATCAGTTATGAGAATTAGCAGAAATATAATAAATACTTTCTCAAAGGAACAACTTGATTCTTTTCAGCAACTGAAGACGTTGGAGGCCGGTGGCAACAACTTCATTTGCTCCTGTGACTTCCTGTCCTTCACACAGGGACAGCAGGCACTGGGCCGTGTCCTGGTCGACTGGCCCGATGACTACCGCTGTGACTCTCCCTCCCACGTGCGGGGCCAGCGGGTGCAGGACGCCCGGCTCTCCCTTTCTGAATGCCACAGGGCGGCCGTGGTGTCCGCCGCGTGCTGTGCCCTCTTCCTTGTTCTCCTGCTCACGGGGGTGCTGTGTCACCGTTTCCACGGACTGTGGTACATGAAGATGATGTGGGCGTGGCTGCAGGCCAAGAGGAAGCCCAGGAAGGCTCCCCGCAGGGACATCTGCTACGACGCCTTCGTGTCCTACAGCGAGCGGGATTCCTACTGGGTGGAGAACCTCATGGTGCAGGAGCTGGAGCAGTTCAACCCTCCCTTTAAGCTGTGTCTTCACAAGCGAGACTTCATTCCTGGCAAGTGGATCATCGACAACATCATTGACTCCATTGAAAAGAGCCACAAAACCATCTTTGTGCTTTCGGAGAACTTTGTGAAGAGCGAGTGGTGCAAGTATGAGCTGGACTTCTCCCATTTCCGTCTCTTTGATGAGAACAATGATGCTGCCATTCTGATTCTGCTGGAGCCCATTGACAAGAAGGCCATTCCCCAGCGCTTCTGTAAGCTGCGGAAGATCATGAACACCAAGACCTACCTGGAGTGGCCCGTGGATGAGACTCAGCAGGAAGGGTTTTGGTTAAATTTGAGAGCTGCAATAAGGTCCTAG

>DpygTLR2 Damaliscus pygargus phillipsi Blesbok EU580541 2352 ATGCCACATGCTTTGTGGACAGCGTGGGTCTGGGCTGTAATCAGCGTGTCCACGGAAGGAGCCTCTGTGGCTTCTTCTCTGTCTTGTGACCCAACTGGTGTCTGCGATGGCCATTCCAGATCTTTAAACTCCATCCCCTTTGGTCTCACGGAAGGTGTGAAAAGCCTTGACCTGTCCAACAACGAGATCACCTATGTCAGCAACAGAGACCTGCAGAGGTGTATGAACCTGAAGACTCTGAGGCTGGGGGGCAATGAAATTCACACGGTGGAGGAAGATTCTTTTTTTCACCTGAGGAATCTTGAATATTTGGACTTATCCTATAATCGCTTATCTAACTTATCATCCTCCTGGTTCAGGTCCCTTTATGTCTTAAAATTCTTAAACTTACTGGGAAATTTATACAAAACACTTGGGGAAACATCCCTTTTTTCTCATCTCCCAAATCTGCGGACCCTGAAACTAGGAAATAGTAACAGCTTCACTCAGATTCATGAAAAGGATTTCACTGGACTGACTTTTCTTGAGGAGCTTGAGATCAGTGCTCAAAATYTGCAGATATATGCGCCAAAGAGTTTAAAGTCAATCCACAACATTAGCCATCTGATTCTTCATCTGAAGCAGCCTGTTTTACTCCTGGACATTCTTGTAGATATTGTAAGTTCCTTAGATTATTTAGAACTGAGAGATACTAATTTGCACACTTTCTATTTTTCAGAAGCATCCATCAGTGAAATTAATACATCAGTTAAAAAGCTTATATTTAGAAATGTGCAATTCACCGATGAAAGTTTTGTTGAAGTTGTCAAACTGTTTAACTATGTTTCTGGGATCTTAGAAGTAGAGTTTGATGACTGTACCCATGATGGAATTGGCGATTTTAGAGCACTGACTTTTAACAGAATTAGATACCTAGGTAACGTGGAGACGTTAACAATACGGAAGTTGCATATCCCACAGTTTTTCTTATTTCATGATCTGAGTAGTATATATCCACTCACAGGTAAAGTTAAAAGAGTCACAATAGAAAACAGTAAGGTTTTCCTGGTTCCTTGTTTACTTTCACAACATTTAATATCGCTAGAATATTTGGATCTCAGTGAAAACTTAATGTCTGAAGAAACCTTGAAAAACTCAGCCTGTGAGCATGCCTGGCCCTTCCTTCAAACCCTGGTTTTAAGGCAGAATCGTTTGAAATCACTAGAAAAAACTGGAGAACTTTTGCTTACTCTGAAAAATCTGAATAACCTTGATATCAGTAAGAATAATTTTCTTTCAATGCCTGAAACTTGTCAGTGGCCAGGAAAAATGAAACAGTTGAACTTATCCAGCACGAGGATACACAGTTTAACCCAGTGCCTTCCCCAGACCCTGGAAATTTTAGATGTTAGCAATAACAATCTCGATTCATTTTCTTTGATTTTGCCGCAACTCAAAGAACTGTATATTTCCAGAAATAAGTTGAAGACTCTACCAGATGCCTCCTTCTTACCCGTGTTATCAGTTATGAGAATTAGCGGAAATATAATAAATACTTTCTCGAAGGAACAACTTGATTCTTTTCCACAACTGAAGGCTTTGGAGGCCGGTGGCAACAACTTCATTTGCTCCTGTGACTTCCTGTCCTTCACACAGGGACAGCAAGCACTGGCCCGTGTCCTGGTCGACTGGCCAGATGGCTACCGCTGTGACGCTCCCTCGCACGTGCGGGGCCAGCGGGTGCAGGACGCCCGGCTCTCCCTTTCTGAATGCCACCGGGCGGCCGTGGTGTCTGCCGTGTGCTGTGCCCTTTTCCTGTTGCTCCTGCTCACGGGGGCGCTGTGTCACCGTTTCCACGGGCTGTGGTACATGAAGATGATGTGGGCCTGGCTCCAGGCCAAGAGGAAGCCCAGGAAGGCTCCCCGCAGGGACCTCTGCTACGACGCCTTTGTGTCCTACAGTGAGCGGGATTCCTACTGGGTGGAGAACCTCATGGTCCAGGAGCTGGAGCACTTCAACCCCCCCTTTAAGCTGTGTCTTCATAAGCGAGACTTCGTTCCTGGCAAGTGGATTATCGACAACATCATTGACTCCATTGAGAAGAGCCACAAAACCATCTTTGTGCTTTCGGAGAACTTTGTGAAGAGCGAGTGGTGCAAGTATGAGCTGGACTTCTCCCATTTCCGTCTCTTTGATGAGAACAATGATGCTGCCATTCTGATTCTGCTGGAGCCCATTGACAAGAAGGCCATTCCCCAGCGCTTCTGTAAGCTGCGGAAGATCATGAACACCAAGACCTACCTGGAGTGGCCCACGGATGAGACTCAGCAGGAAGCGTTTTGGTTAAATTTGAGAGCTGCAATAAGGTCCTAG

>BtraTLR2 Boselaphus tragocamelus Nilgai DQ286731 2355 ATGCCACGTGCTTTGTGGCCAGCGTGGGTCTGGGCTATAATCATCCTGTCCATGGAAGGAGCCTCTGATAAGGCTTCTTCTCTGTCTTGTGACCCAACTGGTGTCTGCGATGGCCGTTCCAGATCTTTAAACTCCATCCCCTCTGGTCTCACGGCAGGTGTGAAAAGCCTTGACCTGTCCAACAATGAGATCACCTATGTCGGCAACAGAGACCTGCAGAGGTGTGTGAACCTGAAGACTCTGAGGCTGGGGGCCAATGAAATTCACACAGTGGAGGAAGATTCTTTTTTTCACCTAAGGAATCTTGAATATTTGGACTTATCCTATAATCGCTTATCTAACTTATCATCCTCCTGGTTCAGGTCCCTTTATGTCTTGAAATTCTTAAACTTACTGGGAAATTTATACAAAACACTTGGGGAAACATCTCTTTTTTCTCATCTCCCAGATCTGCGGACCCTGAAAGTAGGAAATAGTAACAGCTTCACTGAGATTCATGAAAAGGATTTCACTGGACTGACTTTTCTTGAGGAGCTTGAGATCAGTGCTCAAAATCTGCAGATATATGTGCCAAAGAGTTTAAAGTCAATCCAGAACATTAGCCATCTGATTCTACATCTGAAGCAGCCTGTTTTACTCGTGGACATTCTTGTAGATATTGTAAGTTCCTTAGATTGTTTAGAACTAAGAGATACTAATTTGCACACTTTCCATTTTTCAGAAGCATCCATCAGTGAAATGAGTACATCGGTTAAAAAGCTTATATTTAGAAATGTGCAATTCACCGATGAAAGTTTTGTTGAAGTTGTCAAACTGTTTAACTATGTTTCTGGGATTGTAGAAGTAGAGTTTGATGACTGTACCCATGATGGAATTGGCGATTTTAGAGCACTGAGTTTGGACAGAATTAGACACCTAGGTAACGTGGAGACGTTAACAATACGGAAGTTGCATATTCCACAGTTTTTCTTATTTCAGGATCTGAGTAGTATATATCCACTCACAGGCAAAGTTAAAAGAGTCACAATAGAAAACAGTAAGGTTTTTCTGGTTCCTTGTTTACTTTCACAACATTTAAAATCATTAGAATATTTGGATCTCAGTGAAAACTTAATGTCTGAAGAAACCTTGAAAAACTCAGCCTGTAAGGATGCCTGGCCCTTCCTTCAAACCTTGGTTTTAAGGCAGAATCGTTTGAAATCACTAGAAAAAACGGGAGAACTTTTGCTTACTCTGAAAAATCTGAATAACCTTGATATCAGTAAGAATAATTTTCTTTCAATGCCTGAAACTTGTCAGTGGCCAGGAAAAATGAAACAGTTGAACTTATCCAGCACAAAGATACGCAGTTTAACCCAATGCCTTCCCCAGACCCTGGAAATTTTAGATGTTAGCAATAACAATCTCGATTCATTTTCTTTGATTTTGCCGCAACTCAAAGAACTTTATATTTCCAGAAATAAGTTGAAGACTCTACCAGATGCCTCCTTCTTACCCGTGTTATCAGTTATGGGAATTAGCAAAAATATAATAAATACCTTCTCGAAGGAACAACTTGATTCTTTTCAGCAGCTGAAGACTTTGGAGGCCGGTGGCAACAACTTCATTTGCTCTTGTGACTTCCTGTCCTTCACACAGGGACAGCAGGCACTGGGCCGTGTCCTGGTCGACTGGCCCGATGACTACCGCTGTGACTCGCCCTCCCACGTGCGGGGCCAGCGGCTGCAGGACGCCCGGCTCTCCCTTTCTGAATGCCACAGGGCGGCCGTGGTGTCCGCCGCGTGCTGTGCCCTCTTTCTGTTTCTCTTGCTCACGGGGGTGCTGTGTCACCGTTTCCACGGACTGTGGTACATGAAGATAATGTGGGCCTGGCTCCAGGCCAAGCGGAAGCCCAGGAAGGCTCCCCGCAGGGACATCTGCTACGACGCCTTTGTGTCCTACAGTGAGCGGGATTCCTACTGGGTGGAGAACCTCATGGTCCAGGAGCTGGAGCACTTCAACCCTCCCTTTAAGCTGTGTCTTCATAAGCGAGACTTCATTCCTGGCAAGTGGATTATCGACAACATCATTGACTCCATTGAAAAGAGCCACAAAACCATCTTTGTGCTTTCGGAGAACTTTGTGAAGAGCGAGTGGTGCAAGTATGAGCTGGACTTCTCCCATTTCCGTCTCTTTGATGAGAACAATGACGCTGTCATTCTGATTCTGCTGGAGCCCATTGACAAGAAGGCCATTCCCCAGCGCTTCTGTAAGCTGCGGAAGATCATGAACACCAAGACCTACCTGGAGTGGCCCCTGGACGAGACTCAGCAGGAAGGGTTTTGGTTAAATTTGAGAGCTGCAATAAGGTCCTAG

>BbubTLR2 Bubalus bubalis Water Buffalo DQ288130 2355 ATGCCACGTGCTTTGTGGACAGCGTGGGTCTGGGCTGTAATCATCCTGTCCATGGAAGGAGCCTCTCATCAGGCTTCTTCTCTGTCTTGTGACCCAACTGGTGTCTGTGATGGCCATTCCAGATCTTTAAACTCCATCCCCTCTGGTCTCACAGACGGTGTGAAAAGCCTTGACCTGTCCAACAACGAGATCACCTATGTCAGCAACAGAGACCTGCAGAGGTGTGTGAACCTGAAGACTCTGAGGCTGGGGGCCAATGAAATTCACACAGTGGAGGAAGATTCTTTTTTTCACCTGAGGAATCTTGAATATTTGGATTTATCCTATAATCGCTTATCTAACTTATCATCCTCCTGGTTCAGGTCCCTTTATGCCTTGAAATTCTTAAACTTACTGGGAAATGTATACAAAACACTTGGGGAAACATCTCTTTTTTCTCATCTCCCAAATCTGCGGACCCTGAAAGTAGGAAATAGTAACAGCTTCACTGAGATTCATGAAAAGGATTTCACTGGACTCATTTTTCTTGAGGAGCTTGAGATCAGTGCTCAAAATCTGCAGATATATGTGCCAAAGAGTTTAAAGTCAATCCAGAACATTAGCCATCTGATTCTACATCTGAAGCAGCCTGTTTTACTCGTGGACATTCTTGTAGATATTGTAAGTTCCTTAGATTGTTTAGAACTGAGAGATACTAATTTGCACACTTTCCATTTTTCAGAAGCATCCATCAGTGAAATGAATACATCTGTTAAAAAGCTTATATTTAGAAATGTGCAATTCACCGATGAAAGTTTTGTCGAAGTTGTCAAACTGTTTAACTATGTTTCTGGGATCTTAGAAGTAGAGTTTGATGACTGTACCCATGATGGAATTGGCGATTTTAGAGCACTGAGTTTGGACAGAATTAGACACCTAGGTAACGTGGAGACGTTAACAATACGGAAGTTGCATATTCCACAGTTTTTCTTATTTCATGATCTCAGTAGTATATATCCACTCACAGGCAAAGTTAAAAGAGTCACAATAGAAAGCAGTAAGGTTTTTCTGGTTCCTTGTTTACTTTCACAACATTTAAAATCATTAGAATATTTGGATCTCAGTGAAAACTTAATGTCTGAAGAAACCTTGAAAAACTCAGCCTGTAAGGATGCCTGGCCCTTCCTTCAAACCTTGGTTTTAAGGCAGAATCGTTTGAAATCACTAGAAAAAACGGGAGAACTTTTGCTTACTCTGGAAAATCTGAATAACCTTGATATCAGTAAGAATAATTTTCTTTCAATGCCTGAAACTTGTCGGTGGCCAGGAAAAATGAAACAGTTGAACTTATCCAGCACAAGGGTACACAGTTTAACCCAGTGCCTTCCCCAGACCCTGGAAATTTTAGATGTTAGCAATAACAATCTCGATTCATTTTCTTTGATTTTGCCACAACTCAAAGAACTCTATATTTCCAGAAATAAGTTGAAGACTCTACCAGATGCCTCCTTCTTACCCGTGTTATCAGTTATGAGAATTAGCAGAAATATAATAAATACTTTCTCAAAGGAACAACTTGATTCTTTTCAGCAGCTGAAGACTTTGGAGGCCGGTGGCAACAACTTCATTTGCTCCTGTGACTTCCTGTCCTTCACACAGGGACAGCAGGCGCTGGGCCGTGTCCTGGTCGACTGGCCCGCTGAGTACCGCTGTGACTCTCCCTCCCATGTGCGGGGCCAGCGGGTGCAGGACGCCCGGCTCTCCCTTTCTGAATGCCACAGGGCGGCTGTGGTGTCCGCCGCGTGCTGTGCCCTCTTCCTGTTGCTCCTGCTCACGGGGGTTCTGTGTCACCGTTTCCATGGACTGTGGTACATGAAGATGATGTGGGCCTGGCTCCAGGCCAAGAGGAAGCCCAGGAAGGCTCCCCGCAGGGACATCTGCTACGATGCCTTCGTGTCCTACAGCGAGCAGGATTCCTACTGGGTGGAGAACCTCATGGTCCAGGAGCTGGAGCACTTCAACCCTCCCTTTAAGCTGTGTCTTCATAAGCGAGACTTCATTCCTGGCAAGTGGATTATCGACAACATCATTGACTCCATTGAAAAGAGCCACAAAACCATCTTTGTGCTTTCGGAGAACTTTGTGAAGAGCGAGTGGTGCAAGTATGAGCTGGACTTCTCCCATTTCCGTCTCTTTGATGAGAACAATGATGCTGCCATTCTGATTCTGCTGGAGCCCATTGACAAGAAGGCCATTCCCCAGCGCTTCTGTAAGCTGCGGAAGATCATGAACACCAAGACCTACCTGGAGTGGCCCGTGGATGAGACTCAGCAGGAAGGGTTTTGGTTAAATTTGAGAGCTGCAATAAGGTCCTAG

>OariTLR2 Ovis aries Sheep DQ890157 2355 ATGCCACGTGCTTTGTGGACAGCGTGGGTCTGGGCTGTAATCAGCGTGTTCACGGAAGGAGCCTCTGATCAGGCTTCTTCTCTGTCTTGTGACCCAACTGGTGTCTGCGATGGCCATTCCAGATCTTTAAACTCCATCCCCTCTGGTCTCACGGCAAGTGTGAAAAGCCTTGACCTGTCCGACAACGAGATCACCTATGTCGGCAACAGAGACCTGCAGAGGTGTGTGAATCTGAAGACTCTGAGGCTGGGGGCCAATGAAATTCACACGGTGGAGGAAGATTCTTTTTTTCACCTGAGGAATCTTGAATATTTGGACTTATCCTATAATCGCTTATCTAACTTATCATCTTCCTGGTTCAGGTCCCTTTATGTCTTGAAATTCTTAAACTTACTGGGAAATTTATACAAAACACTTGGGGAAACATCTCTTTTTTCTCATCTCCCAAATCTGCGGACCCTGAAAGTAGGAAATAGTAACAGCTTCACTCAGATTCATGAAAAGGATTTCACTGGACTGACTTTTCTTGAGGAGCTTGAGATCAGTGCTCAAAATCTGCAGTTGTATGTGCCAAAGAGTTTAAAGTCGATCCAGAACATTAGCCATCTGATTCTGCATCTGAGGCAGCCTGTTTTACTCCTGGACATTCTTATAGATATTGTAAGTTCCTTAGATTATTTAGAACTGAGAGATACTAATTTGCACACTTTCTATTTTTCAGAAGCATCCATCAGTGAAATTAATACATCAGTTAAAAAGCTTACATTTAGAAATGTGCAATTCACCGATGACAGTTTTGTTGAAGTTGTCAAACTGTTTAACTATGTTTCTGGGATCTTAGAAGTAGAGTTTGATGACTGTACCCATGATGGAGTTGGCGATTTTACAGCACTGACTTTGAACAGAATTAGATACCTAGGTAACGTGGAGACGTTAACAATACGGAAGTTGCATATCCCACAGTTTTTCTTATTTTATGATCTGAGTAGTATATATCCACTCACAGGCAAAGTTAAAAGAGTCACAATAGAGAACAGTAAGGTTTTCCTGGTTCCTTGTTTACTTTCACAACATTTAAAATCGCTAGAATATTTGGATCTCAGTGAAAACTTAATGTCTGAAGAAACCTTGAAAAACTCAGCCTGTGAGCATGCCTGGCCCGTCCTTCAAACCCTGGTTTTAAGGCAGAATCGTTTGAAATCACTAGAAAAAACTGGAGAACTTTTGCTTACTCTGAAAAATCTGAATAACCTTGATATCAGTAAGAATAATTTTCTTTCAATGCCTGAAACTTGTCAGTGGCCAGGAAAAATGAAACAGTTGAACTTATCCAGCACGAGGATACACAGTTTAACCCAGTGCCTTCCCCAGACCCTGGAAATTTTAGATGTTAGCAATAATAATCTCGATTCATTTTCTTTGATTTTGCCGCAACTCAAAGAACTGTATATTTCCAGAAATAAGTTGAAGACTCTACCAGATGCCTCCTTCTTACCCGTGTTATCAGTTATGAGAATTAGCGGAAATATAATAAATACTTTCTCGAAGGAACAACTTGATTCTTTTCCACAACTGAAGGCTTTGGAGGCCGGTGGCAACAACTTCATTTGCTCCTGTGACTTCCTGTCCTTCGCACAGGGACAGCAGGCACTGGCCCGTGTCCTGGTCGACTGGCCAGATGGCTACCGCTGTGACGCTCCCTCGCACGTGCGGGGCCAGCGGGTGCAGGATGCCCGCCTCTCCCTTTCTGAATGCCACCGGGCGGCCGTGGTGTCCGCCGTGTGCTGTGCCCTCTTCCTGTTGCTCCTGCTCACGGGGGTGCTGTGTCACCGTTTCCACGGGCTGTGGTACATGAAGATGATGTGGGCCTGGCTCCAGGCCAAGAGGAAGCCCAGGAAGGCTCCCCGCAGGGACCTCTGCTACGACGCCTTTGTGTCCTACAGCGAGCGGGATTCCTACTGGGTGGAGAACCTCATGGTCCAGGAGCTGGAGCACTTCAACCCTCCCTTTAAGCTGTGTCTTCATAAGCGAGACCTTGTCCCTGGCAAATGGATTATCGACAACATCATCGACTCCATTGAAAAGAGCCGCAAAACCATCTTTGTGCTTTCGGAGAGCTTTGTGAGGAGCGAGTGGTGCAAGTATGAGCTGGACTTCTCCCACTTCCGTCTCTTTGATGAGAACAACGATGCTGCCATTCTGATTCTGCTGGAGCCCATTGACAAGAAGGCCGTTCCCCAGCGCTTCTGTAAGCTGCGGAAGATCATGAACACCAGGACCTACCTGGAGTGGCCCACGGATGAGACTCAGCAGGAAGCATTCTGGTTGAATTTGAGAGCTGCAATAAGGTCCTAG

>AmarTLR2 Antidorcas marsupialis Springbok EU580538 2352 ATGCCACATGCTTTGTGGACAGCGTGGGTCTGGGCTGTAATCAGCGTGTCCACGGAAGGAGCCTCTGTGGCTTCTTCTCTGTCTTGTGACCCAACTGGTGTCTGCGATGGCCATTCCAGATCTTTAAACTCCATCCCCTTTGGTCTCACGGAAGGTGTGAAAAGCCTTGACCTGTCCAACAACGAGATCACCTATGTCAGCAACAGAGACCTGCAGAGGTGTGTGAACCTGAAGACTCTGAGGCTGGGGGGCAATGAAATTCACACGGTGGAGGAAGATTCTTTTTTTCACCTGAGGAATCTTGAATATTTGGACTTATCCTATAATCGCTTATCTAACTTATCATCCTCCTGGTTCAGGTCCCTTTATGTCTTAAAATTCTTAAACTTACTGGGAAATTTATACAAAACACTTGGGGAAACATCCCTTTTTTCTCATCTCCCAAATCTGCGGACCCTGAAACTAGGAAATAGTAACAGCTTCACTCAGATTCATGAAAAGGATTTCACTGGACTGACTTTTCTTGAGGAGCTTGAGATCAGTGCTCAAAATCTGCAGATATATGCGCCAAAGAGTTTAAAGTCAATCCACAACATTAGCCATCTGATTCTTCATCTGAAGCAGCCTGTTTTACTCCTGGACATTCTTGTAGATATTGTAAGTTCCTTAGATTATTTAGAACTGAGAGATACTAATTTGCACACTTTCTATTTTTCAGAAGCATCCATCAGTGAAATTAATACATCAGTTAAAAAGCTTATATTTAGAAATGTGCAATTCACCGATGAAAGTTTTGTTGAAGTTGTCAAACTGTTTAACTATGTTTCTGGGATCTTAGAAGTAGAGTTTGATGACTGTACCCATGATGGAATTGGCGATTTTAGAGCACTGACTTTTAACAGAATTAGATACCTAGGTAACGTGGAGACGTTAACAATACGGAAGTTGCATATCCCACAGTTTTTCTTATTTCATGATCTGAGTAGTATATATCCACTCACAGGTAAAGTTAAAAGAGTCACAATAGAAAACAGTAAGGTTTTCCTGGTTCCTTGTTTACTTTCACAACATTTAATATCGCTAGAATATTTGGATCTCAGTGAAAACTTAATGTCTGAAGAAACCTTGAAAAACTCAGCCTGTGAGCATGCCTGGCCCTTCCTTCAAACCCTGGTTTTAAGGCAGAATCGTTTGAAATCACTAGAAAAAACTGGAGAACTTTTGCTTACTCTGAAAAATCTGAATAACCTTGATATCAGTAAGAATAATTTTCTTTCAATGCCTGAAACTTGTCAGTGGCCAGGAAAAATGAAACAGTTGAACTTATCCAGCACGAGGATACACAGTTTAACCCAGTGCCTTCCCCAGACCCTGGAAATTTTAGATGTTAGCAATAACAATCTCGATTCATTTTCTTTGATTTTGCCGCAACTCAAAGAACTGTATATTTCCAGAAATAAGTTGAAGACTCTACCAGATGCCTCCTTCTTACCCGTGTTATCAGTTATGAGAATTAGCGGAAATATAATAAATACTTTCTCGAAGGAACAACTTGATTCTTTTCCACAACTGAAGGCTTTGGAGGCCGGTGGCAACAACTTCATTTGCTCCTGTGACTTCCTGTCCTTCACACAGGGACAGCAAGCACTGGCCCGTGTCCTGGTCGACTGGCCAGATGGCTACCGCTGTGACGCTCCCTCGCACGTGCGGGGCCAGCGGGTGCAGGACGCCCGGCTCTCCCTTTCTGAATGCCACCGGGCGGCCGTGGTGTCTGCCGTGTGCTGTGCCCTTTTCCTGTTGCTCCTGCTCACGGGGGCGCTGTGTCACCGTTTCCACGGGCTGTGGTACATGAAGATGATGTGGGCCTGGCTCCAGGCCAAGAGGAAGCCCAGGAAGGCTCCCCGCAGGGACCTCTGCTACGACGCCTTTGTGTCCTACAGTGAGCGGGATTCCTACTGGGTGGAGAACCTCATGGTCCAGGAGCTGGAGCACTTCAACCCCCCCTTTAAGCTGTGTCTTCATAAGCGAGACTTCGTTCCTGGCAAGTGGATTATCGACAACATCATTGACTCCATTGAGAAGAGCCACAAAACCATCTTTGTGCTTTCGGAGAACTTTGTGAAGAGCGAGTGGTGCAAGTATGAGCTGGACTTCTCCCATTTCCGTCTCTTTGATGAGAACAATGATGCTGCCATTCTGATTCTGCTGGAGCCCATTGACAAGAAGGCCATTCCCCAGCGCTTCTGTAAGCTGCGGAAGATCATGAACACCAAGACCTACCTGGAGTGGCCCACGGATGAGACTCAGCAGGAAGCGTTTTGGTTAAATTTGAGAGCTGCAATAAGGTCCTAG

>BtauTLR2 Bos Taurus Cow AY634629 2355 ATGCCACGTGCTTTGTGGACAGCATGGGTCTGGGCTGTCATCATCCTGTCCACGGAAGGAGCCTCTGACCAGGCTTCTTCTCTGTCTTGTGACCCAACTGGTGTCTGCGATGGCCATTCCAGATCTTTAAACTCCATCCCCTCTGGTCTCACGGCAGGTGTGAAAAGCCTTGACCTGTCCAACAATGATATCACCTATGTCGGCAACAGAGACCTGCAGAGGTGTGTGAACCTGAAGACTCTGAGGCTGGGGGCCAATGAAATTCACACAGTGGAGGAAGATTCTTTTTTTCACCTGAGGAATCTTGAATATTTGGACTTATCCTATAATCGCTTATCTAACTTATCATCCTCCTGGTTCAGGTCCCTTTATGTCTTGAAATTCTTAAACTTACTGGGAAATTTATACAAAACACTTGGGGAAACATCTCTTTTTTCTCATCTCCCAAATCTGCGGACCCTAAAAGTAGGAAATAGTAACAGCTTCACTGAGATTCATGAAAAGGATTTCACTGGACTGACTTTTCTTGAGGAGCTTGAGATCAGTGCTCAAAATCTGCAGATATATGTGCCAAAGAGTTTAAAGTCAATCCAGAACATTAGCCATCTGATTCTACATCTGAAGCAGCCTATTTTACTCGTGGACATTCTTGTAGATATTGTAAGTTCCTTAGATTGTTTTGAACTGAGAGATACTAATTTGCACACTTTCCATTTTTCAGAAGCATCCATCAGTGAAATGAGTACATCGGTTAAAAAGCTTATATTTAGAAATGTGCAATTCACCGATGAAAGTTTTGTTGAAGTTGTCAAACTGTTTAACTATGTTTCTGGGATCTTAGAAGTAGAGTTTGATGACTGTACCCATGATGGAATTGGCGATTTTAGAGCACTGAGTTTGGACAGAATTAGACACCTAGGTAATGTGGAGACGTTAACAATACGGAAGTTGCATATTCCACAGTTTTTCTTATTTCATGATCTGAGTAGTATATATCCACTCACAGGCAGAGTTAAAAGAGTCACAATAGAAAACAGTAAGGTTTTTCTGGTTCCTTGTTTACTTTCACAACATTTAAAATCATTAGAATATTTGGATCTCAGTGAAAACTTAATGTCTGAAGAAACCTTGAAAAACTCAGCCTGTAAGGATGCCTGGCCCTTCCTTCAAACCTTGGTTTTAAGGCAGAATCGTTTGAAATCACTAGAAAAAACGGGAGAACTTTTGCTTACTCTGGAAAATCTGAATAACCTTGATATCAGTAAGAATAATTTTCTTTCAATGCCTGAAACTTGTCAGTGGCCAGGAAAAATGAAACAGCTGAACTTATCCAGCACAAGGATACACAGTTTAACCCAGTGCCTTCCCCAGACCCTGGAAATTTTAGATGTTAGCAATAACAATCTCGATTCATTTTCTTTGATTTTGCCACAACTCAAAGAACTTTATATTTCCAGAAATAAGTTGAAGACTCTACCAGATGCCTCCTTCTTACCCGTGTTATCAGTTATGAGAATTAGCAGAAATATAATAAATACTTTCTCAAAGGAACAACTTGATTCTTTTCAGCAACTGAAGACGTTGGAGGCCGGTGGCAACAACTTCATTTGCTCCTGTGACTTCCTGTCCTTCACACAGGGACAGCAGGCACTGGGCCGTGTCCTGGTCGACTGGCCCGATGACTACCGCTGTGACTCTCCCTCCCATGTGCGGGGCCAGCGGGTGCAGGACGCCCGGCTCTCCCTTTCTGAATGCCACAGGGCGGCCGTGGTGTCCGCAGCGTGCTGTGCCCTCTTCCTGTTGCTCCTGCTCACGGGGGTGCTGTGTCACCGTTTCCACGGACTGTGGTACATGAAGATGATGTGGGCGTGGCTGCAGGCCAAGAGGAAGCCCAGGAAGGCTCCCCGCAGGGACATCTGCTACGACGCCTTCGTGTCCTACAGCGAGCGGGATTCCTACTGGGTGGAGAACCTCATGGTCCAGGAGCTGGAGCACTTCAACCCTCCCTTTAAGCTGTGTCTTCATAAGCGAGACTTCATTCCTGGCAAGTGGATTATCGACAACATCATTGACTCCATTGAAAAGAGCCACAAAACCATCTTTGTGCTTTCGGAGAACTTTGTGAAGAGCGAGTGGTGCAAGTATGAGCTGGACTTCTCCCATTTCCGTCTCTTTGATGAGAACAATGATGCTGCCATTCTGATTCTGCTGGAGCCCATTGACAAGAAGGCCATTCCCCAGCGCTTCTGTAAGCTGCGGAAGATCATGAACACCAAGACCTACCTGGAGTGGCCCGTGGATGAGACTCAGCAGGAAGGGTTTTGGTTAAATTTGAGAGCTGCAATAAGGTCCTAG

>PtroTLR2 Pan troglodytes Chimpanzee XM_001155304 2355 ATGCCACATACTTTGTGGATGGTGTGGGTCTTGGGGGTCATCATCAGCCTCTCCAAGGAAGAATCCTCCAATCAGGCTTCTCTGTCTTGTGACCGCAATGGTATCTGCAAGGGCAGCTCAGGATCTTTAAACTCCATTCCCTCAGGGCTCACAGAAGCTGTAAAAAGCCTTGACCTGTCCAACAACAGGATCACCTACATTAGCAACAGTGACCTACAGAGGTGTGTGAACCTCCAGGCTCTGGTGCTGACATCCAATGGAATTAACACAATAGAGGAAGATTCTTTTTCTTCCCTGGGCAGTCTTGAACATTTAGACTTATCCTATAATTACTTATCTAATTTATCATCTTCCTGGTTCAAGCCCCTTTCTTCTTTAACATTCTTAAACTTACTGGGAAATCCTTACAAAACCCTAGGGGAAACATCTCTTTTTTCTCATCTCACAAAATTGCAAATCCTGAGAGTGGGAAATATGGACACCTTCACTAAGATTCAAAGAAAAGATTTTGCTGGACTTACCTTCCTTGAGGAACTTGAGATTGATGCTTCAGATCTACAGAGCTATGAGTCAAAAAGTTTGAAGTCAATTCAGAACGTAAGTCATCTGATCCTTCATATGAAGCAGCATATTTTACTGCTGGAGATTTTTGTAGATGTTACAAGTTCCGTGGAATGTTTGGAACTACGAGATACTGATTTGGACACTTTCCGTTTTTCAGAACTATCCACTGGTGAAACAAATTCATTGATTAAAAAGTTTACATTTAGAAATGTGAAAATCACCGATGAAAGTTTGTTTCAGGTTATGAAACTTTTGAATCAGATTTCTGGATTGTTAGAATTAGAGTTTGATGACTGTACCCTTAATGGAGTTGGTAATTTTAGAGCATCTGATAATGACAGAGTTATAGATCCAGGTAAAGTGGAAACGTTAACAATCCGGAGGCTGCATATTCCAAGGTTTTACTTATTTTATGATCTGAGCACTTTATATTCACTTACAGAAAGAGTTAAAAGAATCACAGTAGAAAACAGTAAAGTTTTTCTGGTTCCTTGTTTACTTTCACAGCATTTAAAATCATTAGAATACTTGGATCTCAGTGAAAATTTGATAGTTGAAGAATACTTGAAAAATTCAGCCTGTGAGGATGCCTGGCCCTCTCTACAAACTTTAATTTTAAGGCAAAATCATTTGGCATCATTGGAAAAAACCGGAGAGACTTTGCTCACTCTGAAAAACTTGACTAACGTTGATATCAGTAAGAATAGTTTTCATTCTATGCCTGAAACTTGTCAGTGGCCAGAAAAGATGAAATATTTGAACTTATCCAGCACACGAATACACAGTGTAACAGGCTGCATTCCCAAGACACTGGAAATTTTAGATGTTAGCAACAACAATCTCAATTTATTTTCTTTGAATTTGCCGCAACTCAAAGAACTTTATATTTCCAGAAATAAGTTGATGACTCTACCAGATGCCTCCCTCTTACCCATGTTACTAGTATTGAAAATCAGTAGGAATGCAATAACTACGTTTTCTAAGGAGCAACTTGACTCATTTCACACACTGAAGACTTTGGAAGCTGGTGGCAATAACTTCATTTGCTCCTGTGAATTCCTCTCCTTCACTCAGGAGCAGCAAGCACTGGCCAAAGTCTTGATTGATTGGCCAGCAAATTACCTGTGTGACTCTCCATCCCATGTGCGCGGCCAGCAGGTTCAGGATGTCCGCCTCTCGGTGTCGGAATGTCACAGGACAGCACTGGTGTCTGGCATGTGCTGTGCTCTGTTCCTGCTGATCCTGCTCACGGGGGTCCTGTGCCACCGTTTTCATGGCCTGTGGTATATGAAAATGATGTGGGCCTGGCTCCAGGCCAAAAGGAAGCCCAGGAAAGCTCCCAGCAGGAACATCTGCTATGATGCATTTGTTTCTTACAGTGAGCGGGATGCCTACTGGGTGGAGAACCTTATGGTCCAGGAGCTGGAGAACTTCAATCCCCCCTTCAAGTTGTGTCTTCATAAGCGGGACTTCATTCCTGGCAAGTGGATCATTGACAATATCATTGACTCCATTGAAAAGAGCCACAAAACTGTCTTTGTGCTTTCTGAAAACTTTGTGAAGAGTGAGTGGTGCAAGTATGAACTGGACTTCTCCCATTTCCGTCTTTTTGATGAGAACAATGATGCTGCCATTCTCATTCTTCTGGAGCCCATTGAGAAAAAAGCCATTCCCCAGCGCTTCTGCAAGCTGCGGAAGATAATGAACACCAAGACCTACCTGGAGTGGCCCATGGACGAGGCTCAGCGGGAAGGATTTTGGGTAAATCTGAGAGCTGCGATAAAGTCCTAG

>CgriTLR2 Cricetulus griseus Chinese hamster AF113614 2355 ATGTTACATGTTCTGTGGACCTTCTGGATCTTGGTGGCCATGACAGATCTCTCCAGGAAGGGATGTTCTGCTCAGGCATCTCTGTCATGTGATGCTGCTGGTGTGTGTGATGGCCGCTCTAGGTCTTTCACCTCCATCCCCTCAGGACTCACAGCAGCCATGAAGAGCCTTGACCTGTCTAACAACAAGATCACCTCCATTGGCCATGGTGACCTTCGGGGGTGTGTGAACCTCCGGGCCCTGATATTGCAGTCCAGTGGGATCAACACCATAGAGGAAGATGCCTTTTCTTCCCTGAGCAAGCTTGAATATTTGGACCTGTCTGATAATCACTTATCCAACCTATCTTCCTCCTGGTTCAGGCCTCTTTCCTCTTTGAAATACCTAAACTTGCTGGGAAATCCTTATAGGATACTGGGGGAAACACCACTGTTTCTCAACCTCACACATTTACAGACCCTTAGGGTAGGAAATGTTGCCACCTTCAGTGGGATCAGGAGAACAGATTTTGCTGGACTGACTTCTCTTGATGAACTTGAAATCAAAGCGCTAAGTCTCCAGAATTATGAGCCCGGAAGTCTGCAGTCCATTCAAAGCATCCATCACTTAACCTTTCACCTAAGTCAGTCTGATTTCCTGCTGGGGGTTTTTGAAGATACTCTGAGTTCTGTGGGATATTTAGAACTGAGAGATGCTAACTTGGACAGCTTCTACTTTTCAGAACTGTCCACGGATGAAATGAATTCACCGATGAAAAAGCTGGCATTCCAAAATGCAGATCTCACTGACGAAAGTTTTAATGAACTTCTGAAGCTGTTGCGTTACACTCCAGAACTGTTGGAGGTAGAATTTGATGACTGCACCCTCAATGGAGTGGGCGATTTTCAGCCCTCAGAGTCAGACGTAGTGAGAGAGCTAGGCAAAGTAGAAACGCTAATAATACGGAGGCTGCACATCCCCAGATTCTACTCATTTTATGATCTGAGTACTGTATATACCCTCCTGGAGAAAGTTAAGAGAATCACAGTCGAGAACAGCAAGGTTTTTCTGGTCCCTTGTCTGTTCTCACAGCATTTAAAATCATTGGAATTCTTGGACCTCAGCGAAAATTTGATGGTTGAAGAATATTTGAAGAACGCAGCCTGTGAGGGCAGCTGGCCTTCCCTGCAAACTTTAATTTTGAGGCAGAACCGTTTGAAATCAATAGAAAGAACTGGAAAGATTTTACTGACTCTGAAAAACCTGACTGCCCTTGATATCAGCCGGAACAGCTTTCAGTCTATGCCTGACTCTTGCCAGTGGCCAGGAAAGATGCGTTTCCTGAACTTGTCCAGTACAGGGATACAGGCAGTAAAAATGTGCATCCCTCAGACTCTGGAGGTGTTGGATGTTAGTAACAACAATCTCATTTCGTTTTCTTTGTTTTTGCCTCTGCTCCGAGAGCTCTATATTTCCAGGAACAAGCTCCACACTCTCCCCGATGCCTCCTTGTTCCCTGTGTTACTGGTCATGAAGATCAGAGAGAATGCCATAAGTACTTTCTCTAAAGACCAACTTAGTTCTTTTCCCAAACTGGTGAGTCTGGAAGCAGGTGGCAACCACTTCATCTGCTCCTGTGAACTCTTGTCCTTCACCTTGGAGCACCCAGCACTGGTCCAGGTCCTGGCCGGCTGGCCAGACAGTTACCTGTGTGACTCTCCCTCCCGCCTGCGTGGCCAGAGGGTCCAGGATGCCCGGCCCTCAGTCTTGGAGTGCCACCAGACTCTACTAGTGTCTGGTGTCTGCTGTGCCCTTGTACTGTTGATCCTGCTCATAGGTGGCCTGTGCCACCATTTCCATGGGCTATGGTACCTGCGGATGATGTGGGCGTGGCTGCAGGCCAAGAGGAAGCCCAAGAAAGCTCCTTGCAGGGACATTTGCTATGATGCCTTTGTTTCCTACAGCGAGCAGGATTCCTACTGGGTGGAGAACCTCATGGTCCAGCAGTTGGAGAATTCGGAACCTCCCTTCAAGCTATGTCTCCACAAGCGGGACTTTGTTCCTGGCAAGTGGATCATTGACAACATCATCGACTCTATTGAGAAGAGCCACAAAACCCTGTTTGTGCTTTCTGAGAACTTTGTGCGGAGCGAGTGGTGCAAGTATGAGCTGGACTTCTCCCACTTCAGGCTCTTTGATGAGAACAACGACGCCGGCATCCTGGTTTTGCTGGAGCCCATTGAGAAGAAAGCCATCCCCCAGCGCTTCTGCAAGCTTCGCAAGATAATGAACACCAAGACCTACCTGGAATGGCCCTTGGATGAAGGCCAGCAGGAAGTGTTTTGGGTAAATCTGAGAACTGCTATAAAGTCCTAG

>CfamTLR2 Canis familiaris Dog AB189639 2358 ATGTCACGTGTTTTGTGGACATTGTGGGTTTTGGGGGCTGTAACCAACCTCTCCAAGGAAGAGGCCCCTGACCAGTCTTCTTCTCTGTCCTGTGACCCCACTGGTGTCTGCGATGGCCGCTCCAGATCTTTGAACTCCATGCCCTCAGGGCTCACAGCAGCTGTGAGAAGCCTTGACCTCTCCAACAATGAGATCACCTACATTGGCAACAGTGATCTTCGGGATTGTGTGAACCTCAAGGCTCTGAGGCTGGAGTCTAATGGAATTAACACAATAGAGGAAGAATCTTTTCTTTCCCTGTGGAGTCTTGAACATTTGGACTTATCTTATAACCTCTTATCTAACTTATCATCCTCCTGGTTCAGGCCCCTTTCTTCATTGAAGTTCTTAAACCTACTGGGAAATCCTTACAAATCACTTGGGGAAACACCTCTTTTTTCTCAGCTCACAAATCTAAGAATTCTGAAAGTAGGAAATATCTACAGCTTCACTGAGATTCAGGATAAGGATTTTGCTGGGCTAACCTTTCTTGAGGAACTGGAGATCGATGCTTCGAATCTCCAGAGGTATGAGCCAAAGAGTTTGAAATCGATTCAGAACATCAGCTATCTGGCCCTCCGTATGAAGCAGCCTGTTTTACTGGTGGAGATTTTTGTAGATCTTTCCAGTTCCTTGAAACATTTAGAACTGAGAGATACTCATTTGGACACTTTCCACTTTTCAGAGGCATCCATCAATGAAACACATACGTTGGTTAAAAAGTGGACATTTAGAAATGTGAAAGTCACCGATAGAAGTTTTACTGGGGTTGTGAGACTGTTGAATTATGTTTCTGGAGTGTTAGAAGTAGAGTTTGAGGACTGTACCCTTTATGGGCTCGGTGATTTTGACATACCTGATGTGGACAAAATTAAAAATATAGGTCAGATAGAGACACTAACAGTACGGAGGTTGCATATTCCACACTTTTACTCATTTTACGATATGAGTAGTATATATTCACTTACAGAAGATGTTAAAAGAATCACAGTAGAGAACAGCAAAGTTTTTCTGGTTCCTTGTTTACTTTCACAACATTTAAAATCATTAGAATACTTGGATCTCAGTGAAAATTTGATGGTTGAAGAATACTTGAAAAATTCAGCCTGTGAGGATGCCTGGCCCTCTCTACAAACTTTAGTTTTAAGGCAAAATCATTTGGCATCATTGGAAAGAACCGGAGAGACTTTGCTCACTCTGAAAAACTTGACTAACATTGATATCAGTAAGAATAGTTTTCATTCTATGCCTGAAACTTGTCAGTGGCCAGAAAAGATGAAATATTTGAACTTATCCAGCACACGAATACACAGCGTAACAGGCTGCATTCCCAAGACACTGGAAATTTTAGATGTTAGCAACAACAATCTCAATTTATTTTCTTTGAATTTGCCGCAACTCAAAGAACTTTATATTTCCAGAAATAAGTTGATGACTCTACCAGATGCCTCCCTCTTACCCATGTTACTAGTATTGAAAATCAGTAGGAATGCAATAACTACGTTTTCTAAGGAGCAACTTGACTCATTTCACACACTGAAGACTTTGGAAGCTGGTGGCAATAACTTCATTTGCTCCTGTGAATTCCTCTCCTTCACTCAGGAGCAGCAAGCACTGGCCAAAGTCTTGATTGATTGGCCAGCAAATTACCTGTGTGACTCTCCATCCCATGTGCGTGGCCAGCAGGTTCAGGATGTCCGCCTCTCGGTGTCGGAATGTCACAGGACAGCACTGGTGTCTGGCATGTGCTGTGCTCTGTTCCTGCTGATCCTGCTCACGGAGGTCCTGTGCCACCGTTTCCATGGCCTGTGGTATATGAGAATGATGTGGGCCTGGCTCCAGGCCAAAAGGAAGCCCAGGAAAGCCCCCTCCAGGGACGTCTGTTATGACGCCTTTGTGTCTTACAGTGAGCATGATTCCTACTGGGTGGAGAACCTTCTGGTCCAGAAGCTGGAGCACTTCAATCCCCCGTTCAAGTTGTGCCTTCACAAGCGGGACTTTATTCCCGGCAAGTGGATTATTGACAATATCATTGACTCCATCGAGAAGAGCCGCAAAACCATCTTTGTGCTTTCTGAAAACTTCGTGAAGAGCGAGTGGTGCAAGTACGAGCTGGACTTCTCCCATTTTCGCCTCTTTGATGAGAACAGCGATGCTGCCATCCTCATTCTTCTGGAGCCCATTGAGAAAAAGGCCATCCCCCAGCGATTCTGTAAGCTGCGGAAGATAATGAACACCAAGACGTACCTGGAGTGGCCCACCGATGATGCTCAGCAGGAAGGGTTTTGGTTAAATTTGAGAACAGCAATAAAATCCTAG

>GcamTLR2 Giraffa camelopardalis Giraffe EU580542 2355 ATGCCACGTGCTTTGTGGACAGCGTGGGTCTGGGCTGTAATCAGCCTGTCCACGGAAGGAGCCTCTGATCAGGCTTCTTCTCTGTCTTGTGACCCAACTGGTGTCTGCGATGGCCATTCCAGATCTTTAAACTCCATCCCCTCTGGTCTCACGGCAGGTGTGAAAAGCCTTGACTTGTCCAACAACGAGATCACCCATGTYGGCAACAGAGACCTTCAGAGCTGTGTGAACCTGAAGACTCTGAGGCTGGGGGCCAATGAAATTCACACAGTGGAGGAAGATTCTTTTTTTCACCTGAGGAGTCTTGAATATTTGGACTTATCCTATAATCGCTTATCTAACTTATCATCCTCCTGGTTCAGGTCCCTTTATGTCTTGAAATTCTTAAACTTACTGGGAAATTTATACAGAACACTTGGGGAAACATCTCTTTTTTCTCATCTCTCAAATCTGCGGACCCTGAAAGTAGGAAATAGTAACAGCTTCACTGAGATTCATGAAAAGGATTTCACTGGACTGACTTTTCTTGAGGAGCTTGAGATCAGTGCTCGAAATCTGCAGATATATGCGCCAAAGAGTTTAAAGTCAATCCAGAACATTAGCCATCTGATTCTTCATCTGAAGCAGCCTGTTTTACTCCTGGACATTCTTGTAGATATTGTAAGTTCCTTAGATTATTTAGAACTGAGAGATACTAATTTGCACACTTTCCATTTTTCAGAAGCATCCATCAGTGAAATGAATACATCGGTTAAAAAGCTTATATTTAGAAATGTGCAATTCACCGATGAAAGTTTTGTTGAAGTTGTCAAACTGTTTAACTATGTTTCTGGGATCTCAGAAGTAGAGTTTGATGACTGTACCCATGATGGAATTGGCGACTTTAGAGCATTGGCTTTGGAGAGAACTAGATACCTAGGTAATGTGGAGACGTTAACAATACGGAAGTTGCATATTCCACAGTTTTTCTTATTTCAAGATCTTAGTAGTATATATTCACTCACAGGCAAAGTTAAAAGAGTCACAATAGAAAACAGTAAGGTTTTTCTGGTTCCTTGTTTACTTTCACAACATTTAAAATCATTAGAATATTTGGATCTCAGTGAAAACTTAATGTCTGAAGAAACTTTGAAAAACTCGGCCTGTGAGCATGCCTGGCCCTTCCTTCAAACCTTGGTTTTAAGGCAGAATCGTTTGAAATCACTAGAAAAAACTGGAGAACTTTTGCTTACTCTGAAAAACCTGACTAACCTTGATATCAGTAAGAATAATTTTCTTTCAATGCCTGAAACTTGTCAGTGGCCAGGAAAAATGAAACAGTTGAACTTATCCAGCACAAGGATACACAGTTTAACCCACTGCCTTCCCCAGACCCTGGAAATTTTAGATGTTAGCAGTAACAATCTCGATTCATTTTCTTTGATTTTGCCGCAACTCAAAGAACTTTATATTTCCAGAAATAAGTTGAAGACTCTACCAGATGCCTCCTTCTTACCCGTGTTATCAGTTATGAGAATTAGCAGAAATATAATAAATACTTTCTCTAAGGAGCAACTTGATTCTTTTCAACAACTGAAGACTTTGGAGGCCGGTGGCAACAACTTCATTTGCTCCTGTGACTTCCTGTCCTTCATGCAGGGACAGCAGGCGCTGGCCCGGGTCCTGGCCGACTGGCCAGATGACTACTGGTGTGACTCTCCCTCCCATGTGCGGGGCCAGCGGGTGCGGGACGCCCGGCTGACCCTTTCCGAATGCCACAGAACAGCCGTGGTGTCTGCCGTGTGCTGTGCCCTCTTCCTGTTGCTGCTGCTCACAGGGGTTCTGTGTCACCGTCTCCACGGACTGTGGTACATGAAGATGATGTGGGCCTGGCTCCAGGCCAAGAGGAAGCCCAGGAAGGCTCCCCGCAGGGACATCTGCTATGACGCCTTTGTGTCCTACAGTGAGCGGGATTCCTACTGGGTGGAGAACCTCATGGTCCGGGAGCTGGAGCACTTCGACCCTCCCTTTAAGCTGTGTCTTCATAAGCGAGACTTCATTCCTGGCAAGTGGATTATCGACAACATCATTGACTCCATCGAAAAGAGCCACAAAACCATCTTCGTGCTTTCGGAGAACTTTGTGAAGAGCGAGTGGTGCAAGTATGAGCTGGACTTCTCCCATTTCCGCCTCTTTGATGAGAACAATGATGCTGCCATTCTGATTCTGCTGGAGCCCATCGACAAGAAGGCCATTCCCCAGCGCTTCTGTAAGCTGCGGAAGGTCATGAACACCAAGACCTACCTGGAGTGGCCCATGGATGAGACTCAGCAGGAAGGGTTTTGGTTAAATTTGAGAGCTGCAGTAAGGTCCTAG

>ChirTLR2 Capra hircus Goat DQ872435 2355 ATGCCACGTGCTTTGTGGACAGCGTGGGTCTGGGCTGTAATCATCCTGTCCATGGAAGGAGCCTCTCATCAGGCTTCTTCTCTGTCTTGTGACCCAACTGGTGTCTGTGATGGCCATTCCAGATCTTTAAACTCCATCCCCTCTGGTCTCACAGACGGTGTGAAAAGCCTTGACCTGTCCAACAACGAGATCACCTATGTCAGCAACAGAGACCTGCAGAGGTGTGTGAACCTGAAGACTCTGAGGCTGGGGGCCAATGAAATTCACACAGTGGAGGAAGATTCTTTTTTTCACCTGAGGAATCTTGAATATTTGGATTTATCCTATAATCGCTTATCTAACTTATCATCCTCCTGGTTCAGGTCCCTTTATGCCTTGAAATTCTTAAACTTACTGGGAAATGTATACAAAACACTTGGGGAAACATCTCTTTTTTCTCATCTCCCAAATCTGCGGACCCTGAAAGTAGGAAATAGTAACAGCTTCACTGAGATTCATGAAAAGGATTTCACTGGACTCATTTTTCTTGAGGAGCTTGAGATCAGTGCTCAAAATCTGCAGATATATGTGCCAAAGAGTTTAAAGTCAATCCAGAACATTAGCCATCTGATTCTACATCTGAAGCAGCCTGTTTTACTCGTGGACATTCTTGTAGATATTGTAAGTTCCTTAGATTGTTTAGAACTGAGAGATACTAATTTGCACACTTTCCATTTTTCAGAAGCATCCATCAGTGAAATGAATACATCTGTTAAAAAGCTTATATTTAGAAATGTGCAATTCACCGATGAAAGTTTTGTCGAAGTTGTCAAACTGTTTAACTATGTTTCTGGGATCTTAGAAGTAGAGTTTGATGACTGTACCCATGATGGAATTGGCGATTTTAGAGCACTGAGTTTGGACAGAATTAGACACCTAGGTAACGTGGAGACGTTAACAATACGGAAGTTGCATATTCCACAGTTTTTCTTATTTCATGATCTCAGTAGTATATATCCACTCACAGGCAAAGTTAAAAGAGTCACAATAGAAAGCAGTAAGGTTTTTCTGGTTCCTTGTTTACTTTCACAACATTTAAAATCATTAGAATATTTGGATCTCAGTGAAAACTTAATGTCTGAAGAAACCTTGAAAAACTCAGCCTGTAAGGATGCCTGGCCCTTCCTTCAAACCTTGGTTTTAAGGCAGAATCGTTTGAAATCACTAGAAAAAACGGGAGAACTTTTGCTTACTCTGAAAAATCTGAATAACCTTGATATCAGTAAGAATAATTTTCTTTCAATGCCTGAAACTTGTCAGTGGCCAGGAAAAATGAAACAGTTGAACTTATCCAGCACGAGGATACACAGTTTAACCCAGTGCCTTCCCCAGACCCTGGAAATTTTAGATGTTAGCAATAACAATCTCGATTCATTTTCTTTGATTTTGCCGCAACTCAAAGAACTTTATATTTCCAGAAATAAGTTGAAGACTCTACCAGATGCCTCCTTCTTACCCGTGTTATCAGTTATGAGAATTAGCGGAAATATAATAAATACTTTCTCGAAGGAACAACTTGATTCTTTTCCACAACTGAAGGCTTTGGAGGCCGGTGGCAACAACTTCATTTGCTCCTGTGACTTCCTGTCCTTCACACAGGGACAGCAGGCACTGGCCCGTGTCCTGGTCGACTGGCCAGATGGCTACCGCTGTGACGCTCCCTCGCACGTGCGGGGCCAGCGGGTGCAGGACGCCCGGCTCTCCCTTTCTGAATGCCACCGGGCGGCCGTGGTGTCCGCCGTGTGCTGTGCCCTCTTCCTGTTGCTCCTGCTCACGGGGGTGCTGTGTCACCGTTTCCACGGGCTGTGGTACATGAAGATGATGTGGGCCTGGCTCCAGGCCAAGAGGAAGCCCAGGAAGGCTCCCCGCAGGGACCTCTGCTACGACGCCTTTGTGTCCTACAGCGAGCAGGATTCCTACTGGGTGGAGAACCTCATGGTCCAGGAGCTGGAGCACTTCAACCCTCCCTTTAAGCTGTGTCTTCATAAGCGAGACTTCGTCCCTGGCAAGTGGATTATCGACAACATCATTGACTCCATTGAAAAGAGCCGCAAAACCATCTTTGTGCTTTCGGAGAACTTTGTGCGGAGCGAGTGGTGCAAGTATGAGCTGGACTTCTCCCACTTCCGTCTCTTTGATGAGAACAATGATGCTGCCATTCTGATTCTGCTGGAGCCCATTGACAAGAAGGCCATCCCCCAGCGCTTCTGTAAGCTGCGGAAGATCATGAACACCAAGACCTACCTGGAGTGGCCCACGGATGAGACTCAGCAGGAAGCGTTCTGGTTGAATTTGAGAGCTGCAATAAGGTCCTAG

>BindTLR2 Bos indicus Gudali Zebu EU413951 2355 ATGCCACGTGCTTTGTGGACAGCATGGGTCTGGGCTGTCATCATCCTGTCCACGGAAGGAGCCTCTGACCAGGCTTCTTCTCTGTCTTGTGACCCAACTGGTGTCTGCGATGGCCATTCCAGATCTTTAAACTCCATCCCCTCTGGTCTCACGGCAGGTGTGAAAAGCCTTGACCTGTCCAACAACGAGATCACCTATGTCAGCAACAGAGACCTGCAGAGGTGTGTGAACCTGAAGACTCTGAGGCTGGGGGCCAATGAAATTCACACAGTGGAGGAAGATTCTTTTTTTCACCTGAGGAATCTTGAATATTTGGACTTATCCTATAATCGCTTATCTAACTTATCATCCTCCTGGTTCAGGTCCCTTTATGTCTTGAAATTCTTAAACTTACTGGGAAATTTATACAAAACACTTGGGGAAACATCTCTTTTTTCTCATCTCCAAAATCTGCGGACCCTAAAAGTAGGAAATAGTAACAGCTTCACTGAGATTCATGAAAAGGATTTCACTGGACTGACTTTTCTTGAGGAGCTTGAGATCAGTGCTCAAAATCTGCAGATATATGTGCCAAAGAGTTTAAAGTCAATCCAGAACATTAGCCATCTGATTCTACATCTGAAGCAGCCTGTTTTACTCGTGGACATTCTTGTAGATATTGTAAGTTCCTTAGATTGTTTAGAACTGAGAGATACTAATTTGCACACTTTCCATTTTTCAGAAGCATCCATCAGTGAAATGAGTACATCGGTTAAAAAGCTTATATTTAGAAATGTGCAATTCACCGATGAAAGTTTTGTTGAAGTTGTCAAACTGTTTAACTATGTTTCTGGGATCTTAGAAGTAGAGTTTGATGACTGTACCCATGATGGAATTGGCGATTTTAGAGCACTGAGTTTGGACAGAATTAGACACCTAGGTAATGTGGAGACGTTAACAATACGGAAGTTGCATATTCCACAGTTTTTCTTATTTCAAGATCTGAGTAGTATATATCCACTCATAGGCAAAGTTAAAAGAGTCACAATAGAAAGCAGTAAGGTTTTTCTGGTTCCTTGTTTACTTTCACAACATTTAAAATCATTAGAATATTTGGATCTCAGTGAAAACTTAATGTCTGAAGAAACCTTGAAAAACTCAGCCTGTAAGGATGCCTGGCCCTTCCTTCAAACCTTGGTTTTAAGGCAGAATCGTTTGAAATCACTAGAAAAAACGGGAGAACTTTTGCTTACTCTGGAAAATCTGAATAGCCTTGATATCAGTAAGAATAATTTTCTTTCAATGCCTGAAACTTGTCAGTGGCCAGGCAAAATGAAACAGCTGAACTTATCCAGCACAAGGATACACAGTTTAACCCAGTGCCTTCCCCAGACCCTGGAAATTTTAGATGTTAGCAATAACAATCTCGATTCATTTTCTTTGATTTTGCCACAACTCAAAGAACTTTATATTTCCAGAAATAAGTTGAAGACTCTACCAGATGCCTCCTTCTTACCCGTGTTATCAGTTATGAGAATTAGCAGAAATATAATAAATACTTTCTCAAAGGAACAACTTGATTCTTTTCAGCAACTGAAGACGTTGGAGGCCGGTGGCAACAACTTCATTTGCTCCTGTGACTTCCTGTCCTTCACACAGGGACAGCAGGCACTGGGCCGTGTCCTGGTCGACTGGCCCGATGACTACCACTGTGACTCTCCCTCCCACGTGCGGGGCCAGCGGGTGCAGGACGCCCGGCTCTCCCTTTCTGAATGCCACAGGGCGGCCGTGGTGTCCGCCGCTTGCTGTGCCCTCTTCCTGTTGCTCCTGCTCATGGGGGTGCTGTGTCACCGTTTCCACGGACTGTGGTACATGAAGATGATGTGGGCGTGGCTGCAGGCCAAGAGGAAGCCCAGGAAGGCTCCCCGCAGGGACATCTGCTACGACGCCTTCGTGTCCTACAGCGAGCGGGATTCCTACTGGGTGGAGAACCTCATGGTCCAGGAGCTGGAGCAGTTCAACCCTCCCTTTAAGCTGTGTCTTCACAAGCGAGACTTCATTCCTGGCAAGTGGATCATCGACAACATCATTGACTCCATTGAAAAGAGCCACAAAACCATCTTTGTGCTTTCGGAGAACTTTGTGAAGAGCGAGTGGTGCAAGTATGAGCTGGACTTCTCCCATTTCCGTCTCTTTGATGAGAACAATGATGCTGCCATTCTGATTCTGCTGGAGCCCATTGACAAGAAGGCCATTCCCCAGCGCTTCTGTAAGCTGCGGAAGATCATGAACACCAAGACCTACCTGGAGTGGCCCGTGGATGAGACTCAGCAGGAAGGGTTTTGGTTAAATTTGAGAGCTGCAATAAGGTCCTAG

>EcabTLR2 Equus caballus Horse AY429602 2355 ATGCCACATGCTTTGTGGACGGTGTGGGTCTTAGGGGCCGTAATCAGCCTCTCCAAGGAAGGGGTCCCTGATCAGCCTTCTTCTCTGTCTTGTGATCCCACTGGTGTCTGCGATGGCCGCTCCAGATCTTTAAACTCCATCCCCTCAGGCCTCACGGCAGCTGTGAAAAGTCTTGACCTGTCCAACAACAAGATCGCCTCTGTCGGCAACAGTGACCTGTGGAAGTGTGTGAACCTCAAGGCCCTGAGGCTGGGGTCCAATGACATCAACACCATAGAGGAAGACTCTTTTTCCTCCCTGAGGAGTCTTGAACATTTGGACTTGTCCAATAATCACTTATCTAATTTATCGTCCTCCTGGTTCAGGCCCCTTTCTTCTTTAAAATTCTTAAACTTACTGGGAAGCACTTACAAAACACTCGGGGAAACATCTCTTTTTTCTCATCTCACCAATTTGCGAATCCTGAAAGTGGGAAATATTCACTTCACTGAGATTCAGGGAAAGGATTTTGCTGGGCTCACTTTCCTTGAAGAACTTGAGATTGATGCTACAAATCTCCAGCGGTATGAGCCCAAGAGTTTCAAATCAATTCAGAACATCAGTCATTTGATCCTTCGTATGAAGCAGCCTGTTTTACTGCCGGAGATTATTCTAGATACTTTGAGTTCCTTGGAATATTTGGAACTGAGAGATACTTATTTAAACACTTTCCATTTTGCAGAAGTATCTGACCCTGAAACCAATACATTGATTAAAAAGTTCACATTTAGAAATGTGAAAATCACTGATGAAAGTTTTGATGAAATTGTGAAGCTGTTGAATTATATTTCTGGAGTGTCAGAAGCAGAGTTTGATGAGTGTACTCTTGATGGGCTTGGTGAATTTAGGACACCGGATATAGACAAAATTAAAGTTATAGGTAAGCTAGAGACATTAACGATACGGAGGTTGCGTATTCCACAGTTTTACTTATTTCGTGATCTGAGTAGTATTTATTCACTTACAGAAAGAGTTAAAAGGATCACAATAGAAAATAGTAAGGTTTTTCTGGTTCCTTGTTCACTTTCACGACATTTAAAATCATTAGAATATTTGGATCTCAGTGACAATTTAATGGTTGAGGAATACTTGAAAAACTCAGCCTGTGAGCGTGCCTGGCCCTCCCTCCAAACCTTAATTTTAAGGCAAAATCATTTGACGTCATTAGGAAAAACTGGAGAAACTTTGCTTACTCTGAAAAACCTGACTAAACTTGACATCAGCAAGAACAGTTTTCATTCTATGCCTGAAACTTGTCAGTGGCCAGAAAAGATGAAATATTTGAATTTATCCAGCATAAGAATAGATCGTTTAACCCAATGCATTCCCCAGACACTGGAAGTTTTAGATATTAGCAATAACAATCTCAATTCATTTTCTTTGATTTTGCCACAAGTCAAAGAACTTTATATTTCCAGAAATAAGTTGAAGACTCTACCAGATGCCTCCTTCTTACCCATGTTATTAGTCATGAGAATCAGCAGAAAAACAATAAATACTTTCTCTAAGGAGCAACTTGATTCTTTTCAAAAATTGAAGACTTTGGAAGCTGGTGGCAACAATTTCATTTGCTCCTGTGAGTTCCTGTCTTTCACTCAGGAGGAGCAGGCACTGGACCAGATCCTGATCGACTGGCCAGAAAACTACCTGTGTGACTCTCCCTCCCACGTGCGGGGCCAGCGGGTTCAGGACACTCATCTCTCGGTCTCTGAATGCCACAGGACAGCTCTGGTGTCTGCTGTGTGCTGTGCCCTTTTCCTGTCGATCCTGCTCACTGGGGTTCTGTGTCACCATTTCCATGGACTGTGGTACATGAAAATGATGTGGGCCTGGCTCCAGGCCAAAAGGAAGCCCAGGACAGCTCCCCAAAGGGACATCTGTTATGACGCCTTCGTGTCTTACAGTGAACGGGACTCCTACTGGGTGGAGAACCTCATGGTGCAGGAGCTGGAGCACTTCAACCCTCCCTTTAAGTTGTGTCTTCATAAGCGGGACTTTATTCCTGGCAAATGGATTATTGACAATATCATTGACTCGATTGAAAAGAGCCACAAAACCATCTTTGTGCTTTCTGAAAACTTTGTGAAGAGTGAGTGGTGTAAGTACGAACTGGACTTCTCCCATTTTCGTCTCTTTGATGAGAACAATGATGCTGCCATTCTCATTCTTCTGGAGCCCATTGACAAAAAGGCCATTCCCCAGCGTTTCTGTAAGCTGCGGAAGATAATGAACACCAAGACCTACCTGGAGTGGCCCACTGATGAAGCTCAGCAGGAGGGGTTTTGGTTAAATTTGAGAGCCGCCATAAAGTCCTAG

>HsapTLR2 Homo sapiens Human BC033756 2355 ATGCCACATACTTTGTGGATGGTGTGGGTCTTGGGGGTCATCATCAGCCTCTCCAAGGAAGAATCCTCCAATCAGGCTTCTCTGTCTTGTGACCGCAATGGTATCTGCAAGGGCAGCTCAGGATCTTTAAACTCCATTCCCTCAGGGCTCACAGAAGCTGTAAAAAGCCTTGACCTGTCCAACAACAGGATCACCTACATTAGCAACAGTGACCTACAGAGGTGTGTGAACCTCCAGGCTCTGGTGCTGACATCCAATGGAATTAACACAATAGAGGAAGATTCTTTTTCTTCCCTGGGCAGTCTTGAACATTTAGACTTATCCTATAATTACTTATCTAATTTATCGTCTTCCTGGTTCAAGCCCCTTTCTTCTTTAACATTCTTAAACTTACTGGGAAATCCTTACAAAACCCTAGGGGAAACATCTCTTTTTTCTCATCTCACAAAATTGCAAATCCTGAGAGTGGGAAATATGGACACCTTCACTAAGATTCAAAGAAAAGATTTTGCTGGACTTACCTTCCTTGAGGAACTTGAGATTGATGCTTCAGATCTACAGAGCTATGAGCCAAAAAGTTTGAAGTCAATTCAGAACGTAAGTCATCTGATCCTTCATATGAAGCAGCATATTTTACTGCTGGAGATTTTTGTAGATGTTACAAGTTCCGTGGAATGTTTGGAACTGCGAGATACTGATTTGGACACTTTCCATTTTTCAGAACTATCCACTGGTGAAACAAATTCATTGATTAAAAAGTTTACATTTAGAAATGTGAAAATCACCGATGAAAGTTTGTTTCAGGTTATGAAACTTTTGAATCAGATTTCTGGATTGTTAGAATTAGAGTTTGATGACTGTACCCTTAATGGAGTTGGTAATTTTAGAGCATCTGATAATGACAGAGTTATAGATCCAGGTAAAGTGGAAACGTTAACAATCCGGAGGCTGCATATTCCAAGGTTTTACTTATTTTATGATCTGAGCACTTTATATTCACTTACAGAAAGAGTTAAAAGAATCACAGTAGAAAACAGTAAAGTTTTTCTGGTTCCTTGTTTACTTTCACAACATTTAAAATCATTAGAATACTTGGATCTCAGTGAAAATTTGATGGTTGAAGAATACTTGAAAAATTCAGCCTGTGAGGATGCCTGGCCCTCTCTACAAACTTTAATTTTAAGGCAAAATCATTTGGCATCATTGGAAAAAACCGGAGAGACTTTGCTCACTCTGAAAAACTTGACTAACATTGATATCAGTAAGAATAGTTTTCATTCTATGCCTGAAACTTGTCAGTGGCCAGAAAAGATGAAATATTTGAACTTATCCAGCACACGAATACACAGTGTAACAGGCTGCATTCCCAAGACACTGGAAATTTTAGATGTTAGCAACAACAATCTCAATTTATTTTCTTTGAATTTGCCGCAACTCAAAGAACTTTATATTTCCAGAAATAAGTTGATGACTCTACCAGATGCCTCCCTCTTACCCATGTTACTAGTATTGAAAATCAGTAGGAATGCAATAACTACGTTTTCTAAGGAGCAACTTGACTCATTTCACACACTGAAGACTTTGGAAGCTGGTGGCAATAACTTCATTTGCTCCTGTGAATTCCTCTCCTTCACTCAGGAGCAGCAAGCACTGGCCAAAGTCTTGATTGATTGGCCAGCAAATTACCTGTGTGACTCTCCATCCCATGTGCGTGGCCAGCAGGTTCAGGATGTCCGCCTCTCGGTGTCGGAATGTCACAGGACAGCACTGGTGTCTGGCATGTGCTGTGCTCTGTTCCTGCTGATCCTGCTCACGGGGGTCCTGTGCCACCGTTTCCATGGCCTGTGGTATATGAAAATGATGTGGGCCTGGCTCCAGGCCAAAAGGAAGCCCAGGAAAGCTCCCAGCAGGAACATCTGCTATGATGCATTTGTTTCTTACAGTGAGCGGGATGCCTACTGGGTGGAGAACCTTATGGTCCAGGAGCTGGAGAACTTCAATCCCCCCTTCAAGTTGTGTCTTCATAAGCGGGACTTCATTCCTGGCAAGTGGATCATTGACAATATCATTGACTCCATTGAAAAGAGCCACAAAACTGTCTTTGTGCTTTCTGAAAACTTTGTGAAGAGTGAGTGGTGCAAGTATGAACTGGACTTCTCCCATTTCCGTCTTTTTGATGAGAACAATGATGCTGCCATTCTCATTCTTCTGGAGCCCATTGAGAAAAAAGCCATTCCCCAGCGCTTCTGCAAGCTGCGGAAGATAATGAACACCAAGACCTACCTGGAGTGGCCCATGGACGAGGCTCAGCGGGAAGGATTTTGGGTAAATCTGAGAGCTGCGATAAAGTCCTAG

>CibeTLR2 Capra ibex Ibex EU580540 2355 ATGCCACGTGCTTTGTGGACAGCGTGGGTCTGGGCTGTAATCAGCGCGTTCACGGAAGGAGCCTCTGATCAGGCTTCTTCTCTGTCTTGTGACCCAACTGGTGTCTGCGATGGCCATTCCAGATCTTTAAACTCCATCCCCTCTGGTCTCACGGCAGGTGTGAAAAGCCTTGACCTGTCCGACAACGAGATCACCTATGTCGGCAACAGAGACCTGCAGAGGTGTGTGAACCTGAAGACTCTGAGGCTGGGGGCCAATGAAATTCACACGGTGGAGGAAGATTCTTTTTTTCACCTGAGGAATCTTGAATATTTGGACTTATCCTATAATCGCTTATCTAACTTATCATCTTCCTGGTTCAGGTCCCTTTACGTCTTGAAATTCTTAAACTTACTGGGAAATTTATACAAAACACTTGGGGAAACATCTCTTTTTTCTCATCTCCCAAATCTGCGGACCCTGAAAGTAGGAAATAGTAACAGCTTCACTCAGATTCATGAAAAGGATTTCACTGGACTGACTTTTCTTGAGGAGCTTGAGATCAGTGCTCAAAATCTGCAGTTATATGTGCCAAAGAGTTTAAAGTCGATCCAGAACATTAGCCATCTGATTCTTCATCTGAAGCAGCCTGTTTTACTCCTGGACATTCTTATAGATATTGTAAGTTCCTTAGATTATTTAGAACTGAGAGATACTAATTTGCACACTTTCTATTTTTCAGAAGCATCCATCAGTGAAATTAATACATCAGTTAAAAAGCTTATATTTAGAAATGTGCAATTCACCGATGAAAGTTTTGTTGAAGTTGTCAAACTGTTTAACTATGTTTCTGGGATCTTAGAAGTAGAGTTTGATGACTGTACCCATGATGGAATTGGCGATTTTACAGCACTGACTTTGAACAGAATTAGATACCTAGGTAACGTGGAGACGTTAACAATACGGAAGTTGCATATCCCACAGTTTTTCTTATTTTATGATCTGAGTAGTATATATCCACTCACAGGCAAAGTTAAAAGAGTCACAATAGAAAACAGTAAGGTTTTCCTGGTTCCTTGTTTACTTTCACAACATTTAAAATCGCTAGAATATTTGGATCTCAGTGAAAACTTAATGTCTGAAGAAACCTTGAGAAACTCAGCCTGTGAGCATGCCTGGCCCTTCCTTCAAACCCTGGTTTTAAGGCAGAATCGTTTGAAATCACTAGAAAAAACTGGAGAACTTTTGCTTACTCTGAAAAATCTGAATAACCTTGATATCAGTAAGAATAATTTTCTTTCAATGCCTGAAACTTGTCAGTGGCCAGGAAAAATGAAACAGTTGAACTTATCCAGCACGAGGATACACAGTTTAACCCAGTGCCTTCCCCAGACCCTGGAAATTTTAGATGTTAGCAATAACAATCTCGATTCATTTTCTTTGATTTTGCCGCAACTCAAAGAACTTTACATTTCCAGAAATAAGTTGAAGACTCTACCAGATGCCTCCTTCTTACCCGTGTTATCAGTTATGAGAATTAGCGGAAATATAATAAATACTTTCTCGAAGGAACAACTTGATTCTTTTCCACAACTGAAGGCTTTGGAGGCCGGTGGCAACAACTTCATTTGCTCCTGTGACTTCCTGTCCTTCACACAGGGACAGCAGGCACTGGCCCGTGTCCTGGTCGACTGGCCAGATGGCTACCGCTGTGACGCTCCCTCGCACGTGCGGGGCCAGCGGGTGCAGGACGCCCGGCTCTCCCTTTCTGAATGCCACCGGGCGGCCGTGGTGTCCGCCGTGTGCTGTGCCCTCTTCCTGTTGCTCCTGCTCACGGGGGTGCTGTGTCACCGTTTCCACGGGCTGTGGTACATGAAGATGATGTGGGCCTGGCTCCAGGCCAAGAGGAAGCCCAGGAAGGCTCCCCGCAGGGACCTCTGCTACGACGCCTTTGTGTCCTACAGCGAGCAGGATTCCTACTGGGTGGAGAACCTCATGGTCCAGGAGCTGGAGCACTTCAACCCTCCCTTTAAGCTGTGTCTTCATAAGCGAGACTTCGTCCCTGGCAAGTGGATTATCGACAACATCATTGACTCCATTGAAAAGAGCCGCAAAACCATCTTTGTGCTTTCGGAGAACTTTGTGCGGAGCGAGTGGTGCAAGTATGAGCTGGACTTCTCCCACTTCCGTCTCTTTGATGAGAACAATGATGCTGCCATTCTGATTCTGCTGGAGCCCATTGACAAGAAGGCCATCCCCCAGCGCTTCTGTAAGCTGCGGAAGATCATGAACACCAAGACCTACCTGGAGTGGCCCACGGATGAGACTCAGCAGGAAGCGTTCTGGTTGAATTTGAGAGCTGCAATAAGGTCCTAG

>MmurTLR2 Microcebus murinus Mouse Lemur ENSMICG00000003197 2355 ATGCCATATGCTTTGTGGACGGTATGGGTGTTGGGGGCTGTCATTAGCCTCTCCAAGGAAGGGCCCTTTGGTCAGGCTTCTCTGACTTGTGACTCCACTGGTGTCTGTGTTGGCCTCTCGAGCTCTTTCAACTCCATTCCCCCAGGGCTTACAGCGGCTGTGAAAAGTCTTGACCTGTCCAACAACAAGATCACCTATATTGGCAGCAGTGACCTGCAGAGGTGTGTGAACCTCCAGGCTCTGGTGCTAAAGTCCAATGAAATTAACACAATAGATGAAGATTCTTTTTATTCCCTGGGCAGTCTGAAACATTTGGACTTATCCTATAATGACTTATCTAATTTATCATCCTCCTGGTTCAGGCCCCTTTCTTCCTTGGAATCCTTAAACTTACTGGGAAATCCTTACAGAACACTCGGGGAAACGTCTCTCTTTTCTCATCTCACAAATTTGCGAACCCTGAGAGTGGGAAATACTAACAGCTTCACTAAGATTCAGAGAAAGGATTTTGCTGGGCTCACTTTTCTTCAGGAACTTGAGATTGATGCTTCAGCTCTACAGAGTTATGAGCCAAAAAGTTTGAGGTCAATTCAGAATATAAGTTATCTGATTCTTCATATGAGAGAGCCTATTTTACAACTGGAGATTTTTGTAGATCTTTTAAGTTCCGTGGAATACGTGGAACTGAGAAATGCTGATTTGTACAATTTCCATTTTTCAGAACTCTCCATCAGTGAAACAAATCCATTGATTAAAAAGTTTACATTGAGAAAAGTGAAAATCACCGATGAAAGTTTGGCTCAAGTTCTAAAATTCCTGAGTTATGCTTCTGAGTTGTTAGAGATAGAGTTTGATGACTGTACTCTTGATGGAGTAGGTGATTTTAGTACATTTGATGAAGACATAGTTAAAGATCCAGGTAAAGTAGAAACATTAACTATACGGAGGTTGCGTATTCTACATTTTTATTCATTTTATGATCTGAGGACAATATATTCACTTGCAGGAAGAGTTAAAAGAATCACAGTAGAAAACAGTAAGGTTTTTCTGGTTCCTTGTTTACTTTCACAACATTTAAAATCATTAGAATACTTGGATCTCAATGAAAATTTGATGTTAGAGGAACAGTTGAAAAATTCAGCCTGTGAGAATGCCTGGCCCTCTCTACATACCTTAATTTTAAGGCAAAATCATTTAACGTCATTAGGAAAAACTGCAGAGATTTTGCTTACTCTGAAAAACCTGACTCACCTTGATATCAGTAAGAATCACTTTCATACTATGCCTAAAACTTGTCAGTGGCCAGAAAAGATGAAATATTTGAACTTATCCAACACACGAATACACAATTTAACCTACTGCATTCCCCAGACACTGGAAATTTTAGATGTGAGCGATAACAACCTCAATTCATTTTCTGTGACTTTGCCACAACTCAAAGAACTTTATATTTCCAAAAATAAGTTGAAGACTCTACCAGATGCCTCCTTCTTACCTGCATTACTGGTGTTGAACATCAGAACGAACACAGTAAATTCTTTTTCTAAGGAGCAACTTGATTCTTTTCACAAACTGAAAACTTTGGAAGCTGGTGGCAACACCTTCATTTGCTCCTGTGAGTTCCTCTCTTTCACTCGGGAGCAGCAAGCACTGGCCACAGTCTTCATCAATTGGACCGAAAACTACCTGTGTGATTCTCCGTCCTACGTGCGGGGCCAGCGGGTTCAGGATGCCCGTCTCTCCGTGTCTGAGTGTCACAGGGCGGCACTGGCGTCCGGCATGTGCTGTGCCCTCTTCCTGTTGATCCTGGTCACCGGGGTGCTGTGCCACCGTTTCCATGGAGTGTGGTACATGAAAATGATGTGGGCCTGGCTCCAGGCCAAAAGGAAGCCCCGGAAGGCTCCCAGGAGGGACGTCTGTTACGATGCATTTGTTTCTTACAGTGAACACGATTCCTACTGGGTGGAGAACCTGATGGTCCAGGAGCTGGAGAATTTCAACCCCCCCTTCAAGTTGTGTCTTCATAAGCGAGACTTCATTCCTGGCAAATGGATCATTGACAATATCATTGACTCCATCGAAAAGAGCCACAAAACTGTCTTTGTGCTCTCTGAAAACTTTGTGAAGAGCGAGTGGTGCAAGTATGAACTGGATTTCTCCCATTTTCGGCTCTTCGATGAGAACGATGATGCTGCCATTCTCATCCTTCTGGAGCCCATCGAGAAGAAAGCCATTCCTCAGCGCTTCTGTAAGCTGCGGAAGATCATGAACACTAAGACCTACCTGGAGTGGCCCATTGACGAGGCTCTGCAGGAGGGATTCTGGTTAAATCTGAGAGCTGCAATAAAGTCCTAG

>CjacTLR2 Callithrix jacchus Marmoset ENSCJAG00000021811 2355 ATGCCACATACTTTGTGGATGGTATGGGCCTTGGGGGCCATCATCAGCCTCTCCAAGGAAGAATCCTCCGATCAGGCATCTCTGTCTTGTGACCACAATGGTATCTGCAAGGGCAGCTCAGGATCTTTAAACTCAATTCCCTCAGGGCTCACAGCAGCTGTAAAAAGCCTTGACCTGTCTAACAACAGGATCACCTACATTAGCAACAGTGACCTACAGAAGTGTGTGAACCTTCAGACTCTGGTGCTAACATCCAATGGAATTAACAGAATAGAGGAAGATTCATTTTCTTCCCTTGGCAGTCTTGAACATTTAGACTTATCCTATAATTACTTATCTAATTTATCATCCTCCTGGTTCAAGCCCCTTTCTTCTTTGACATCCTTAAACTTACTGGAAAATCCTTACAAAATCCTTGGGGAAACATCTCTTTTTTCTCATCTCACAAAATTGCGAATTCTGAGAGTAGGAAATACGGACACCTTCACTAAGATTCAAAGAAAAGATTTTGCTGGACTTACCTTCCTTGAGGAACTTGAGATTGATGCTTCAAATCTACAGAGTTATGACCCAAAAAGTTTGAAGTCAATTCAGAATATAAGTCATCTGGTCCTTCATATGAAGCAGAATATTTTACTGCTGGAGATTTTTGTAGATATTACAAGTTCCGTGGAATATTTGGAACTGCAAGATACTGATTTGGACACTTACCATTTTTCAGAACCATCCACTGGTGAAACAAATTCATTGATTAAAAAGTTTACATTTAGAAATGTGAAAATCACCGATGAAAGTTTGTTTCAGGTCCTGAAACTTTTGAATCAGATTTCTGGGCTGTTAGAATTAGAGTTTGATGACTGTACCCTTAATGGAGTTGGTGATTTTAGAGCACCTGATAATGACAGAGCTATAGATCCAGGTAAAGTGGAGACGTTCACAGTCCGGAGGTTGCATATTCCACAGTTTTACCTATTTTATGATCTGAGTACTTTATATTCACTCACAGAAAGAATTAAAAGAATCACAGTAGAAAACAGTAAAGTTTTTCTGGTTCCTTGTTCACTTTCACAACATTTAAAATCATTAGAATACTTGGATCTCAGTGAAAATTTGATGGTTGAAGAACAGTTGAAAAATTCAGCCTGTGAGGATGCCTGGCCCTCTCTACAAACTTTAATTTTAAGGCAAAATCACTTGGCATCATTGGAAGAGACTGGAAAGACTTTGCTTACTCTGAAAAACTTGACCAACCTTGATGTCAGTAAGAATAGTTTTCGTTCTATGCCTGAAACTTGTCAGTGGCCAGAAAAGATGAAATATTTGAACTTATCCAGCACACGAATACATAGCGTAACAGGCTGCATTCCCAAGACGCTGGAAATTTTAGATGTTAGCAACAACAATCTCAATTTATTTTCTTTGCATTTGCCGCAACTCAAAGAACTTTATATTTCCAGAAATAAGTTGGCGACTCTACCAGATGCCTCCCTCTTACCTGTGTTACTAGTGTTGAAAATCAGTAGGAATACAATAAATATGTTTTCCAAGGAGCAAATTGACTCTTTTCTCACACTGAAGACTTTGGAAGCTGGTGGCAATAATTTCATTTGCTCCTGCGAATTCCTCTCCTTCACTCAGAAGCAGCAAGCATTGGGCAAAGTTCTGATCGATTGGCCAGAAAACTACCTGTGTGACTCTCCATCCCACGTGCGTGGCCAACGGGTTCAGGATGTCCACCTCTCGGTGTCAGAATGCCACAGGGTGGCACTAGTGTCTGTTATGTGCTGCGCCCTGTTCCTGCTGGTCCTGCTCACGGTGGTTCTGTGCCACCGTTTCCATGGCCTGTGGTACATGAAAATGATGTGGGCCTGGCTCCGTGCCAAAAGAAAGCCCAGGAAAGCTCCCAGCAGGGACATCTGCTATGATGCATTTGTTTCTTACAGTGAGCAGGATGCCTACTGGGTGGAGAACCTTATGGTCCAGGAGCTGGAGAACTTCAATCCCCCCTTCAAGTTGTGTCTTCATAAGCGGGACTTCATTCCTGGCAAGTGGATCATTGACAATATCATCGACTCCATTGAAAAGAGCCACAAAACTGTCTTTGTACTTTCTGAAAACTTTGTGAAGAGTGAGTGGTGCAAGTATGAACTGGACTTCTCCCATTTCCGTCTTTTTGATGAGAACAATGATGCTGCCATTCTCATTCTTCTGGAGCCCATTGAGAAAAAAGCCATTCCCCAGCGCTTCTGCAAGCTACGGAAGATAATGAACACCAAGACTTACCTCGAGTGGCCCACAGACGAGGCCCAGCAGGAAGTGTTTTGGGTAAATCTGAGAGCTGCAATAAAGTCCTAG

>MfasTLR2 Macaca fascicularis Crab-Eating Macaque AYQ45573 2355 ATGCCACATACTTTGTGGATGGTGTGGGTCTTGGGAGTCATCATCAGCCTCTCCAAGGAAGAATCCTCCAATCAGGCTTCTCTGTCTTGTGACCACAATGGTATTTGCAAGGGCAGCTCAGGATCTTTAAACTCCATTCCCTCAGGGCTCACAGAAGCTGTAAAAAGCCTTGACCTGTCCAACAACAGGATCACCTACATTAGCAACAGTGACCTACAGAGGTATGTGAACCTCCAAGCTCTGGTGCTGACATCCAATGGAATTAACACAATAGAGGAAGATTCTTTTTCTTCCCTGGGCAGACTTGAACATTTAGACTTATCCTATAATTACTTATCTAATTTATCGTCCTCCTGGTTCAAGCCCCTTTCTTCTTTAAAATTCTTAAACTTACTGGGAAATCCTTACAAAACCCTCGGGGAAACATCTCTTTTTTCTCATCTCACAAAATTGCGAATCCTGAGAGTAGGAAATATGGACACCTTCACTAAGATTCAAAGAAAAGATTTTGCTGGACTTACCTTCCTTGAGGAACTTGAGATTGATGCTTCAGATCTACAGAGCTATGAGCCAAAAAGTTTGAAGTCAATTCAGAATGTAAGTCATCTGATCCTTCATATGAAGCAGCATATTTTACTGCTGGAGATTTTTGTAGATCTTACAAGTTCCGTGGAATGTTTGGAACTGCGAGATACTGATTTGGACACTTTCCATTTTTCAGAACTATCCACTGGTGAAACAAATTCATTGATTAAAAAGTTTACATTTAGAAATGTGAAAATCACCGATGAAAGTTTGTTTCAAGTCATGAAACTTTTGAGTCAGATTTCTGGATTGTTAGAATTAGAGTTTGATGACTGTACCCTTAATGGAGTTGGTGATTTTAGAGGATCTGATAATGACAGAGTTATAGATCCAGGTAAAGTGGAAACAGTAACAATCCGGAGGCTGCATATTCCACAGTTTTACTCATTTAATGATCTGAGCACTTTATATCCACTCACAGAAAGAGTTAAAAGAATCACAGTAGAAAACAGTAAAGTTTTTCTGGTTCCTTGTTTACTTTCACGACATTTAAAATCATTAGAATACTTGGATCTAAGTGAAAATTTGATGGTGGAAGAATACTTGAAAAATTCAGCCTGTGAGGATGCCTGGCCCTCTCTACAAACTTTAATTCTAAGGCAAAATCACTTGGCATCATTGGGAAAAACCGGAGAGACTTTGCTTACTCTGAAAAACTTGACTAACCTTGATATCAGTAAGAATACTTTTCATTATATGCCTGAAACTTGTCAATGGCCAGAAAAGATGAAATATTTGAACTTATCCAGCACACGAATACACAGTGTAACAGGCTGCATTCCCAAGACACTGGAAATTTTAGATATTAGCAACAACAATCTCAATTTATTTTCTTTGAATTTGCCGCAACTCAAAGAACTTTATATTTCCAGAAATAAGTTGATGACTCTACCAGATGCCTCCCTCTTACCCATGTTACTAGTGTTGAAAATCAGTAGGAATACAATAACGACATTTTCTAAGGAGCAACTTGACTCTTTTCACACATTGAAGACTTTGGAAGCTGGTGGCAATAACTTCATTTGCTCCTGTGAATTCCTGTCCTTCACTCAGGAGCAGCAAGCACTGGCCAAAGTCCTGGTTGATTGGCCAGCAAATTACCTGTGTGACTCTCCATCCCATGTGCGTGGCCAGCGGGTTCAGGATGTCCGCCTCTCAGTGTCGGAATGTCACAGGGCAGCACTGGTGTCTGGCATGTGCTGTGCTCTGTTCCTGCTGATCCTGCTCATGGGGGTCCTGTGCCACCGTTTCCACGGCCTGTGGTACATGAAAATGATGTGGGCCTGGCTCCAGGCCAAAAGGAAGCCCAGGAAAGCTCCCAACAGGGACATCTGCTATGATGCGTTTGTTTCTTACAGTGAGCGGGATGCCTACTGGGTGGAGAACCTTATGGTCCAGGAGCTGGAGAACTTCAATCCCCCCTTCAAGTTGTGTCTTCATAAGCGGGACTTCATTCCTGGCAAGTGGATCATTGACAATATCATTGACTCCATTGAAAAGAGCCACAAAACTGTCTTTGTGCTTTCTGAAAACTTTGTGAAGAGTGAGTGGTGCAAGTATGAACTGGACTTCTCCCATTTCCGTCTTTTTGATGAGAACAATGATGCTGCCATTCTTGTTCTTCTGGAGCCCATTGAGAAAAAAGCCATTCCCCAGCGCTTCTGCAAGCTGCGGAAGATAATGAACACCAAGACCTACCTGGAGTGGCCCATGGATGAGGCTCGGCAGGAAGGGTTTTGGGTAAATCTGAGAGCTGCGATAAAGTCTTAG

>MmusTLR2 Mus musculus Mouse BC014639 2355 ATGCTACGAGCTCTTTGGCTCTTCTGGATCTTGGTGGCCATAACAGTCCTCTTCAGCAAACGCTGTTCTGCTCAGGAGTCTCTGTCATGTGATGCTTCTGGGGTGTGTGATGGCCGCTCCAGGTCTTTCACCTCTATTCCCTCCGGACTCACAGCAGCCATGAAAAGCCTTGACCTGTCTTTCAACAAGATCACCTACATTGGCCATGGTGACCTCCGAGCGTGTGCGAACCTCCAGGTTCTGATTTTGAAGTCCAGCAGAATCAATACAATAGAGGGAGACGCCTTTTATTCTCTGGGCAGTCTTGAACATTTGGATTTGTCTGATAATCACCTATCTAGTTTATCTTCCTCCTGGTTCGGGCCCCTTTCCTCTTTGAAATACTTAAACTTAATGGGAAATCCTTACCAGACACTGGGGGTAACATCGCTTTTTCCCAATCTCACAAATTTACAAACCCTCAGGATAGGAAATGTAGAGACTTTCAGTGAGATAAGGAGAATAGATTTTGCTGGGCTGACTTCTCTCAATGAACTTGAAATTAAGGCATTAAGTCTCCGGAATTATCAGTCCCAAAGTCTAAAGTCGATCCGCGACATCCATCACCTGACTCTTCACTTAAGCGAGTCTGCTTTCCTGCTGGAGATTTTTGCAGATATTCTGAGTTCTGTGAGATATTTAGAACTAAGAGATACTAACTTGGCCAGGTTCCAGTTTTCACCACTGCCCGTAGATGAAGTCAGCTCACCGATGAAGAAGCTGGCATTCCGAGGCTCGGTTCTCACTGATGAAAGCTTTAACGAGCTCCTGAAGCTGTTGCGTTACATCTTGGAACTGTCGGAGGTAGAGTTCGACGACTGTACCCTCAATGGGCTCGGCGATTTCAACCCCTCGGAGTCAGACGTAGTGAGCGAGCTGGGTAAAGTAGAAACAGTCACTATCCGGAGGTTGCATATCCCCCAGTTCTATTTGTTTTATGACCTGAGTACTGTCTATTCCCTCCTGGAGAAGGTGAAGCGAATCACAGTAGAGAACAGCAAGGTCTTCCTGGTTCCCTGCTCGTTCTCCCAGCATTTAAAATCATTAGAATTCTTAGACCTCAGCGAAAATCTGATGGTTGAAGAATATTTGAAGAACTCAGCCTGTAAGGGAGCCTGGCCTCCTCTACAAACCTTAGTTTTGAGCCAGAATCATTTGAGATCAATGCAAAAAACAGGAGAGATTTTGCTGACTCTGAAAAACCTGACCTCCCTTGACATCAGCAGGAACACTTTTCATCCGATGCCCGACAGCTGTCAGTGGCCAGAAAAGATGCGCTTCCTGAATTTGTCCAGTACAGGGATCCGGGTGGTAAAAACGTGCATTCCTCAGACGCTGGAGGTGTTGGATGTTAGTAACAACAATCTTGACTCATTTTCTTTGTTCTTGCCTCGGCTGCAAGAGCTCTATATTTCCAGAAATAAGCTGAAAACACTCCCAGATGCTTCGTTGTTCCCTGTGTTGCTGGTCATGAAAATCAGAGAGAATGCAGTAAGTACTTTCTCTAAAGACCAACTTGGTTCTTTTCCCAAACTGGAGACTCTGGAAGCAGGCGACAACCACTTTGTTTGCTCCTGCGAACTCCTATCCTTTACTATGGAGACGCCAGCTCTGGCTCAAATCCTGGTTGACTGGCCAGACAGCTACCTGTGTGACTCTCCGCCTCGCCTGCACGGCCACAGGCTTCAGGATGCCCGGCCCTCCGTCTTGGAATGTCACCAGGCTGCACTGGTGTCTGGAGTCTGCTGTGCCCTTCTCCTGTTGATCTTGCTCGTAGGTGCCCTGTGCCACCATTTCCACGGGCTGTGGTACCTGAGAATGATGTGGGCGTGGCTCCAGGCCAAGAGGAAGCCCAAGAAAGCTCCCTGCAGGGACGTTTGCTATGATGCCTTTGTTTCCTACAGTGAGCAGGATTCCCATTGGGTGGAGAACCTCATGGTCCAGCAGCTGGAGAACTCTGACCCGCCCTTTAAGCTGTGTCTCCACAAGCGGGACTTCGTTCCGGGCAAATGGATCATTGACAACATCATCGATTCCATCGAAAAGAGCCACAAAACTGTGTTCGTGCTTTCTGAGAACTTCGTACGGAGCGAGTGGTGCAAGTACGAACTGGACTTCTCCCACTTCAGGCTCTTTGACGAGAACAACGACGCGGCCATCCTTGTTTTGCTGGAGCCCATTGAGAGGAAAGCCATTCCCCAGCGCTTCTGCAAACTGCGCAAGATAATGAACACCAAGACCTACCTGGAGTGGCCCTTGGATGAAGGCCAGCAGGAAGTGTTTTGGGTAAATCTGAGAACTGCAATAAAGTCCTAG

>MdomTLR2 Monodelphis domestica Gray Short-Sailed Opossum XM_001375753 2358 ATGAGACAAATCAGGTGGTCAGTGTGGATCTTGTGCACCATAATCAATTTCTCAGAAGAAGATGCTCCTAAGCAGACTTCTCTGTCATGTGATCCCGCTGGTGGTGTCTGTGATGGCCATTCTAGGTCTTTGGACACCATTCCTTCAGACCTGACAGAGGCTGTGAGACAGCTGGACCTGTCCTTCAACAAGATCTCACACATCAGAGAACTAGACCTTCGGAATTGTGTCAACCTGGAAGCTTTGTTGTTGCAGTCCAACCGAATTCGCTCAATAGACCCAGATTCTTTTCAGTTCCTGAGAAATCTGAAACATCTGGATTTGTCCTCCAATAGCCTATCCTACTTATCACCTTCCTGGTTTAATTCTCTCTCTTCTTTGCGGGTTCTATATTTGCATAAAAATCCCTACCAGCGACTTGGGCAAAGTCCTCTTTTTTCTCATCTTCCCAGTTTGAGGGTTCTTAAAGTGGGGAGCTATGACAGATTCTCAGAACTTCAGAAACAAGACTTTGAAGGGCTGCGTGAGCTGGAGGACCTTGAGATTGAGGCAGCTTATTTTCAGAAATATGAACCAGGGAGTCTAAAATCCATTCATAAGATAAACCACTTGGCTCTCAGCATCAGGAAGCCAAACTTACTACCTGACATCTTAGTTGACCTTGCAAGTTCTTTGCAGTATTTGGAGTTTAGAAAGACTAATCTGGGAGAATTTAATTTTTCAATGCCCCCCAATACCCCCAGCCCACTTATTGAGAAGATTATATTGAGAGAAGTGATAGTCACAGATAAGAGTTTTATTGACATTCTTAAAATGTTTTCCTATGCTTCTGAGTTGTTGGAGATTGTGCTGGAGGATTGTATACTTAATGGACTTGGCCTTTGGGAAGAGGATTTCATAAGTGAAATTCATCATGAAGTTAAAGTTGAAACAATCATCATACGGAGGTTACGGATTGGAAATTTTTTCTTATTTCGTGACCTGAGTCCTGTGTATTCATTATTACAAGGAATCAAGAGGATCACCCTCACAAGTAGCAAGGTTTTCCTTGTCCCTTGTGATCTCTCCAAAAGTCTGAAGTCTTTAGAATATTTTGATCTCAGTGACAGTTTGATGAGTGAAGGTGCTCTGGAAAATTCAGCTTGTGAGGGAGCCTGGCCTAGCCTGCACACCTTAAACTTGAGTCAAAATAAATTCGAATCACTGAAAACAATGGGGGAACTCTTGCTGACTGTGAAAAACCTGACTCACCTTGACATTAGCAAGAATAATTTGGACTCTATGCCTGACAGTTGCCAGTGGCCAGGGAAGTTGAAATATTTAAACATCTCCAGCACAAAAACCTATACGGTGACTTCCTGTATTCCCCAGACCTTGGAAATCTTAGATATCAGCAATAACCAGCTCAGCGATTTTAATTTAAATCTGCCACACCTTAAAGAGCTTTATCTTTCCAAAAATAAGCTAAAGACCCTACCAGATGCTGCTCATTTCCCCCACTTGGTGGTAATGAACATCAGTCAGAACACACTGTACACCTTCTCGAAGGCACAACTAGAATCCTTTCAGACAACGGAAATCTTGGCAGCAGGAGGCAACAACTTCATTTGCTCTTGCGAATTTCTGTCTTTTGTTCACCATGAGCAGACCCTGCTGGTCAAAGTTCTGACAGACTGGCCGGGAGGCTACCTCTGTGATGCTCCCTTCCAGGTGAGGGGCCAACGAGTGCAGGATGTCCAGCTTTCCTTCTCCGAATGCTACCGAGTGGAGCTGGTGGCTGCCATCTGCTCTTCTCTTTTCTTGCTTGCCTTGCTGGCTGGGGTTCTCTGCTACCAGTTTCATGGCATCTGGTACATGAAGATGATGTGGGCATGGCTCCAAGCCAAGAGAAAGCCCAGGAGAAACCCAGACCGGGAGATCTGCTATGATGCCTTTGTTTCCTACAGTGAAGGTGACTCACACTGGGTGGAGAACTTCATGGTCCAAGAACTGGAGAACTTTGATCCGCCTTTCAGACTGTGCCTCCACAAGAGGGACTTTGTGCCTGGCAAGTGGATCATTGACAATATCATCGACTCCATCGACAAGAGTCACAAGACACTCTTTGTGCTTTCAGAAAGCTTTGTCAAGAGTGAGTGGTGTAAGTATGAGTTGGACTTCTCCCACTTTCGTCTCTTTGATGAGAACAATGACACAGCCATTCTGATCCTCCTGGAGCCAATTGAGAAGAAGACCATTCCCCAGAGGTTCTGCAAGCTTCGAAAGATCATGAACACCAAGACGTACCTGGAGTGGCCCAGGGATGAAGGTCTGCAGGAGGAGTTTTGGTTCAACTTGAGAGCTGCCATAAAGTCATAG

>PpygTLR2 Pongo pygmaeus Orangutan ENSPPYG00000015131 2352 ATGCCACATACTTTGTGGATGGTGTGGGTCTTGGGGGTCATCATCAGCCTCTCCAAGGAAGAATCCTCCAGTCAGGCTCTGTCTTGTGACCGCAATGGTATCTGCAAGGGCAGCTCAGGATCTTTAAACTCCATTCCCTCAGGGCTCACAGAAGCTGTAAAAAGCCTTGACCTGTCCAACAACAGGATCACCTACATTAGCAACAGTGACCTACAGAGGTGTGTGAACCTCCAGGCTCTGGTGCTGACATCCAATGGAATTAACACAATAGAGGAAGATTCTTTTTCTTCCCTGGGCAGTCTTGAACATTTAGACTTATCCTATAATTACTTATCTAATTTATCGTCTTCCTGGTTCAAGACCCTTTCTTCTTTGACATTCTTAAACTTACTGGGAAATCCTTACAAAACCCTAGGGGAAACATCTCTTTTTTCTCATCTCACAAAATTGCGAATGCTGAGAGTAGGAAATATGGACACCTTCACTAAGATTCAAAGAAAAGATTTTGCTGGACTTACCTTCCTTGAGGAACTTGAGATTGATGCTTCAGATCTACAGAGCTATGAGCCAAAAAGTTTGAAGTCAATTCAGAACGTAAGTCATCTGATCCTTCATATGAAGCAGCATACTTTACTGCTGGAGATTTTTGTAGATGTTACAAGTTCCGTGGAATGTTTGGAACTGCAAGATACTGATTTGGACACTTTCCATTTTTCAGAACTATCCACTGGTGAAACAAATTCATTGATTAAAAAGTTTACATTTAGAAATGTGAAAATCACCGATGAAGGTTTGTTTCAGGTCATGAAACTTTTGAATCAGATTTCTGGATTGTTAGAATTAGAGTTTGATGACTGTACCCTTAATGGAGTTGGTAATTTTAGAGCATCTGATAATGACAGAGTTATAGATCCAGGTAAAGTGGAAACGTTAACAATTCGGAGGCTGCATATTCCAAGGTTTTACTTATTTTATGATCTGAGCACTTTATATTCACTCACAGAAAGAGTTAAAAGAATCACAGTAGAAAACAGTAAAGTTTTTCTGGTTCCTTGTTCACTTTCACGACATTTAAAATCATTAGAATACTTGGATCTCAGTGAAAATTTGATGGTTGAAGAATACTTGAAAAATTCAGCCTGTGAGGATGCCTGGCCCTCTCTACGAACTTTAATTTTAAGGCAAAATCATTTGGCATCATTGGAAAAAACCGGAGAGACTTTGCTTACTCTGAAAAACTTGACTAACCTTGATATCAGTAAGAATAGTTTTCATTCTATGCCTGAAACTTGTCAGTGGCCAGAAAAGATGAAATATTTGAACTTATCCAGCACACGAATACACAGTGTAACAGGCTGCATTCCCAAGACACTGGAAATTTTAGATGTTAGCAACAACAATCTCAATTTATTTTCTTTGAATTTGCCGCAACTCAAAGAACTTTATATTTCCAGAAATAAGTTGATGACTCTACCAGATGCCTCCCTCTTACCCATGTTACTAGTATTGAAAATCAGTAGGAATACAATAACTACTTTTTCTAAGGAGCAACTTGACTCATTTCACACACTGAAGACTTTGGAAGCTGGTGGCAATAACTTCATTTGCTCCTGTGAATTCCTCTCCTTCACTCAGGAGCAGCAAGCACTGGCCAAAGTCCTGATTGATTGGCCAGCAAATTACCTGTGTGACTCTCCATCCCATGTGCATGGCCAGCGGGTTCAGGACGTCCGCCTCTCGGTGTCGGAATGTCACAGGACAGCACTGGTGTCTGGCATGTGCTGTGCTCTGTTCCTGCTGATCCTGCTCACGGGGGTCCTGTGCCACCGTTTCCATGGCCTGTGGTACATGAAAATGATGTGGGCCTGGCTCCAGGCCAAAAGGAAGCCCAGGAAAGCTCCCAGCAGGGACATCTGCTATGATGCATTTGTTTCTTACAGTGAGCGTGATGCCTACTGGGTGGAGAACCTTATGGTCCAGGAGCTGGAGAACTTCAATCCCCCCTTCAAGTTGTGTCTTCATAAGCGGGACTTCATTCCTGGCAAGTGGATCATTGACAATATCATTGACTCCATTGAAAAGAGCCACAAAACTGTCTTTGTGCTTTCTGAAAACTTTGTGAAGAGTGAATGGTGCAAGTATGAACTGGACTTCTCCCATTTCCGTCTTTTTGATGAGAACAATGATGCTGCCATTCTCATTCTTCTGGAGCCCATTGAGAAAAAAGCCATTCCCCAGCGCTTCTGCAAGCTGCGGAAGATAATGAACACCAAGACCTACCTGGAGTGGCCCATGGATGAGGCTCAGCGGGAAGGGTTTTGGGTAAATCTGAGAGCTGCGATAAAGTCCTAG

>OanaTLR2 Ornithorhynchus anatinus Platypus Ultra539:4,343,001-4,345,358 (omAna1) 2358 ATGCAGAGACACCTCATCTGCTGTACGGGCATCCTATGGGCTCTTCTGAATCTCACCGATAAAGTCACAGCTGAACACATTTCCCTTCTCTGTGATGCCACGGGTTTCTGCAATGGGTCCTCCCTGGCTTTCAGCACCATTCCACCGGGCCTCACCGTTACAGTGCAACAGCTCGACCTGTCCAAGAACAGCATCAGACACGTCGGGAAAACAGATCTACAGAATTGTGTGAACCTGAAAGCTCTGATGCTCCAGGCCAACCAGATTGGCACAATAGATGAAGACGCCTTTCTTTCCCTGGGAATTCTAGAACTTTTGGATTTATCCAAGAATCGCCTGTCTAGTTTGTCTCCCTCCTGGTTCCAATCTCTTTCTTCCTTGCTATCCTTAAACTTATTAGAAAATCCATATCCAGACCTTGGACAAAGTCCCCTTTTTGCTCACCTCAAAAAACTGAGATCTTTAAAAGTGGGCAATGGTCAGTACTTCTCTGAGATTCGGAAACAAGATTTTGAAGGAATTTCAGATCTGGAAGAACTTGAGATTGATGGTTCAAACCTTCAGCAGTATCAACCCCACAGTTTGCAATCAACTCAGAATATAAATCACCTGCTGTTAAATGTAACCAAATCTGATATAGTGCTAAAGATTTTAAGCGATCTTACATCTTCTAAACCGAGAAATATTTCAATGTCGATCCCAGTAGCCAGTCCTCAACTAAAGAGAATTGAATTAAGACATGTCGAGATGACAGATGAAAGTGCGATTACAATTTTTAAAATGCTATCTCTTGCCGTTCGGTTGCTAGAGGTAACTCTAGAAGATGGTGTGCTTTTTGGAGTTGGAGATTGGTATGATGATTCAGGCACATGGAACGATAATTATACAGGTTCCTTAGAAACAGTCATAATTAGAAGGTTACAGATTCCAAAGTTTTTCTTATTTCGAGACATGAAACCTGTGTACTCCCTAGTAGGAAGACTCAAGACACTTACAATTGAAAATTCAAAGGTTTTCTTGGTTCCTTGTCCCTTTTCACGAAGTTTTGAATTACTGGAATATTTGGATCTTAGTGGAAATATATTATTTGATGAATTACTGAAGAATTCTGCCTGTGACGGTGCATGGCCCTCCCTACAAACCTTAAATTTAAGTCAGAATAGTTTGCGATATGTGGATCAAACGGGGGCAAGTTTAAGTACTCTGAAAAAACTGATCAATCTTGACATTAGCCAGAACAAGTTTGATTCCATGCCCGAATCTTGCCAGTGGCCAGAAAATCTTAAATATTTGAATATGTCTGGCACCCGAATTCCCAGATTGACAGACTGCATCCCTAGAACGCTTGAAATTTTAGATGTCAGTAATAATAATCTCAAAGATTTTATATTGAACCTCCCACGGCTCAAAGAACTGTACCTTTCCAAAAACAAGCTGAAGACCTTACCGGAGGGTAGGCTGTACCCAAACTTATTGGTCATGAGGATCAGCAAGAACGCACTGAATGTTTTCTCCAGGAAAGAATTTGAGTCTTTCCAGAAACTGAAGGCTCTAGAAGTTGGTGGCAACAACTTCATTTGCTCTTGTGTTTTTCTGACTTTCCTTCAGGACCAGCAGGCAATAGCCAGTGTTCTGACCGATTGGCCCGAAAACTATATATGTGATTCACCATCCCACGTGAGGGGCCAAAGAGTTCAGGATACTCATCTTCCACTTTTTGAATGCCACAGAGCTCTGTTAGTGTCCGTAATATGTTGCATTCTTTTTCTGATCATAATACTCACTGTCGCTCTCTGCCACCATTTTCATGGAATCTGGTATATGAAAATGACTTGGGCCTGGCTCCAGGCCAAAAGGAAGCCCAGAAAAACCCAGAATAAGGAAATCTACTTTGATGCTTTTGTTTCCTACAGTGAAGAAGATTCCTACTGGGTGGAAAATCTAATGGTGCAGGAACTAGAACACTCTAACCCGCCCTTTAGATTGTGTCTTCATAAAAGAGACTTTGTGCCTGGCAAATGGATCATTGACAACATCATTGACTCCATTGAAAAGAGTCACAAAACTCTGTTTGTGCTGTCAGAAAACTTCGTTAAGAGTGAGTGGTGCAAGTACGAGCTGGACTTCTCCCACTTCCGACTCTTCGATGAGAACAATGATTCTGCCATTCTAATTCTTCTGGAGCCCATCGAGAAGAAGGCAATTCCCCAAAGGTTCTGCAAGCTGCGGAAGATCATGAACACCAAGACCTACCTCAAATGGCCGTCAGATGAGACTGAGAGAGAAACATTTTGGCTAAACCTAAAATCAGCAATAAAGACAGAGCATTTCATTGAAGACTCAACTCTGTTGCACTGA

>OpriTLR2 Ochotona princeps Pika ENSOPRG00000008736 2349 ATGCCACTTGCTTTGTGGACAGCGTTGGCCCTGGGGGCCATGGTCAGCTCCTCCAAGGAAGGGTCTTTGGATCTCCTCTCTTGCGACCCCGTTGGCATCTGTGATGGGAGCTCCAGATCTTTCCAGTCCATTCCATCAGGACTTGTGGGAACCATCAAAAGCCTTGACCTGTCCAACAATGAGATCACCTCTATTGGCCACAGTGACCTGCAAAGGTGTGTGCAGCTGAGAGCTCTGAGGCTGACGTCCAATAGAATTCACACGATCGACAAAGATTCTTTCTCCTCCTTGAGCAGCCTTGAGCACTTGGACTTAGCCAGTAATCTCCTATCTAATTTATCAGCTGCCTGGTTCAGGCCTCTTTCTTCTTTGAAATTCTTAAACTTACTGGGAAACCCTTACAGAACCCTGGGGGAGACGCCTCTGTTTTCTCCCCTTGCATGTTTGCGAATCCTTCGCATAGGAAACCCAAACACCTTCATGAAGATTCAGAGGAAGGATTTTGCTGGACTCAGCTTCCTGGAGGAACTGGAGGTTGATGCCTCCAATCTCCAGGGTTATGAGCCTGAGGGCCTGAGGTCCATTCAGAACATCACTCACCTGATCCTGAACATGAGACGGCCGACTTTGCTGTTGGACATATTCCCAGACCTCTTGAGTTCTGTGGGCCACCTGGAGCTGAGAGAGACCGATCTGAACTATTTCCATTTCTCGGGGCTAACTGTCACCAAATCGAATCCCCGGATCACAAAATTCACATTCAGAAATGTGAAGATCACCGACGAGAGTGGTAATGAGGTACTGAAACTACTGACTTACATTACCGGATTATTAGAATTAGAGTTTGATGACTGTACCCTGAATGGAATTGGTGACTTTCAGGTGGTTGATAAAGACACAATTCAAGATCTAGGGAAAGTGGAAACATTAACGATACGTAACCTGCACATCCCCAAGTTCTACTTATTTTATGATATGAGTACGATCTATTCCCTCACAGAAAGACTTAAGAGAGTCACAGTAGAAAACAGCAAGGTTTTCCTGGTGCCCTGTTCATTTTCCCAACATTTGAGATCATTGGAATACTTGGATCTTGGTGAGAATTTGATGGTTGAGGAATACTTGCAAAATTCAGCCTGTGAGAATGCCTGGCCCTCCCTACACACGTTGATTTTAAGGCAAAACCATTTGACGTCGTTGGAAAGAACTGGGGAGATTCTGCTGACGCTGAGGAACCTGACTGATCTCGACGTCAGTAGGAACGGTTTCCACGCTATGCCTGACTCGTGTCAATGGCCCGAGAAGTTGGCCCGTCTGAACTTATCAACCACGCGGATTGACAGTCTGACCTCCTGCATTCCGCAGACACTGCAAGTGTTAGATGTCAGTAACAACAACCTCAATTCGTTTTCATTGACTTTGCCAAAGCTGCAGGAACTGTACATTTGCAGAAATATGCTGAAGACTCTCCCGGGGGCTGCCTCCTTACCCATGCTACTGGTGCTGAAGGTAAGCAGGAACACAATCAACACCTTCTCAAAGGAAGAGGTAGAGGCCTTCCAGCAGCTCAAGACTCTGGAAGCCGGAGGCAACAATTTCATCTGCTCCTGTGAGTTCCTGTCTTTCATGCAGGAGCAGAAAGCACTGGCCAAGGTCCTGACAGACTGGCCAGAGGGCTACCTGTGTGATTCGCCATCCCACGTGCGTGGCCAGAGGGTGCAAGACGTGCACTTGTCGGTGACTGAGTGCCACAAGGCAGCCGTGGTGTCTGGCGTGTGCTGTGCCCTCATCTTATTGATGCTGCTCACAGGGGTCCTATGCCACCGTTTGCATGGACTGTGGTACCTCAAGATGATGTGGGCCTGGTTGCAGGCCAAAAGGAAGCCCAAGAAAGCTCCCAGCAGGGACATCTGCTATGATGCGTTTGTGTCCTACAGTGAGCAGGATTCCTACTGGGTGGAGAACCTTCTGGTCCAAGAGCTGGAGAACTTCAACCCCCCATTCAGGCTGTGTCTTCACAAGCGAGACTTTGTTCCTGGCAAGTGGATCATCGATAACATCATCGACTCTATTGAGAAGAGTCACAAGACTGTGTTTGTGCTGTCCGAGAACTTTGTGCAGAGTGAGTGGTGCAAGTATGAGCTGGACTTCTCGCATTTCCGCCTCTTTGATGAGAACAATGACGCTGCTATTCTTATTCTCCTGGAGCCCATTGAGAAAAAGGCAATCCCCCAGCGCTTCTGCAAGCTGCGCAAGATCATGAACACCAGGACATACCTGGAGTGGCCCTCTGAGGAGGATCAGCACACCATGTTCTGGGTGAACTTGAGGGCTGCCATCAGGTCCTAG

>SscrTLR2 Sus scrofa Pig AY392087 2358 ATGCCATGTGCTTTGTGGACAGCATGGGTCTTGGGGATTGTAATCAGCCTCTCCAAGGAAGGAGCCCCCCATCAGGCTTCTTCTCTGTCTTGTGACCCTGCTGGTGTCTGCGACGGCCGCTCCAGATCTTTGAGCTCCATCCCCTCAGGGCTCACGGCAGCTGTGAAAAGCCTTGACCTGTCCAATAACAGGATCGCCTATGTGGGCAGCAGTGACCTGCGGAAATGCGTGAACCTCAGAGCTCTGAGGCTGGGGGCCAATAGCATTCATACCGTGGAGGAAGATTCTTTTTCTTCCCTGGGAAGTCTCGAACATCTGGACTTATCCTATAATCACTTGTCTAACTTATCATCCTCTTGGTTCAAGTCCCTTTCCACCTTGAAATTCTTAAACTTACTGGGAAATCCTTATAAAACACTCGGGGAAGCACCGCTTTTTTCTCATCTCCCAAATCTGCGAATCCTGAAAATAGGCAATAATGACACCTTCCCTGAGATTCAGGCCAAGGATTTCCAGGGGCTCACTTTTCTCCAGGAACTTGAGATTGGTGCCTCACATCTGCAGAGGTACGCGCCAAAGAGTCTGAGGTCAATTCAGAACATCAGCCATCTGATTCTTCACATGAGGCGGCCTGCTCTGCTGCCGAAGATTTTCGTAGATCTTTTAAGTTCCTTGGAATATTTAGAATTGAGAAACACTGATTTTAGCACTTTCAATTTTTCAGACGTATCCATCAATGAACACTGCACAGTGATGAAAAAATTTACGTTTCGAAAGGCAGAAATCACTGACGCAAGTTTTACGGAAATTGTGAAACTGTTAAATTATGTTTCTGGAGCCTTAGAAGTAGAGTTTGATGACTGCACCCTTAACGGACGTGGTGATCTTAGTACATCTGCTTTGGACACAATTAAATCCCTCGGTAATGTGGAGACATTAACAGTCCGGAGGTTGCATATTCCACAGTTTTTCTTGTTTTATGATCTAAGAAGTATATATTCACTCACAGGAGCAGTTAAAAGAATCACAATAGAAAACAGTAAGGTTTTTCTGGTTCCCTGTTCCCTTTCACAGCATTTAAAATCACTGGAATATTTAGATCTCAGTGAAAATTTAATGTCTGAAGAATACTTGAAAAACTCAGCCTGTGAGCATGCCTGGCCCTTCCTACACACTTTAATTTTAAGGCAAAATCATTTGAAATCATTAGAAAAAACTGGAGAAGTTTTGGTTACTCTGAAAAACCTGACTAACCTTGATATCAGTAAGAATAATTTTGATTCCATGCCGGAAACTTGCCAGTGGCCGGAAAAGATGAAATATTTGAACTTATCCAGCACGAGAATACACAGTTTAACCCATTGTCTTCCCCAGACCCTGGAAGTTTTAGATATTAGCAATAACAATCTCAACTCGTTTTCTTTGAGTCTGCCACAACTCAAAGAACTTTATATTTCCAGAAATAAGTTGAAGACGCTCCCAGATGCCTCCTTTCTACCCATGTTGTCAGTTCTGAGAATCAGCAGAAATACGATCAATACTTTCTCTAAGGAGCAACTTGATTCTTTTCAAAAGTTGAAGACTTTGGAAGCTGGAGGCAACAACTTCATCTGCTCCTGTGACTTCCTGTCCTTCACTCAGGGGCAGCAGGCGCTGGCCCAGGTGCTGAGCGACTGGCCGGAGAACTACCTGTGTGACTCTCCATCCCACGTGCGGGGCCAGCGGGTCCAGGACACCCGCCTCTCGCTCACTGAGTGCCACAGGGTGGCCGTGGTGTCCGTCGTGTGCTGTGCCCTCTTCCTGCTGCTCCTGCTCACGGGGGCTCTGTGCCACCACTTCCACGGACTGTGGTGCATGAAGATGATGTGGGCCTGGCTCCAGGCCAAGAGGAAGCCGCGGAAAGCTCCCCGCAGGGACGTGTGCTATGACGCTTTCGTGTCCTACAGTGAGCAGGACTCCTACTGGGTGGAGAACCTCATGGTCCAGGAGCTGGAGCACTTCCAGCCTCCCTTTAAGTTGTGTCTTCATAAGCGCGACTTCATTCCAGGCAAGTGGATTATTGACAACATCATCGACTCCATCGAAAAGAGCCAGAAAACCATCTTCGTGCTTTCCGAGAACTTTGTGAAGAGCGAGTGGTGCAAGTACGAGCTGGACTTCTCCCACTTCCGTCTCTTTGATGAGAACGATGACACCGCCATCCTCATTCTGCTGGAACCCATCGAGAAGAAGACCATTCCCCAGCGTTTCTGTAAGCTGCGGAAGATAATGAACACCAGGACCTACCTGGAGTGGCCCGCGGATGAGACGCAGCGGGAAGGGTTTTGGTTAAATTTGAGAGCTGCGATCAAGTCCTAG

>OcunTLR2 Oryctolagus cuniculus Rabbit AY101393 2355 ATGCCACCTGCGTTGTGGACGGTGTGGGCCTTGGGGGCTATAGTCAGCCTCCCCACGGAAGGGGCTCCTGACCTGCCCTCTCTGTCTTGTGACCCTGCCGGCATCTGCGATGGGCGCTCCAGATCCTTCCAATCCATCCCCTCGGGGCTCATGGCAACGGTGAAAAGCCTTGACCTGTCCAACAACGAGATCACCTATATCAGAGACAGTGACTTGCACAGGTGTGTGCACCTCAGGGCTCTGATGCTGATGTCCAATGGAATCGACACGATAGATGAAGATTCTTTCTCTTCCCTGGGCAGCCTCGAGCACCTGGACTTATCCAATAACCACCTATCTCATTTATCCGCCGCCTGGTTCAGGCCTCTCTCGTCCCTGAAATTCTTAAACTTGCTGGAAAACCCTTACAGAACCCTTGGGGAGACATCTCTGTTTTCTCACCTCCCACATTTGCGAATCCTGAAAGTAGGAAGCCTTTATGCCTTTGCGAATATTCGGAGGATGGATTTTGCCGGAGTCCGCTCTCTCGAGGAACTTGAGATTGATGGGTCAAACCTGCAGAGCTACGAGCCACGGGGCTTGGGATCCATCCCCAACGTGAGCCGCCTGGTTCTCCACCTGAGGCAGCCCACCTTGCTGCTCAAGCTCTTCCCAGACCTTTTAAGTTCTGTGGAGTGTCTGGAGCTGAGAGAGACTGATCTGGAAGATTTCCGTTTTTCGGGGCTGTCTGTCACCGAACCGAATCCACGGATTACCAGATTTATCTTTAGAAGTGTGAAAATCACCGATGAGAGTTGTAATGAAATACTCAAACTTCTGGCTTATGTGCCTGAGTTACTGGACTTAGAGTTTGATGACTGCACCCTTAATGGAGTTGGTGATTTTGAAGTACCAGACCGAGACCTAAGTCAAGATCTGGGTAAAGTCGAAACGTTAACAATACGGAGGTTGCACATCCCCAAGTTCTACTTATTTTACGATCTGAGTACAATCTATTCACTCTCAGAAAGGGTGAAGAGGGTCACAGTGGAAAACAGTAAGGTTTTTTTGGTTCCTTGTTCATTCTCACAACATTTAAAATCATTAGAATACTTAGATCTCAGTGAAAATCTGATGGTGGAAGAATATTTGAAAAATTCAGCCTGCGAGAAGGCCTGGCCCTCTCTGCAGACGTTAATTTTAAGGCAAAATCATTTGACATCATTAGAAAAAACCGGAGAGACCCTGCTGACGCTGAAAAACCTGACCGACCTTGACATCAGTAAGAATACTTTCCACGCCATGCCTGACACGTGTCAGTGGCCGGAAAGGTTGAGACGGCTGAACCTGTCCAGCACACGGATACACAGTTTGACGTACTGCATCCCACAGACCCTGGAAATCCTAGACGTTAGCAATAACAATCTCAACGTGTTTTCTTTGACTCTGCCCAGACTCAAAGAACTTTATATTTCCAGAAATATGTTAAAGACCCTCCCAGGTGCCTCCTTGTTACCTATGCTACTGGTCATGAAAATCAGCAGGAATTCGATAAACACTTTTTCGAAGGAGCAGCTCGAATCCTTTCAGAAGGTGACGGCCTTGGAAGCCGGAGGGAACAACTTCATCTGCTCCTGCGACTTCCTGTCTTTGACTCAGGAGCAGCCCGCGCTGACCAGGGTCCTGCCGGACTGGCCAGAGAGCTACCTGTGTGACTCCCCATCCCATGTGCGTGGCCAGCAGGTGCAGGACGCGCGCCTGTCGGTGTCCGAGTGCCACAGGGCAGCGGTGGTGTCTGGCGTGTGCTGTGCCCTCCTCCTGCTGACGCTGCTCGCCGTGGTCCTGTGCCGCCATTTCCACGGGCTGTGGTACCTGAAGATGACGTGGGCCTGGCTGCAGGCCAAGAGGAAGCCGAGGAAAGCGGCCTGCAGGGACGTGTGTTACGATGCGTTCGTGTCCTACAGCGAGCACGACTCCTACTGGGTGGAGAACCTGCTGGTGCAGGAGCTGGAGAACTCCGACCCCCCGTTCAGGCTGTGTCTGCACAAGCGGGACTTTGTGCCCGGCAAGTGGATCATTGACAACATCATCGACTGCATCGAGAAGAGCCGCAAGACCGTCTTCGTGCTCTCGCAGAACTTCGTCAAGAGCGAGTGGTGCAAGTACGAGCTGGACTTCTCCCACTTCCGCCTCTTTGATGAGAACAACGACGCCGCCATTCTGGTGCTCCTGGAGCCCATTGAGAAAAAGGCCATCCCCCAGCGCTTCTGCAAGCTGCGCAAGATCATGAACACCAGGACCTACCTGGAGTGGCCCACGGAGGAGGCTGAGCACGCCGGGTTCTGGGGGAACTTGAGGGCCGCCATAAAGTCCTAG

>RnorTLR2 Rattus norvegicus Rat AY151255 2355 ATGCTGCAAGCTCTTTGGCTCTTCTGGATCTTGATGGCTGTGATAGGCCTCTCAAGGGAAGGCCATTCTGCCCAGGCATCTCTGTCATGTGATGCTGCTGGTGTGTGTGATGGCAGCTCCAGGTCTTTCACCTCTATTCCCTCGGGACTCACAGCAAACACAAAGAAGCTTGACCTGTCTTTCAACAAGATCACCTACATTGGCCATGGTGACTTGCGAGCCTGTGTGAACCTCCGGGTTCTGACATTGGAGTCCAGCGGAATCAACACAATAGAGGGAGATGCCTTTTATTCTCTGGGCAGTCTTGAACACTTGGACTTGTCTAATAATCACCTATCTAGTTTATCTTCCTCCTGGTTCAGGCCCCTTTCCTCTTTGAAATACTTAAACTTAATGGGAAATCCTTACAGGACACTGGGGGAAACATCACTGTTCTCCAATCTCACAAATTTACAAACCCTTAGGGTAGGAAATGTTGACACTTTCAGTGAGATAAGGAGAATAGATTTTGCTGGGCTGACCTCTCTCAACGAACTTGAAATTCAGGTATTAAGTCTCGGCAACTATGAGTCCCGAAGTCTACAGTCAATTAGAGACATCTATCACCTGACCCTGCACTTGAGCGAGTCTGCTTTCCTGCTGGGGATTTTTGCAGATATTCTGAGTTCCGTGAGATATTTAGAACTAAGAGATACTAACTTGGCTAGGTTCCAATTTTCTGAACTGTCTGTAGACGAAATCAATTCGCCAATGAAGAAGCTGGCATTCCGGAATGCAGATCTCACCGATAAAAGTTTTAATGAACTCCTGAAGCTGTTGCGTTACATCTTGGAACTGATGGAGGTGGAGTTTGATCACTGCACCCTCAATGGGGTTGGTAATTTCAACCCCTCAGAGTCAGACGTAGTGAGGGAGCTAGGTAAAGTAGAAACGGTAACAATACGGAGCCTGCACATCCCCCAGTTCTATTTGTTCTATGATCTGAGCACTGTGTATTCCCTCCTGGAGAAAGTGAAGCGAATCACAGTAGAGAACAGTAAGGTCTTTCTGGTTCCCTGCTCTTTCTCACAGCATTTAAAATCATTAGAGTTCTTAGACCTCAGCGAAAATCTGATGGTTGAAGAGTATTTGAAAAACTCAGCCTGTGAGGGTGGCTGGCCTTCTCTACAAAGCTTGGTTTTGAGTCAGAATCATTTGAGATCAATACGAAAAACTGCTGAGATTTTGCTGACTCTGAAAAACCTGACAGCCCTTGACATCAGCAAGAACAGTTTTCAGCCTATGCCCGACAGTTGTCAGTGGCCAGGAAAGATGCGCTTCCTGAACTTGTCCAGTACAGGGATACAGGCCGTCAAGACATGCATTCCTCAGACCCTGGAGGTGTTGGATGTTAGTAACAACAATCTCGATTCATTTTCTTTGTTTTTGCCTCGGCTCCAAGAGCTGTATATTTCCAGAAATAAACTGAAGACACTCCCAGAAGCTTCATTGTTCCCTGTGTTACAGGTCATGAAGATCAGAGAGAACGCAATAAGTACTTTCTCTAAAGACCAGCTTGGTTCTTTTCCCAAACTGGAGACTCTGGAAGCAGGTGACAACCATTTCATCTGCTCCTGTGAACTCCTGTCCTTCATTCTGGAGAGGCCAGCCCTGGTCCATGTCCTGGTTGACTGGCCAGACAGCTACCTGTGTGATTCTCCGCCTCGCCTGCATGGCCAGAGGCTTCAGGATGCCCGGCCCTCAGTCTTGGAGTGTCACCAGGCCGCACTGGTCTCTGGAGTCTGCTGTGCCCTTCTCCTGTTGATCCTGCTCTTAGGCGCCCTGTGTTACCATTTCCATGGGCTGTGGTATCTGAGAATGATGTGGGCGTGGCTCCGGGCCAAGAGGAAGCCCAAGAAAGCTCCTTGCAGGGACCTTTGCTATGATGCCTTTGTTTCCTACAGCGAGCAGGATTCCTATTGGGTGGAGAACCTCATGGTCCAGCAGCTGGAGAACTCTGACCCACCCTTCAAGCTGTGTCTCCACAAGCGGGACTTTGTTCCGGGCAAATGGATCATTGACAACATCATTGATTCCATCGAAAAGAGCCACAAAACTGTGTTCGTGCTTTCTGAGAACTTCGTACGCAGTGAGTGGTGCAAGTATGAACTGGACTTCTCCCACTTCAGGCTCTTTGACGAGAACAATGACGCGGCCATCCTTGTTTTGCTGGAACCCATTGAGAAGAAAGCCATTCCCCAGCGCTTCTGCAAACTGCGCAAGATAATGAACACTAAGACATACCTGGAGTGGCCCTTGGATGAAGGCCAGCGGGAAGTGTTTTGGGCAAATCTGAGAACTGCAATAAAATCCTAG

>MmulTLR2 Macaca mulatta Rhesus Monkey XM_001087830 2355 ATGCCACATACTTTGTGGATGGTGTGGGTCTTGGGAGTCATCATCAGCCTCTCCAAGGAAGAATCCTCCAATCAGGCTTCTCTGTCTTGTGACCACAATGGTATTTGCAAGGGCAGCTCAGGATCTTTAAACTCCATTCCCTCAGTGCTCACAGAAGCTGTAAAATGCCTTGACCTGTCCAACAACAGGATCACCTACATTAGCAACAGTGACCTACAGAGGTATGTGAACCTCCAAGCTCTGGTGCTGACATCCAATGGAATTAACACAATAGAGGAAGATTCTTTTTCTTCCCTGGGCAGACTTGAACATTTAGACTTATCCTATAATTACTTATCTAATTTATCGTCCTCCTGGTTCAAGCCCCTTTCTTCTTTAAAATTCTTAAACTTACTGGGAAATCCTTACAAAACCCTCGGGGAAACATCTCTTTTTTCTCATCTCACAAAATTGCGAATCCTGAGAGTAGGAAATATGGACACCTTCACTAAGATTCAAAGAAAAGATTTTGCTGGACTTACCTTCCTTGAGGAACTTGAGATTGATGCTTCAGATCTACAGAGCTATGAGCCAAAAAGTTTGAAGTCAATTCAGAATGTAAGTCATCTGATCCTTCATATGAAGCAGCATATTTTACTGCTGGAGATTTTTGTAGATCTTACAAGTTCCGTGGAATGTTTGGAACTGCGAGATACTGATTTGAACACTTTCCATTTTTCAGAACTATCCACTGGTGAAACAAATTCATTGATTAAAAAGTTTACATTTAGAAATGTGAAAATCACCGATGAAAGTTTGTTTCAAGTCATGAAACTTTTGAGTCAGATTTCTGGATTGTTAGAATTAGAGTTTGATGACTGTACCCTTAATGGAGTTGGTGATTTTAGAGGATCTGATAATGACAGAGTTATAGATCCAGGTAAAGTGGAAACATTAACAATCCGGAGGCTGCATATTCCACAGTTTTACTCATTTAATGATCTGAGCACTTTATATCCACTCACAGAAAGAGTTAAAAGAATCACAGTAGAAAACAGTAAAGTTTTTCTGGTTCCTTGTTTACTTTCACGACATTTAAAATCATTAGAATACTTGGATCTAAGTGAAAATTTGATGGTGGAAGAATACTTGAAAAATTCAGCCTGTGAGGATGCCTGGCCCTCTCTACAAACTTTGATTCTAAGGCAAAATCACTTGGCATCATTGGGAAAAACCGGAGAGACTTTGCTTACTCTGAAAAACTTGACTAACCTTGATATCAGTAAGAATACTTTTCATTATATGCCTGAAACTTGTCAATGGCCAGAAAAGATGAAATATTTGAACTTATCCAGCACACGAATACACAGTGTAACAGGCTGCATTCCCAAGACACTGGAAATTTTAGATATTAGCAACAACAATCTCAATTTATTTTCTTTGAATTTGCCGCAACTCAAAGAACTTTATATTTCCAGAAATAAGTTGATGACTCTACCAGATGCCTCCCTCTTACCCATGTTACTAGTGTTGAAAATCAGTAGGAATACAATAACGACATTTTCTAAGGAGCAACTTGACTCTTTTCACACATTGAAGACTTTGGAAGCTGGTGGCAATAACTTCATTTGCTCCTGTGAATTCCTGTCCTTCACTCAGGAGCAGCAAGCACTGGCCAAAGTCCTGGCTGATTGGCCAGCAAATTACCTGTGTGACTCTCCATCCCATGTGCGTGGCCAGCGGGTTCAGGATGTCCGCCTCTCAGTGTCGGAATGTCACAGGGCAGCACTGGTGTCTGGCATGTGCTGTGCTCTGTTCCTGCTGATCCTGCTCATGGGGGTCCTGTGCCACCGTTTCCACGGCCTGTGGTACATGAAAATGATGTGGGCCTGGCTCCAGGCCAAAAGGAAGCCCAGGAAAGCTCCCAACAGGGACATCTGCTATGATGCGTTTGTTTCTTACAGTGAGCGGGATGCCTACTGGGTGGAGAACCTTATGGTCCAGGAGCTGGAGAACTTCAATCCCCCCTTCAAGTTGTGTCTTCATAAGCGGGACTTCATTCCTGGCAAGTGGATCATTGACAATATCATTGACTCCATTGAAAAGAGCCACAAAACTGTCTTTGTGCTTTCTGAAAACTTTGTGAAGAGTGAGTGGTGCAAGTATGAACTGGACTTCTCCCATTTCCGTCTTTTTGATGAGAACAATGATGCTGCCATTCTTGTTCTTCTGGAGCCCATTGAGAAAAAAGCCATTCCCCAGCGCTTCTGCAAGCTGCGGAAGATAATGAACACCAAGACCTACCTGGAGTGGCCCATGGATGAGGCTCGGCAGGAAGGGTTTTGGGTAAATCTGAGAGCTGCGATAAAGTCTTAG

>PtroTLR2P Pan troglodytes Chimpanzee chr4:157,851,337-157,852,026 (panTro2) 690

ACTTTCTCTAAAGGGCAACTTGACTCTTTTCACACACTGAAGACTTTGGAAGCTGGTGGC

AACAACTTCATTTGCTCCTGTGAATTCCTCTCCTTCACTCAGGAGCAGCAAGCACTGGCC

AAATTTCTTATCGGTTGACCAGCAAACTACCTGTGTGACTCTCCATCCCACGTGTGTGGC

CAACAGGTTCAGGATGTCTGCCTTTTGGTGTCGGAGTGCCAAAGGGTGGCACTGGTGTCT

GGCATGTGCTGTGCCCTGTTCCTGCTGATCCTGCCCATGGGGGTTCTGTGCCACTGTTTC

CACAGCCCATGGTACATGAAAATGATGTGGGCCTGGCTCTAGGCCAAAAGGAAGCACAGG

AAAGCTTGCAGCAGGGACATCTGCTATGACGCATTTGTTTCTTATAGTGAGCAGGATGCC

TACTGGGTGGAGAACCTTATGGTCCAGGAGCTGGAGAACTTCAATCCCCTCTTCAAGTTG

TGTCTTCATAAGTGGGACTTCATTCCTGGCAAGTGGATCATTGACAATATCATTGACTCC

ATCGAAAAGAGACACAAAACTGTCTTTGTGCTTTCTGAAAACTTTGTGAAGAGTGAGTGG

TGCAAGAAGAAACTGGACTTTTCCCATTTCCGTCTTTTTGATGAGAACAATGATGCTGCC

ATTCTCATTCTTCTGGAGCCCATTAAGAAG

>HsapTLR2P Homo sapiens Human ENSG00000250771 690

ACTTTCTCTAAAGGGCAACTTGACTCTTTTCACACACTGAAGACTTTGGAAGCTGGTGGC

AACAACTTCATTTGCTCCTGTGAATTCCTCTCCTTCACTCAGGAGCAGCAAGCACTGGCC

AAAGTTCTTATCGGTTGGCCAGCAAACTACCTGTGTGACTCTCCATCCCACGTGTGTGGC

CAACAGGTTCAGGACGTCTGCCTTTTGGTGTCGGAGTGCCACAGGGTGGCACTGGTGTCT

GGCATGTGCTGTGCCCTGTTCCTGCTGATCCTGCCCATGGGGGTTCTGTGCCACTGTTTC

CACAGCCCGTGGTACATGAAAATGATGTGGGCCTGGCTCTAGGCCAAAAGGAAGCACAGG

AAAGCTTGCAGCAGGGACATCTGCTATGACGCATTTGTTTCTTATAGTGAGCAGGATGCC

TACTGGGTGGAGAACCTTATGGTCCAGGAGCTGGAGAACTTCAATCCCCTCTTCAAGTTG

TGTCTTCATAAGTGGGACTTCATTCCTGGCAAGTGGATCATTGACAATATCATTGACTCC

ATTGAAAAGAGACACAAAACTGTCTTTGTGCTTTCTGAAAACTTTGTGAAGAGTGAGTGG

TGCAAGAAGGAACTGGACTTCTCCCATTTCCGTCTTTTTGATGAGAACAGTGATGCTGCC

ATTCTCATTCTTCTGGAGCCCATTAAGAAG

>PpygTLR2P Pongo pygmaeus Orangutan chr4:159,471,113-159,471,801 (ponAbe2) 689

ACTTTCTCTAAAGGGCAACTTGACTCTTTTCACACACTGAAGACTTTGGAAGCTGGTGGC

AACAACTTCATTTGCTCCTGTGAATTCCTCTCCTTCACTCAGGAGCAGCAAGCACTGGCC

AAAGTTCTTATCGGCTGGCCAGCAAACTACCTGTGTGACTCTCCATCCCATGTGTGTGGC

CAACAGGTTCAGGACGTCTGCCTTTTGGTGTCAGAGTGCCACAGGGTGGCACTGGTGTCT

GGCATGTGCTGTGCCCTGTTCCTGCTGATCCTGCCCACAGGGGTTCTGTGCCACTGTTTC

CACAGCCTGTGGTACATGAAAATGATGTGGGCCTGGCTCCAGGCCAAAAGGAAGCACAGG

AAAGCTCCCAGCAGGGACATCTGCTATGACGCATTTGTTTCTTACAGTGAGTGGGATGCC

TACTGGGTGGAGAACCTTATGGTCCAGGAGCTGGAGAACTTCAATCCCCCCTCCAAGTTG

TGTCTTCATAAGCGGGACTTCATTCCTGGCAAGTGGATCATTGACAATATCATTGACTCC

ATTGAAAAGAGCCACAAAACTGTCTTTGTGCTTTCTGAAAACTTTGTGAAGAGTGAGTGG

TGCAAGAATGACCTGGACTTCTCCATTTCCGTCTTTTTGATGAGAACAATGGTGCTGCCA

TTCTCATTCTTCTGGAGCCCATTAAGAAG

>CjacTLR2P Callithrix jacchus Marmoset chr3_GL284948_random:24,514-25,200 (calJac3) 687

TCAAAAGGGGAACTTGACTCTTTTCACACACTGAAGACTTTGGAAGCTGGTGGCAATAAC

TTCATTTGCTCCTTCCTCTCCTTCACTCAGGAGCAGCACCAAAGTCCTGAGTGATTGGCA

AACTACCTGTGTGACTCTCCATCCCACATGTGCGGCCAGCGGGTTGAGGATGTCCACCTC

TTGCTGTCGGAATGCCACAGGGTGGCACTGGTGTCTGGCACGTGCTGTGCCCTGTTCCTG

CTGATCCTGCTCATGGCGGTTCTGTGCCACCGTTTCCATGGCCTGTGGTATGTATTTTCA

TGTACTATCATGTACTGTGATGTGAGCTTGGCTCCAGGCCAAAAGGAAGCCCAGGAAAGC

TTCCAGCAGGGACATTTGCTATGATGCGTTTGTTTCTTACAGTGAGCGGGATGCCTACTG

GGTGGAGAACCTTATGGTCCAGGAGCTGGAGAACTTCAACCCCCTTCAAGTTGTGTCTTC

ATAAGACGGACTTCATTCCTGGCAAGTGGATCATTGACAATATCATTGACGCCATTGAAA

AGAGCCATGAAACTTTGTGCTTCTGAAAACTTTATGAAGAATGAGTGGTGCAATGAACCA

CTCACTGGACTGGACTTCTCCCATTTTCGTCTTTTTGATGAGAACAATGATGCTGCCGTT

CTCGTTCTTCTGGAGTTCACTGAGAAA

>MmulTLR2P Macaca mulatta Rhesus Monkey chr5:145,888,130-145,888,813 (rheMac2) 684

ACTTTCTCTAAAGGGCAACTTGACTCTTTTCACACACTGAAGACTTTGGAAGCTGGTGGC

AACAACTTCATTTGCTCCTGAGAATTCCTCTCCTTCACTCAGGAGCAGCAAGCGCTGGCC

AAAATTCTTATCGGTTGGCCAGAAAACTACCTGTGTGACTCTCCATCCCATGCGCACGGC

CAACAGGTTCATGACGTCCGTCTCTTGGTGTTGGAGTGCCATAGGGCAGCACTGGTGTCT

GGCATGTGCCGTGCCTTGTTCCTGCTGATCCTGCTCATGGGGGTCCTGTGCCACCGTTTC

CAAGGCCTGTGGTACATGCAAATGATGTGGGCCTGGCTCCAGGCCAAAAGGAAGCCCAGG

AAAGCTCCCAACAGGGACATCTGCTATGATGCAGTTTCTCATAGTGAGCAAGATACCTAC

TGGGTGGAGAACCTTATGGTCCAGGAGCTGGAGAACTTCAATCCCCCCTTCAAGTTGTGT

CTTCATAAGCGGGACTTCATTCCCGGCAAGTGGATCATTGACAATATCATTGACTCCACT

GAAAAGAGCCACAAAATGTTGTGTTTTCTGAAATTTTTTGTGAAGAGTCAGTGGTGCAAG

TATGAACTGGACTTCTCCCATTTCCGTCTTTTTGATGAGAACAATGATATTGCCATTCTC

CTTCTTCTGGAGCCCATTGAGAAG

>EcabTLR2P Equus caballus Horse chr2:80,156,948-80,157,649 (equCab2) 702

TTCTCTAAGGAGCAACTTGATTCTTTTCAAGAACTGAAGACTTTGGAAGCCGGTGGCAAC

AATTTCATTTGCTCCTGTGAGTTCCCGTCTTTCACTCAGGAGGAGCAGACACTGGACCAG

ATCCTGATCGACTGGCCAGAAAACTACCTGTGTGACTCTCCCTCCCACGTGCGGGGCCAG

CGGGTTCAGGACACTCGTCTCTCGGTCTCTGAATGCCACAGGACAGCTCTGGTGTCTGCT

GTGTGCTGTGCCCTTTTCCTGTCGATCCTGCTCACTGGGGTTCTGTGTCACCATTTCCAT

GGACTGTGGTACATGAAAATGATGTGGGCCTGGCTCCAGGCCAAAAGGAAGCCCAGGACA

GCTCCCCAAAGGGACATCTGTTATGACGCCTTCGTGTCTTACAGTGAACGGGACTCCTAC

TGGGTGGAGAACCTCATGGTGCAGGAGCTGGAGCACTTCAACCCTCCCTTTAAGTTGTGT

CTTCATAAGCGGGACTTTATTCCTGGCAAATGGATTATTGACAATATCATTGACTCGATT

GAAAAGAGCCACAAAACCATCTTTGTGCTTTCTGAAAACTTTGTGAAGAGTGAGTGGTGT

AAGTACGAACTGGACTTCTCCCATTTTCGTCTCTTTGATGAGAACAATGATGCTGCCATT

CTCATTCTTCTGGAGCCCATTGACAAAAAGGCCATTCCCCAG

>CfamTLR2P Canis familiaris Dog chr15:54,474,215-54,474,652 (canFam2) 438

ACTTTCTCGAAGGAGCAACTGGATTCCTTTCACAAGCTGCAGACCCTGGAGGCCGGTGGC

AACAACTTCCTTTGCTCCTGTGAATTCCTGTCTTTCACTCAGGAGCAGCAGGCCCTGGCC

GGGCTCCTGGTCAGCTGGCCAGAGGACTACCTGTGTGACTTGGCTCCCGGCTTCTGAGTT

CCACTGGGTGGCTCTGGTGGCCCCTGTGCTGTGTCCTGCTCCTGCTGGCCCAGGGGGCTC

CTCCCCCGCCCCCCAGACATCTGTTATGATGCCTTTGCTTTACAGGGAGCAGGATGCCTA

CTGGGTGGAGCACCTTCTGGTCCAGGAGCTGGAGCACTTCCACCCTGGGACTTTATTCCT

GGCAAATGGATTATTGACAATATCACTGACTCCATCAAGAAGAGCCACAAAACCATCTTT

GAGCTTTCTGAAAACTTC
